# Progression of pathology in PINK1-deficient mouse brain from splicing via ubiquitination, ER stress, and mitophagy changes to neuroinflammation

**DOI:** 10.1186/s12974-017-0928-0

**Published:** 2017-08-02

**Authors:** Sylvia Torres-Odio, Jana Key, Hans-Hermann Hoepken, Júlia Canet-Pons, Lucie Valek, Bastian Roller, Michael Walter, Blas Morales-Gordo, David Meierhofer, Patrick N. Harter, Michel Mittelbronn, Irmgard Tegeder, Suzana Gispert, Georg Auburger

**Affiliations:** 1 Experimental Neurology, Goethe University Medical School, 60590 Frankfurt am Main, Germany; 2 Institute of Clinical Pharmacology, Goethe University Medical School, 60590 Frankfurt am Main, Germany; 3 Edinger-Institute (Institute of Neurology), Goethe University Medical School, 60590 Frankfurt am Main, Germany; 4 Institute for Medical Genetics, Eberhard-Karls-University of Tuebingen, 72076 Tuebingen, Germany; 5 Department of Neurology, University Hospital San Cecilio, 18012 Granada, Spain; 6 Max Planck Institute for Molecular Genetics, Ihnestraße 63-73, 14195 Berlin, Germany; 7 Luxembourg Centre of Neuropathology (LCNP), Luxembourg, Luxembourg; 8 Department of Pathology, Laboratoire National de Santé, Dudelange, Luxembourg; 9 Luxembourg Centre for Systems Biomedicine (LCSB), University of Luxembourg, Esch-sur-Alzette, Luxembourg, Luxembourg; 10 Department of Oncology, Luxembourg Institute of Health, NORLUX Neuro-Oncology Laboratory, Luxembourg, Luxembourg

**Keywords:** Parkinson’s disease, Ubiquitin kinase PINK1, Mitochondrial dysfunction, Antiviral response, Neuroinflammation

## Abstract

**Background:**

PINK1 deficiency causes the autosomal recessive PARK6 variant of Parkinson’s disease. PINK1 activates ubiquitin by phosphorylation and cooperates with the downstream ubiquitin ligase PARKIN, to exert quality control and control autophagic degradation of mitochondria and of misfolded proteins in all cell types.

**Methods:**

Global transcriptome profiling of mouse brain and neuron cultures were assessed in protein-protein interaction diagrams and by pathway enrichment algorithms. Validation by quantitative reverse transcriptase polymerase chain reaction and immunoblots was performed, including human neuroblastoma cells and patient primary skin fibroblasts.

**Results:**

In a first approach, we documented *Pink1*-deleted mice across the lifespan regarding brain mRNAs. The expression changes were always subtle, consistently affecting “intracellular membrane-bounded organelles”. Significant anomalies involved about 250 factors at age 6 weeks, 1300 at 6 months, and more than 3500 at age 18 months in the cerebellar tissue, including *Srsf10*, *Ube3a*, *Mapk8*, *Creb3*, and *Nfkbia*. Initially, mildly significant pathway enrichment for the spliceosome was apparent. Later, highly significant networks of ubiquitin-mediated proteolysis and endoplasmic reticulum protein processing occurred. Finally, an enrichment of neuroinflammation factors appeared, together with profiles of bacterial invasion and MAPK signaling changes—while mitophagy had minor significance. Immunohistochemistry showed pronounced cellular response of Iba1-positive microglia and GFAP-positive astrocytes; brain lipidomics observed increases of ceramides as neuroinflammatory signs at old age.

In a second approach, we assessed PINK1 deficiency in the presence of a stressor. Marked dysregulations of microbial defense factors *Ifit3* and *Rsad2* were consistently observed upon five analyses: (1) *Pink1* ^−/−^ primary neurons in the first weeks after brain dissociation, (2) aged *Pink1* ^−/−^ midbrain with transgenic A53T-alpha-synuclein overexpression, (3) human neuroblastoma cells with *PINK1*-knockdown and murine *Pink1* ^−/−^ embryonal fibroblasts undergoing acute starvation, (4) triggering mitophagy in these cells with trifluoromethoxy carbonylcyanide phenylhydrazone (FCCP), and (5) subjecting them to pathogenic RNA-analogue poly(I:C). The stress regulation of *MAVS*, RSAD2, DDX58, IFIT3, IFIT1, and *LRRK2* was PINK1 dependent. Dysregulation of some innate immunity genes was also found in skin fibroblast cells from PARK6 patients.

**Conclusions:**

Thus, an individual biomarker with expression correlating to progression was not identified. Instead, more advanced disease stages involved additional pathways. Hence, our results identify PINK1 deficiency as an early modulator of innate immunity in neurons, which precedes late stages of neuroinflammation during alpha-synuclein spreading.

**Electronic supplementary material:**

The online version of this article (doi:10.1186/s12974-017-0928-0) contains supplementary material, which is available to authorized users.

## Background

Parkinson’s disease (PD) is the second most prevalent neurodegenerative disease after Alzheimer’s disease (AD). An autosomal recessive variant of PD, PARK6, is caused by loss-of-function mutations in PINK1 (PTEN-induced kinase 1) [1]. PINK1 activates ubiquitin by phosphorylation [2–4] and exerts quality control over mitochondria, controlling their translational repair, fusion, and elimination by mitophagy [5–9]. In this role, it cooperates with the downstream ubiquitin ligase PARKIN, which is responsible for another autosomal recessive variant of PD, named PARK2 [10–12].

Independent from their role for mitochondria, PINK1 and PARKIN are transcriptionally induced by trophic and nutrient deprivation stress [13, 14], and they influence the trophic cell state through modulation of the signaling from glial cell line-derived neurotrophic factor (GDNF) and its receptor tyrosine kinase RET [15–17]. Unexpectedly, PARKIN was recently discovered to modulate also the cellular resistance against microbial invasion [18].

Thus, in spite of the deep knowledge about the roles of PINK1 and PARKIN for selective mitophagy, there is an important need to conduct unbiased surveys to elucidate additional functions of PINK1 and PARKIN in stress responses. Our previous OMICS work observed only mild changes in mRNA, protein abundance and posttranslational modifications in response to PINK1 loss-of-function, failing to identify individual biomarkers that correlate with disease progression [19–22].

Transcriptome dysregulations below 1.5-fold are usually regarded with skepticism and are difficult to validate with other experimental techniques. However, in age-associated neurodegenerative disorders, they cannot be disregarded. It is known that the 1.5-fold increase of beta-amyloid precursor protein (APP) dosage on chromosome 21 leads to typical AD symptoms and neuropathology already by the age of 40 years in most Down syndrome cases [23], so the triggers of any AD manifestation at ages 60–90 must be equivalent to an APP gene dosage much smaller than 1.5-fold. Similarly, the manifestation of PD is triggered by alpha-synuclein around age 35 years via a twofold gene dosage increase [24], around age 50 via a 1.5-fold gene dosage [25], and around age 70 by a 1.3-fold gene dosage [26, 27]. In practically all chromosomal trisomies, the 1.5-fold increase in dosage of some genes results in embryonal lethality, so any old-age pathology triggered from these genes would result from <1.5-fold expression dysregulation.

The relevance of subtle expression changes has been taken into account by modern analysis tools based on “gene set enrichment analyses” [28]. Therefore, we now re-investigated our global transcriptome data with automated biomathematics tools, accepting that some false positive and false negative results will have to be dealt with, but hoping that the significant enrichment of pathways and subcellular compartments will identify PINK1-deficiency effects that are validated by the consistency over time as well as across tissues and species.

For this aim, we (1) analyzed the global transcriptome profile of *Pink1* ^−/−^ mouse brain tissue at three ages, (2) surveyed *Pink1*-dependent regulations of the global transcriptome in neuron-rich primary cultures from postnatal mouse brain at 12 days after the acute brain dissection stress, (3) validated the results in aged *Pink1* ^−/−^ brain where the transgenic overexpression of A53T-alpha-synuclein (gene symbol *SNCA*) exerted chronic neurotoxic stress, (4) tested the cellular response of microglia and astrocytes in *Pink1* ^−/−^ brain by immunohistochemistry, (5) used lipidomics to study pro-inflammatory signals, (6) performed a systematic assessment of the expression of key factors of antiviral state in human neuroblastoma cells with lentiviral *PINK1*-Knock-Down (KD), studying the time course after acute starvation stress, (7) re-assessed the same key factors of antiviral state in human *PINK1*-(KD) neuroblastoma versus *Pink1* ^−/−^ murine embryonal fibroblasts after mitophagy via FCCP drug treatment, and (8) tested the same factors after stress with the pathogenic poly(I:C) RNA regarding PINK1-dependent expression regulation. Primary skin fibroblasts from three patients at advanced age with manifest PD due to G309D-PINK1 mutations were employed to assess the relevance of these data for the human disease.

## Methods

### Mouse breeding and brain dissection

*Pink1* ^−/−^ and wildtype (WT) control mice, which were derived from common ancestors and share the strain 129/SvEv genetic background, were bred and genotyped as previously reported [29]. Brain tissue from *Pink1* ^−/−^+A53T-SNCA double mutant mice was obtained as published [20].

### Global transcriptomics

Affymetrix oligonucleotide microarray profiling was performed with Genechip mouse genome 430 2.0 arrays as previously [30, 31], using cRNA from the brain cerebellar tissue as reported before [20] and from neuron-rich primary cultures from 3 *Pink1* ^−/−^ versus 3 age and sex-matched WT control mice. Hybridization occurred on Affymetrix Genechip mouse genome 430 2.0 arrays, which represent 39,000 transcripts, of which more than half are anonymous or poorly understood, according to PubMed and GeneCards database searches. The biomathematical analysis was performed in the institute for medical genetics at Tuebingen University.

### Bioinformatic analyses

For protein-protein interaction (PPI) network analysis, the software tool String v.10 (https://string-db.org/) with standard settings has been employed to visualize networks of significant dysregulations [32]. As recommended, gene symbols of factors with significant dysregulation were entered into the Multiple Proteins window with the *Mus musculus* option, the matching of the input with the correct factors was accepted, and the graphic interaction diagram was generated and archived. The Analysis button was used to generate automated network statistics; significant functional enrichments of GO (Gene Ontology) terms and KEGG pathways were exported into EXCEL files.

For an additional comprehensive transcriptome analysis, gene set enrichment analysis (GSEA, v2.2.3, http://software.broadinstitute.org/gsea/index.jsp) [28] was applied in order to see, if a priori defined sets of genes show statistically significant, concordant differences between mutant/WT in 18 months old cerebellum samples. For every gene, only the one entry with the lowest adjusted *p* value and the according log_2_ transformed ratio was taken. GSEA default settings and Reactome v5.2 and KEGG v5.2 gene set database were used. Pathways with *p* value ≤0.05 and FDR *q* ≤ 0.25 were regarded as significant. Heat maps were produced with the Perseus software.

### Primary neuron culture

Neuron-rich primary cultures from the dissociated brain cerebral cortex of postnatal mice were prepared as previously described [33]. In short, 500,000 cells per well were seeded on a 0.01% (*w*/*v*) poly-d-lysine coated 6-well plate. In order to limit the growth of dividing cells, cytosine ß-D-Arabinoside was added on the second day of culture as before. The 3 plate pairs (mutant versus WT), where many singular neurons in homogeneous density with a dense network of processes contrasted with very few astrocytes and microglia present, were chosen out of a total of 12 plate pairs on culture day 12 for RNA extraction.

### Brain homogenate from aged mice

Mouse aging and dissection of mice was carried out as before, employing cerebellum from single mutant *Pink1* ^−/−^ at three ages (10 mutants versus 10 WT) and midbrain from adult double mutant *Pink1* ^−/−^+A53T-SNCA mice at age 18 months (5 mutants versus 5 WT) for the extraction of global RNA and cDNA synthesis [20].

### GFAP and Iba1 immunohistochemistry

Immunohistochemistry was performed using an automated staining system (Leica Bond-III, Nussloch, Germany). The following antibodies were used: anti-GFAP, rabbit polyclonal antibody, dilution 1:14,000 (DakoCytomation, Glostrup, Denmark); anti-Iba1, rabbit antibody, dilution 1:1000 (Wako Pure Chemical Industries, Osaka, Japan).

Representative areas of striatum, substantia nigra, neocortex, and brainstem of *Pink1* ^−/−^ (*n* = 1) and wild-type (*n* = 1) animals were analyzed using ImageJ software (Version 1.51 h; National Institutes of Health, Bethesda, Maryland, USA). We quantified positive cells in relation to all cells.

### Tissue preparation for lipid analysis

Matched pairs of male and female *Pink1* ^−/−^ and *Pink1* ^+/+^ mice were used for analysis of bioactive lipids in brain tissue. Mice were 9–13 months (four each), 17 months (three each), and 21 months (four each) old at the time of tissue preparation (mean age 15.5 −/− and 17 +/+). Mice were sacrificed by carbon dioxide. Blood was drawn into K+ EDTA microvettes (Sarstedt) for plasma analysis by cardiac puncture. Subsequent intracardial perfusion with saline removed rests of blood. The lumbar spinal cord, olfactory bulb, and hippocampus were dissected, and tissue pieces of 3–5 mg were excised, rapidly frozen in liquid nitrogen, and stored at −80 °C until analysis. The precise tissue weight was determined on precision scales directly before tissue homogenization.

### Analysis of lipid signaling molecules

Sphingolipids were analyzed by liquid chromatography tandem mass spectrometry (LC-MS/MS) in different regions of the nervous system (olfactory bulb, hippocampus, spinal cord) at three different ages (8.5–12.5 weeks, 17.5 weeks, and 21 weeks). LC-MS/MS analyses were done on an API4000 triple quadrupole mass spectrometer equipped with an APCI (atmospheric pressure chemical ionization) ion source for the analysis of ceramides and with an ESI (Electrospray Ionization) ion source for the analysis of sphingosines, Glu-Cer/Lac-Cer (Sciex, Darmstadt, Germany) [34, 35]. All quadrupoles were working at unit resolution. Concentrations of the calibration standards, quality controls, and samples were evaluated by MultiQuant 3.0 (Sciex, Darmstadt, Germany) using the internal standard method (isotope-dilution mass spectrometry). Calibration curves were calculated by linear regression with 1/x weighting. The coefficient of correlation for all measured sequences was at least 0.99.

Tissue pieces of approximately 4 mg were homogenized in 200-μl extraction buffer (50 μl per mg). For analysis of sphingolipids, 20 μl plasma or homogenized tissue samples consisting in 0.4 mg tissue were extracted with methanol:chloroform:HCl (15:83:2) after spiking with the respective internal standards, which was Cer17:0 for ceramides and sphingosine-D7 and sphingosine-1-phosphate-D7 for sphingosines. A Luna C18 column (150 mm × 2 mm ID, 5 μm particle size, 100 Å pore size; Phenomenex, Aschaffenburg, Germany) was used for chromatographic separation. The HPLC mobile phases consisted of water-formic acid (100:0.1, *v*/*v*) (A) and acetonitrile-tetrahydrofuran-formic acid (50:50:0.1, *v*/*v*/*v*) (B). For separation, a gradient program was used at a flow rate of 0.3 ml/min. The initial buffer composition was 60% (A)/40% (B). It was maintained for 0.6 min, then linearly changed to 0% (A)/100% (B) over 3.9 min, and held for 6.5 min. Subsequently, the ratio was linearly changed back within 0.5 min to 60% (A)/40% (B) and then held for another 4.5 min. The running time for every sample was 16 min. The injection volumes were 15 μl for ceramides and 10 μl for sphingosines. The analyses were done in multiple reaction monitoring (MRM) mode. For every analyte, two transitions were recorded, one for quantification and the other for identification. The peak area of the analyte was normalized for the peak area of the internal standard. Precursor to product ion transitions for quantification were: *m/z* 539 → 264 for C_16:0_-Cer, *m/z* 567 → 264 for C_18:0_-Cer, *m/z* 595 → 264 for C_20:0_-Cer, *m/z* 651 → 264 for C_24:0_-Cer, *m/z* 649 → 264 for C_24:1_-Cer, *m/z* 700 → 264 for C_16:0_-GluCer, *m/z* 729 → 264 for C_18:0_-GluCer, *m/z* 862 → 264 for C_16:0_-LacCer, *m/z* 891 → 264 for C18:0-LacCer, *m/z* 973 → 264 for C24:1-LacCer, *m/z* 553 → 264 for C17:0-Cer, *m/z* 300 → 282 for Sph and *m/z* 380 → 264 for S1P. The dwell times were 15 or 50 ms.

To assess genotype-dependent differences on individual ceramides in specific regions, mice of different ages were summarized. To assess progression over age, ceramides across regions were pooled, log2-transformed to linearize the data, and subsequently summed to get a global readout for all ceramides. Total ceramides were then plotted over time and genotype-dependent differences at different ages were analyzed using two-way ANOVA for “genotype x age”.

### Neuroblastoma starvation

The human SH-SY5Y neuroblastoma cell line with dopaminergic properties was stably transduced by lentivirus either with a control (NT for Non-Target knock-down) shRNA or a shRNA directed against *PINK1* and maintained under puromycin (1 μg/ml) selection in RPMI medium containing 10% Fetal Calf Serum (FCS), as published already [6]. These *PINK1*-KD and NT control cell lines had the stability of their KD controlled repeatedly over many months. They were switched to HBSS medium without FCS, to subject them to a starvation time course as previously described [13].

### Quantitative reverse transcriptase real-time polymerase chain reaction (qPCR)

RNA was isolated with the RNeasy mini kit (Qiagen) and then treated with DNase I. cDNA was synthesized with SuperScript III reverse transcriptase using oligo(dT)_20_ and random primers (Invitrogen). cDNA from 20 to 25 ng RNA were utilized in a 20 μl reaction volume using the StepOnePlus Real-Time PCR System and the appropriate murine (lowercase) or human (uppercase) TaqMan gene expression assays (Applied Biosystems): for mouse *Pink1* (Mm00550827_m1), *Creb3* (Mm00501607_m1), *Ddx58* (Mm01216853_m1), *Hebp1* (Mm00469161_m1), *Ifit1* (Mm00515153_m1), *Ifit3* (Mm01704846_s1), *Irf3* (Mm00516784_m1), *Mapk8* (Mm01218957_m1, Mm01218946_m1, Mm00489514_m1), *Mapk9* (Mm00444239_m1), *Mapk14* (Mm01301009_m1), *Mavs* (Mm00523170_m1), *Mfn1* (Mm00612599_m1), *Nfkbia* (Mm00477798_m1), *Rsad2* (Mm00491265_m1), *Srsf10* (Mm01193320_m1), *Tbk1* (Mm00451150_m1), *Tnf* (Mm00443258_m1), for human *PINK1* (Hs00260868_m1), *DDX58* (Hs01061436_m1), *HEBP1* (Hs00211123_m1), *IFIT1* (Hs03027069_s1), *IFIT3* (Hs00155468_m1), *IRF3* (Hs01547283_m1), *LRRK2* (Hs00411197_m1), *MAVS* (Hs00920075_m1), *MFN1* (Hs00966851_m1), *RSAD2* (Hs00369813_m1), *SQSTM1* (Hs00177654_m1), *TBK1* (Hs00179410_m1). mRNA expression was normalized to the TATA binding protein gene expression or the Hypoxanthine Phosphoribosyltransferase 1 gene expression (*Tbp*: Mm00446973_m1, *TBP*: Hs99999910_m1, *HPRT1:* Hs99999909_m1). Relative expression changes were calculated with the 2^−ΔΔCt^ method [36].

### Triggering mitophagy via treatment with FCCP

The drug FCCP, which is known to uncouple the mitochondrial membrane gradient and trigger mitophagy [37], was administered over 24 h at 10 μM concentration to human SH-H5Y neuroblastoma cells or murine embryonal fibroblasts, which had been cultured in DMEM plus 10% FCS and grown to confluency (approximately 4 × 10^6^ cells) in T25 flasks as previously described [38]; then, the cells were collected, and the RNA was extracted with TRIzol methodology.

### Stressing cells with a pathogenic RNA-analogue

The synthetic dsRNA polymer poly(I:C), which induces the RNA sensors that activate innate immunity [39, 40], was purchased from InvivoGen in the low molecular weight variant with the transfection agent LyoVec and used at a concentration of 1 μg/ml (for SH-SY5Y cells) or 2 μg/ml (for MEFs) as recommended by the manufacturer during 16 h before harvesting the cells and extracting RNA/protein.

### Human primary skin fibroblast cultures

Previously established primary skin fibroblast cultures from 3 homozygous PARK6 patients (passages 12–14) were employed as published [12, 19, 41–43], in addition to one sex-/age-matched control (the principal investigator G.A., passage 14) and four matched control fibroblast lines from Coriell depository (catalog number AG02261/passage 6/age 61, AG06103/passage 16/age 29, AG06858/passage 5, age 47, AG12207/passage 14/age 68).

### Quantitative immunoblotting

The isolation of total proteins from the primary skin fibroblasts was carried out as described [42]. Samples of 20 μg were heated at 90 °C for 5 min and then separated in 10% tris–glycine polyacrylamide gels, using Precision Plus Protein™ All Blue Standards as size marker. Transfer to nitrocellulose membranes (Protran, GE Healthcare) was done at 50 V for 90 min, with blocking in 5% BSA solution in 1X TBS-T for 1 h at room temperature (RT). Primary antibodies against LRRK2 (1:1000, NBP1–49954, Novus), IFIT3 (1:500, 15,201–1-AP, Proteintech), IFIT1 (1:500, 23,247–1-AP, Proteintech), DDX58 (1:700, 3743, Cell Signaling Tech), RSAD2 (1:500, 11,833–1-AP, Proteintech), and β-Actin (1:5000, A5441, Sigma-Aldrich) occurred in 1X TBS-T solutions overnight at 4 °C. Fluorescent-labeled α-mouse (1:15,000, IRDye 800CW, Li-Cor) and α-rabbit (1:15,000, IRDye 680RD, Li-Cor) were the secondary antibodies. Fluorescence detection occurred on the Li-Cor Odyssey Classic Instrument.

### Statistical analyses

Statistical significance was assessed using ANOVA or unpaired *t* test with Welch’s correction in the GraphPad Prism 5 software.

## Results

### Global transcriptome profile of *Pink1*^−/−^ mouse brain and its progression during aging

Previously, we documented the effects of PINK1 deficiency on brain regions such as midbrain, striatum, and cerebellum at different ages in the absence of stress or in the presence of A53T-alpha-synuclein overexpression as stressor, identifying marked effects of PINK1-deficiency on mitochondrial biology and excitability via global transcriptome profiles [20–22, 29, 44]. Irrespective of the size of fold-changes, we now document all significant expression dysregulations in *Pink1* ^−/−^ brain, which were consistent (1) across the lifespan and (2) across diverse brain areas (Additional file 1: Table S1). As expected, the loss of *Pink1* transcript constituted the most significant effect, being detected by two microarray probesets. An additional downregulation was observed for the splicing repressor *Srsf10* (also called *Fusip1*) (to 63% on average), a stress response factor which regulates the levels of the inflammasome component Caspase 1 [45]. A downregulation was found also for the vesicle endocytosis factor *Clta* (to 32%), which controls antibody isotype switching [46]. A converse upregulation was documented by two independent probesets for the splicing activator *Srrm1* (to 187%), which is responsible for splicing of the lymphocyte surface protein CD44 [47]. Another upregulation detected by two independent oligonucleotide probesets appeared for the precursor RNA processing factor *Hnrnpr*, which is known to regulate c-Fos and thus neuronal excitability, but also the expression of classical and non-classical MHC class I proteins (probeset *2610528B01Rik* showing transcript elevation to 172% and probeset *Gm17388* to 131%) [48, 49]. Currently, there is almost no experimental evidence implicating PINK1 in splicing [22] or in endocytosis [50]. Against our expectations, these expression dysregulations did not increase with age, so the fold-changes at age 18 months were very similar to those at 6 weeks. Thus, the focus on the expression of individual transcripts failed to identify markers of pathology progression.

### Bioinformatic pathway analysis: Gene Ontology Enrichment by STRING

To further elucidate the expression effects of PINK1-deficiency in nervous tissue, we now considered the enrichment of functional pathways and of protein interaction networks, employing automated bioinformatics tools. This approach was focused on the cerebellum, in view of several advantages: (1) The cerebellum is relatively big and easy to dissect with negligible anatomical variance; (2) its expression changes will not get diluted as in other brain regions where substantial neuron population heterogeneity renders many effects minimal upon mixed tissue analysis; and (3) it does not suffer from the neuron loss and astrogliosis, which are expected to occur in the vulnerable brain regions of PD models and which would distort expression profiles.

To illustrate the impact of PINK1 deficiency on specific gene networks within the global cerebellar transcriptome and to visualize the progression from age 6 weeks via 6 months until age 18 months, we produced a STRING diagram of protein-protein interaction clusters for each age (Additional file 2: Fig. S1A–S1C). In these figures, it is evident that the progression of pathology involves progressively increasing numbers of individual factors and pathways, so that at age 18 months, only the most severe effects could be visualized.

**Fig. 1:**
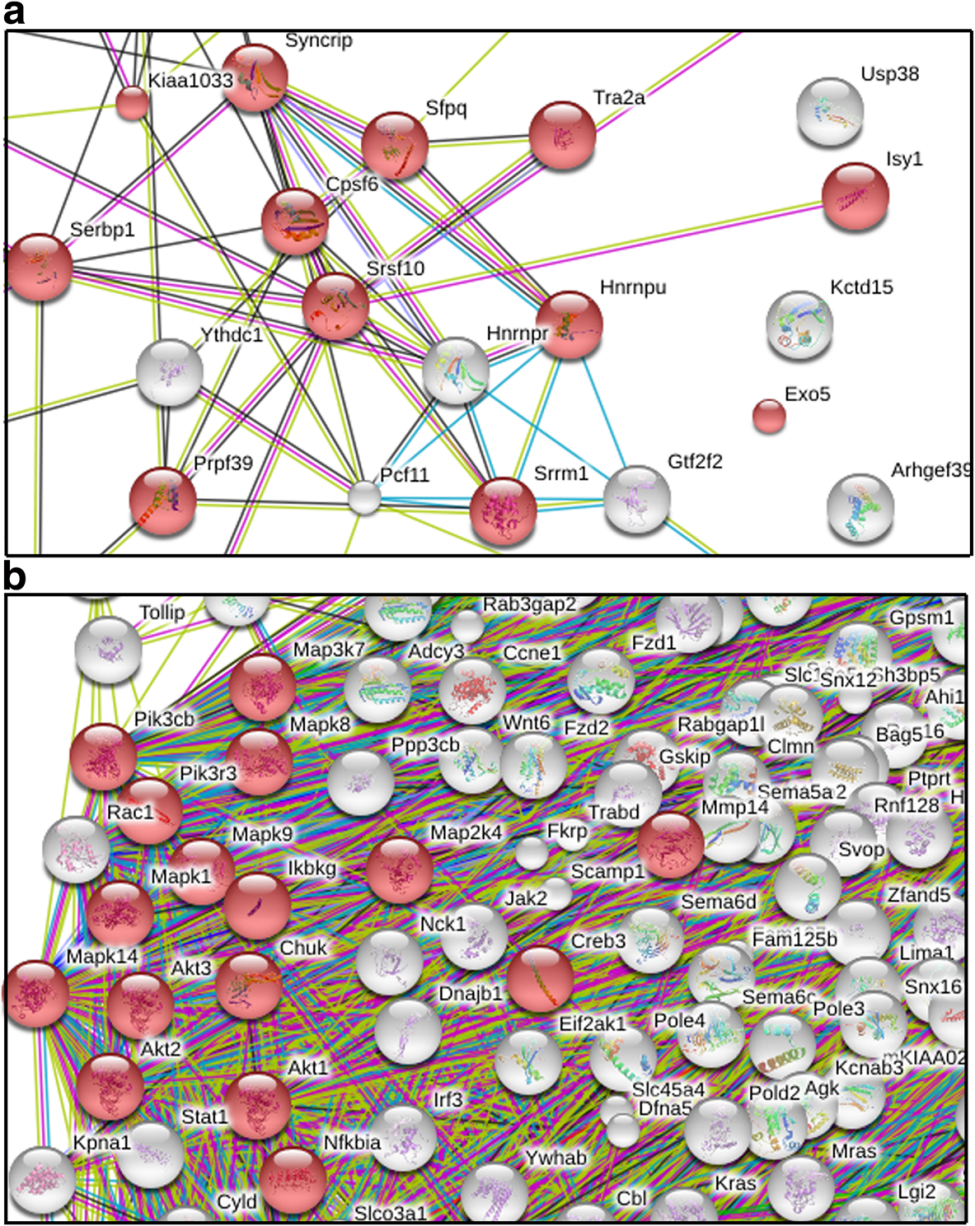
Details from the STRING interaction diagrams within the global transcriptome profiles of *Pink1*-deficient mouse cerebellar tissue. **a** At age 6 weeks, a network of splicing factors around *Srsf10* was dysregulated. *Red symbols* indicate membrane-associated factors. The complete profile interaction diagram can be found in Additional file 2: Fig. S1A. **b** At age 18 months, a much denser network including inflammatory factors like *Creb3*, *Irf3*, *Nfkbia*, *Mapk8*, *Mapk9*, and *Stat1* was dysregulated. Components of the “Toll-like receptor signaling pathway” factors are highlighted in *red color*. The complete profile interaction diagram can be found in Additional file 2: Fig. S1C

In mice at age 6 weeks, around 250 factors showed significant expression changes (with false discovery rate, FDR, adjusted *p* value below 0.05) in Affymetrix oligonucleotide microarray studies (Additional file 3: Table S2A, first datasheet). Their analysis regarding the enrichment of specific pathways and protein-protein interactions with the automated bioinformatics tools at the STRING web-server detected significant dysregulation for 158 molecules within the Gene Ontology (GO) Cellular Component term “intracellular membrane-bounded organelle” (FDR *q* value = 6.39e−08) (Additional file 3: Table S2B), in good consistency with the known mitochondrial localization of PINK1 protein during stress. Interestingly, an enrichment was notable for the KEGG pathway “Spliceosome” (*q* value = 0.04 only, in view of the few dysregulations at this initial stage of pathology) (a blown-up interaction diagram detail around the splicing factor *Sfrs10* is shown in Fig. 1a). The STRING diagram (Fig. 1a, Additional file 2: Fig. S1A), which represents this age, shows the intracellular membrane-bounded organelle factors highlighted in red color.

In mice at age 6 months, over 1300 factors showed expression changes at this significance threshold (Additional file 3: Table S2A, second datasheet). Their bioinformatics analysis detected significant dysregulation for 826 molecules within the GO Cellular Component term "intracellular membrane-bounded organelle" (*q* value = 7.61e−16) (Additional file 3: Table S2C). Significant enrichment in the KEGG pathways “Ubiquitin-mediated proteolysis” (*q* = 1.01e−05) and “Protein processing in endoplasmic reticulum” (*q* = 3.34e−04) were notable. The corresponding STRING protein interaction diagram (Additional file 2: Fig. S1B) for this age again shows the intracellular membrane-bounded organelle factors highlighted in red color.

In mice at age 18 months, about 3500 factors showed expression changes at this significance threshold, too many to be processed by STRING bioinformatics (its limit is at 2000 nodes). Thus, only the about 1600 dysregulated factors with an adjusted *p* value below 0.01 were analyzed regarding interactions and enrichments (Additional file 3: Table S2A, third datasheet). Significant enrichment for 1031 molecules within the GO Cellular Component term intracellular membrane-bounded organelle (*q* value 6.34e−67) was detected (Additional file 3: Table S2D). At this stage of progression, significant enrichment was observed in several GO biological processes (Additional file 3: Table S2E), particularly 537 factors in “cellular response to stimulus” (*q* value 2.82e−14), 165 factors in “cellular response to stress” (*q* value 1.02e−09), and 23 factors in “regulation of mitophagy” (*q* value 0.038). These data are in agreement with the notion that the well-documented role of PINK1 in mitophagy is a small part of a much broader role of PINK1 in stimulus-dependent signaling and in stress responses. Significant enrichment was also observed in multiple KEGG pathways (Additional file 3: Table S2F), prominently for 47 factors in the “MAPK signaling pathway” (*q* value 2.64e−06), for 32 factors in the "ubiquitin-mediated proteolysis pathway" (*q* value 2.64e−06), for 34 factors in the "Protein processing in endoplasmic reticulum" pathway (*q* value 1.68e−05), and for 20 factors in the “Bacterial invasion of epithelial cells” pathway (*q* value 6.37e−05). Mildly significant was the enrichment of 21 factors of the “Dopaminergic synapse” pathway (*q* value 0.005), 16 factors of the “GABAergic synapse” pathway (*q* value 0.008), and 18 factors of the “Glutamatergic pathway” pathway (*q* value 0.015). These data are in excellent consistency with previously established roles of PINK1 as a component of MAPK signaling [51, 52], as an activator of PARKIN which prevents bacterial invasions [18], and as a modifier of dopaminergic, GABAergic, and glutamatergic signaling in the nigrostriatal pathway [44, 53–56]. The agreement of our bioinformatics analysis of the global transcriptome with previously published cell biology data provides credibility to this automated approach.

In the STRING interaction diagram of cerebellar transcriptome changes at age 18 months, the appearance of neuroinflammatory factors was highlighted, manually placing the components of the significantly enriched KEGG pathways “HTLV-I infection”, “Toll-like receptor signaling”, “TNF signaling pathway”, “Fc epsilon RI signaling”, “T cell receptor signaling pathway”, “RIG-1-like receptor signaling”, “Hepatitis C”, and “Influenza A” in the lower left corner and highlighting the Toll-like receptor signaling pathway factors in red color (Additional file 2: Fig. S1C). A detail of this interaction diagram with focus on the inflammatory factors *Creb3, Irf3, Nfkbia, Mapk8, Mapk9,* and *Stat1* is shown in Fig. 1b.

Heat maps were then used to focus on prominent examples among the significantly altered pathways, representing each dysregulated component at the three ages, not only for the Parkinson-resistant cerebellar tissue but also for comparing the Parkinson-vulnerable midbrain/brainstem and striatum (Table 1). This approach visualizes the temporal and spatial appearance of pathology as well as the effect sizes in a color code, with deep red mirroring strong upregulations and deep blue shades for strong downregulations. It also illustrates the number of factors and possibly the relevance of each pathway. Although the effect of PINK1 on ubiquitin-mediated proteolysis and on mitophagy are excellently studied, providing proof of principle that these moderate changes of many pathway components are relevant, it was novel to detect a selective dysregulation in particular for *Ube3a* that has a known role in the degradation of cytoplasmic misfolded proteins like alpha-synuclein [57–59], for *Xiap* with an established function in ubiquitination, mitochondrial apoptosis, and innate immunity [60], for *Dnm1l* (encoding DRP1) with previously defined effects on PINK1-dependent mitochondrial fission [7, 61], as well as for *Slc6a1* as a GABA transporter known to be induced by neuroinflammation [62]. The regional comparison in Table 1 made it evident that PINK1-deficiency-mediated downregulations of stress-response factors dominate in the PD-resistant cerebellar tissue, while the same factors show converse progressive upregulation in the PD-affected midbrain/brainstem tissue, e.g., *Keap1*, *Mbtps1*, *Rad23a*, *Sec61a1*, *Hif1a*, *Pi4k2a*, the autophagy factors *Map1lc3b* and *Sqstm1*. Further opposing regulations occurred for the ubiquitin-binding factor *Tollip*, which regulates Toll-like receptor signaling as well as the autophagic clearance of ubiquitin conjugates and protein aggregates [63]. This supersensitive upregulation in tissues, which are known to suffer PD-specific stress, might represent a cellular effort to compensate PINK1 deficiency further downstream.

**Table 1:**
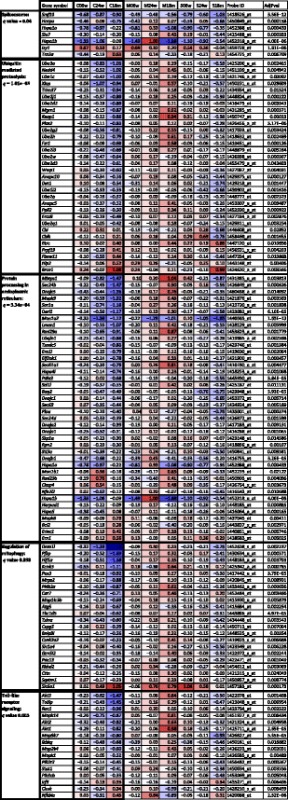
Global transcriptome analysis with prominent pathway dysregulations upon STRING assessment at 3 ages in different brain regions of *Pink1* ^−/−^ mice, illustrated as heat maps

Expression dysregulations in Affymetrix microarrays are shown in different brain regions (C = cerebellum, M = midbrain, S = striatum) across the mouse lifespan (6 W = 6 weeks, 24 W = 24 weeks = 6 months, 18 M = 18 months), for genes detected by a specific oligonucleotide probe ID, together with the Adjusted *p* values (AdjPval) as determined by Affymetrix transcriptome bioinformatics via software platform R and the bioconductor package Limma. Displayed are the genes contained in five exemplary pathways, which exhibited significant enrichment according to STRING bioinformatics, as indicated by the false discovery rate *q* values. The ranked order of all dysregulated pathways upon STRING assessment can be found in Additional file 3: Table S2

If the STRING analysis is conducted with high stringency only for those cerebellar factors, which are significantly downregulated with >1.5-fold change, then much information is lost and the KEGG pathway of MAPK signaling becomes prominent at the different ages (Additional file 4: Table S3). This marked effect is not unexpected, since PINK1 (short for PTEN induced kinase 1) is downstream of PTEN, a phosphatase that antagonizes the kinase PI3K, which is an upstream modulator of the MAPK phosphorylation cascade at various levels. Furthermore, the interaction of PINK1 with the MAPK pathway has already been demonstrated experimentally in neuroblastoma cells, astrocytes, HeLa, and HepG2 liver cells [51, 64–66].

### Bioinformatic pathway assessment by Gene Set Enrichment analysis

Going even further, instead of considering only the expression dysregulations beyond a significance threshold, we employed a tool that takes all genes with their expression ratios into account to compare them with known gene sets of functional relevance. This approach even recognizes compound effects, when most components of a pathway are subtly dysregulated in the same direction. This Gene Set Enrichment Analysis (GSEA) software thus performs a comprehensive assessment of the complete transcriptome data and is useful as an independent complement of the STRING approach. In the cerebellum, the most salient result was a significant effect for the KEGG pathway “Parkinson’s disease”, but this biomathematical finding was mainly due to the loss of *Pink1* transcript. Again, at the age of 6 weeks among the KEGG pathways, spliceosome downregulation appeared prominently (Additional file 5: Table S4). Downregulations of “Antigen processing and presentation” as well as "Ubiquitin-mediated proteolysis" were already significant. Among the Reactome pathways at this age, downregulations in the “innate immune system” already had nominal significance. At the age of 6 months, again an impairment of "endoplasmic reticulum stress responses" appeared with a significant downregulation of the Reactome pathway “Activation of chaperone genes by Xbp1s”. At the age of 18 months, among the Reactome pathways, “antiviral mechanism by IFN stimulated genes” became prominent, and in particular the “Negative regulators of RIG I and MDA5 signaling” just touched significance in FDR. For the maximal pathology at the age of 18 months in the cerebellum, the top dozen dysregulated pathways from the KEGG database and the top dozen dysregulated pathways from the Reactome database were documented with individual components and their expression scores (Additional file 6: Table S5). Moreover, four Reactome pathways with special relevance for this manuscript were analyzed in their progressive dysregulation by heat maps (Additional file 7: Table S6). Thus, the temporal order of pathway involvement and the late-stage prominence of neuroinflammation were reproducible in an alternative approach.

### qPCR validation of candidate dysregulations in aged *Pink1*^−/−^ mouse cerebellum

Experimental validation was performed for several dysregulated components of these pathways. As shown in Additional file 8: Fig. S2A, a significant downregulation was confirmed for the stress responsive splicing factor *Srsf10* [67], while significant upregulations were observed for the cerebellar mRNA levels of *Creb3* (human LZIP, synonymous LUMAN), which influences endoplasmic reticulum protein processing [68, 69], and for *Nfkbia* (NF-Kappa-B inhibitor alpha), which cooperates with *Creb3* to modulate the nuclear control of stress and inflammation responses [70–73], but not for other candidates within the *Mapk* (MAP kinase) gene family (Additional file 8: Fig. S2A). Of particular interest was the dysregulation of *Ube3a* within the ubiquitination pathway, because only one splice isoform changed its expression, which might be target of the spliceosome adaptation observed above, and because UBE3A is involved in the degradation of alpha-synuclein as the main driver of Parkinson pathogenesis [59].

We then focused on JNK1 (*Mapk8* mRNA) expression, a crucial component of the stress-dependent phosphorylation cascades that control endoplasmic reticulum protein processing, neuroinflammation, and apoptotic or autophagic cell death [74–77]. JNK1 activity is also regulated by alternative splicing [78, 79]; therefore, three representative exon-exon junctions were assessed. Quantitative reverse transcriptase PCR (qPCR) in *Pink1* ^−/−^ cerebellum at age 18 months in the absence of additional stressors demonstrated a significant but mild upregulation, 1.15-fold ±0.04 SEM with *p* = 0.01 (Additional file 8: Fig. S2B). This finding is consistent with a previous report of increased p38 MAPK activation in PINK1-deficient mouse astrocytes [51]. However, the expression change of JNK1 was too subtle to be validated by quantitative immunoblots. This technique has inherent variability and limited linearity, so it requires an impractically high number of samples to detect 1.1-fold changes. Thus, a subtle dysregulation of the spliceosome pathway, the endoplasmic reticulum protein processing pathway, and neuroinflammatory response pathways was observable in global microarray transcriptome profiles upon unbiased bioinformatics analyses in old *Pink1* ^−/−^ mouse cerebellum, and the alteration of these pathways could be validated experimentally by qPCR for several key factors.

Overall, PINK1 deficiency triggered a dysregulated expression of many membrane-associated factors already in the first weeks of life. The progression of pathology occurred mainly through involvement of more pathways and protein interaction networks, rather than through stronger expression dysregulation. Pathological mitophagy and neuroinflammation profiles were apparent by the age of 18 months in *Pink1* ^−/−^ cerebellum.

### Activation of the cellular immune response in *Pink1*^−/−^ mouse brain

**Fig. 2:**
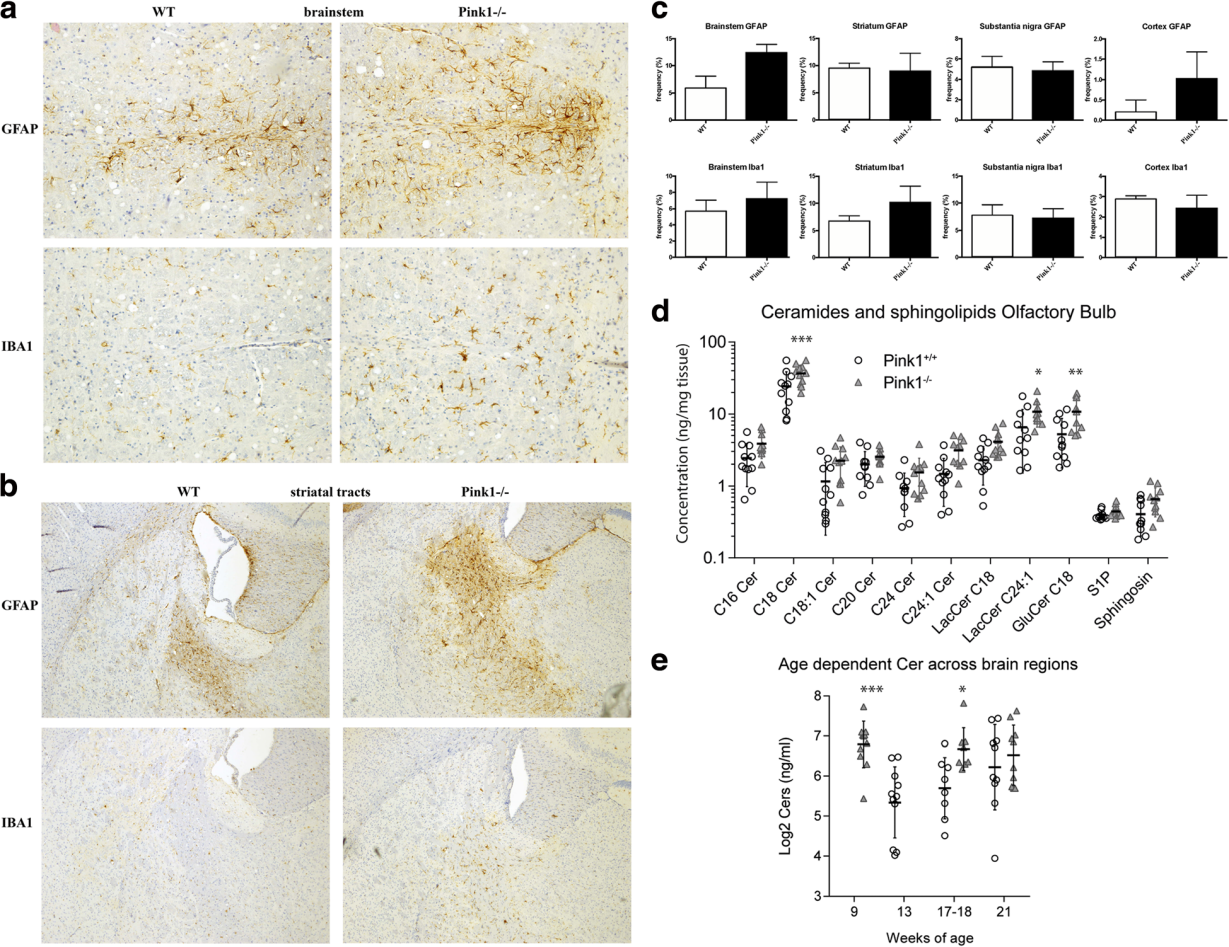
Astroglial and microglial response, as well as ceramide accumulation in *Pink1*-deficient mouse brain. The immunohistochemical staining of GFAP as astroglial marker and Iba1 as microglial marker in *Pink1*−**/−** mouse brain at the age of 18 months demonstrated a mild increase in the frequency of inflammatory glial reactions, best detectable along the corticospinal tract where it traverses the brainstem (**a**, 10× objective used) and the striatal region (**b**, 4× objective). **c** The elevated frequency of this glial response was demonstrated in cell counts for astrogliosis and microgliosis in relation to total cells per area (nuclear counterstain) in 18-month-old brains that underwent automated immunohistochemistry of GFAP1 and Iba1 demonstrated region-specific mild effects. The substantia nigra was less affected than areas that are penetrated by myelinated axons. Data are displayed as *bar graphs*, illustrating mean values of serial sectioned and randomly picked mouse brain areas. Brain graphs show standard error of the mean (SEM) of technical replicates with at least *n* = 3 sections per genotype per region. Statistical analysis would be inappropriate, given that the serial sections were from too few inbred animals. **d** Increased levels of ceramides, glucosyl-ceramides, and lactosyl-ceramides were observed by liquid chromatography tandem mass spectrometry in the olfactory bulb summarized for ages of 9–13 months, 17 months, and 21 months (*n* = 3 or 4 mice of each age and genotype). The scatter shows individual mice, the line is the mean, and whiskers show the standard deviation. **e** Log2-transformed total ceramides across brain regions reveal age-dependent differences between genotype, i.e., an increase over time in wildtype mice that contrast with very high levels in younger *Pink1* ^−/−^ mice which remain at this elevated level. Two-way ANOVA differed significantly between genotypes. *Asterisks* illustrate the significance (multivariate ANOVA, subsequent *t* test for each lipid, Holm-Šidák adjusted *p* values, *p < 0.05, **p < 0.01, ***p < 0.001)

To test if the molecular profile of immune activation is reflected by cellular responses in the brain tissue, immunohistochemistry of GFAP as marker of astroglia and of Iba1 as marker of microglia was performed. Parallel processing of the sections was necessary to visualize their subtly increased immunoreactivity in diverse areas of the *Pink1* ^−/−^ brain, which was prominent at the myelinated fiber tracts, e.g., the corticospinal projections through the striatum, the brainstem, and also in the cerebellar white matter (Fig. 2a, b). This increase was not detectable in nuclei like the striatal matrix or the substantia nigra and was not consistent in the cerebral cortex upon counting reactive cells in sections at even intervals throughout the brain (Fig. 2c). Thus, the dysregulation of membrane-associated factors in old animals leads to a cellular immune response most likely at myelinated axons.

### Accumulation of ceramides in *Pink1*^−/−^ mouse brain

To further assess the relevance of the subtle transcriptional changes at the effector molecule level, we decided to analyze ceramides of different chain lengths and saturation and their sugar-modified metabolites because ceramides trigger lethal mitophagy [80], lactosyl-ceramides maintain neuroinflammation [81], and ceramides accumulate upon lysosomal dysfunctions and LRRK2-deficiency [82] and are expelled by activated astrocytes via exosomes [83]. Hence, ceramide pathology may be a key factor for the progression of Parkinson’s disease.

Ceramides, glucosyl-ceramides, and lactosyl-ceramides were increased in the *Pink1* ^*−/−*^ olfactory bulb starting from 9 months on (Fig. 2d). Analysis of variance for repeated measurements revealed significant differences between genotypes (*F* (1:20) = 7297; *P* = 0.0137) and for the interaction “ceramide by genotype” (*F* (10:200) = 4.558; *P* < 0.0001). Subsequent post-hoc analyses for each ceramide and sphingolipid separately were significant for Cer18:0 (*P* < 0.0001), LacCer24:1 (*P* = 0.0285), and GluCer18:0 (*P* = 0.0042). For individual ceramides, there was no site-specific significant progression with increasing age, but analysis of log-2 transformed total ceramides revealed age-dependent differences. In wildtype animals, log-2 Cers linearly increased over time, but in Pink1^−/−^, levels were already increased at 9 months and remained at this elevated level, so that genotype-dependent differences were strongest in young mice (2-way ANOVA “genotype x age” for genotype *F* (1, 54) = 16.98, *P* = 0.0001; for the interaction *F* (2, 54) = 2.531, *P* = 0.089) (Fig. 2e). Hence, the observed increase of ceramide levels is strongly suggestive of ongoing neuro-inflammation in the brain starting in young adulthood, particularly in the olfactory bulb—a brain area which is among the first to be affected by Parkinson’s disease.

### Global transcriptome profile of *Pink1*^−/−^ mouse neuron-rich primary cultures

Given that PINK1 is important for stress responses [5, 6, 13], and in view of the enhanced release of inflammatory cytokines from acutely prepared *Pink1* ^−/−^ brain slices [84], we decided to study very young *Pink1* ^−/−^ neuron-rich primary culture in vitro—where the previous mechanical dissection, the lack of glial support, and the presence of the toxin cytosine arabinoside (cytarabine) exert combined stress [85, 86]. Hence, murine neuron-rich primary cultures were established at postnatal age and maintained for 12 days to assess early effects of PINK1 deficiency on global transcriptome readouts (Additional file 9: Table S7). Given that genomic insertion events may influence the expression of neighboring genes across a distance of 3 MegaBases [87], it was reassuring that only one dysregulated transcript, *Nipal3*, was encoded in the vicinity of *Pink1*.

Two findings had obvious credibility, but limited novelty, namely the dysregulation of *Dram1* and *Ret* mRNA levels. Ordered by significance, the *Dram1* (the abbreviation stands for DNA-damage regulated autophagy modulator 1) transcript 1.7-fold upregulation was the strongest early change after *Pink1* loss (*p* value ≈ 0.0001). Similar to PINK1, DRAM1 is connected to the pathway of selective autophagy, since it promotes the targeting of mycobacteria to degradation [88], and is thus contributing to innate immunity responses. *DRAM1* mRNA is selectively downregulated in brain tissue of PD patients [89]. A similarly meaningful observation was the 1.7-fold upregulation of *Ret* (proto-oncogene RET receptor tyrosine kinase) (*p* value ≈ 0.001). RET trophic receptor signaling was already shown to rescue histological and biochemical phenotypes of *Pink1* deletion in flies [15]. In a human neuroblastoma cell line, the administration of its receptor ligand GDNF compensates morphological and bioenergetic deficits of *PINK1*-deficient cells without affecting mitophagy. Furthermore, GDNF stimulation rescued mitochondrial defects in PARKIN-deficient cells, while the loss of dopaminergic midbrain neurons in aged RET-deficient animals was rescued by PARKIN overexpression and exacerbated by PARKIN deficiency [16]. Intriguingly, GDNF transfected macrophages significantly ameliorated neuroinflammation and neurodegeneration in a PD mouse model [90]. Thus, the *Ret* mRNA upregulation in *Pink1*-deficient primary neuron cultures after dissociation stress may also form part of the neuroinflammatory response pathway.

In addition to the known candidates, we observed a remarkable and novel enrichment of additional factors involved in antiviral state, innate immunity, and neuroinflammation (Additional file 9: Table S7) including *Gin1* (gypsy retrotransposon integrase 1) [91], *Hmox1* (Heme oxygenase 1) [92, 93], *Hebp1* (Heme-binding protein 1) [94], *Snx16* (Sorting nexin 16) [95], *Timp1* (Tissue inhibitor of metalloproteinase 1) [96, 97], *Dnmt2* (DNA methyltransferase 2) [98–101], *Rsad2* (Radical S-adenosyl methionine domain containing 2 = Viperin) [102–106], *Adamts4* (a disintegrin-like and metallopeptidase—reprolysin type—with thrombospondin type 1 motif, 4) [107–109], *Csde1* (Cold shock domain containing E1, RNA binding = UNR) [110], and *Ifit3* (interferon-induced protein with tetratricopeptide repeats 3) [111, 112].

Further enrichments in the transcriptome profile were apparent for DNA/RNA-quality control and for antioxidant/hypoxia factors. The DNA/RNA quality control factors included *Zfp148*, *Cdk7*, *Tia1*, *Mbd1*, *Mus81*, *Mat2a*, and *Hipk2* [113–127]. The hypoxia/antioxidant factors, which are credible in view of the well-documented oxidative stress and bioenergetics deficit in PINK1-deficient cells [21, 41], included *Srxn1*, *Hlf*, *Steap1*, *Tsga10*, and *Hipk2* [126–132].

Intriguingly, *Foxp1* (Forkhead Box P1) showed a 1.7-fold downregulation (*p* value ≈ 0.03). This differentiation factor of midbrain dopaminergic neurons was previously found downregulated also in mice with knock-out of alpha-synuclein, while its upregulation was documented in mice with transgenic overexpression of A53T-alpha-synuclein, which model the PARK1 and PARK4 variants of PD [30, 31, 133]. Thus, the dysregulation of *Foxp1* transcripts levels appears to respond both to acute stress and to constant mutations of at least two PD genes.

Overall, already at neonatal stages, a significant expression dysregulation of innate immunity factors in *Pink1* ^−/−^ primary neurons became apparent under in vitro stress conditions.

### Transcript analysis in the brain from adult *Pink1*^−/−^ mice with chronic A53T-SNCA stress

For independent validation of these observations, and to exclude that a microbial infection of the cultures had produced artifacts, we next studied the transcript levels of candidate genes by qPCR in brain homogenate extracts from independent animals. Given that the stress-evoked observations in primary *Pink1* ^−/−^ neurons were not evident in global transcriptome screenings of single-mutant *Pink1* ^−/−^ mouse brains even at old age [20], we reasoned that additional challenges may be necessary to manifest PINK1-dependent stress response dysregulations. Thus, we studied double-mutant mice, where the *Pink1* ^−/−^ is combined with 1.5-fold overexpression of A53T-alpha-synuclein (SNCA) selectively in neurons as a trigger of Parkinsonian pathology and as a genetic interactor of PINK1 [19, 20]. These double-mutant mice show a potentiated phenotype with appearance of Lewy-like inclusion bodies and with lethality from the age of 14 months onward [20]. The global transcriptome in several brain regions throughout their lifespan was previously compared to single mutant *Pink1* ^−/−^ mice, and the data are publically available in a database [20]. It has always been our experience that the heterogeneity of neuron populations in midbrain/brainstem and the dissection variance make it very difficult to demonstrate subtle expression changes.

In midbrain from adult double-mutant *Pink1* ^−/−^+A53T-SNCA mice at the age of 18 months, a significant 1.16-fold upregulation for *Mapk9* (*p* value = 0.0007), a significant 2.15-fold upregulation for *Rsad2* (*p* value = 0.0007), a trend towards significance (*p* value = 0.08) for the 0.87-fold downregulation (a dysregulation so subtle that it would usually be deemed irrelevant) of *Hebp1*, and a significant 1.92-fold upregulation for *Tnf* alpha were observed (*p* value = 0.03) (see Additional file 10: Fig. S3).

Thus, also in midbrain, the PINK1 deficiency led to dysregulated expression levels of key factors in the anti-microbial defense, when an additional challenge represented by mutant A53T-alpha-synuclein exceeded the allostatic threshold.

### Systematic study of antiviral factor mRNAs in human *PINK1*-knockdown neuroblastoma cells after acute starvation

**Fig. 3:**
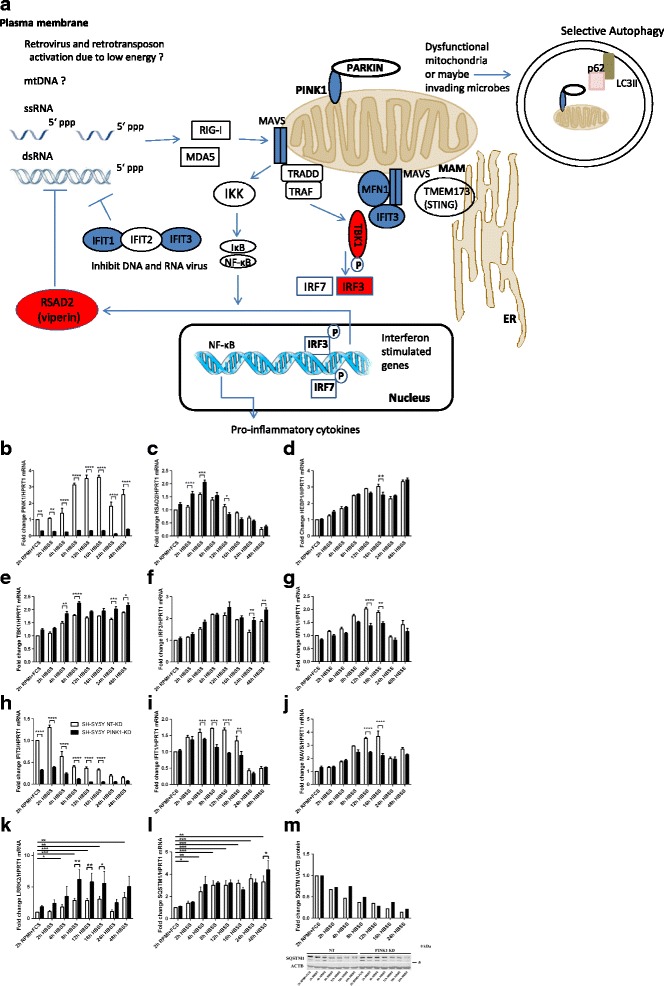
Systematic analysis of key factors in the mitochondria-associated innate immunity pathway, using acute starvation of human SH-SY5Y neuroblastoma cells to reveal the transcriptional regulation of stress responses and their dependence on PINK1. Transcript levels were documented in control non-target knock-down (NT-KD) versus *PINK1*-knock-down (*PINK1*-KD) cells for the following inflammatory factors. **a** Summary scheme on the detection of pathogen DNA/RNA in the cytosol and the mitochondria-associated triggering of innate immunity, as altered during starvation by PINK1. Viral RNA (ssRNA or dsRNA) is recognized in the cytosol by helicase RIG-I and MDA5. This triggers the dimerization of MAVS in the outer mitochondrial membrane (OMM) which recruits transactivators (such as TRADD, TRAF, IKK family) leading to nuclear translocation of phosphorylated IRF3, IRF7, and NF-κB and promoting the transcription of IFN stimulated genes and pro-inflammatory cytokines, respectively. Effector molecules such as IFIT1, IFIT2, IFIT3, and RSAD2 (viperin) inhibit virus DNA/RNA replication. IFIT3 also functions as a scaffold to facilitate interaction between MAVS and TBK1 and represents a positive feedback of DDX58 (RIG-I) signaling through MAVS. Low ΔΨm or decreased ROS inhibit MAVS-mediated signaling. Mitochondria cooperate with the endoplasmic reticulum (ER) to regulate lipid synthesis and antiviral signaling at the mitochondria-associated membranes (MAM), possibly by interactions of MAVS/MFN1 with TMEM173 (STING). MFN1 leads to the redistribution of MAVS along mitochondria and a fusion of the mitochondrial network that promotes the interaction between MAVS and STING. Low energy induces the localization of PINK1 to the OMM and recruitment of PARKIN from the cytosol, which is the signal for dysfunctional mitochondria to be digested in the autophagosome. Abbreviations: *5′ ppp* 5′ triphosphate, *ΔΨm* mitochondrial membrane potential, *IκB* inhibitor of κ light polypeptide gene enhancer in B cells, *IKK* IκB Kinase, *LC3II* phosphatidylethanolamine conjugate of the autophagy-related protein LC3 (MAP1LC3), *MDA5* melanoma differentiation-associated gene 5, *NF-κB* nuclear factor κB, *p62* sequestosome-1, adaptor between polyubiquitinated substrates and autophagic machinery, *PARKIN* ubiquitin ligase, its loss-of-function leads to the PARK2 variant of Parkinson’s disease, *RIG-1 (DDX58)* retinoic acid-inducible gene-1 (DExD/H-Box RNA Helicase 58), *ROS* reactive oxygen species, *ssRNA/dsRNA* single-stranded/double-stranded RNA, *TMEM173 (STING)* transmembrane Protein 173, *TRADD* tumor necrosis factor receptor type 1 associated death domain protein, *TRAF* TNF-receptor-associated factor*.* **b** *PINK1* (PTEN induced kinase 1) as a known determinant of selective mitophagy and autosomal recessive Parkinson’s disease; *HPRT1* (Hypoxanthine Phosphoribosyltransferase 1) as a loading control. **c** *RSAD2* (= viperin, radical S-adenosyl methionine domain containing 2) as an interferon-inducible lipid-droplet associated virus inhibitory factor. **d** *HEBP1* (heme binding protein 1) that contains a natural ligand for formyl peptide receptor-like receptor 2. **e** *TBK1* (TANK-binding kinase 1) that phosphorylates interferon regulatory factors in response to toll-like receptor activation. **f** *IRF3* (interferon regulatory factor 3) as a regulator of type I Interferon gene transcription. **g** *MFN1* (mitofusin 1) as PARKIN-dependent factor in mitochondrial dynamics and mitochondria-associated anti-microbial signaling. **h** *IFIT3* (interferon-induced protein with tetratricopeptide repeats 3) as a detector of pathogen DNA/RNA. **i** *IFIT1* (interferon-induced protein with tetratricopeptide repeats 3) as a detector of pathogen DNA/RNA. **j** *MAVS* (mitochondria-associated viral sensor) as inducer of interferon-dependent long-term expression of defense factors. **k**–**m** The levels of known autophagy factors during this starvation time course exhibited progressive consumption of p62 in spite of its transcriptional induction, in parallel to a *PINK1*-modulated transcriptional induction of *LRRK2.* Four independent experiments compared their expression during a nutrient and trophic deprivation time course triggered by a culture switch from RPMI growth medium to HBSS starvation medium. The bar graphs show mean and standard error of the mean, illustrating the significance with asterisks (*p < 0.05, **p < 0.01, ***p < 0.001, ****p < 0.0001)

We decided to test in vitro whether (I) similar observations indeed occur in neural cells or have to be attributed to microglia, (II) the mouse data can be translated to human, (III) transient changes can be defined after acute stress, and (IV) trophic and nutrient deprivation stress with concomitant autophagy can trigger these stress response changes, always in dependence on PINK1 deficiency. The starvation stress was initially chosen rather than established Parkinsonian toxins like MPTP or CCCP [6, 134], since PINK1 was recently shown to respond to mutations in ATXN2, which is a known starvation response factor [135–138], and in view of the known transcriptional induction of PINK1 and PARKIN during trophic deprivation [13]. The human neuroblastoma cell line SH-SY5Y with stable lentiviral knockdown (KD) of *PINK1* versus a non-target (NT) control sequence were subjected to acute starvation by changing the culture conditions from RPMI medium with 10% Fetal Calf Serum (FCS) to HBSS medium (low glucose, no amino acids) without FCS. The transcriptional regulation was documented over 2 days for several key factors of the antiviral defense by qPCR. The overview on this systematic survey of the mitochondria-associated antiviral defense pathway is represented in Fig. 3a, with blue color illustrating deficiency and PINK1-dependent downregulation, while red color illustrates PINK1-dependent upregulation. The KD was stable, since *PINK1* mRNA in this cell line was reduced to 30% before stress and to 9% after stress at the 16-h time point (Fig. 3b). As described, the starvation induced *PINK1* mRNA in a phasic manner with a peak at 12 h [13]. Similar to the previous findings in *Pink1* ^−/−^ mice, an upregulation of *RSAD2* and a downregulation of *HEBP1* were observed in the starving *PINK1*-deficient neuroblastoma cells. *RSAD2* was induced up to 1.6-fold in control cells, and this response was potentiated by 46% in *PINK1*-KD cells, with strong significance at 2 and 4 h (Fig. 3c). The up to 3.3-fold induction of *HEBP1* levels in control cells was significantly diminished at 16 h in *PINK1*-KD cells (Fig. 3d).

Systematic further assessment of the innate immunity pathway revealed further regulations, with PINK1-deficiency modulating the alterations caused by starvation. A supersensitive transcriptional upregulation was observed not only for *RSAD2* but also for *TBK1* and *IRF3*. *TBK1* was induced up to 1.9-fold in control cells, and this induction was further increased to 2.2-fold in *PINK1*-KD cells, with strong significance at 4, 8, 24, and 48 h (Fig. 3e). Similarly, *IRF3* levels were elevated up to 2.2-fold in control cells, and this elevation was exacerbated to 2.4-fold in *PINK1*-KD cells with strong significance at 24 and 48 h (Fig. 3f).

In contrast, a downregulation was observed for a known posttranslational modification target of PINK1/PARKIN signaling, mitochondrial MFN1. The 2.0-fold starvation-triggered induction of *MFN1* transcript was blunted in *PINK1*-KD cells by 32%, with strong significance at 12 and 16 h (Fig. 3g). Starvation triggered an initial short upregulation and later consistent downregulation of *IFIT3* levels to 15% in control cells. In *PINK1*-KD cells, a strong downregulation of *IFIT3* levels appeared already at 2 h and was stably diminished further to 6% of control at 48 h, with significant differences between NT and *PINK1*-KD cells from 2 to 16 h (Fig. 3h). The starvation-triggered up to 1.7-fold induction of *IFIT1* levels was also diminished in *PINK1*-KD cells by 41% with strong significance at 8, 12, and 16 h (Fig. 3i). Finally, the starvation-triggered almost 3.7-fold induction of the mitochondria-associated innate immunity factor *MAVS* was blunted in *PINK1*-KD cells by 40%, with strong significance at 12 and 16 h (Fig. 3j).

No significant dysregulation was documented for the transcript levels of *TMEM173* (STING) as an endoplasmic reticulum-associated antiviral factor (data not shown). Given that experimental testing of multiple mRNAs at multiple time-points followed by statistical evaluation via Student’s *t* tests will exaggerate the significance, an adjustment of the significance threshold according to Bonferroni is useful, by dividing the nominal *p* values indicated in each panel of Fig. 3 through the number of analyses done. After this correction, the downregulation of *PINK1*, *MFN1*, *MAVS*, *IFIT1*, and *IFIT3* and the upregulation of *RSAD2* and *TBK1* remain significant as particularly robust findings.

In order to provide a time-correlation and comparison with autophagy factors that are also regulated by starvation, the curves for *LRRK2* and *SQSTM1* are shown as well (Fig. 3k–m), given that LRRK2 is a positive regulator of inflammation and autophagy, with mutations that trigger autosomal dominant Parkinson’s disease; *SQSTM1* (sequestosome 1 = p62) was studied as an adaptor between the autophagy machinery and ubiquitinated cargo, whose mutation cause neurodegenerative disorders such as motor neuron disease. A significant indirect correlation was observed again between the PINK1-deficiency and an exacerbated induction of *LRRK2* transcript levels, as previously reported for fibroblasts and neuronal cells derived from PARK6 patients [139].

Overall, the data confirm that key factors of the anti-microbial response are modulated in their transcriptional regulation by PINK1 in human neural cells after acute starvation stress.

### Systematic study of antiviral factor mRNAs in human *PINK1*-knockdown neuroblastoma cells after triggering mitophagy by FCCP

To further assess if indeed mitochondrial dysfunction underlies changes in innate immunity, we used the uncoupling drug FCCP to trigger mitophagy and then study the transcriptional response of the above key factors in *PINK1*-knockdown and control SH-SY5Y neuroblastoma cells. Mitophagy needs at least 12 h to occur and subsequent transcriptional responses require additional time. Therefore, the RNA extraction and qPCR analyses were performed later than in the previous experiment, at 24 h after drug administration. As shown in Additional file 11: Fig. S4 above, FCCP in SH-SY5Y cells elicited a transcriptional induction of the mitochondria associated factors *PINK1*, *DDX58*, and *MAVS* and of *TBK1*, accompanied by a downregulation of the RNA sensor *IFIT1*, in control cells. A significant difference between control cells and *PINK1* knockdown cells after FCCP treatment was only observed for *MAVS*, which failed to be induced in the absence of PINK1. These data confirm that key inflammatory factors respond to mitochondrial dysfunction and that particularly the levels of the mitochondrial antiviral signaling factor MAVS depend on PINK1 levels.

### Systematic study of antiviral factor mRNAs in *Pink1*^−/−^ murine embryonal fibroblasts after triggering mitophagy by FCCP treatment

*Pink1* ^−/−^ murine embryonal fibroblasts (MEFs) are a useful tool to test if the presence of lentiviral knock-down double-strand RNA in the human neuroblastoma cells distorts the results and to test whether these effects apply only to neuroinflammatory processes, or represent responses of the innate immunity system of any cell also outside the nervous system. Therefore, MEFs were subjected to FCCP treatment and the transcriptional response of the key antiviral factors was studied in the presence or absence of PINK1. As shown in Additional file 11: Fig. S4 below, FCCP treatment of MEF cells after 24 h led to transcriptional upregulation of murine *Mavs*, accompanied by downregulation of *Rsad2*, *Ddx58*, *Tbk1*, *Irf3*, *Mfn1*, *Ifit3*, and *Ifit1*, both in control WT cells and *Pink1* ^−/−^ cells. Thus, again the mitochondrial antiviral signaling factor *Mavs* showed a selective dependence on mitochondrial dysfunction, whereas the levels of all other antiviral key factors decreased in parallel to the FCCP-triggered autophagic elimination of mitochondria. Fibroblasts tolerate a reduction of mitochondrial mass by prominent glycolysis, whereas neuronal cells depend on mitochondria and will compensate mitophagy with mitochondrial biogenesis [37]. Therefore, fibroblasts and neurons may respond to FCCP with opposing regulations of mitochondria-dependent innate immunity factor levels even in control cells.

### Systematic study of antiviral factor mRNAs in *PINK1*-deficient cells after activating innate immunity with poly(I:C) as a pathogenic RNA-analogue

**Fig. 4:**
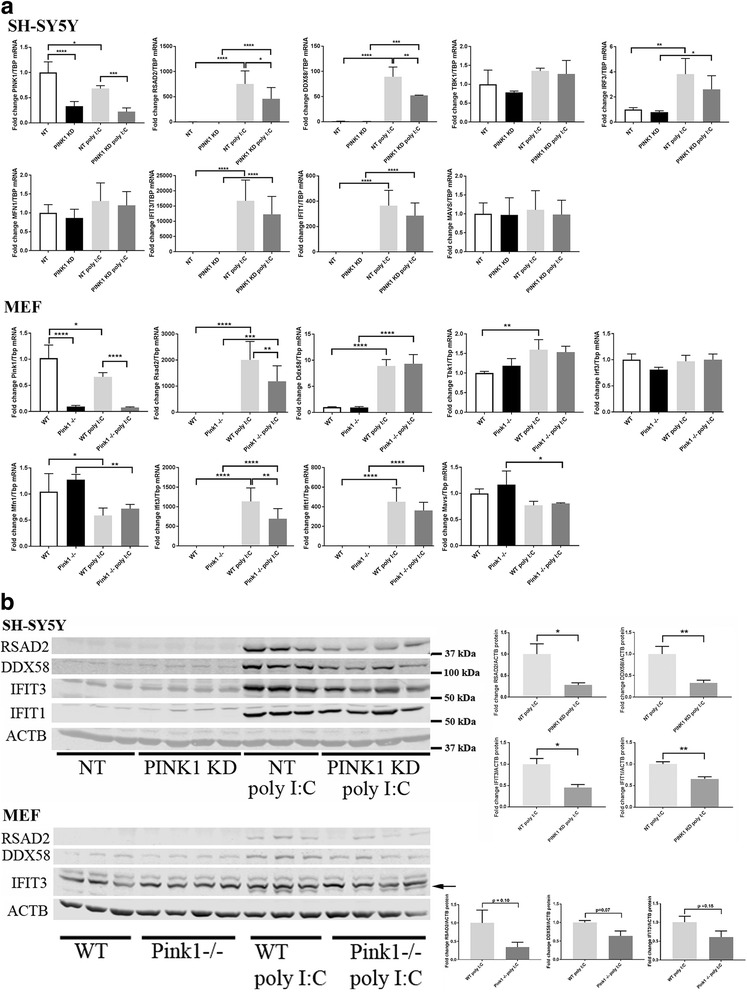
Poly(I:C) triggered expression responses of innate immunity factors in dependence on PINK1. **a** Transcriptional changes: SH-SY5Y human neuroblastoma (above) and murine embryonal fibroblast cells (below) were studied regarding the expression of key inflammatory factors in untreated versus drug-treated cells, comparing control with PINK1-deficiency (*n* = 8 each for *RSAD2*, *DDX58*, *IFIT3*, and *IFIT1* in man, and *n* = 4 each for *Rsad2*, *Ddx58*, *Ifit3*, *Ifit1* in mouse). *TBP* transcript levels were used as loading controls to normalize the data. The bar graphs show mean and standard error of the mean, illustrating the significance with asterisks (*p < 0.05, **p < 0.01, ***p < 0.001, ****p < 0.0001). **b** Protein changes: representative quantitative immunoblots and their statistical evaluation in bar graphs (*n* = 3 WT versus 4 mutant) showed a massive induction of the RNA sensors RSAD2, DDX58, IFIT3 (in human as well as mouse), and IFIT1 (human-specific antibody), which was blunted in PINK1-deficient cells (significance demonstrable only in neural cells). The beta-Actin protein level was used as loading control to normalize the quantitative results

In view of the strong PINK1-dependent dysregulation of several sensors of pathogenic RNA and of an RNA-virus budding suppressor, namely *RSAD2*, *IFIT3*, and *IFIT1* in the starvation experiment (Fig. 3c, h, i), we also stressed both cell types (neuroblastoma and MEF cells) with poly(I:C) as a pathogenic RNA-analogue and determined the transcriptional response after 16 h (Fig. 4a). Neuroblastoma cells responded by massive inductions of *RSAD2*, *DDX58*, *IFIT3*, and *IFIT1*, moderate induction of *IRF3*, and a downregulation of *PINK1*. The additional knockdown of *PINK1* significantly blunted the upregulations of *RSAD2* and *DDX58* in SH-SY5Y cells. In MEF cells, again a downregulation of *Pink1* was found, now paralleled by moderate downregulation of the other two mitochondrial outer membrane factors, *Mfn1* and *Mavs*. Massive inductions of *Rsad2*, *Ddx58*, *Ifit3*, and *Ifit1* reoccurred, now with a moderate increase of *Tbk1*. The *Pink1* deletion significantly blunted the upregulations of *Rsad2* and *Ifit3* in MEFs.

The substantial upregulations of RNA sensors and their modulation by PINK1 encouraged us to attempt validating these effects also at the protein level, so quantitative immunoblots were performed (Fig. 4b). In neuroblastoma cells, the poly(I:C) triggered inductions were significantly blunted by *PINK1* knockdown for RSAD2, DDX58, IFIT3, and IFIT1. In comparison, MEF cells also responded by strong inductions to poly(I:C), but the PINK1-dependent reductions of RSAD2, DDX58, and IFIT3 were not significant. IFIT1 could not be analyzed, since the available antibodies in our hands recognized only the human, but not the murine variant. Overall, these data clearly establish PINK1 as enhancer of anti-microbial responses and particularly of RNA sensors.

### Expression analysis of key antiviral factors in primary skin fibroblasts from PARK6 patients

**Fig. 5:**
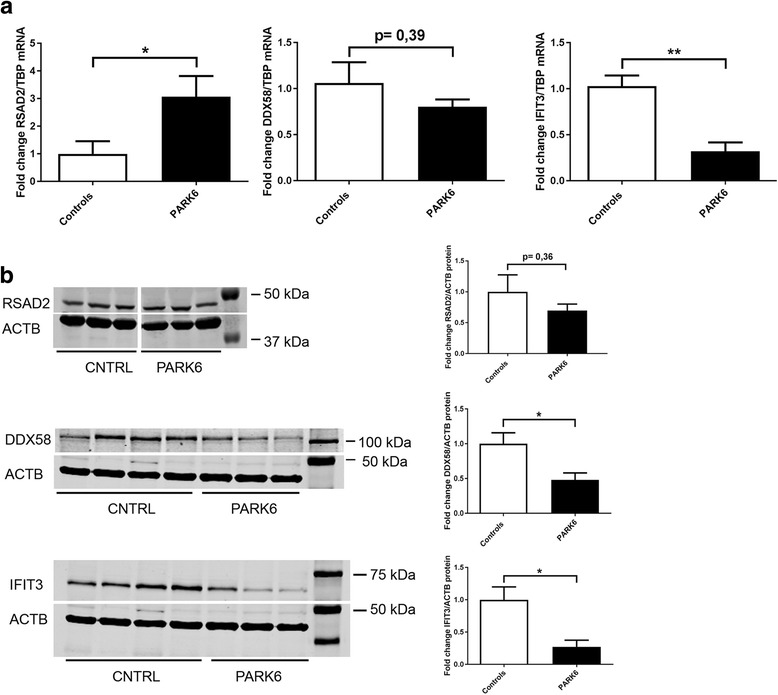
Altered antiviral factors in primary skin fibroblasts from PARK6 patients. **a** The levels of *RSAD2*, *DDX58*, and *IFIT3* transcript as well as (**b**) the levels of RSAD2, DDX58, and IFIT3 protein were assessed in unstressed cells from 3 homozygous G309D-PINK1 PD patients versus matched controls. The bar graphs show mean and standard error of the mean, illustrating the significance with asterisks (* *p* < 0.05 and ** *p* < 0.01)

Particularly strong and early PINK1-dependent effects were further assessed by qPCR in unstressed primary skin fibroblasts from PARK6 patients versus control individuals, which were previously characterized regarding mitochondrial dysfunction and expression profiles and were found to constitute a useful model of PD [1, 5, 12, 19, 41–43]. In the fibroblasts of homozygous PARK6 patients, *IFIT3* transcript was reduced to 31% (*p* = 0.0059), while *RSAD2* transcript was increased to 307% (*p* = 0.044) (Fig. 5a). These data in fibroblasts from manifest PARK6 patients at advanced age confirmed the previous findings in starving neuroblastoma cells, verifying that the loss of function of PINK1 leads to a deficit of the mitochondria-associated antiviral RNA sensor *IFIT3*, in parallel to a supersensitive induction of downstream *RSAD2* (viperin) as an inhibitor of many RNA and DNA viruses. In quantitative immunoblots, IFIT3 protein was reduced to 0.27-fold (±0.10, *p* = 0.03) (Fig. 5b). The RSAD2 protein levels were not significantly changed within this analysis of three patients versus three control samples. These data confirm that the IFIT3 mRNA downregulation is not offset by compensatory molecular efforts, but instead is translated into a deficit of this key antiviral factor at the protein level, thus altering innate immunity responses at the advanced age of manifest PARK6 patients.

## Discussion

The progression of pathology in PD tissues and its animal and cell models is being documented intensively at the clinical, histology, imaging, neurophysiology, and molecular levels, especially in cases with monogenic pathogenesis [140–143]. There is an urgent need of risk biomarkers for the presymptomatic detection and preventive therapy, as well as of progression biomarkers for the objective quantification of disease severity and of therapeutic benefits. However, the brain tissue from patients is available only at final stages of disease, and most available autopsies are from genetically undefined and therefore heterogeneous variants of PD. Conversely, the peripheral cells and tissues from patients reflect only some initial abnormalities of pathogenesis [19, 41, 43] and do not progress to a selective cell death. Thus, the analysis of brain tissue from postnatal age until the multimorbid old age, e.g., in PINK1-mutant or alpha-synuclein-mutant mice [30, 133, 144, 145], holds great promise. Several studies in the past have employed unbiased global OMICS approaches to screen the activity of practically all known genes [20–22, 29, 30]. Nonetheless, in spite of the enormous recent progress in PD genetics and in the characterization of corresponding disease models, it has remained difficult to identify and establish individual molecular progression biomarkers. It was expected that the levels of any such molecular marker would correlate with the severity of disease, similar to hemoglobin levels in anemia or to creatinine levels in kidney dysfunction.

Our novel progression analysis of global transcriptome profiles in PINK1-deficient brain tissue across lifespan now indicates that subsequent stages of pathology are characterized by the involvement of increasing numbers of subtly dysregulated pathways rather than stronger expression anomalies of individual candidates. This in vivo approach identified several pathways to be prominently PINK1-dependent, in good agreement with previous in vitro findings in most cases. Importantly, for the first time, we establish a temporal order and provide a quantitative value for the significance of each pathway.

In the scenario documented, a first disease stage is defined by a mild adaptation of the nuclear splicing machinery, which persists without increase throughout all ages. This is completely novel evidence; the notion of a splicing adaptation to PINK1-deficiency is currently supported only by an OMICS study into posttranslational modifications of the brain in a genetic mouse model of PD, where a strongly altered arginine-methylation of the splicing factor PSF was observed, caused either by PINK1 deficiency or by alpha-synuclein gain of function [22]. The transcriptional dysregulation of *Srsf10* as a spliceosome component was reproduced by qPCR. Given that spliceosomal alteration is a constant feature in *Pink1* ^−/−^ brain and that *Ube3a* and *Mapk8* are regulated by alternative splicing, we investigated the dysregulation of their splice isoforms and demonstrated a selective effect on the shorter splice isoform of UBE3A, a ubiquitination enzyme responsible for alpha-synuclein degradation [59].

A second disease stage shows manifest anomalies in the ubiquitin-dependent degradation of proteins and in the protein processing at the endoplasmic reticulum (ER). These data are in excellent agreement with the established role of PINK1 as a ubiquitin kinase [146]. Scarce evidence exists until now on the role of PINK1 as a modifier of ER stress [147–151], but our microarray biomathematics support the notion that alterations at the ER appear earlier than mitophagy and have stronger significance. Indeed, the transcriptional dysregulation of *Creb3* and *Nfkbia* as ER stress and inflammation factors was similarly reproduced by qPCR as the dysregulation of *Ube3a* as a component of ubiquitination pathways. Thus, both the ubiquitin kinase effects and the early alteration of widespread protein processing support a novel concept, where PINK1 has a general role for subcellular degradation rather than a function restricted to the selective elimination of dysfunctional mitochondria. Vesicular and lysosomal pathway dysregulations are more prominent at early ages upon GSEA bioinformatics than mitochondrial pathways.

A third stage, at old age in the *Pink1* ^−/−^ mouse, cerebellar tissue shows no change of the effect size in expression dysregulation in parallel with the progression of pathology, e.g., for the components of the splicing machinery. Instead, the number of dysregulated factors and pathways, the significance of the intracellular membrane-bounded organelle enrichment, and the appearance of altered neurotransmission, mitophagy, anti-microbial, and neuroinflammatory profiles were clearly age-associated. Our experimental validation of increased *Mapk8 and Nfkbia* transcript levels is in agreement with a previous report about *Pink1* ^−/−^ astrocyte JNK1 signaling [51] and with evidence for an interaction between PINK1 and the NF-kappaB pathway [152–156].

Indeed, neuroinflammation is well documented in late stages of PD [157–161]. Initially, it was thought to be triggered by the debris resulting from neuronal loss [162]. Further detailed study of genetic mouse and cell models demonstrated the neuroinflammation to precede neuronal loss [163], and current concepts propose alpha-synuclein aggregates and their extracellular extrusion to act as triggers of toll-like-receptor upregulation, cytokine release, and microglia activation [164–175]. In contrast, our observations demonstrate the enrichment of neuroinflammatory dysregulations quite early in the disease course, in brain tissues where alpha-synuclein aggregation was not detectable [29]. The concept that mitochondrial dysfunction alone is sufficient to modulate the innate immunity factors was also supported by our FCCP and poly(I:C) experiments in human neuroblastoma cells and murine embryonal fibroblasts. These data showed the mitochondrial antiviral signaling factor MAVS to be selectively responsive to proton gradient and PINK1 changes, and the induction of DDX58, IFIT3 and IFIT1 as sensors of pathogenic dsRNA and of RSAD2 as viral replication suppressor to be modified by PINK1.

Thus, we suggest that innate immunity is triggered within neurons via PINK1-associated mitochondrial dysfunction. Indeed, evidence has accumulated over the past 5 years that dysfunctional mitochondria are releasing damage-associated molecular patterns (DAMPs), e.g., the hypomethylated DNA/RNA and formylated peptides which are characteristic for bacteria and the mitochondrial endosymbiont [176, 177]. This release triggers the innate immune system and in particular a mitochondria-associated pathway of defense against abnormal DNA/RNA [178–180]. In good compatibility with this concept, recent findings confirm that neuroinflammation in PD can be modulated by the formyl-peptide receptors [181]. We have lately shown in another human hereditary disorder, Perrault syndrome, that mitochondrial dysfunction can be a strong trigger of the innate immune system via mtDNA accumulation, with subsequent early-onset infertility, growth deficits, and age-associated neurodegeneration [38]. In the pathways involved, the IFIT protein family together with DDX58 (RIG-I) is responsible of the recognition of pathogenic DNA/RNA in the cytosol, while RSAD2 (viperin) inhibits viral budding from membranes via lipid raft alteration. The mitochondria-associated MAVS/MFN1/IFIT3 complex then triggers phosphorylation and ubiquitination events that ultimately lead to NFkappaB-mediated nuclear regulations and to TBK1/IRF3-mediated interferon signals for neighboring cells [111, 182] (Fig. 3a). Of course, the resulting stress metabolism and impairment of cell growth together with alterations of mitochondrial calcium buffering would influence synaptic plasticity and excitability, contributing to the known alterations of calcium homeostasis and neural transmission in PARK6 mouse models [44, 143, 183].

A similar mechanism could operate in PINK1 and PARKIN deficient cells, which are being used as models of PD and are known to have an impairment of selective mitophagy, as was suggested by a recent report of high visibility that PARKIN also mediates resistance to microbial invasion [18]. The PINK1-dependent subtle molecular mismanagement of variably reoccurring life events such as infections (in our data mimicked by polyI:C) or hunger (mimicked by HBSS medium) may determine, if the clinical manifestation of Parkinson’s disease occurs early or late in life. It was recently demonstrated that the downregulation of Ataxin-2, a lipid-storage factor and mTOR-repressor upstream from PINK1, may postpone death in a mouse model of motor neuron disease from 20 to over 300 days [135, 184–186].

The innate immunity problems of PINK1-deficient cells become detectable even at the postnatal age upon the presence of a stressor. Stress exposure of *Pink1* ^−/−^ primary neuron-rich cultures leads to the significant expression dysregulation of the abnormal DNA/RNA sensor *Ifit3* and the iron-sulfur-cluster detector *Rsad2*, confirmed in Pink1-KO+A53T-SNCA double mutant brain, in human neuroblastoma cells, in *Pink1* ^−/−^ MEFs, and in PARK6 patient skin fibroblasts. The starvation dataset links PINK1 deficiency to a transcriptional downregulation of mitochondria-associated innate immunity factors such as *MAVS*, *MFN1*, and *IFIT3*, and the FCCP dataset shows altered expression of *MAVS* and *MFN* in MEFs and a PINK1-dependent blunting of *MAVS* induction in SH-SY5Y cells. MFN1 and MAVS are localized to the mitochondrial outer membrane, respond to an alteration in the mitochondrial proton gradient, and mediate the elimination of dysfunctional mitochondria via autophagy [187–190]. In addition, MAVS expression also modulates antiviral signaling via IFIT3 [112]. The starvation dataset also indicates a parallel PINK1-dependent transcriptional induction of downstream innate immunity factors such as *TBK1*, *IRF3*, and *RSAD2*. As shown in the poly(I:C) dataset at transcript and protein level, the regulation of IFIT3 and RSAD2 is altered by PINK1 deficiency. RSAD2 upregulation and mitochondrial relocalization triggers the lipogenesis for viral envelope formation [191, 192]. Beyond the established role of PINK1 for selective mitophagy, our novel data therefore indicate that PINK1 also modulates the mitochondria-associated anti-microbial defense pathway.

The changes in very young *Pink1* ^−/−^ brain and *Pink1* ^−/−^ cells are perfectly compatible with previous in vitro observations [84, 153, 154, 193–198]. Importantly, a highly visible publication was made during the final stage of our project, which reported PINK1 and Parkin in murine macrophages and fibroblasts to repress an immune-response eliciting pathway via the trafficking of mitochondrial-derived-vesicles, but not via mitophagy [199]. The present data from *Pink1* ^−/−^ mouse brain, human neuroblastoma cells, and patient fibroblasts serve as additional corroboration in vivo, providing a spatio-temporal framework and identifying crucial molecular mediators.

Still, as an important caveat, we have to mention several limitations of our findings: (I) Various technical approaches were focused on different brain regions. The previously published global transcriptome profiles had focused on midbrain/brainstem, which were selected because they are responsible for the characteristic mid-stage motor deficit of Parkinson’s disease. The re-assessment of these data showed these regions to be less informative within the 2-year lifespan of mice than the cerebellum. Consequently, for a deeper understanding of the subtle early consequences of *Pink1*-ablation, we focused the studies of histology and ceramides on the brainstem and olfactory bulb, where Parkinson’s disease starts. Furthermore, demonstration of the expected glial pathology uncovered a preferential affection of myelinated tracts. Thus, at present, it is cumbersome to extrapolate the expression data and other findings into a coherent picture of phenomenology and pathology progression in time and space, integrating observations from mouse and man, so further analyses in-depth are needed. (II) The impaired response of *Pink1* ^−/−^-deficient cells against polyI:C suggests a vulnerability towards viral infections. Although we present suggestive data at the transcript, protein, and cellular level, with several artificial stressors, this issue can only unequivocally be proven once *Pink1* ^−/−^ organisms are exposed to virus in future experiments, with quantification of viability and propagation rates.

## Conclusion

Our present findings support the concept that neuroinflammation in PD may occur long before neuronal loss and before the formation of alpha-synuclein aggregates. PINK1-mediated dysregulations of antimicrobial defense and dysfunctional mitochondria are activating the innate immunity.

The neuroinflammatory profile appeared in *Pink1*-deficient mouse brain after earlier ubiquitination and ER-associated degradation alterations, so these data might define markers of disease progression. Although PINK1-mutations are a rare cause of PD, PINK1 is upstream of several other Parkinson’s disease genes and its inactivity results in specific mitochondrial dysfunctions as a trigger for the selective neurodegenerative pattern that is frequent [200–203], so our findings are probably relevant for the multifactorial sporadic old-age patients with PD.

## Additional files

Additional file 1: Table S1. Global transcriptome profile of *Pink1* ^−/−^ brain regions (C = cerebellum, M = midbrain, S = striatum) across the mouse lifespan (6 W = 6 weeks, 24 W = 24 weeks = 6 months, 18 M = 18 months), showing all expression dysregulations which were consistent in all regions at all ages with significance (multiple testing adjusted *p* value <0.05). ID identifies the Affymetrix microarray chip probe. AveExpr quantifies the average expression strength among the tissues and ages for each transcript. *F*, *P* value, and AdjPvalue refer to statistical values. For each column, 3 mutant versus 3 WT tissues were analyzed to represent a specific brain region and age. (XLS 27 kb)

Additional file 2: Fig. S1. Global transcriptome profile of *Pink1*-deficient mouse cerebellar tissue at ages 6 weeks **(A)**, 6 months **(B)**, and 18 months **(C)**, illustrating the network with respect to interactions between the encoded proteins, employing the STRING web-server multiple proteins algorithm. (ZIP 11629 kb)

Additional file 3: Table S2. STRING bioinformatics assessment of all significant changes, including subtle effects. Ranked list of the significant transcript expression changes in PINK1-deficient mouse cerebellar tissue at ages 6 weeks, 6 months, and 18 months, which were used as input for the STRING analysis **(A)**. Significant functional enrichments in the protein-protein-interaction network according to the STRING database is shown regarding GO Cellular component at 6 weeks with all factors of AdjPvalue <0.05 **(B),** 6 months with all factors of AdjPvalue <0.05 (**C**), and 18 months with all factors of AdjPvalue <0.01, with the term “intracellular membrane-bounded organelle” being prominent at all 3 ages **(D).** Prominent enrichments were highlighted in yellow color. In GO Molecular Process at 18 months with all factors of AdjPvalue <0.01 the relevance of stimulus- and stress-dependent pathways is apparent, e.g., endoplasmic reticulum stress, and a significant enrichment of mitophagy factors appears here in lines 495 and 518 **(E)**. Among KEGG pathways at 18 months with all factors of AdjPvalue <0.01, the highest ranked pathways include “MAPK signaling”, “Ubiquitin-mediated proteolysis”, “Protein processing in endoplasmic reticulum” and “Bacterial invasion of epithelial cells” **(F)**. Orange color was used to highlight some MAPK-dependent synaptic signaling pathways with significant enrichment, purple color was used for ubiquitination and protein processing pathways, green color was used for neuroinflammation pathways. (ZIP 366 kb)

Additional file 4: Table S3. STRING bioinformatics assessment with high stringency of only >1.5-fold downregulations with significance. Ranked KEGG pathway dysregulations are shown for cerebellum at each age. The prominent MAPK signaling is highlighted in purple color, neuroinflammation pathways in red, ubiquitination pathways in blue, endoplasmic reticulum stress response pathways in orange, the mTOR signaling pathway (mimicked by starvation stress during in vitro experiments) in dark gray, the vesicular pathways in light brown. Subsequently, the interaction diagrams are shown for each age, with components of the prominent MAPK signaling pathway highlighted in red color. Finally, a heatmap shows all components of the MAPK signaling pathway with their expression changes at each age in cerebellum, with clearly progressive downregulation for several factors at the far end. (PDF 643 kb)

Additional file 5: Table S4. GSEA bioinformatics assessment—ranked pathways by age. Ranked pathway dysregulations are shown separately on different datasheets for the predefined Reactome and KEGG gene sets regarding down- and upregulations, and progressive ages. Green background colors for the nominal *p* value and the False Discovery Rate *q* values indicate significance according to GSEA recommendations. Neuroinflammation pathways are highlighted in red color, MAPK signaling in purple, Parkinson’s disease in yellow, ubiquitination pathways in blue, endoplasmic reticulum stress response pathways in orange, the mTOR signaling pathway (mimicked by starvation stress during in vitro experiments) in dark gray, the vesicular pathways in light brown. (XLSX 123 kb)

Additional file 6: Table S5. GSEA bioinformatics assessment—top dozen pathways with components. In *Pink1* ^−/−^ cerebellum at the age of 18 months the top dozen dysregulated KEGG pathways and the top dozen dysregulated Reactome pathways are shown with individual components. The nominal *p* values, false discovery rate *q* values and family wise error *p* values are shown on top, the Rank Metric Score indicates the log2 expression values for each factor, and YES reflects significance. (PDF 1540 kb)

Additional file 7: Table S6. GSEA bioinformatics assessment—heat maps. Heat maps to illustrate the progression of expression dysregulation for all individual components of selected pathways of relevance, namely (A) the Reactome pathway “Negative regulators of RIG I and MDA5 signaling”, (B) the Reactome pathway “Antiviral mechanism by IFN stimulated genes”, (C) the Reactome pathway “Antigen processing ubiquitination proteasome degradation”, and (D) the Reactome pathway “Activation of chaperone genes by Xbp1s”. (PDF 135 kb)

Additional file 8: Fig. S2. Transcript changes of *Pink1*-deficient mouse cerebellar tissue at the age of 18 months in qPCR analyses represented in bar graphs. **(A)** Significant downregulation of *Srsf10* mRNA and upregulations of *Creb3* and *Nfkbia* mRNAs confirm the alteration within spliceosomal, ER stress and neuroinflammation pathways. The significant dysregulation of a *Ube3a* splice isoform is particularly interesting as a potential target of the spliceosome alterations and in view of its role in the degradation of alpha-synuclein. The scheme of *Ube3a* exon intron structure with the location of different Taqman assays was adapted from the Thermo Fisher Scientific internet site. **(B)** Significant upregulations of *Mapk8* mRNA at 3 different exon junctions, together with a scheme of the *Mapk8* exon intron structure and the location of 3 different Taqman assays (modified from the Thermo Fisher Scientific internet site). Non-significant changes of the MAPK phosphorylation cascade components *Mapk9* and *Mapk14* mRNAs demonstrate the selectivity of transcriptional regulation. Significant upregulations in the downstream nuclear transcription regulators *Creb3* and *Nfkbia* in the stress and inflammation response may reflect biological responses to the *Mapk8* upregulation. The bar graphs show mean and standard error of the mean (10 *Pink1* ^−/−^ versus 10 WT), illustrating the significance with asterisks (* *p* < 0.05 and ** *p* < 0.01). (TIFF 13007 kb)

Additional file 9: Table S7. PINK1-deficient transcriptome profile of primary neuron cultures. Ranked list of significant expression changes in global transcriptome profile of primary neuron cultures at 12 days after dissection of brains at postnatal stage, taken from 3 *Pink1* ^−/−^ and 3 age- and sex- matched WT controls from the same ancestors. FC = fold change. (PDF 60 kb)

Additional file 10: Fig. S3. Transcription dysregulation of innate immunity factors in the midbrain of 18-month-old *Pink1*-deficient mice with chronic stress from A53T-SNCA overexpression. The levels of the stress-triggered pro-inflammatory factor *Mapk9*, the lipid-droplet associated antiviral effector *Rsad2* (viperin), the formylpeptide receptor activating *Hebp1*, and the immunostimulant *Tnf* (TNF-alpha) transcripts were assessed in brain from adult double mutant *Pink1* ^−/−^+A53T-SNCA mice. These data corroborate the brain tissue dysregulation of two factors that were previously found dysregulated in the global transcriptome profile of *Pink1* ^−/−^ primary neuron cultures, taken from postnatal mice and maintained in vitro over 12 days. Data are displayed as bar graphs, illustrating mean values and standard error of the mean (SEM). *Tbp* transcript levels were used as loading controls to normalize the data. Significant differences were highlighted with asterisks (*p < 0.05; **p < 0.01; ***p < 0.001). (TIFF 17632 kb)

Additional file 11: Fig. S4. Transcriptional response of innate immunity factors to 24 h treatment with uncoupling drug FCCP and subsequent mitophagy, in dependence on PINK1. Three independent experiments in SH-SY5Y human neuroblastoma (above) and murine embryonal fibroblast cells (below) documented the expression of key inflammatory factors in untreated versus drug-treated cells, comparing control with PINK1-deficiency. The bar graphs show mean and standard error of the mean, illustrating the significance with asterisks (* *p* < 0.05, ** *p* < 0.01, *** *p* < 0.001). (TIFF 538 kb)

## Abbreviations

AdjPvalue: Adjusted *P*-value
APP: Amyloid precursor protein
AveExpr: Average expression
BSA: Bovine serum albumin
cDNA: Complementary DNA
Cer: Ceramide
DAMPs: Damage-associated molecular patterns
DNA: Deoxyribonucleic acid
dsRNA: Double-stranded RNA
ER: Endoplasmic reticulum
ES: Enrichment score
Fc epsilon RI signaling: Fc epsilon RI refers to the high affinity immunoglobulin E receptor
Fc gamma R-mediated phagocytosis: Fc gamma receptors phagocytose opsonized microbes
FC: Fold change
FCS: Fetal calf serum
FDR: False discovery rate
GDNF: Glial cell line-derived neurotrophic factor
GlcCer: Glucosylceramide
GO: Gene Ontology consortium
HBSS: Hank’s buffered salt solution
HTLV-I: Human T-lymphotropic virus
KD: Knock-down
KEGG: Kyoto encyclopedia of genes and genomes
KO: Knock-out
LacCer: Lactosylceramide
MAPK: Mitogen-activated protein kinase
mtDNA: Mitochondrial DNA
NES: Negative enrichment score
NT: Non-target knock-down
OMICS: Studies into global cell content, comprising transcriptomics, proteomics, lipidomics
PCR: Polymerase chain reaction
PD: Parkinson’s disease
PINK1: PTEN-induced kinase 1
Poly(I:C): Polyinosinic-polycytidylic acid
qPCR: Quantitative reverse-transcriptase real-time PCR
RNA: Ribonucleic acid
RT: Room temperature
SEM: Standard error of the mean
SNCA: Alpha-synuclein
ssRNA: Single-strand RNA
STRING: Search tool for the retrieval of interacting genes
TBS-T: Tris-buffered saline with Tween-20
TRP: Transient receptor potential
WT: Wild-type

## References

[1] Valente EM, Abou-Sleiman PM, Caputo V, Muqit MM, Harvey K, Gispert S, Ali Z, Del Turco D, Bentivoglio AR, Healy DG (2004). Hereditary early-onset Parkinson’s disease caused by mutations in PINK1. Science.

[2] Wauer T, Simicek M, Schubert A, Komander D (2015). Mechanism of phospho-ubiquitin-induced PARKIN activation. Nature.

[3] Lazarou M, Sliter DA, Kane LA, Sarraf SA, Wang C, Burman JL, Sideris DP, Fogel AI, Youle RJ (2015). The ubiquitin kinase PINK1 recruits autophagy receptors to induce mitophagy. Nature.

[4] Ordureau A, Heo JM, Duda DM, Paulo JA, Olszewski JL, Yanishevski D, Rinehart J, Schulman BA, Harper JW (2015). Defining roles of PARKIN and ubiquitin phosphorylation by PINK1 in mitochondrial quality control using a ubiquitin replacement strategy. Proc Natl Acad Sci U S A.

[5] Gehrke S, Wu Z, Klinkenberg M, Sun Y, Auburger G, Guo S, Lu B (2015). PINK1 and Parkin control localized translation of respiratory chain component mRNAs on mitochondria outer membrane. Cell Metab.

[6] Parganlija D, Klinkenberg M, Dominguez-Bautista J, Hetzel M, Gispert S, Chimi MA, Drose S, Mai S, Brandt U, Auburger G, Jendrach M (2014). Loss of PINK1 impairs stress-induced autophagy and cell survival. PLoS One.

[7] Mai S, Klinkenberg M, Auburger G, Bereiter-Hahn J, Jendrach M (2010). Decreased expression of Drp1 and Fis1 mediates mitochondrial elongation in senescent cells and enhances resistance to oxidative stress through PINK1. J Cell Sci.

[8] Chen Y, Dorn GW (2013). PINK1-phosphorylated mitofusin 2 is a Parkin receptor for culling damaged mitochondria. Science.

[9] Scarffe LA, Stevens DA, Dawson VL, Dawson TM (2014). Parkin and PINK1: much more than mitophagy. Trends Neurosci.

[10] Heo JM, Ordureau A, Paulo JA, Rinehart J, Harper JW: The PINK1-PARKIN mitochondrial ubiquitylation pathway drives a program of OPTN/NDP52 recruitment and TBK1 activation to promote mitophagy. Mol Cell. 2015;60:7–20. 10.1016/j.molcel.2015.08.016 PMC4592482 26365381

[11] Geisler S, Holmstrom KM, Skujat D, Fiesel FC, Rothfuss OC, Kahle PJ, Springer W (2010). PINK1/Parkin-mediated mitophagy is dependent on VDAC1 and p62/SQSTM1. Nat Cell Biol.

[12] Exner N, Treske B, Paquet D, Holmstrom K, Schiesling C, Gispert S, Carballo-Carbajal I, Berg D, Hoepken HH, Gasser T (2007). Loss-of-function of human PINK1 results in mitochondrial pathology and can be rescued by parkin. J Neurosci.

[13] Klinkenberg M, Gispert S, Dominguez-Bautista JA, Braun I, Auburger G, Jendrach M (2012). Restriction of trophic factors and nutrients induces PARKIN expression. Neurogenetics.

[14] Mei Y, Zhang Y, Yamamoto K, Xie W, Mak TW, You H (2009). FOXO3a-dependent regulation of Pink1 (Park6) mediates survival signaling in response to cytokine deprivation. Proc Natl Acad Sci U S A.

[15] Klein P, Muller-Rischart AK, Motori E, Schonbauer C, Schnorrer F, Winklhofer KF, Klein R (2014). Ret rescues mitochondrial morphology and muscle degeneration of Drosophila Pink1 mutants. EMBO J.

[16] Meka DP, Muller-Rischart AK, Nidadavolu P, Mohammadi B, Motori E, Ponna SK, Aboutalebi H, Bassal M, Annamneedi A, Finckh B (2015). Parkin cooperates with GDNF/RET signaling to prevent dopaminergic neuron degeneration. J Clin Invest.

[17] O'Flanagan CH, O'Neill C (1846). PINK1 signalling in cancer biology. Biochim Biophys Acta.

[18] Manzanillo PS, Ayres JS, Watson RO, Collins AC, Souza G, Rae CS, Schneider DS, Nakamura K, Shiloh MU, Cox JS (2013). The ubiquitin ligase parkin mediates resistance to intracellular pathogens. Nature.

[19] Hoepken HH, Gispert S, Azizov M, Klinkenberg M, Ricciardi F, Kurz A, Morales-Gordo B, Bonin M, Riess O, Gasser T (2008). Parkinson patient fibroblasts show increased alpha-synuclein expression. Exp Neurol.

[20] Gispert S, Brehm N, Weil J, Seidel K, Rub U, Kern B, Walter M, Roeper J, Auburger G (2015). Potentiation of neurotoxicity in double-mutant mice with Pink1 ablation and A53T-SNCA overexpression. Hum Mol Genet.

[21] Auburger G, Gispert S, Jendrach M (2014). Mitochondrial acetylation and genetic models of Parkinson's disease. Prog Mol Biol Transl Sci.

[22] Auburger G, Gispert S, Brehm N (2016). Methyl-arginine profile of brain from aged PINK1-KO+A53T-SNCA mice suggests altered mitochondrial biogenesis. Parkinson's Dis.

[23] Head E, Powell D, Gold BT, Schmitt FA (2012). Alzheimer’s disease in Down syndrome. Eur J Neurodegener Dis.

[24] Olgiati S, Thomas A, Quadri M, Breedveld GJ, Graafland J, Eussen H, Douben H, de Klein A, Onofrj M, Bonifati V (2015). Early-onset parkinsonism caused by alpha-synuclein gene triplication: Clinical and genetic findings in a novel family. Parkinsonism Relat Disord.

[25] Konno T, Ross OA, Puschmann A, Dickson DW, Wszolek ZK (2016). Autosomal dominant Parkinson’s disease caused by SNCA duplications. Parkinsonism Relat Disord.

[26] Chiba-Falek O, Touchman JW, Nussbaum RL (2003). Functional analysis of intra-allelic variation at NACP-Rep1 in the alpha-synuclein gene. Hum Genet.

[27] Mellick GD, Maraganore DM, Silburn PA (2005). Australian data and meta-analysis lend support for alpha-synuclein (NACP-Rep1) as a risk factor for Parkinson’s disease. Neurosci Lett.

[28] Subramanian A, Tamayo P, Mootha VK, Mukherjee S, Ebert BL, Gillette MA, Paulovich A, Pomeroy SL, Golub TR, Lander ES, Mesirov JP (2005). Gene set enrichment analysis: a knowledge-based approach for interpreting genome-wide expression profiles. Proc Natl Acad Sci U S A.

[29] Gispert S, Ricciardi F, Kurz A, Azizov M, Hoepken HH, Becker D, Voos W, Leuner K, Muller WE, Kudin AP (2009). Parkinson phenotype in aged PINK1-deficient mice is accompanied by progressive mitochondrial dysfunction in absence of neurodegeneration. PLoS One.

[30] Kurz A, Double KL, Lastres-Becker I, Tozzi A, Tantucci M, Bockhart V, Bonin M, Garcia-Arencibia M, Nuber S, Schlaudraff F (2010). A53T-alpha-synuclein overexpression impairs dopamine signaling and striatal synaptic plasticity in old mice. PLoS One.

[31] Kurz A, Wohr M, Walter M, Bonin M, Auburger G, Gispert S, Schwarting RK (2010). Alpha-synuclein deficiency affects brain Foxp1 expression and ultrasonic vocalization. Neuroscience.

[32] Franceschini A, Szklarczyk D, Frankild S, Kuhn M, Simonovic M, Roth A, Lin J, Minguez P, Bork P, von Mering C, Jensen LJ (2013). STRING v9.1: protein-protein interaction networks, with increased coverage and integration. Nucleic Acids Res.

[33] Löffner F, Lohmann SM, Walckhoff B, Walter U, Hamprecht B (1986). Immunocytochemical characterization of neuron-rich primary cultures of embryonic rat brain cells by established neuronal and glial markers and by monospecific antisera against cyclic nucleotide-dependent protein kinases and the synaptic vesicle protein synapsin I. Brain Res.

[34] Schiffmann S, Sandner J, Birod K, Wobst I, Angioni C, Ruckhaberle E, Kaufmann M, Ackermann H, Lotsch J, Schmidt H (2009). Ceramide synthases and ceramide levels are increased in breast cancer tissue. Carcinogenesis.

[35] Zschiebsch K, Fischer C, Pickert G, Haussler A, Radeke H, Grosch S, Ferreiros N, Geisslinger G, Werner ER, Tegeder I (2016). Tetrahydrobiopterin attenuates DSS-evoked colitis in mice by rebalancing redox and lipid signalling. J Crohn's Colitis.

[36] Schmittgen TD, Livak KJ (2008). Analyzing real-time PCR data by the comparative C(T) method. Nat Protoc.

[37] Rakovic A, Shurkewitsch K, Seibler P, Grunewald A, Zanon A, Hagenah J, Krainc D, Klein C (2013). Phosphatase and tensin homolog (PTEN)-induced putative kinase 1 (PINK1)-dependent ubiquitination of endogenous Parkin attenuates mitophagy: study in human primary fibroblasts and induced pluripotent stem cell-derived neurons. J Biol Chem.

[38] Gispert S, Parganlija D, Klinkenberg M, Drose S, Wittig I, Mittelbronn M, Grzmil P, Koob S, Hamann A, Walter M (2013). Loss of mitochondrial peptidase Clpp leads to infertility, hearing loss plus growth retardation via accumulation of CLPX, mtDNA and inflammatory factors. Hum Mol Genet.

[39] Liu Y, Zhang YB, Liu TK, Gui JF (2013). Lineage-specific expansion of IFIT gene family: an insight into coevolution with IFN gene family. PLoS One.

[40] Ueta M, Kawai T, Yokoi N, Akira S, Kinoshita S (2011). Contribution of IPS-1 to polyI:C-induced cytokine production in conjunctival epithelial cells. Biochem Biophys Res Commun.

[41] Hoepken HH, Gispert S, Morales B, Wingerter O, Del Turco D, Mulsch A, Nussbaum RL, Muller K, Drose S, Brandt U (2007). Mitochondrial dysfunction, peroxidation damage and changes in glutathione metabolism in PARK6. Neurobiol Dis.

[42] Klinkenberg M, Thurow N, Gispert S, Ricciardi F, Eich F, Prehn JH, Auburger G, Kogel D (2010). Enhanced vulnerability of PARK6 patient skin fibroblasts to apoptosis induced by proteasomal stress. Neuroscience.

[43] Auburger G, Klinkenberg M, Drost J, Marcus K, Morales-Gordo B, Kunz WS, Brandt U, Broccoli V, Reichmann H, Gispert S, Jendrach M (2012). Primary skin fibroblasts as a model of Parkinson’s disease. Mol Neurobiol.

[44] Dehorter N, Lozovaya N, Mdzomba BJ, Michel FJ, Lopez C, Tsintsadze V, Tsintsadze T, Klinkenberg M, Gispert S, Auburger G, Hammond C (2012). Subthalamic lesion or levodopa treatment rescues giant GABAergic currents of PINK1-deficient striatum. J Neurosci.

[45] Shkreta L, Toutant J, Durand M, Manley JL, Chabot B (2016). SRSF10 connects DNA damage to the alternative splicing of transcripts encoding apoptosis, cell-cycle control, and DNA repair factors. Cell Rep.

[46] Wu S, Majeed SR, Evans TM, Camus MD, Wong NM, Schollmeier Y, Park M, Muppidi JR, Reboldi A, Parham P (2016). Clathrin light chains’ role in selective endocytosis influences antibody isotype switching. Proc Natl Acad Sci U S A.

[47] Cheng C, Sharp PA (2006). Regulation of CD44 alternative splicing by SRm160 and its potential role in tumor cell invasion. Mol Cell Biol.

[48] Fukuda A, Nakadai T, Shimada M, Hisatake K (2009). Heterogeneous nuclear ribonucleoprotein R enhances transcription from the naturally configured c-fos promoter in vitro. J Biol Chem.

[49] Reches A, Nachmani D, Berhani O, Duev-Cohen A, Shreibman D, Ophir Y, Seliger B, Mandelboim O (2016). HNRNPR regulates the expression of classical and nonclassical MHC class I proteins. J Immunol.

[50] Kim KS, Kim JS, Park JY, Suh YH, Jou I, Joe EH, Park SM (2013). DJ-1 associates with lipid rafts by palmitoylation and regulates lipid rafts-dependent endocytosis in astrocytes. Hum Mol Genet.

[51] Choi I, Kim J, Jeong HK, Kim B, Jou I, Park SM, Chen L, Kang UJ, Zhuang X, Joe EH (2013). PINK1 deficiency attenuates astrocyte proliferation through mitochondrial dysfunction, reduced AKT and increased p38 MAPK activation, and downregulation of EGFR. Glia.

[52] Park JH, Ko J, Park YS, Park J, Hwang J, Koh HC. Clearance of damaged mitochondria through PINK1 stabilization by JNK and ERK MAPK signaling in chlorpyrifos-treated neuroblastoma cells. Mol Neurobiol. 2016;54:1844–57. 10.1007/s12035-016-9753-1 26892626

[53] Kitada T, Pisani A, Porter DR, Yamaguchi H, Tscherter A, Martella G, Bonsi P, Zhang C, Pothos EN, Shen J (2007). Impaired dopamine release and synaptic plasticity in the striatum of PINK1-deficient mice. Proc Natl Acad Sci U S A.

[54] Martella G, Platania P, Vita D, Sciamanna G, Cuomo D, Tassone A, Tscherter A, Kitada T, Bonsi P, Shen J, Pisani A (2009). Enhanced sensitivity to group II mGlu receptor activation at corticostriatal synapses in mice lacking the familial parkinsonism-linked genes PINK1 or Parkin. Exp Neurol.

[55] Madeo G, Schirinzi T, Martella G, Latagliata EC, Puglisi F, Shen J, Valente EM, Federici M, Mercuri NB, Puglisi-Allegra S (2014). PINK1 heterozygous mutations induce subtle alterations in dopamine-dependent synaptic plasticity. Mov Disord.

[56] Feligioni M, Mango D, Piccinin S, Imbriani P, Iannuzzi F, Caruso A, De Angelis F, Blandini F, Mercuri NB, Pisani A, Nistico R (2016). Subtle alterations of excitatory transmission are linked to presynaptic changes in the hippocampus of PINK1-deficient mice. Synapse.

[57] Mishra A, Godavarthi SK, Maheshwari M, Goswami A, Jana NR (2009). The ubiquitin ligase E6-AP is induced and recruited to aggresomes in response to proteasome inhibition and may be involved in the ubiquitination of Hsp70-bound misfolded proteins. J Biol Chem.

[58] LaVoie MJ, Cortese GP, Ostaszewski BL, Schlossmacher MG (2007). The effects of oxidative stress on parkin and other E3 ligases. J Neurochem.

[59] Mulherkar SA, Sharma J, Jana NR (2009). The ubiquitin ligase E6-AP promotes degradation of alpha-synuclein. J Neurochem.

[60] Latour S, Aguilar C (2015). XIAP deficiency syndrome in humans. Semin Cell Dev Biol.

[61] Sandebring A, Thomas KJ, Beilina A, van der Brug M, Cleland MM, Ahmad R, Miller DW, Zambrano I, Cowburn RF, Behbahani H (2009). Mitochondrial alterations in PINK1 deficient cells are influenced by calcineurin-dependent dephosphorylation of dynamin-related protein 1. PLoS One.

[62] Su J, Yin J, Qin W, Sha S, Xu J, Jiang C (2015). Role for pro-inflammatory cytokines in regulating expression of GABA transporter type 1 and 3 in specific brain regions of kainic acid-induced status epilepticus. Neurochem Res.

[63] Lu K, Psakhye I, Jentsch S (2014). Autophagic clearance of polyQ proteins mediated by ubiquitin-Atg8 adaptors of the conserved CUET protein family. Cell.

[64] Hirota Y, Yamashita S, Kurihara Y, Jin X, Aihara M, Saigusa T, Kang D, Kanki T (2015). Mitophagy is primarily due to alternative autophagy and requires the MAPK1 and MAPK14 signaling pathways. Autophagy.

[65] Park JH, Ko J, Park YS, Park J, Hwang J, Koh HC (2017). Clearance of damaged mitochondria through PINK1 stabilization by JNK and ERK MAPK signaling in chlorpyrifos-treated neuroblastoma cells. Mol Neurobiol.

[66] Cang X, Wang X, Liu P, Wu X, Yan J, Chen J, Wu G, Jin Y, Xu F, Su J, Wan C (2016). PINK1 alleviates palmitate induced insulin resistance in HepG2 cells by suppressing ROS mediated MAPK pathways. Biochem Biophys Res Commun.

[67] Yamamoto K, Furukawa MT, Fukumura K, Kawamura A, Yamada T, Suzuki H, Hirose T, Sakamoto H, Inoue K (2016). Control of the heat stress-induced alternative splicing of a subset of genes by hnRNP K. Genes Cells.

[68] DenBoer LM, Hardy-Smith PW, Hogan MR, Cockram GP, Audas TE, Lu R (2005). Luman is capable of binding and activating transcription from the unfolded protein response element. Biochem Biophys Res Commun.

[69] Liang G, Audas TE, Li Y, Cockram GP, Dean JD, Martyn AC, Kokame K, Lu R (2006). Luman/CREB3 induces transcription of the endoplasmic reticulum (ER) stress response protein Herp through an ER stress response element. Mol Cell Biol.

[70] Jang SW, Kim YS, Lee YH, Ko J (2007). Role of human LZIP in differential activation of the NF-kappaB pathway that is induced by CCR1-dependent chemokines. J Cell Physiol.

[71] Jang SW, Kim YS, Kim YR, Sung HJ, Ko J (2007). Regulation of human LZIP expression by NF-kappaB and its involvement in monocyte cell migration induced by Lkn-1. J Biol Chem.

[72] Li X, Long J, He T, Belshaw R, Scott J (2015). Integrated genomic approaches identify major pathways and upstream regulators in late onset Alzheimer’s disease. Sci Rep.

[73] Ahmad A, Crupi R, Campolo M, Genovese T, Esposito E, Cuzzocrea S (2013). Absence of TLR4 reduces neurovascular unit and secondary inflammatory process after traumatic brain injury in mice. PLoS One.

[74] Perier C, Bove J, Wu DC, Dehay B, Choi DK, Jackson-Lewis V, Rathke-Hartlieb S, Bouillet P, Strasser A, Schulz JB (2007). Two molecular pathways initiate mitochondria-dependent dopaminergic neurodegeneration in experimental Parkinson’s disease. Proc Natl Acad Sci U S A.

[75] Ren H, Fu K, Mu C, Li B, Wang D, Wang G (2010). DJ-1, a cancer and Parkinson’s disease associated protein, regulates autophagy through JNK pathway in cancer cells. Cancer Lett.

[76] Dzamko N, Zhou J, Huang Y, Halliday GM (2014). Parkinson’s disease-implicated kinases in the brain; insights into disease pathogenesis. Front Mol Neurosci.

[77] Brown M, Strudwick N, Suwara M, Sutcliffe LK, Mihai AD, Ali AA, Watson JN, Schroder M (2016). An initial phase of JNK activation inhibits cell death early in the endoplasmic reticulum stress response. J Cell Sci.

[78] Han SY, Kim SH, Heasley LE (2002). Differential gene regulation by specific gain-of-function JNK1 proteins expressed in Swiss 3T3 fibroblasts. J Biol Chem.

[79] Dreskin SC, Thomas GW, Dale SN, Heasley LE (2001). Isoforms of Jun kinase are differentially expressed and activated in human monocyte/macrophage (THP-1) cells. J Immunol.

[80] Sentelle RD, Senkal CE, Jiang W, Ponnusamy S, Gencer S, Selvam SP, Ramshesh VK, Peterson YK, Lemasters JJ, Szulc ZM (2012). Ceramide targets autophagosomes to mitochondria and induces lethal mitophagy. Nat Chem Biol.

[81] Mayo L, Trauger SA, Blain M, Nadeau M, Patel B, Alvarez JI, Mascanfroni ID, Yeste A, Kivisakk P, Kallas K (2014). Regulation of astrocyte activation by glycolipids drives chronic CNS inflammation. Nat Med.

[82] Ferrazza R, Cogo S, Melrose H, Bubacco L, Greggio E, Guella G, Civiero L, Plotegher N (2016). LRRK2 deficiency impacts ceramide metabolism in brain. Biochem Biophys Res Commun.

[83] Wang G, Dinkins M, He Q, Zhu G, Poirier C, Campbell A, Mayer-Proschel M, Bieberich E (2012). Astrocytes secrete exosomes enriched with proapoptotic ceramide and prostate apoptosis response 4 (PAR-4): potential mechanism of apoptosis induction in Alzheimer disease (AD). J Biol Chem.

[84] Kim J, Byun JW, Choi I, Kim B, Jeong HK, Jou I, Joe E (2013). PINK1 deficiency enhances inflammatory cytokine release from acutely prepared brain slices. Exp Neurobiol.

[85] Bolon B, Dorman DC, Bonnefoi MS, Randall HW, Morgan KT (1993). Histopathologic approaches to chemical toxicity using primary cultures of dissociated neural cells grown in chamber slides. Toxicol Pathol.

[86] Ahlemeyer B, Kolker S, Zhu Y, Hoffmann GF, Krieglstein J (2003). Cytosine arabinofuranoside-induced activation of astrocytes increases the susceptibility of neurons to glutamate due to the release of soluble factors. Neurochem Int.

[87] Meier ID, Bernreuther C, Tilling T, Neidhardt J, Wong YW, Schulze C, Streichert T, Schachner M (2010). Short DNA sequences inserted for gene targeting can accidentally interfere with off-target gene expression. FASEB J.

[88] Meijer AH, van der Vaart M (2014). DRAM1 promotes the targeting of mycobacteria to selective autophagy. Autophagy.

[89] Tatura R, Kraus T, Giese A, Arzberger T, Buchholz M, Hoglinger G, Muller U. Parkinson’s disease: SNCA-, PARK2-, and LRRK2- targeting microRNAs elevated in cingulate gyrus. Parkinsonism Relat Disord. 2016;33:115–21. 10.1016/j.parkreldis.2016.09.028 27717584

[90] Zhao Y, Haney MJ, Gupta R, Bohnsack JP, He Z, Kabanov AV, Batrakova EV (2014). GDNF-transfected macrophages produce potent neuroprotective effects in Parkinson’s disease mouse model. PLoS One.

[91] Lynch C, Tristem M (2003). A co-opted gypsy-type LTR-retrotransposon is conserved in the genomes of humans, sheep, mice, and rats. Curr Biol.

[92] Syapin PJ (2008). Regulation of haeme oxygenase-1 for treatment of neuroinflammation and brain disorders. Br J Pharmacol.

[93] Koliaraki V, Kollias G (2011). A new role for myeloid HO-1 in the innate to adaptive crosstalk and immune homeostasis. Adv Exp Med Biol.

[94] Devosse T, Dutoit R, Migeotte I, De Nadai P, Imbault V, Communi D, Salmon I, Parmentier M (2011). Processing of HEBP1 by cathepsin D gives rise to F2L, the agonist of formyl peptide receptor 3. J Immunol.

[95] Le Blanc I, Luyet PP, Pons V, Ferguson C, Emans N, Petiot A, Mayran N, Demaurex N, Faure J, Sadoul R (2005). Endosome-to-cytosol transport of viral nucleocapsids. Nat Cell Biol.

[96] Li L, Cheng FW, Wang F, Jia B, Luo X, Zhang SQ (2014). The activation of TLR7 regulates the expression of VEGF, TIMP1, MMP2, IL-6, and IL-15 in Hela cells. Mol Cell Biochem.

[97] Shi M, Movius J, Dator R, Aro P, Zhao Y, Pan C, Lin X, Bammler TK, Stewart T, Zabetian CP (2015). Cerebrospinal fluid peptides as potential Parkinson disease biomarkers: a staged pipeline for discovery and validation. Mol Cell Proteomics.

[98] Phalke S, Nickel O, Walluscheck D, Hortig F, Onorati MC, Reuter G (2009). Retrotransposon silencing and telomere integrity in somatic cells of Drosophila depends on the cytosine-5 methyltransferase DNMT2. Nat Genet.

[99] Durdevic Z, Hanna K, Gold B, Pollex T, Cherry S, Lyko F, Schaefer M (2013). Efficient RNA virus control in Drosophila requires the RNA methyltransferase Dnmt2. EMBO Rep.

[100] Vivekanandan P, Daniel HD, Kannangai R, Martinez-Murillo F, Torbenson M (2010). Hepatitis B virus replication induces methylation of both host and viral DNA. J Virol.

[101] Thiagarajan D, Dev RR, Khosla S (2011). The DNA methyltranferase Dnmt2 participates in RNA processing during cellular stress. Epigenetics.

[102] Mattijssen S, Pruijn GJ (2012). Viperin, a key player in the antiviral response. Microbes Infect/Institut Pasteur.

[103] Goossens KE, Karpala AJ, Rohringer A, Ward A, Bean AG (2015). Characterisation of chicken viperin. Mol Immunol.

[104] Helbig KJ, Beard MR (2014). The role of viperin in the innate antiviral response. J Mol Biol.

[105] Saitoh T, Satoh T, Yamamoto N, Uematsu S, Takeuchi O, Kawai T, Akira S (2011). Antiviral protein Viperin promotes Toll-like receptor 7- and Toll-like receptor 9-mediated type I interferon production in plasmacytoid dendritic cells. Immunity.

[106] Qiu LQ, Cresswell P, Chin KC (2009). Viperin is required for optimal Th2 responses and T-cell receptor-mediated activation of NF-kappaB and AP-1. Blood.

[107] Yatabe T, Mochizuki S, Takizawa M, Chijiiwa M, Okada A, Kimura T, Fujita Y, Matsumoto H, Toyama Y, Okada Y (2009). Hyaluronan inhibits expression of ADAMTS4 (aggrecanase-1) in human osteoarthritic chondrocytes. Ann Rheum Dis.

[108] Phillips KL, Jordan-Mahy N, Nicklin MJ, Le Maitre CL (2013). Interleukin-1 receptor antagonist deficient mice provide insights into pathogenesis of human intervertebral disc degeneration. Ann Rheum Dis.

[109] Satoh K, Suzuki N, Yokota H (2000). ADAMTS-4 (a disintegrin and metalloproteinase with thrombospondin motifs) is transcriptionally induced in beta-amyloid treated rat astrocytes. Neurosci Lett.

[110] Boussadia O, Niepmann M, Creancier L, Prats AC, Dautry F, Jacquemin-Sablon H (2003). Unr is required in vivo for efficient initiation of translation from the internal ribosome entry sites of both rhinovirus and poliovirus. J Virol.

[111] Zhou X, Michal JJ, Zhang L, Ding B, Lunney JK, Liu B, Jiang Z (2013). Interferon induced IFIT family genes in host antiviral defense. Int J Biol Sci.

[112] Liu XY, Chen W, Wei B, Shan YF, Wang C (2011). IFN-induced TPR protein IFIT3 potentiates antiviral signaling by bridging MAVS and TBK1. J Immunol.

[113] Bai L, Merchant JL (2001). ZBP-89 promotes growth arrest through stabilization of p53. Mol Cell Biol.

[114] Sayin VI, Nilton A, Ibrahim MX, Agren P, Larsson E, Petit MM, Hulten LM, Stahlman M, Johansson BR, Bergo MO, Lindahl P (2013). Zfp148 deficiency causes lung maturation defects and lethality in newborn mice that are rescued by deletion of p53 or antioxidant treatment. PLoS One.

[115] Nigg EA (1996). Cyclin-dependent kinase 7: at the cross-roads of transcription, DNA repair and cell cycle control?. Curr Opin Cell Biol.

[116] Ko LJ, Shieh SY, Chen X, Jayaraman L, Tamai K, Taya Y, Prives C, Pan ZQ (1997). p53 is phosphorylated by CDK7-cyclin H in a p36MAT1-dependent manner. Mol Cell Biol.

[117] Nilson KA, Guo J, Turek ME, Brogie JE, Delaney E, Luse DS, Price DH (2015). THZ1 reveals roles for Cdk7 in co-transcriptional capping and pausing. Mol Cell.

[118] Aulas A, Stabile S, Vande Velde C (2012). Endogenous TDP-43, but not FUS, contributes to stress granule assembly via G3BP. Mol Neurodegener.

[119] Ash PE, Vanderweyde TE, Youmans KL, Apicco DJ, Wolozin B (2014). Pathological stress granules in Alzheimer’s disease. Brain Res.

[120] Heck MV, Azizov M, Stehning T, Walter M, Kedersha N, Auburger G (2014). Dysregulated expression of lipid storage and membrane dynamics factors in Tia1 knockout mouse nervous tissue. Neurogenetics.

[121] Waterfield M, Khan IS, Cortez JT, Fan U, Metzger T, Greer A, Fasano K, Martinez-Llordella M, Pollack JL, Erle DJ (2014). The transcriptional regulator Aire coopts the repressive ATF7ip-MBD1 complex for the induction of immunotolerance. Nat Immunol.

[122] Liu Q, Liu L, Zhao Y, Zhang J, Wang D, Chen J, He Y, Wu J, Zhang Z, Liu Z (2011). Hypoxia induces genomic DNA demethylation through the activation of HIF-1alpha and transcriptional upregulation of MAT2A in hepatoma cells. Mol Cancer Ther.

[123] Liu Q, Chen J, Liu L, Zhang J, Wang D, Ma L, He Y, Liu Y, Liu Z, Wu J (2011). The X protein of hepatitis B virus inhibits apoptosis in hepatoma cells through enhancing the methionine adenosyltransferase 2A gene expression and reducing S-adenosylmethionine production. J Biol Chem.

[124] Blanco MG, Matos J (2015). Hold your horSSEs: controlling structure-selective endonucleases MUS81 and Yen1/GEN1. Front Genet.

[125] Dehe PM, Coulon S, Scaglione S, Shanahan P, Takedachi A, Wohlschlegel JA, Yates JR, Llorente B, Russell P, Gaillard PH (2013). Regulation of Mus81-Eme1 Holliday junction resolvase in response to DNA damage. Nat Struct Mol Biol.

[126] Hofmann TG, Glas C, Bitomsky N (2013). HIPK2: A tumour suppressor that controls DNA damage-induced cell fate and cytokinesis. BioEssays.

[127] Nardinocchi L, Puca R, Givol D, D'Orazi G (2010). HIPK2-a therapeutical target to be (re)activated for tumor suppression: role in p53 activation and HIF-1alpha inhibition. Cell Cycle.

[128] Findlay VJ, Tapiero H, Townsend DM (2005). Sulfiredoxin: a potential therapeutic agent?. Biomed Pharmacother.

[129] Roychoudhury J, Clark JP, Gracia-Maldonado G, Unnisa Z, Wunderlich M, Link KA, Dasgupta N, Aronow B, Huang G, Mulloy JC, Kumar AR (2015). MEIS1 regulates an HLF-oxidative stress axis in MLL-fusion gene leukemia. Blood.

[130] Otto T, Fandrey J (2008). Thyroid hormone induces hypoxia-inducible factor 1alpha gene expression through thyroid hormone receptor beta/retinoid x receptor alpha-dependent activation of hepatic leukemia factor. Endocrinology.

[131] Kleven MD, Dlakic M, Lawrence CM (2015). Characterization of a single b-type heme, FAD, and metal binding sites in the transmembrane domain of six-transmembrane epithelial antigen of the prostate (STEAP) family proteins. J Biol Chem.

[132] Hagele S, Behnam B, Borter E, Wolfe J, Paasch U, Lukashev D, Sitkovsky M, Wenger RH, Katschinski DM (2006). TSGA10 prevents nuclear localization of the hypoxia-inducible factor (HIF)-1alpha. FEBS Lett.

[133] Gispert S, Kurz A, Brehm N, Rau K, Walter M, Riess O, Auburger G (2015). Complexin-1 and Foxp1 expression changes are novel brain effects of alpha-synuclein pathology. Mol Neurobiol.

[134] Schwarting RK, Sedelis M, Hofele K, Auburger GW, Huston JP (1999). Strain-dependent recovery of open-field behavior and striatal dopamine deficiency in the mouse MPTP model of Parkinson’s disease. Neurotox Res.

[135] Sen NE, Drost J, Gispert S, Torres-Odio S, Damrath E, Klinkenberg M, Hamzeiy H, Akdal G, Gulluoglu H, Basak AN, Auburger G (2016). Search for SCA2 blood RNA biomarkers highlights Ataxin-2 as strong modifier of the mitochondrial factor PINK1 levels. Neurobiol Dis.

[136] Sen NE, Gispert S, Auburger G (2017). PINK1 and Ataxin-2 as modifiers of growth. Oncotarget.

[137] Auburger G, Sen NE, Meierhofer D, Basak AN, Gitler AD. Efficient prevention of neurodegenerative diseases by depletion of starvation response factor Ataxin-2. Trends Neurosci. 2017;40:507–16. 10.1016/j.tins.2017.06.004 28684172

[138] Becker LA, Huang B, Bieri G, Ma R, Knowles DA, Jafar-Nejad P, Messing J, Kim HJ, Soriano A, Auburger G (2017). Therapeutic reduction of ataxin-2 extends lifespan and reduces pathology in TDP-43 mice. Nature.

[139] Azkona G, Lopez de Maturana R, Del Rio P, Sousa A, Vazquez N, Zubiarrain A, Jimenez-Blasco D, Bolanos JP, Morales B, Auburger G, et al. LRRK2 expression is deregulated in fibroblasts and neurons from Parkinson patients with mutations in PINK1. Mol Neurobiol. 2016. doi:10.1007/s12035-016-0303-7. 10.1007/s12035-016-0303-7 PMC5808058 27975167

[140] Hernandez DG, Paisan-Ruiz C, McInerney-Leo A, Jain S, Meyer-Lindenberg A, Evans EW, Berman KF, Johnson J, Auburger G, Schaffer AA (2005). Clinical and positron emission tomography of Parkinson's disease caused by LRRK2. Ann Neurol.

[141] Braak H, Bohl JR, Muller CM, Rub U, de Vos RA, Del Tredici K (2006). Stanley Fahn Lecture 2005: the staging procedure for the inclusion body pathology associated with sporadic Parkinson’s disease reconsidered. Mov Dis.

[142] Tuin I, Voss U, Kessler K, Krakow K, Hilker R, Morales B, Steinmetz H, Auburger G (2008). Sleep quality in a family with hereditary parkinsonism (PARK6). Sleep Med.

[143] Kessler KR, Hamscho N, Morales B, Menzel C, Barrero F, Vives F, Gispert S, Auburger G (2005). Dopaminergic function in a family with the PARK6 form of autosomal recessive Parkinson's syndrome. J Neural Transm.

[144] Platt NJ, Gispert S, Auburger G, Cragg SJ (2012). Striatal dopamine transmission is subtly modified in human A53Talpha-synuclein overexpressing mice. PLoS One.

[145] Subramaniam M, Althof D, Gispert S, Schwenk J, Auburger G, Kulik A, Fakler B, Roeper J (2014). Mutant alpha-synuclein enhances firing frequencies in dopamine substantia nigra neurons by oxidative impairment of A-type potassium channels. J Neurosci.

[146] Durcan TM, Fon EA (2015). The three ‘P’s of mitophagy: PARKIN, PINK1, and post-translational modifications. Genes Dev.

[147] Samann J, Hegermann J, von Gromoff E, Eimer S, Baumeister R, Schmidt E (2009). Caenorhabditits elegans LRK-1 and PINK-1 act antagonistically in stress response and neurite outgrowth. J Biol Chem.

[148] Li L, Hu GK. Pink1 protects cortical neurons from thapsigargin-induced oxidative stress and neuronal apoptosis. Biosci Rep. 2015;35:e00174. 10.1042/BSR20140104 PMC4340272 25608948

[149] Erpapazoglou Z, Corti O (2015). The endoplasmic reticulum/mitochondria interface: a subcellular platform for the orchestration of the functions of the PINK1-Parkin pathway?. Biochem Soc Trans.

[150] Van Laar VS, Roy N, Liu A, Rajprohat S, Arnold B, Dukes AA, Holbein CD, Berman SB (2015). Glutamate excitotoxicity in neurons triggers mitochondrial and endoplasmic reticulum accumulation of Parkin, and, in the presence of N-acetyl cysteine, mitophagy. Neurobiol Dis.

[151] Celardo I, Costa AC, Lehmann S, Jones C, Wood N, Mencacci NE, Mallucci GR, Loh SH, Martins LM (2016). Mitofusin-mediated ER stress triggers neurodegeneration in pink1/parkin models of Parkinson’s disease. Cell Death Dis.

[152] Sha D, Chin LS, Li L (2010). Phosphorylation of parkin by Parkinson disease-linked kinase PINK1 activates parkin E3 ligase function and NF-kappaB signaling. Hum Mol Genet.

[153] Akundi RS, Huang Z, Eason J, Pandya JD, Zhi L, Cass WA, Sullivan PG, Bueler H (2011). Increased mitochondrial calcium sensitivity and abnormal expression of innate immunity genes precede dopaminergic defects in Pink1-deficient mice. PLoS One.

[154] Lee HJ, Jang SH, Kim H, Yoon JH, Chung KC (2012). PINK1 stimulates interleukin-1beta-mediated inflammatory signaling via the positive regulation of TRAF6 and TAK1. Cell Mol Life Sci.

[155] Duan X, Tong J, Xu Q, Wu Y, Cai F, Li T, Song W (2014). Upregulation of human PINK1 gene expression by NFkappaB signalling. Mol Brain.

[156] Lim GG, Chua DS, Basil AH, Chan HY, Chai C, Arumugam T, Lim KL (2015). Cytosolic PTEN-induced Putative Kinase 1 Is Stabilized by the NF-kappaB Pathway and Promotes Non-selective Mitophagy. J Biol Chem.

[157] Nolan YM, Sullivan AM, Toulouse A (2013). Parkinson’s disease in the nuclear age of neuroinflammation. Trends Mol Med.

[158] More SV, Kumar H, Kim IS, Song SY, Choi DK (2013). Cellular and molecular mediators of neuroinflammation in the pathogenesis of Parkinson’s disease. Mediat Inflamm.

[159] Hunot S, Hirsch EC (2003). Neuroinflammatory processes in Parkinson's disease. Ann Neurol.

[160] Ouchi Y, Yagi S, Yokokura M, Sakamoto M (2009). Neuroinflammation in the living brain of Parkinson's disease. Parkinsonism Relat Disord.

[161] Dzamko N, Geczy CL, Halliday GM (2015). Inflammation is genetically implicated in Parkinson's disease. Neuroscience.

[162] Mosley RL, Benner EJ, Kadiu I, Thomas M, Boska MD, Hasan K, Laurie C, Gendelman HE (2006). Neuroinflammation, oxidative stress and the pathogenesis of Parkinson’s disease. Clin Neurosci Res.

[163] Sekiyama K, Sugama S, Fujita M, Sekigawa A, Takamatsu Y, Waragai M, Takenouchi T, Hashimoto M (2012). Neuroinflammation in Parkinson’s disease and related disorders: a lesson from genetically manipulated mouse models of alpha-synucleinopathies. Parkinson’s Dis.

[164] Sanchez-Guajardo V, Tentillier N, Romero-Ramos M (2015). The relation between alpha-synuclein and microglia in Parkinson’s disease: recent developments. Neuroscience.

[165] Moehle MS, West AB (2015). M1 and M2 immune activation in Parkinson’s disease: foe and ally?. Neuroscience.

[166] Hirsch EC, Jenner P, Przedborski S (2013). Pathogenesis of Parkinson’s disease. Mov Dis.

[167] Lema Tome CM, Tyson T, Rey NL, Grathwohl S, Britschgi M, Brundin P (2013). Inflammation and alpha-synuclein’s prion-like behavior in Parkinson’s disease—is there a link?. Mol Neurobiol.

[168] Beraud D, Twomey M, Bloom B, Mittereder A, Ton V, Neitzke K, Chasovskikh S, Mhyre TR, Maguire-Zeiss KA (2011). Alpha-synuclein alters toll-like receptor expression. Front Neurosci.

[169] Lastres-Becker I, Ulusoy A, Innamorato NG, Sahin G, Rabano A, Kirik D, Cuadrado A (2012). Alpha-synuclein expression and Nrf2 deficiency cooperate to aggravate protein aggregation, neuronal death and inflammation in early-stage Parkinson’s disease. Hum Mol Genet.

[170] Gao HM, Kotzbauer PT, Uryu K, Leight S, Trojanowski JQ, Lee VM (2008). Neuroinflammation and oxidation/nitration of alpha-synuclein linked to dopaminergic neurodegeneration. J Neurosci.

[171] Theodore S, Cao S, McLean PJ, Standaert DG (2008). Targeted overexpression of human alpha-synuclein triggers microglial activation and an adaptive immune response in a mouse model of Parkinson disease. J Neuropathol Exp Neurol.

[172] Kim C, Ho DH, Suk JE, You S, Michael S, Kang J, Joong Lee S, Masliah E, Hwang D, Lee HJ, Lee SJ (2013). Neuron-released oligomeric alpha-synuclein is an endogenous agonist of TLR2 for paracrine activation of microglia. Nat Commun.

[173] Reynolds AD, Glanzer JG, Kadiu I, Ricardo-Dukelow M, Chaudhuri A, Ciborowski P, Cerny R, Gelman B, Thomas MP, Mosley RL, Gendelman HE (2008). Nitrated alpha-synuclein-activated microglial profiling for Parkinson’s disease. J Neurochem.

[174] Tang Y, Le W (2016). Differential roles of M1 and M2 microglia in neurodegenerative diseases. Mol Neurobiol.

[175] Schapansky J, Nardozzi JD, LaVoie MJ (2015). The complex relationships between microglia, alpha-synuclein, and LRRK2 in Parkinson’s disease. Neuroscience.

[176] Zhang Q, Raoof M, Chen Y, Sumi Y, Sursal T, Junger W, Brohi K, Itagaki K, Hauser CJ (2010). Circulating mitochondrial DAMPs cause inflammatory responses to injury. Nature.

[177] Manfredi AA, Rovere-Querini P (2010). The mitochondrion—a Trojan horse that kicks off inflammation?. N Engl J Med.

[178] West AP, Khoury-Hanold W, Staron M, Tal MC, Pineda CM, Lang SM, Bestwick M, Duguay BA, Raimundo N, MacDuff DA (2015). Mitochondrial DNA stress primes the antiviral innate immune response. Nature.

[179] Arnoult D, Soares F, Tattoli I, Girardin SE (2011). Mitochondria in innate immunity. EMBO Rep.

[180] Scott I (2010). The role of mitochondria in the mammalian antiviral defense system. Mitochondrion.

[181] Mollica A, Stefanucci A, Costante R, Pinnen F (2012). Role of formyl peptide receptors (FPR) in abnormal inflammation responses involved in neurodegenerative diseases. Anti-inflamm Anti-allergy Agents Med Chem.

[182] Diamond MS, Farzan M (2013). The broad-spectrum antiviral functions of IFIT and IFITM proteins. Nat Rev Immunol.

[183] Carron R, Filipchuk A, Nardou R, Singh A, Michel FJ, Humphries MD, Hammond C (2014). Early hypersynchrony in juvenile PINK1(−)/(−) motor cortex is rescued by antidromic stimulation. Front Syst Neurosci.

[184] Becker LA, Huang B, Bieri G, Ma R, Knowles DA, Jafar-Nejad P, Messing J, Kim HJ, Soriano A, Auburger G, et al. Therapeutic reduction of ataxin 2 extends lifespan and reduces pathology in TDP-43 mice. Nature. 2017;544:367–71. 10.1038/nature22038 PMC5642042 28405022

[185] Lastres-Becker I, Nonis D, Eich F, Klinkenberg M, Gorospe M, Kotter P, Klein FA, Kedersha N, Auburger G (1862). Mammalian ataxin-2 modulates translation control at the pre-initiation complex via PI3K/mTOR and is induced by starvation. Biochim Biophys Acta.

[186] Bar DZ, Charar C, Dorfman J, Yadid T, Tafforeau L, Lafontaine DL, Gruenbaum Y (2016). Cell size and fat content of dietary-restricted Caenorhabditis elegans are regulated by ATX-2, an mTOR repressor. Proc Natl Acad Sci U S A.

[187] Sun X, Sun L, Zhao Y, Li Y, Lin W, Chen D, Sun Q (2016). MAVS maintains mitochondrial homeostasis via autophagy. Cell Discov.

[188] Ziviani E, Tao RN, Whitworth AJ (2010). Drosophila parkin requires PINK1 for mitochondrial translocation and ubiquitinates mitofusin. Proc Natl Acad Sci U S A.

[189] Gegg ME, Cooper JM, Chau KY, Rojo M, Schapira AH, Taanman JW (2010). Mitofusin 1 and mitofusin 2 are ubiquitinated in a PINK1/parkin-dependent manner upon induction of mitophagy. Hum Mol Genet.

[190] Koshiba T, Yasukawa K, Yanagi Y, Kawabata S (2011). Mitochondrial membrane potential is required for MAVS-mediated antiviral signaling. Sci Signal.

[191] Seo JY, Cresswell P (2013). Viperin regulates cellular lipid metabolism during human cytomegalovirus infection. PLoS Pathog.

[192] Seo JY, Yaneva R, Hinson ER, Cresswell P (2011). Human cytomegalovirus directly induces the antiviral protein viperin to enhance infectivity. Science.

[193] Ellis GI, Zhi L, Akundi R, Bueler H, Marti F (2013). Mitochondrial and cytosolic roles of PINK1 shape induced regulatory T-cell development and function. Eur J Immunol.

[194] Gurung P, Lukens JR, Kanneganti TD (2015). Mitochondria: diversity in the regulation of the NLRP3 inflammasome. Trends Mol Med.

[195] Lee HJ, Chung KC (2012). PINK1 positively regulates IL-1beta-mediated signaling through Tollip and IRAK1 modulation. J Neuroinflammation.

[196] Yunfu W, Guangjian L, Ping Z, Yanpeng S, Xiaoxia F, Wei H, Jiang Y, Jingquan H, Songlin W, Hongyan Z (2014). PINK1 and its familial Parkinson's disease-associated mutation regulate brain vascular endothelial inflammation. J Mol Neurosci.

[197] Kang R, Zeng L, Xie Y, Yan Z, Zhou B, Cao L, Klionsky DJ, Tracey KJ, Li J, Wang H, et al. A novel PINK1- and PARK2-dependent protective neuroimmune pathway in lethal sepsis. Autophagy. 2016;12:2374–85. 10.1080/15548627.2016.1239678 PMC5173260 27754761

[198] Zhu J, Qu Y, Lin Z, Zhao F, Zhang L, Huang Y, Jiang C, Mu D (1653). Loss of PINK1 inhibits apoptosis by upregulating alpha-synuclein in inflammation-sensitized hypoxic-ischemic injury in the immature brains. Brain Res.

[199] Matheoud D, Sugiura A, Bellemare-Pelletier A, Laplante A, Rondeau C, Chemali M, Fazel A, Bergeron JJ, Trudeau LE, Burelle Y (2016). Parkinson’s disease-related proteins PINK1 and Parkin repress mitochondrial antigen presentation. Cell.

[200] Ryan BJ, Hoek S, Fon EA, Wade-Martins R (2015). Mitochondrial dysfunction and mitophagy in Parkinson’s: from familial to sporadic disease. Trends Biochem Sci.

[201] Celardo I, Martins LM, Gandhi S (2014). Unravelling mitochondrial pathways to Parkinson’s disease. Br J Pharmacol.

[202] de Vries RL, Przedborski S (2013). Mitophagy and Parkinson’s disease: be eaten to stay healthy. Mol Cell Neurosci.

[203] Corti O, Lesage S, Brice A (2011). What genetics tells us about the causes and mechanisms of Parkinson’s disease. Physiol Rev.

