# Supplementary figures and images for: Progression of pathology in PINK1-deficient mouse brain from splicing via ubiquitination, ER stress, and mitophagy changes to neuroinflammation
Source: J Neuroinflammation. 2017 Aug 2;14:154. doi: 10.1186/s12974-017-0928-0 (PMC5541666; doi:10.1186/s12974-017-0928-0)

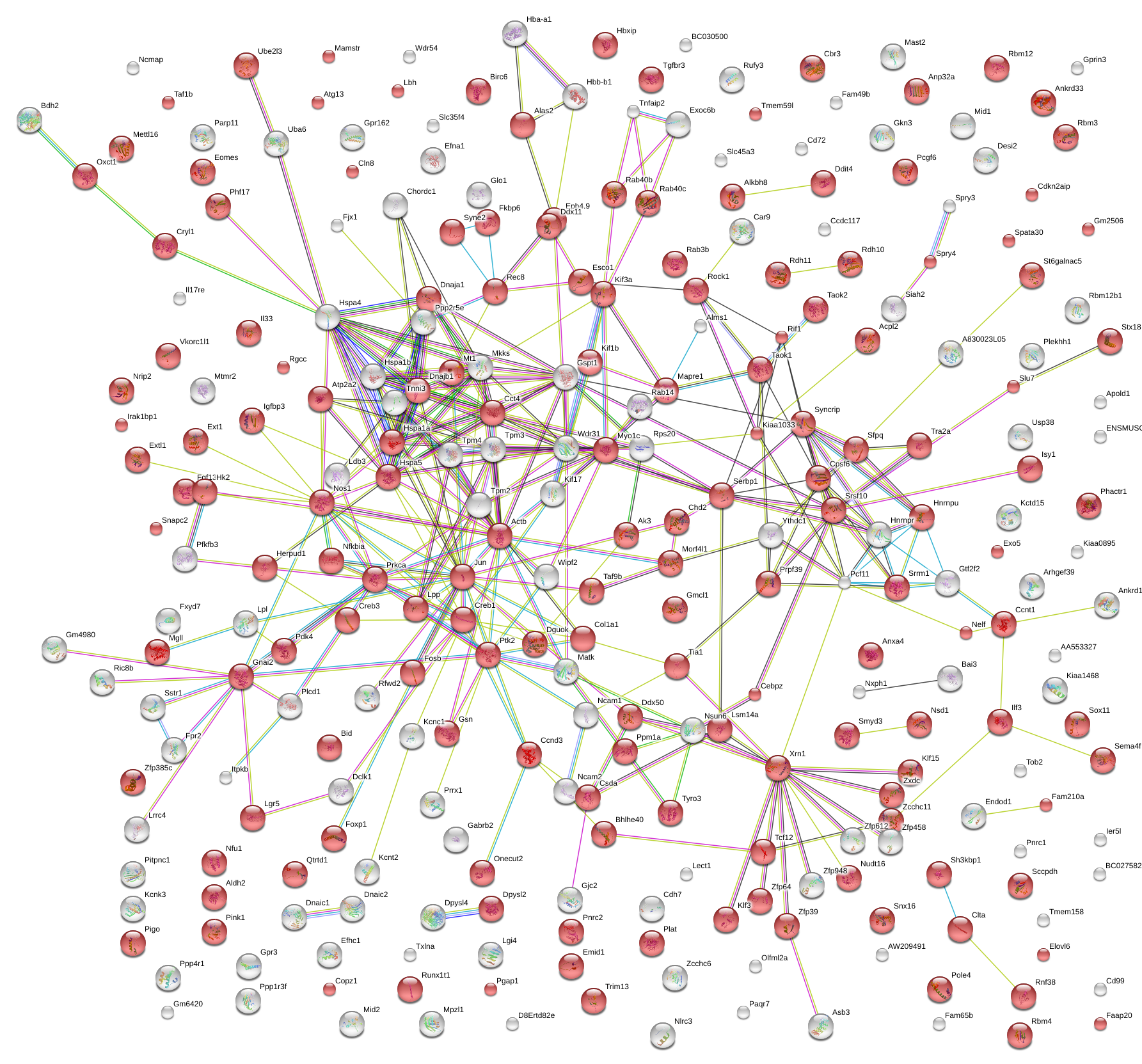

Supplement: Supplementary file 2 — Global transcriptome profile of Pink1-deficient mouse cerebellar tissue at ages 6 weeks (A), 6 months (B), and 18 months (C), illustrating the network with respect to interactions between the encoded proteins, employing the STRING web-server multiple proteins algorithm. (ZIP 11629 kb) [file 12974_2017_928_MOESM2_ESM.zip › TorresAuburger-SupplFigureS1A.tif]

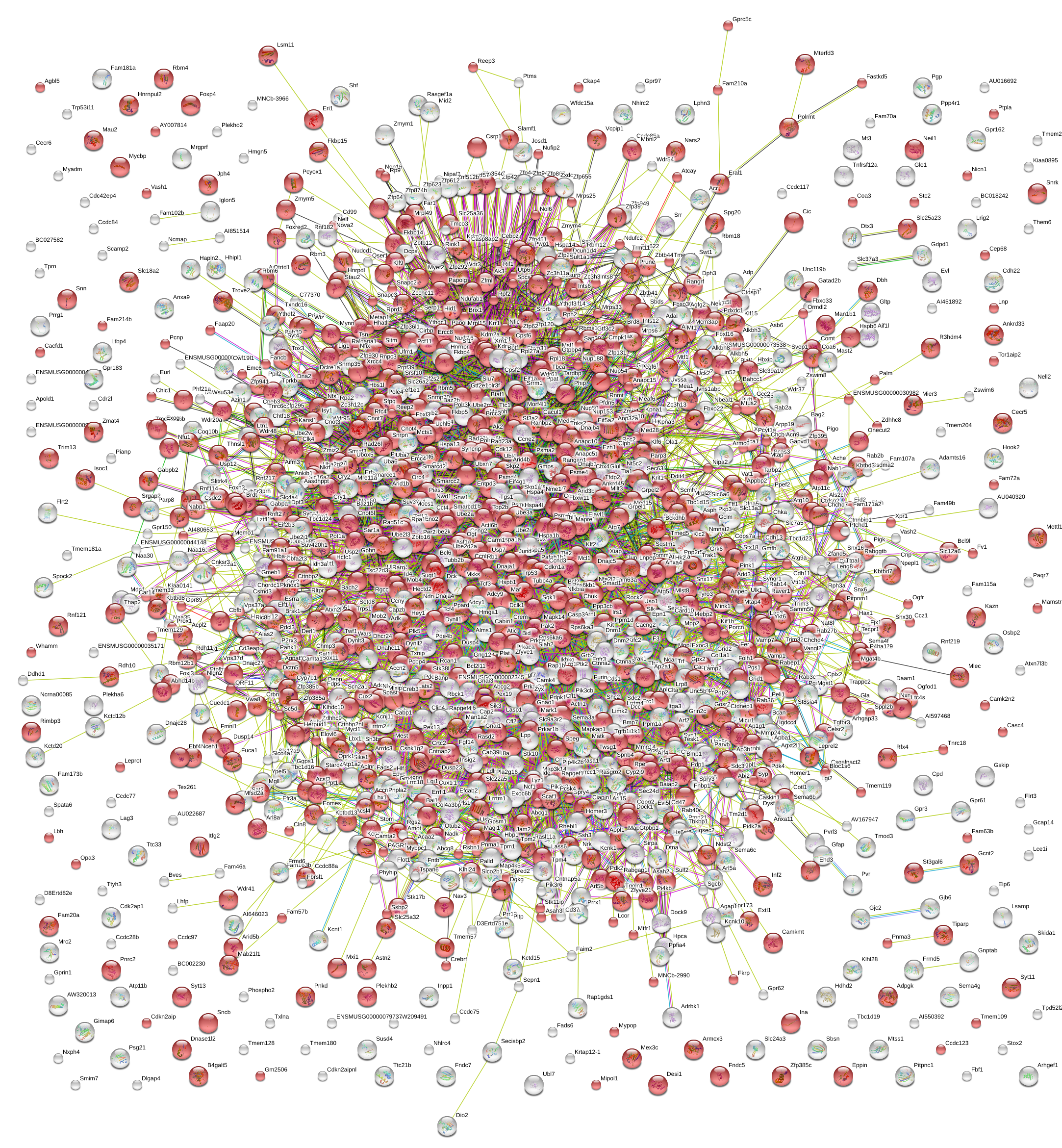

Supplement: Supplementary file 2 — Global transcriptome profile of Pink1-deficient mouse cerebellar tissue at ages 6 weeks (A), 6 months (B), and 18 months (C), illustrating the network with respect to interactions between the encoded proteins, employing the STRING web-server multiple proteins algorithm. (ZIP 11629 kb) [file 12974_2017_928_MOESM2_ESM.zip › TorresAuburger-SupplFigureS1B.tif]

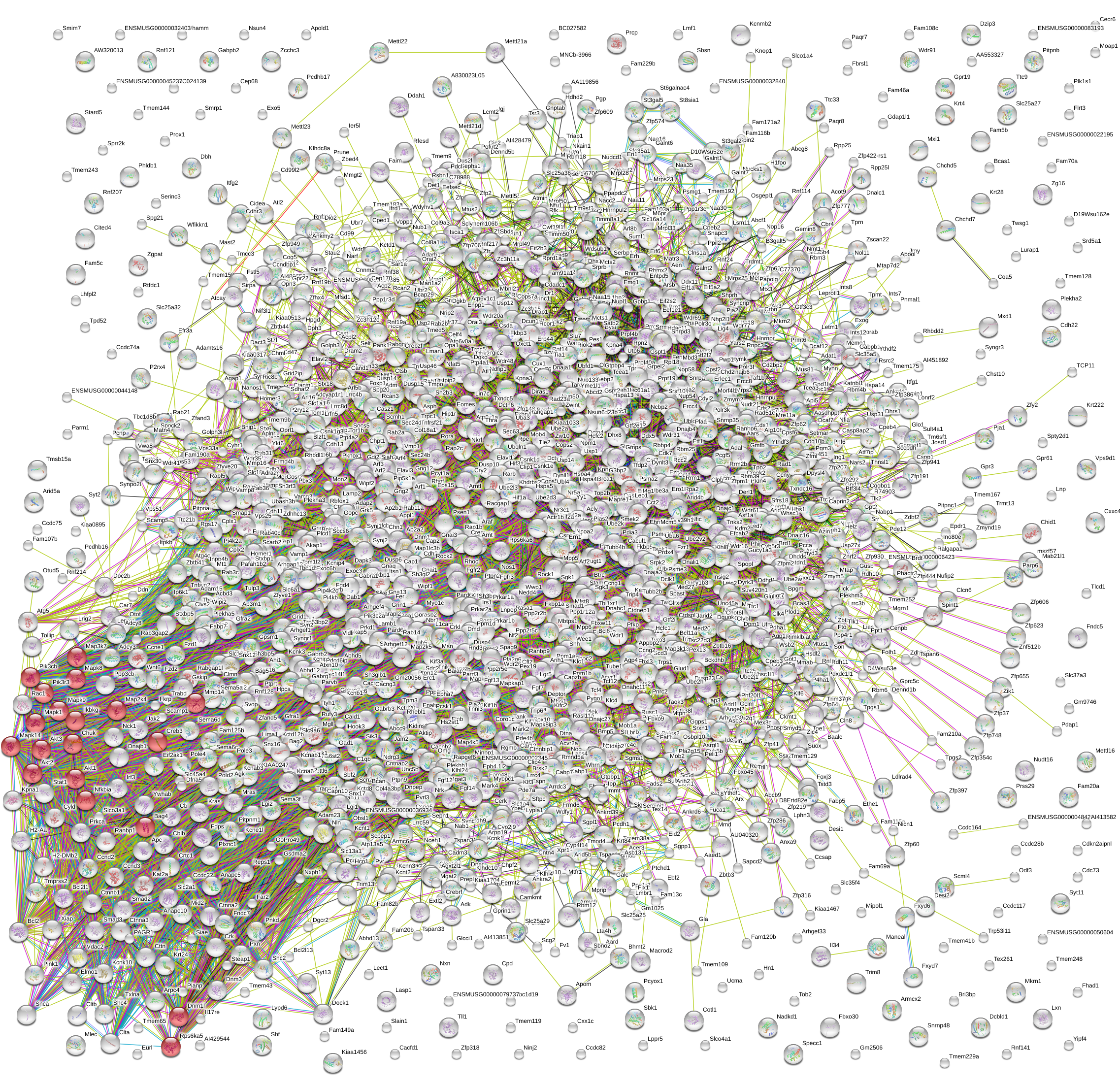

Supplement: Supplementary file 2 — Global transcriptome profile of Pink1-deficient mouse cerebellar tissue at ages 6 weeks (A), 6 months (B), and 18 months (C), illustrating the network with respect to interactions between the encoded proteins, employing the STRING web-server multiple proteins algorithm. (ZIP 11629 kb) [file 12974_2017_928_MOESM2_ESM.zip › TorresAuburger-SupplFigureS1C.tif]

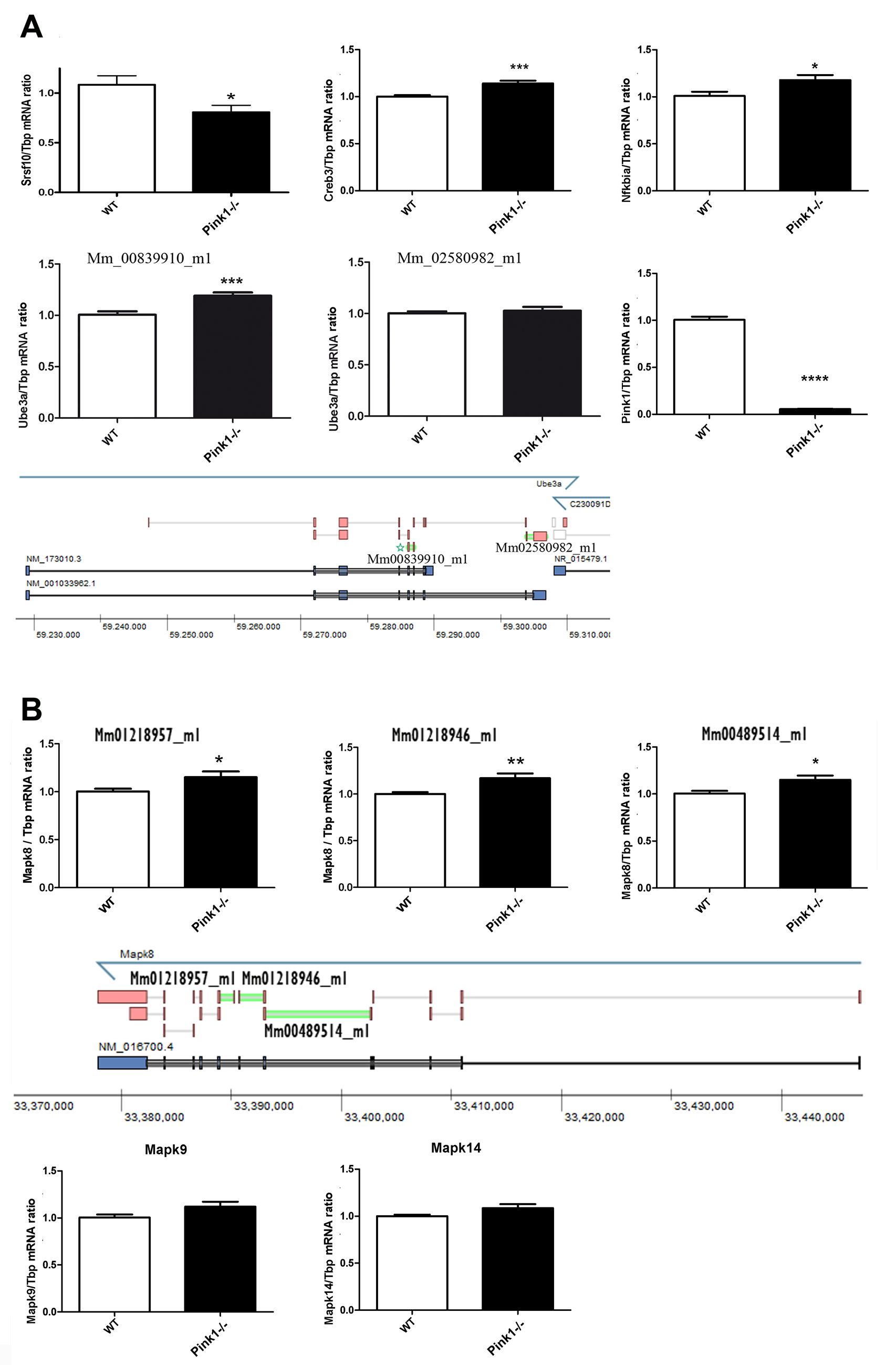

Supplement: Supplementary file 8 — Transcript changes of Pink1-deficient mouse cerebellar tissue at the age of 18 months in qPCR analyses represented in bar graphs. (A) Significant downregulation of Srsf10 mRNA and upregulations of Creb3 and Nfkbia mRNAs confirm the alteration within spliceosomal, ER stress and neuroinflammation pathways. The significant dysregulation of a Ube3a splice isoform is particularly interesting as a potential target of the spliceosome alterations and in view of its role in the degradation of alpha-synuclein. The scheme of Ube3a exon intron structure with the location of different Taqman assays was adapted from the Thermo Fisher Scientific internet site. (B) Significant upregulations of Mapk8 mRNA at 3 different exon junctions, together with a scheme of the Mapk8 exon intron structure and the location of 3 different Taqman assays (modified from the Thermo Fisher Scientific internet site). Non-significant changes of the MAPK phosphorylation cascade components Mapk9 and Mapk14 mRNAs demonstrate the selectivity of transcriptional regulation. Significant upregulations in the downstream nuclear transcription regulators Creb3 and Nfkbia in the stress and inflammation response may reflect biological responses to the Mapk8 upregulation. The bar graphs show mean and standard error of the mean (10 Pink1 −/− versus 10 WT), illustrating the significance with asterisks (* p < 0.05 and ** p < 0.01). (TIFF 13007 kb) [file 12974_2017_928_MOESM8_ESM.tif]

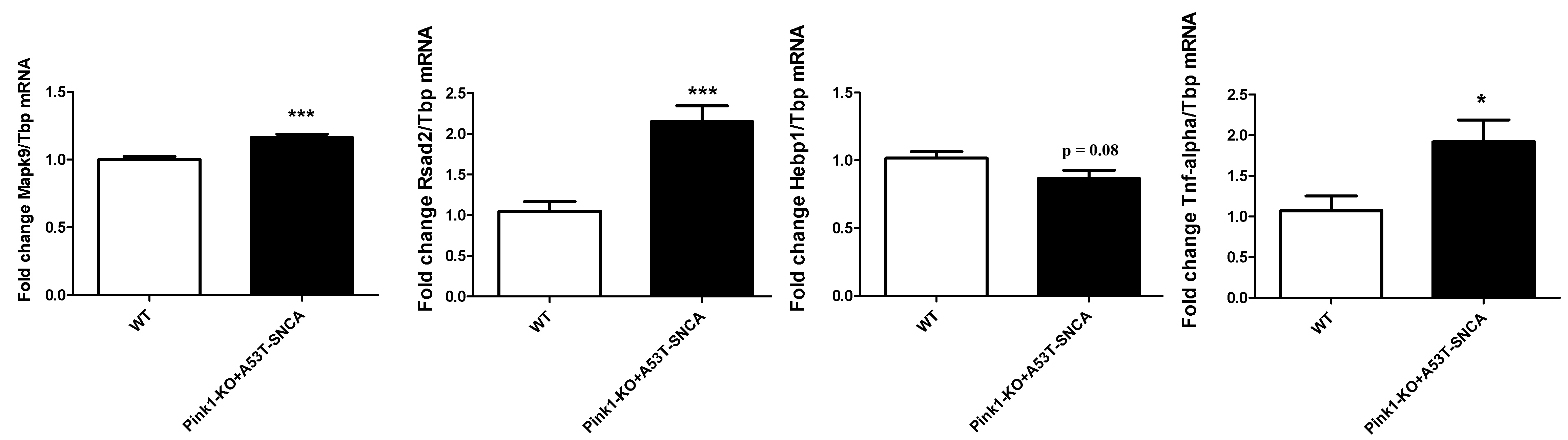

Supplement: Supplementary file 10 — Transcription dysregulation of innate immunity factors in the midbrain of 18-month-old Pink1-deficient mice with chronic stress from A53T-SNCA overexpression. The levels of the stress-triggered pro-inflammatory factor Mapk9, the lipid-droplet associated antiviral effector Rsad2 (viperin), the formylpeptide receptor activating Hebp1, and the immunostimulant Tnf (TNF-alpha) transcripts were assessed in brain from adult double mutant Pink1 −/−+A53T-SNCA mice. These data corroborate the brain tissue dysregulation of two factors that were previously found dysregulated in the global transcriptome profile of Pink1 −/− primary neuron cultures, taken from postnatal mice and maintained in vitro over 12 days. Data are displayed as bar graphs, illustrating mean values and standard error of the mean (SEM). Tbp transcript levels were used as loading controls to normalize the data. Significant differences were highlighted with asterisks (*p < 0.05; **p < 0.01; ***p < 0.001). (TIFF 17632 kb) [file 12974_2017_928_MOESM10_ESM.tif]

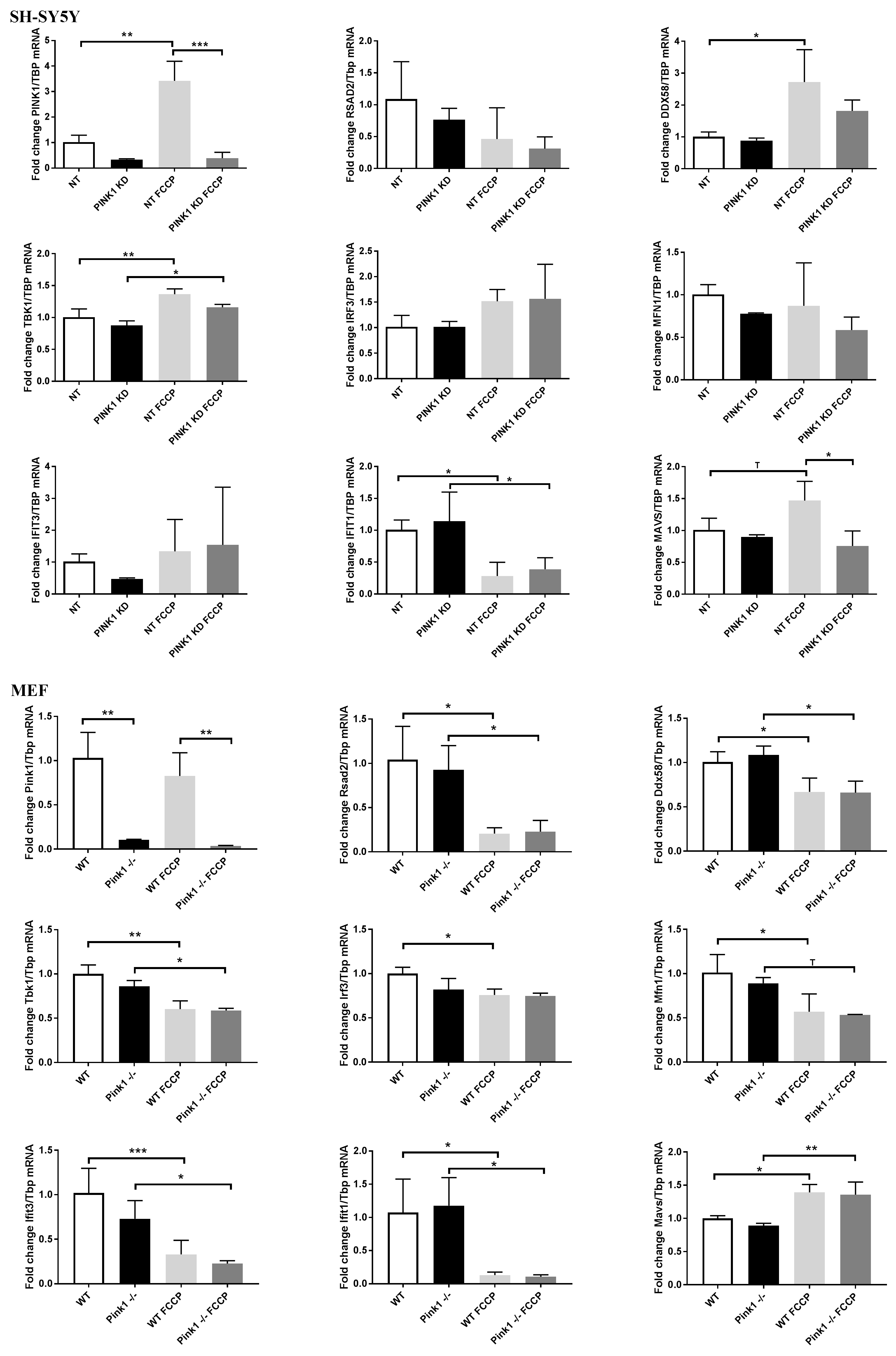

Supplement: Supplementary file 11 — Transcriptional response of innate immunity factors to 24 h treatment with uncoupling drug FCCP and subsequent mitophagy, in dependence on PINK1. Three independent experiments in SH-SY5Y human neuroblastoma (above) and murine embryonal fibroblast cells (below) documented the expression of key inflammatory factors in untreated versus drug-treated cells, comparing control with PINK1-deficiency. The bar graphs show mean and standard error of the mean, illustrating the significance with asterisks (* p < 0.05, ** p < 0.01, *** p < 0.001). (TIFF 538 kb) [file 12974_2017_928_MOESM11_ESM.tif]
