# Supplementary material for: Progression of pathology in PINK1-deficient mouse brain from splicing via ubiquitination, ER stress, and mitophagy changes to neuroinflammation
Source: J Neuroinflammation. 2017 Aug 2;14:154. doi: 10.1186/s12974-017-0928-0 (PMC5541666; doi:10.1186/s12974-017-0928-0)
Supplement: Supplementary file 4 — STRING bioinformatics assessment with high stringency of only >1.5-fold downregulations with significance. Ranked KEGG pathway dysregulations are shown for cerebellum at each age. The prominent MAPK signaling is highlighted in purple color, neuroinflammation pathways in red, ubiquitination pathways in blue, endoplasmic reticulum stress response pathways in orange, the mTOR signaling pathway (mimicked by starvation stress during in vitro experiments) in dark gray, the vesicular pathways in light brown. Subsequently, the interaction diagrams are shown for each age, with components of the prominent MAPK signaling pathway highlighted in red color. Finally, a heatmap shows all components of the MAPK signaling pathway with their expression changes at each age in cerebellum, with clearly progressive downregulation for several factors at the far end. (PDF 643 kb) [file 12974_2017_928_MOESM4_ESM.pdf]

STRING analysis in cerebellum of Pink1-/- mice for KEGG pathways >1.5-fold downregulated factors, confidence 0.9, p<0.05

Age 6 weeks

Term:	p-value	n
Estrogen signaling pathway	1,41E-04	5
MAPK signaling pathway	1,66E-02	5
Cocaine addiction	2,03E-02	3
Legionellosis	3,39E-02	3
Amphetamine addiction	4,36E-02	3

Age 6 months

Term:	p-value	n
MAPK signaling pathway	1,46E-03	13
Estrogen signaling pathway	2,98E-03	8
Dopaminergic synapse	3,08E-03	9
Cocaine addiction	4,29E-03	6
FoxO signaling pathway	5,41E-03	9
TNF signaling pathway	8,24E-03	8
Protein processing in endoplasmic reticulum	2,92E-02	9

Age 18 months

Term:	p-value	n
MAPK signaling pathway	7,73E-06	36
SNARE interaction in vesicular transport	1,31E-04	11
Thyroid hormone signaling pathway	1,85E-04	21
Retrograde endocannabinoid signaling	1,30E-03	18
Ras signaling pathway	3,29E-03	28
Glycerophospholipid metabolism	3,94E-03	16
Proteoglycans in cancer	7,65E-03	27
Neurotrophin signaling pathway	9,14E-03	18
Rap1 signaling pathway	9,16E-03	26
Endocytosis	9,95E-03	26
Protein processing in endoplasmic reticulum	1,27E-02	22
Ubiquitin mediated proteolysis	1,96E-02	19
Dopaminergic synapse	2,01E-02	18
Circadian entrainment	2,34E-02	15
Renal cell carcinoma	2,69E-02	12
Platelet activation	3,38E-02	18
Phosphatidylinositol signaling system	4,89E-02	13

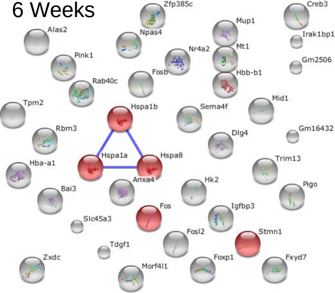
6 Weeks
Zfp385c
Creb3
Alas2
Npas4
Mup1
Irak1bp1
Nr4a2
Mt1
Pink1
Gm2506
Hbb-b1
Fosb
Rab40c
Mid1
Tpm2
Hspa1b
Sema4f
Gm16432
Rbm3
Dlg4
Hspa1a
Hspa8
Trim13
Hba-a1
Anxa4
Hk2
Pigo
Bai3
Igfbp3
Fos
Slc45a3
Stmn1
Fosl2
Tdgf1
Zxdc
Foxp1
Fxyd7
Morf4l1

6 Months

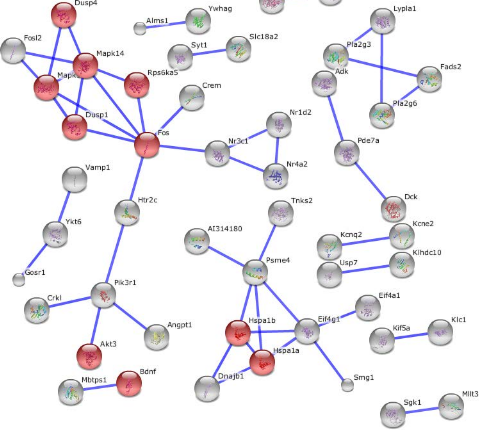
Dusp4
Mapk14
Mapk
Fosl2
Rps6ka5
Dusp1
Fos
Alms1
Ywhag
Syt1
Slc18a2
Crem
Cdk5r1
Sema3a
Lypla1
Pla2g3
Fads2
Adk
Pla2g6
Nr1d2
Nr3c1
Nr4a2
Pde7a
Dck
Vamp1
Ykt6
Gosr1
Htr2c
Pik3r1
Crkl
Angpt1
Akt3
Bdnf
Mbtps1
Tnks2
AI314180
Psme4
Kcnq2
Kcne2
Usp7
Klhdc10
Eif4a1
Eif4g1
Hspa1b
Hspa1a
Dnajb1
Smg1
Kif5a
Klc1
Sgk1
Mllt3

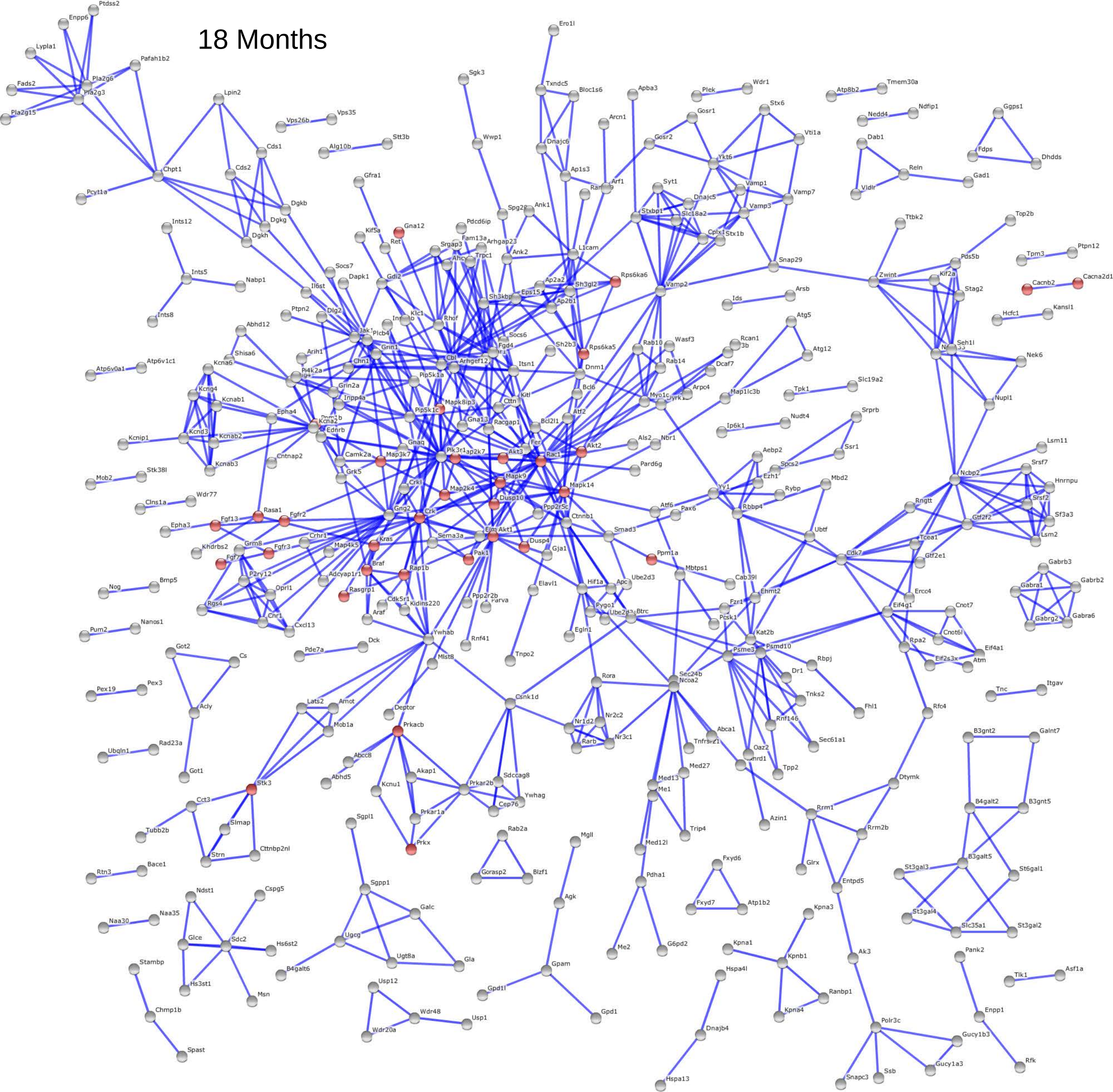

18 Months
Ptdss2
Enpp6
Lypla1
Pafah1b2
Fads2
Pla2g6
Pla2g3
Pla2g15
Lpin2
Chpt1
Pcyt1a
Cds1
Cds2
Dgkb
Dgkg
Dgkh
Vps26b
Vps35
Alg10b
Stt3b
Ero1l
Sgk3
Txndc5
Bloc1s6
Apba3
Plek
Wdr1
Atp8b2
Tmem30a
Nedd4
Ndfip1
Ggps1
Wwp1
Dnajc6
Arcn1
Gosr1
Stx6
Vti1a
Gosr2
Ykt6
Dab1
Reln
Vldlr
Fdps
Dhdds
Gad1
Ap1s3
Arf1
Syt1
Vamp1
Vamp7
Dnajc5
Vamp3
Stxbp1
Slc18a2
Cplx1
Stx1b
Snap29
Ints12
Ints5
Nabp1
Ints8
Gfra1
Kif5a
Gna12
Ret
Pdcd6ip
Fam13a
Srgap3
Arhgap23
Ank1
Ank2
Spg20
Trpc1
L1cam
Rps6ka6
Ap2a2
Sh3gl2
Ap2b1
Eps15
Sh3kbp1
Vamp2
Ttbk2
Top2b
Pds5b
Tpm3
Ptpn12
Zwint
Kif2a
Cacnb2
Cacna2d1
Stag2
Arsb
Ids
Hcfc1
Kansl1
Socs7
Dapk1
Gdi2
Il6st
Ptpn2
Dlg2
Klc1
Rhof
Jak1
Plcb4
Socs6
Fgd4
Sh2b3
Rps6ka5
Dnm1
Abhd12
Shisa6
Grin1
Arih1
Chn1
Cbl
Arhgef12
Itsn1
Atp6v0a1
Atp6v1c1
Kcna6
Pi4k2a
Grin2a
Pip5k1a
Bcl6
Kitl
Kcng4
Kcnab1
Inpp4a
Pip5k1c
Mapk8ip3
Cttn
Atf2
Epha4
Kcna2
Ednrb
Gna13
Racgap1
Bcl2l1
Kcnd3
Kcnab2
Kcnip1
Kcnab3
Cntnap2
Camk2a
Map3k7
Gnaq
Pik3r1
Akt3
Rac1
Akt2
Mob2
Stk38l
Grk5
Crkl
Mapk9
Map2k4
Dusp10
Mapk14
Ppp2r5c
Clns1a
Wdr77
Epha3
Fgf13
Rasa1
Fgfr2
Gng2
Crk
Elk1
Akt1
Ctnnb1
Khdrbs2
Grm8
Fgf7
Fgfr3
Crhr1
Map4k5
Kras
Sema3a
Pak1
Dusp4
Gja1
Smad3
Ppm1a
Braf
Rap1b
Adcyap1r1
P2ry12
Nog
Bmp5
Opri1
Rasgrp1
Rgs4
Cdk5r1
Kidins220
Ppp2r2b
Parva
Elavl1
Hif1a
Apc
Ube2d3
Cnr1
Cxcl13
Araf
Pygo1
Btrc
Egln1
Pum2
Nanos1
Ywhab
Rnf41
Got2
Cs
Pde7a
Dck
Mlst8
Tnpo2
Acly
Pex19
Pex3
Lats2
Amot
Mob1a
Deptor
Csnk1d
Rora
Ubqln1
Rad23a
Got1
Prkacb
Abcc8
Abhd5
Akap1
Kcnu1
Prkar2b
Sdccag8
Ywhag
Cep76
Nr1d2
Nr2c2
Rarb
Nr3c1
Stk3
Cct3
Slmap
Tubb2b
Strn
Cttnbp2nl
Prkar1a
Prkx
Sgpl1
Rab2a
Gorasp2
Blzf1
Mgll
Rtn3
Bace1
Sgpp1
Galc
Ugcg
Ugt8a
Gla
B4galt6
Agk
Gpam
Gpd1l
Gpd1
Naa30
Naa35
Ndst1
Cspg5
Glce
Sdc2
Hs6st2
Hs3st1
Msn
Stambp
Chmp1b
Spast
Usp12
Wdr48
Usp1
Wdr20a
Wasf3
Rcan1
Rab10
Rab14
Dcaf7
Atg5
Atg12
Myo1c
Arpc4
Map1lc3b
Tpk1
Slc19a2
Seh1l
Nek6
Nupl1
Ip6k1
Nudt4
Srprb
Ssr1
Als2
Nbr1
Pard6g
Aebp2
Spcs2
Ezh1
Yy1
Rybp
Mbd2
Atf6
Pax6
Rbbp4
Ubtf
Lsm11
Srsf7
Ncbp2
Hnrnpu
Srsf2
Sf3a3
Lsm2
Rngtt
Gtf2f2
Tcea1
Gtf2e1
Cdk7
Mbtps1
Cab39l
Ehmt2
Gabrb3
Gabra1
Gabrb2
Gabrg2
Gabra6
Ercc4
Eif4g1
Cnot7
Cnot6l
Fzr1
Pcsk1
Kat2b
Psme3
Psmd10
Rpa2
Eif4a1
Eif2s3x
Atm
Rbpj
Dr1
Sec24b
Ncoa2
Tnks2
Fhl1
Tnc
Itgav
Rnf146
Abca1
Tnfrsf21
Oaz2
Sec61a1
Tpp2
Rfc4
B3gnt2
Galnt7
Med27
Med13
Me1
Trip4
Azin1
Dtymk
Rrm1
Rrm2b
B4galt2
B3gnt5
Med12l
Glrx
Fxyd6
Pdha1
Fxyd7
Atp1b2
Entpd5
St3gal3
B3galt5
St6gal1
St3gal4
Slc35a1
St3gal2
Kpna3
Me2
G6pd2
Kpna1
Kpnb1
Ak3
Pank2
Hspa4l
Ranbp1
Kpna4
Tlk1
Asf1a
Enpp1
Dnajb4
Polr3c
Gucy1b3
Gucy1a3
Rfk
Snapc3
Ssb
Hspa13

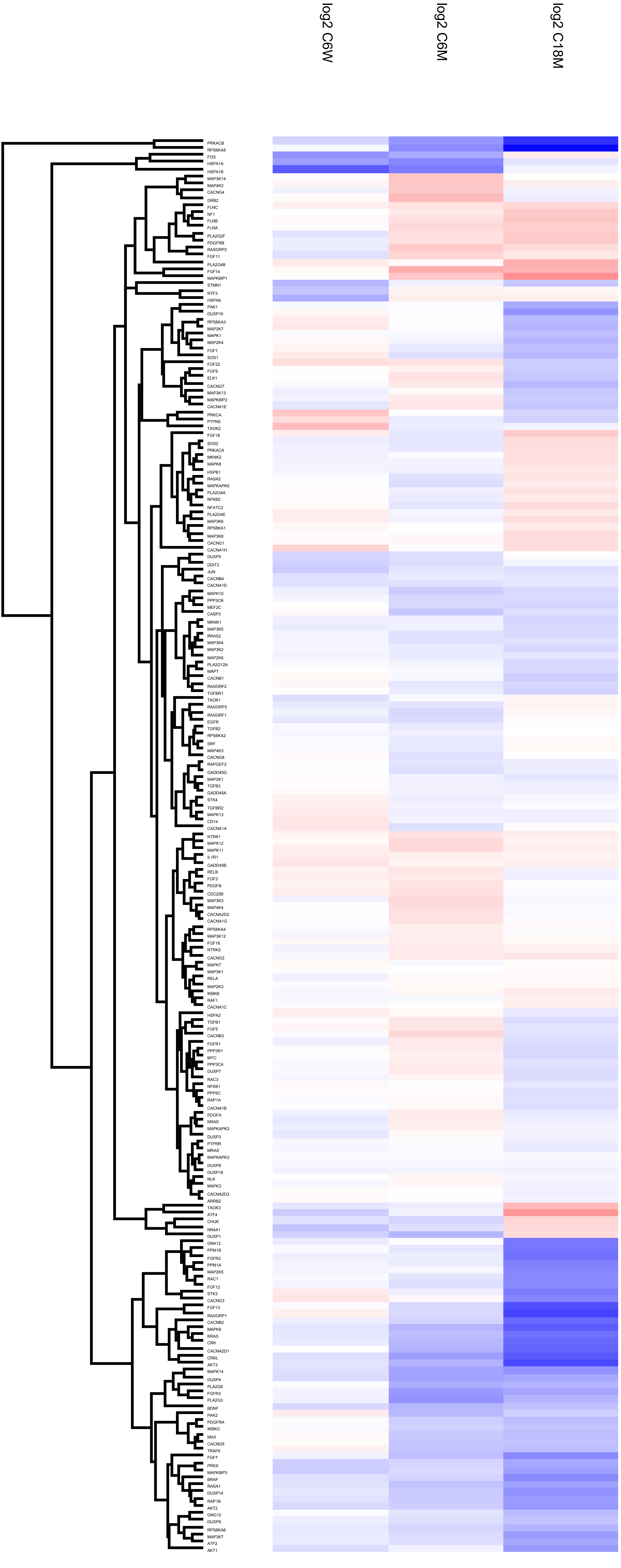
log2 C6W
log2 C6M
log2 C18M
PRKACB
RPS6KA5
FOS
HSPA1A
HSPA1B
MAP3K14
MAP4K2
CACNG4
GRB2
FLNC
NF1
FLNB
FLNA
PLA2G2F
PDGFRB
RASGRP2
FGF11
PLA2G4B
FGF14
MAPK8IP1
STMN1
NTF3
HSPA8
PAK1
DUSP10
RPS6KA3
MAP2K7
MAPK1
MAP2K4
FGF1
SOS1
FGF22
FGF9
ELK1
CACNG7
MAP3K13
MAPK8IP2
CACNA1E
PRKCA
PTPN5
TAOK2
FGF18
SOS2
PRKACA
MKNK2
MAPK8
HSPB1
RASA2
MAPKAPK5
PLA2G4A
NFKB2
NFATC2
PLA2G4E
MAP3K6
RPS6KA1
MAP3K8
CACNG1
CACNA1H
DUSP5
DDIT3
JUN
CACNB4
CACNA1D
MAPK10
PPP3CB
MEF2C
CASP3
MKNK1
MAP3K5
RRAS2
MAP3K4
MAP3K2
MAP2K6
PLA2G12A
MAPT
CACNB1
RASGRF2
TGFBR1
TAOK1
RASGRP3
RASGRF1
EGFR
TGFB2
RPS6KA2
SRF
MAP4K3
CACNG8
RAPGEF2
GADD45G
MAP2K1
TGFB3
GADD45A
STK4
TGFBR2
MAPK13
CD14
CACNA1A
NTRK1
MAPK12
MAPK11
IL1R1
GADD45B
RELB
FGF3
PDGFB
CDC25B
MAP3K3
MAP4K4
CACNA2D2
CACNA1G
RPS6KA4
MAP3K12
FGF16
NTRK2
CACNG2
MAPK7
MAP3K1
RELA
MAP2K3
IKBKB
RAF1
CACNA1C
HSPA2
TGFB1
FGF5
CACNB3
FGFR1
PPP3R1
MYC
PPP3CA
DUSP7
RAC3
NFKB1
PPP5C
RAP1A
CACNA1B
PDGFA
NRAS
MAPKAPK2
DUSP3
PTPRR
MRAS
MAPKAPK3
DUSP8
DUSP16
NLK
MAPK3
CACNA2D3
ARRB2
TAOK3
ATF4
CHUK
NR4A1
DUSP1
GNA12
PPM1B
FGFR2
PPM1A
MAP2K5
RAC1
FGF12
STK3
CACNG3
FGF13
RASGRP1
CACNB2
MAPK9
KRAS
CRK
CACNA2D1
CRKL
AKT3
MAPK14
DUSP4
PLA2G6
FGFR3
PLA2G3
BDNF
PAK2
PDGFRA
IKBKG
MAX
CACNG5
TRAF6
FGF7
PRKX
MAPK8IP3
BRAF
RASA1
DUSP14
RAP1B
AKT2
GNG12
DUSP6
RPS6KA6
MAP3K7
ATF2
AKT1
