# Supplementary material for: Progression of pathology in PINK1-deficient mouse brain from splicing via ubiquitination, ER stress, and mitophagy changes to neuroinflammation
Source: J Neuroinflammation. 2017 Aug 2;14:154. doi: 10.1186/s12974-017-0928-0 (PMC5541666; doi:10.1186/s12974-017-0928-0)
Supplement: Supplementary file 6 — GSEA bioinformatics assessment—top dozen pathways with components. In Pink1 −/− cerebellum at the age of 18 months the top dozen dysregulated KEGG pathways and the top dozen dysregulated Reactome pathways are shown with individual components. The nominal p values, false discovery rate q values and family wise error p values are shown on top, the Rank Metric Score indicates the log2 expression values for each factor, and YES reflects significance. (PDF 1540 kb) [file 12974_2017_928_MOESM6_ESM.pdf]

Table: GSEA Results Summary

Dataset	GSEA_18M_collapsed
Phenotype	NoPhenotypeAvailable
Upregulated in class	na_neg
GeneSet	KEGG_SNARE_INTERACTIONS_IN_VESICULAR_TRANSPORT
Enrichment Score (ES)	-0.66728276
Normalized Enrichment Score (NES)	-1.7324626
Nominal p-value	0.0
FDR q-value	0.06256143
FWER p-Value	0.063

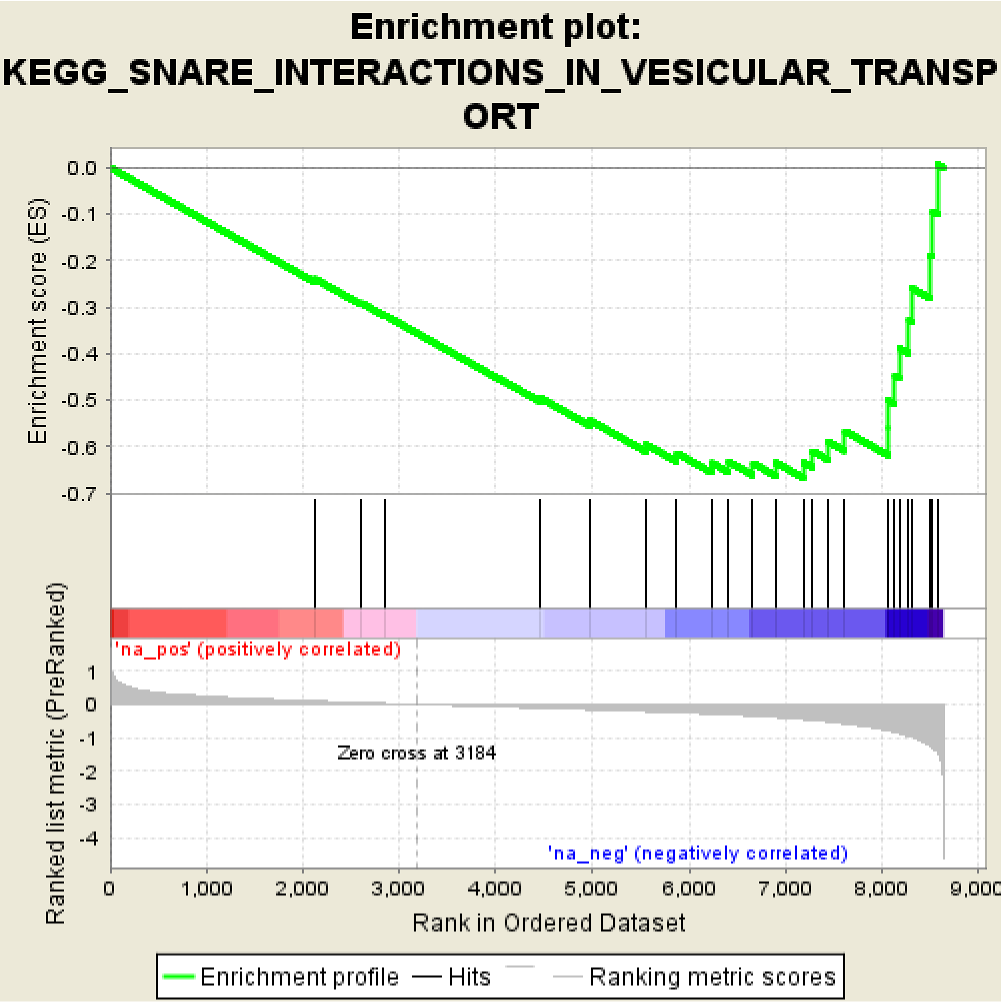

Fig 1: Enrichment plot: KEGG_SNARE_INTERACTIONS_IN_VESICULAR_TRANSPORT

Profile of the Running ES Score & Positions of GeneSet Members on the Rank Ordered List

Table: GSEA details [plain text format]

	PROBE	GENE SYMBOL	GENE_TITLE	RANK IN GENE LIST	RANK METRIC SCORE	RUNNING ES	CORE ENRICHMENT
1	VAMP5	VAMP5 Entrez, Source	vesicle-associated membrane protein 5 (myobrevin)	2125	0.101	-0.2392	No
2	STX12	STX12 Entrez, Source	syntaxin 12	2605	0.052	-0.2909	No
3	STX8	STX8 Entrez, Source	syntaxin 8	2860	0.028	-0.3183	No
4	STX1A	STX1A Entrez, Source	syntaxin 1A (brain)	4451	-0.109	-0.4948	No
5	VTI1B	VTI1B Entrez, Source	vesicle transport through interaction with t-SNAREs homolog 1B (yeast)	4971	-0.156	-0.5434	No
6	VAMP4	VAMP4 Entrez, Source	vesicle-associated membrane protein 4	5554	-0.210	-0.5953	No
7	SNAP23	SNAP23 Entrez, Source	synaptosomal-associated protein, 23kDa	5870	-0.243	-0.6138	No
8	SEC22B	SEC22B Entrez, Source	SEC22 vesicle trafficking protein homolog B (S. cerevisiae)	6233	-0.284	-0.6347	No
9	SNAP25	SNAP25 Entrez, Source	synaptosomal-associated protein, 25kDa	6408	-0.306	-0.6321	No
10	VAMP8	VAMP8 Entrez, Source	vesicle-associated membrane protein 8 (endobrevin)	6661	-0.346	-0.6356	Yes
11	BNIP1	BNIP1 Entrez, Source	BCL2/adenovirus E1B 19kDa interacting protein 1	6899	-0.388	-0.6342	Yes
12	STX3	STX3 Entrez, Source	syntaxin 3	7185	-0.449	-0.6339	Yes
13	STX17	STX17 Entrez, Source	syntaxin 17	7278	-0.474	-0.6092	Yes
14	BET1	BET1 Entrez, Source	BET1 homolog (S. cerevisiae)	7442	-0.521	-0.5893	Yes
15	STX18	STX18 Entrez, Source	syntaxin 18	7614	-0.576	-0.5663	Yes

16	GOSR1	GOSR1 Entrez, Source	golgi SNAP receptor complex member 1	8064	-0.790	-0.5595	Yes
17	STX6	STX6 Entrez, Source	syntaxin 6	8066	-0.791	-0.5007	Yes
18	GOSR2	GOSR2 Entrez, Source	golgi SNAP receptor complex member 2	8133	-0.827	-0.4468	Yes
19	SNAP29	SNAP29 Entrez, Source	synaptosomal-associated protein, 29kDa	8182	-0.861	-0.3882	Yes
20	YKT6	YKT6 Entrez, Source	YKT6 v-SNARE homolog (S. cerevisiae)	8279	-0.946	-0.3288	Yes
21	VTI1A	VTI1A Entrez, Source	vesicle transport through interaction with t-SNAREs homolog 1A (yeast)	8314	-0.982	-0.2597	Yes
22	VAMP3	VAMP3 Entrez, Source	vesicle-associated membrane protein 3 (cellubrevin)	8501	-1.238	-0.1891	Yes
23	VAMP2	VAMP2 Entrez, Source	vesicle-associated membrane protein 2 (synaptobrevin 2)	8526	-1.314	-0.0940	Yes
24	VAMP1	VAMP1 Entrez, Source	vesicle-associated membrane protein 1 (synaptobrevin 1)	8580	-1.433	0.0066	Yes

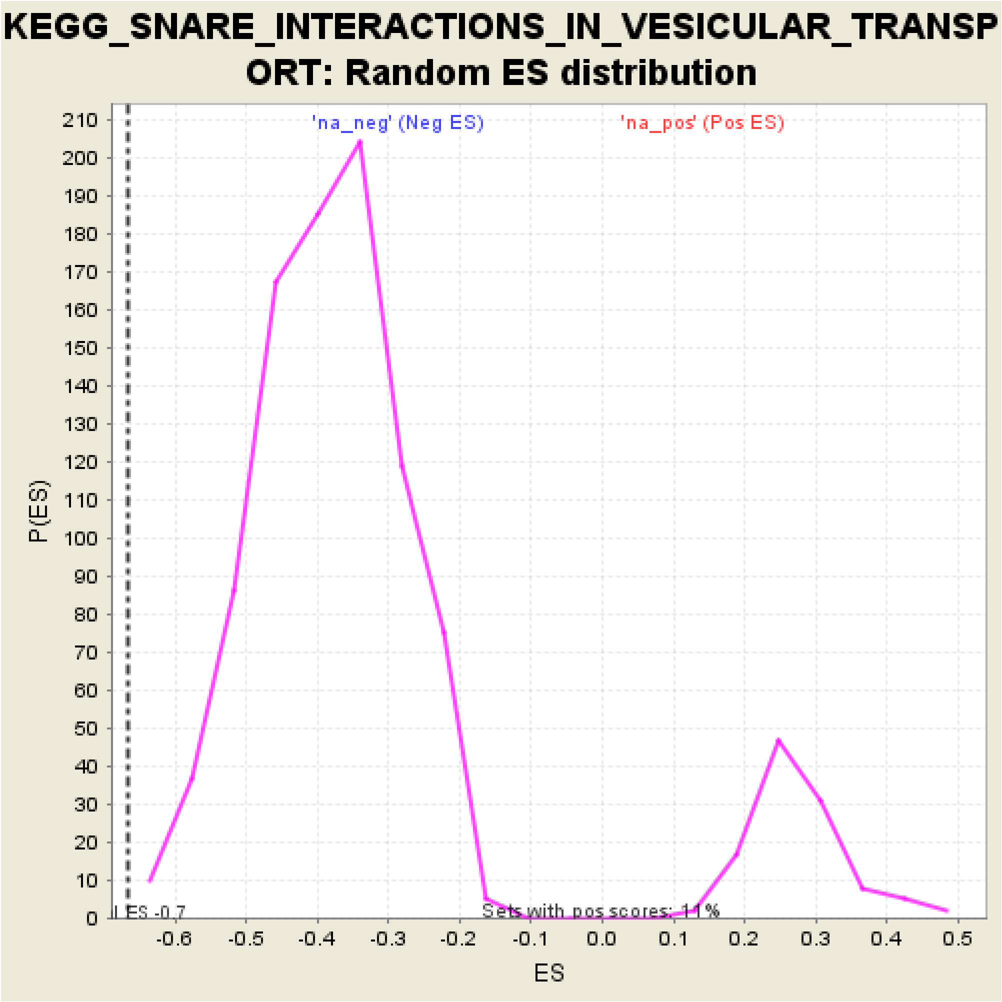

Fig 2: KEGG_SNARE_INTERACTIONS_IN_VESICULAR_TRANSPORT: Random ES distribution
Gene set null distribution of ES for KEGG_SNARE_INTERACTIONS_IN_VESICULAR_TRANSPORT

Table: GSEA Results Summary

Dataset	GSEA_18M_collapsed
Phenotype	NoPhenotypeAvailable
Upregulated in class	na_neg
GeneSet	KEGG_MTOR_SIGNALING_PATHWAY
Enrichment Score (ES)	-0.6273622
Normalized Enrichment Score (NES)	-1.7259375
Nominal p-value	0.0010660981
FDR q-value	0.035719242
FWER p-Value	0.072

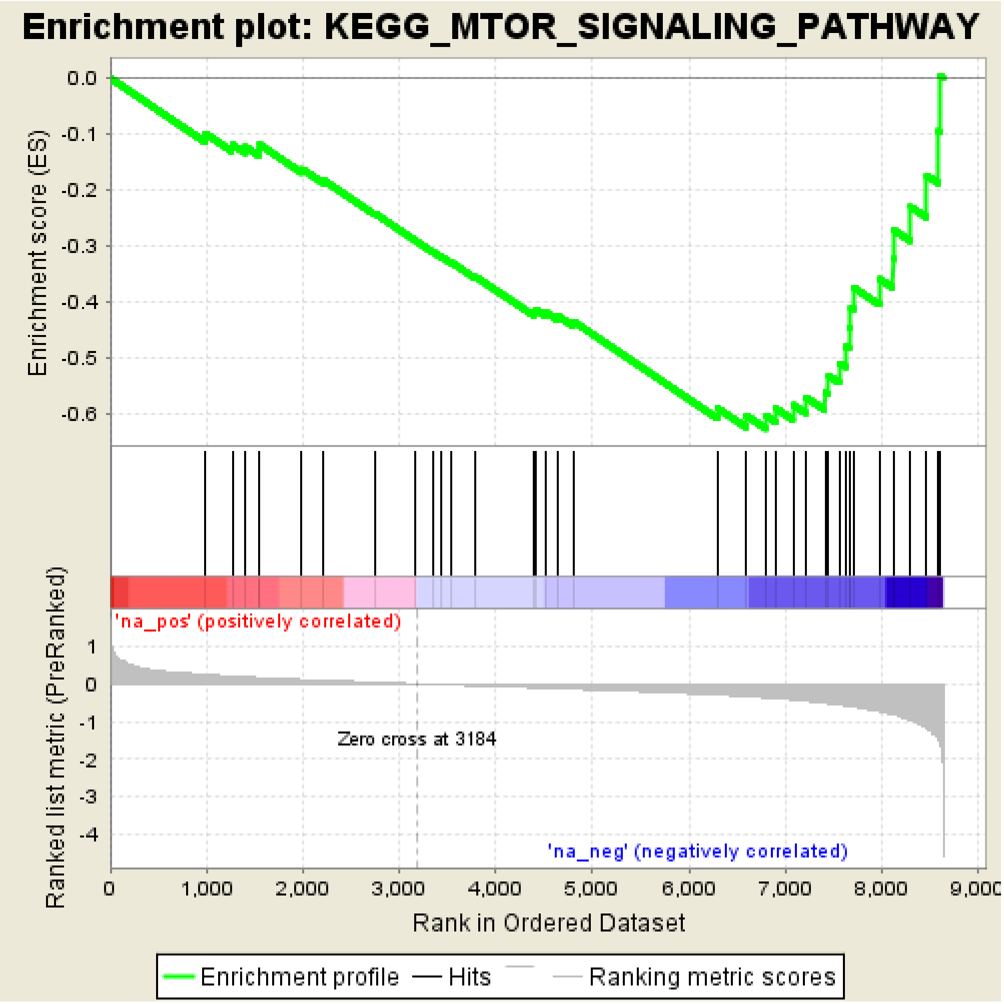

Fig 1: Enrichment plot: KEGG_MTOR_SIGNALING_PATHWAY Profile of the Running ES Score & Positions of GeneSet Members on the Rank Ordered List

Table: GSEA details [plain text format]

	PROBE	GENE	GENE_TITLE	RANK	RANK	RUNNING	CORE

		SYMBOL		IN GENE LIST	METRIC SCORE	ES	ENRICHMENT
1	FIGF	FIGF Entrez, Source	c-fos induced growth factor (vascular endothelial growth factor D)	984	0.246	-0.0988	No
2	PIK3CD	PIK3CD Entrez, Source	phosphoinositide-3-kinase, catalytic, delta polypeptide	1265	0.200	-0.1186	No
3	PIK3R5	PIK3R5 Entrez, Source	phosphoinositide-3-kinase, regulatory subunit 5, p101	1401	0.182	-0.1227	No
4	RPS6KA1	RPS6KA1 Entrez, Source	ribosomal protein S6 kinase, 90kDa, polypeptide 1	1540	0.165	-0.1283	No
5	RPS6KB2	RPS6KB2 Entrez, Source	ribosomal protein S6 kinase, 70kDa, polypeptide 2	1550	0.164	-0.1188	No
6	PIK3R2	PIK3R2 Entrez, Source	phosphoinositide-3-kinase, regulatory subunit 2 (p85 beta)	1989	0.114	-0.1625	No
7	PIK3CG	PIK3CG Entrez, Source	phosphoinositide-3-kinase, catalytic, gamma polypeptide	2211	0.092	-0.1824	No
8	PGF	PGF Entrez, Source	placental growth factor, vascular endothelial growth factor-related protein	2741	0.039	-0.2414	No
9	RPS6KA2	RPS6KA2 Entrez, Source	ribosomal protein S6 kinase, 90kDa, polypeptide 2	3164	0.002	-0.2904	No
10	PDPK1	PDPK1 Entrez, Source	3-phosphoinositide dependent protein kinase-1	3347	-0.015	-0.3106	No
11	ULK1	ULK1 Entrez, Source	unc-51-like kinase 1 (C. elegans)	3432	-0.022	-0.3190	No
12	DDIT4	DDIT4 Entrez, Source	DNA-damage-inducible transcript 4	3539	-0.030	-0.3294	No
13	RPS6	RPS6 Entrez, Source	ribosomal protein S6	3791	-0.052	-0.3553	No
14	PRKAA1	PRKAA1 Entrez, Source	protein kinase, AMP-activated, alpha 1 catalytic subunit	4396	-0.103	-0.4189	No
15	TSC1	TSC1 Entrez, Source	tuberous sclerosis 1	4410	-0.105	-0.4138	No
16	MAPK3	MAPK3 Entrez, Source	mitogen-activated protein kinase 3	4509	-0.114	-0.4179	No

17	PRKAA2	PRKAA2 Entrez, Source	protein kinase, AMP-activated, alpha 2 catalytic subunit	4632	-0.123	-0.4242	No
18	RPS6KB1	RPS6KB1 Entrez, Source	ribosomal protein S6 kinase, 70kDa, polypeptide 1	4796	-0.138	-0.4344	No
19	EIF4E2	EIF4E2 Entrez, Source	eukaryotic translation initiation factor 4E member 2	6298	-0.292	-0.5903	No
20	PIK3CB	PIK3CB Entrez, Source	phosphoinositide-3-kinase, catalytic, beta polypeptide	6592	-0.333	-0.6032	No
21	PIK3CA	PIK3CA Entrez, Source	phosphoinositide-3-kinase, catalytic, alpha polypeptide	6801	-0.370	-0.6038	Yes
22	EIF4E	EIF4E Entrez, Source	eukaryotic translation initiation factor 4E	6901	-0.388	-0.5906	Yes
23	CAB39	CAB39 Entrez, Source	calcium binding protein 39	7078	-0.423	-0.5841	Yes
24	PIK3R3	PIK3R3 Entrez, Source	phosphoinositide-3-kinase, regulatory subunit 3 (p55, gamma)	7209	-0.454	-0.5703	Yes
25	EIF4B	EIF4B Entrez, Source	eukaryotic translation initiation factor 4B	7409	-0.510	-0.5610	Yes
26	MAPK1	MAPK1 Entrez, Source	mitogen-activated protein kinase 1	7446	-0.522	-0.5319	Yes
27	RPS6KA3	RPS6KA3 Entrez, Source	ribosomal protein S6 kinase, 90kDa, polypeptide 3	7559	-0.558	-0.5093	Yes
28	IGF1	IGF1 Entrez, Source	insulin-like growth factor 1 (somatomedin C)	7626	-0.580	-0.4801	Yes
29	VEGFC	VEGFC Entrez, Source	vascular endothelial growth factor C	7663	-0.591	-0.4466	Yes
30	RPS6KA6	RPS6KA6 Entrez, Source	ribosomal protein S6 kinase, 90kDa, polypeptide 6	7676	-0.599	-0.4098	Yes
31	CAB39L	CAB39L Entrez, Source	calcium binding protein 39-like	7709	-0.612	-0.3746	Yes
32	ULK2	ULK2 Entrez, Source	unc-51-like kinase 2 (C. elegans)	7975	-0.729	-0.3589	Yes
33	AKT1	AKT1 Entrez, Source	v-akt murine thymoma viral oncogene homolog 1	8115	-0.817	-0.3231	Yes

34	AKT2	AKT2 Entrez, Source	v-akt murine thymoma viral oncogene homolog 2	8121	-0.821	-0.2713	Yes
35	BRAF	BRAF Entrez, Source	v-raf murine sarcoma viral oncogene homolog B1	8297	-0.967	-0.2301	Yes
36	PIK3R1	PIK3R1 Entrez, Source	phosphoinositide-3-kinase, regulatory subunit 1 (p85 alpha)	8463	-1.179	-0.1741	Yes
37	AKT3	AKT3 Entrez, Source	v-akt murine thymoma viral oncogene homolog 3 (protein kinase B, gamma)	8586	-1.473	-0.0945	Yes
38	HIF1A	HIF1A Entrez, Source	hypoxia-inducible factor 1, alpha subunit (basic helix-loop-helix transcription factor)	8605	-1.575	0.0037	Yes

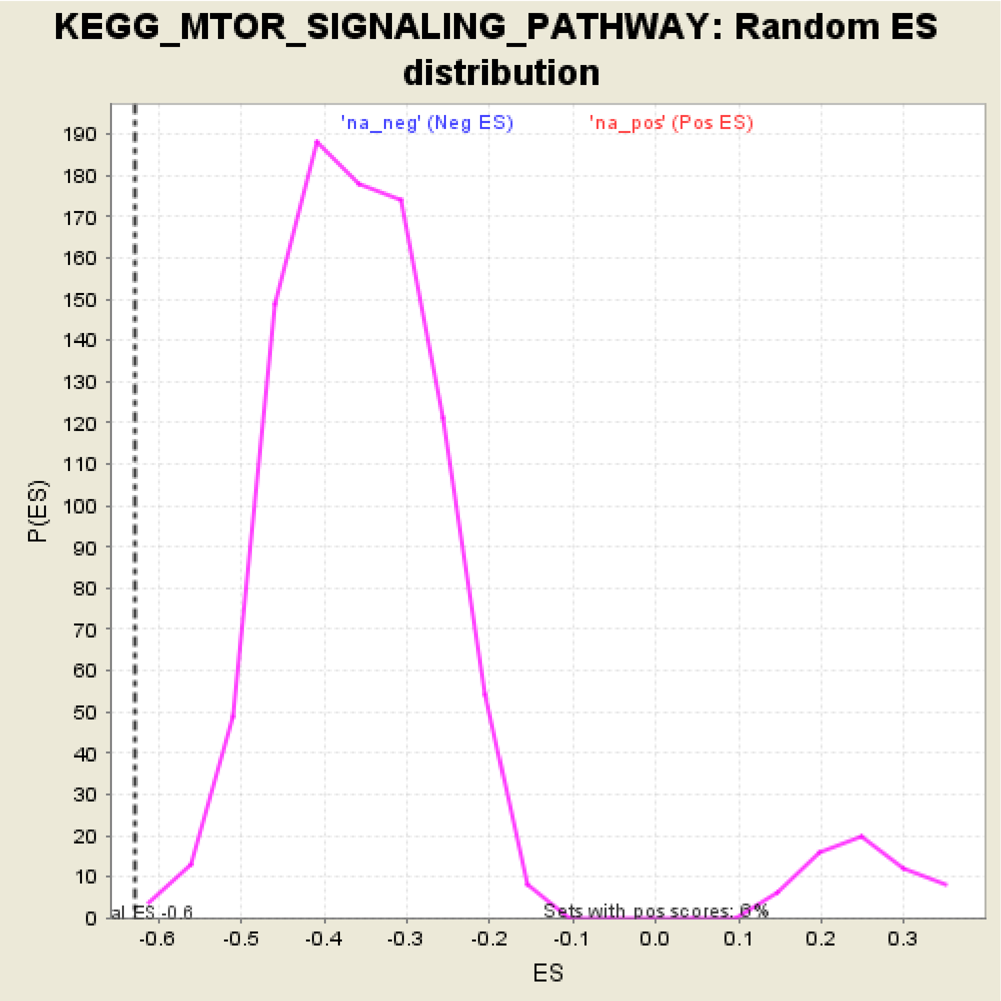

Fig 2: KEGG_MTOR_SIGNALING_PATHWAY: Random ES distribution
Gene set null distribution of ES for KEGG_MTOR_SIGNALING_PATHWAY

Table: GSEA Results Summary

Dataset	GSEA_18M_collapsed
Phenotype	NoPhenotypeAvailable
Upregulated in class	na_neg
GeneSet	KEGG_PROGESTERONE_MEDIATED_OOCYTE_MATURATION
Enrichment Score (ES)	-0.5790663
Normalized Enrichment Score (NES)	-1.7147609
Nominal p-value	0.0
FDR q-value	0.030667158
FWER p-Value	0.093

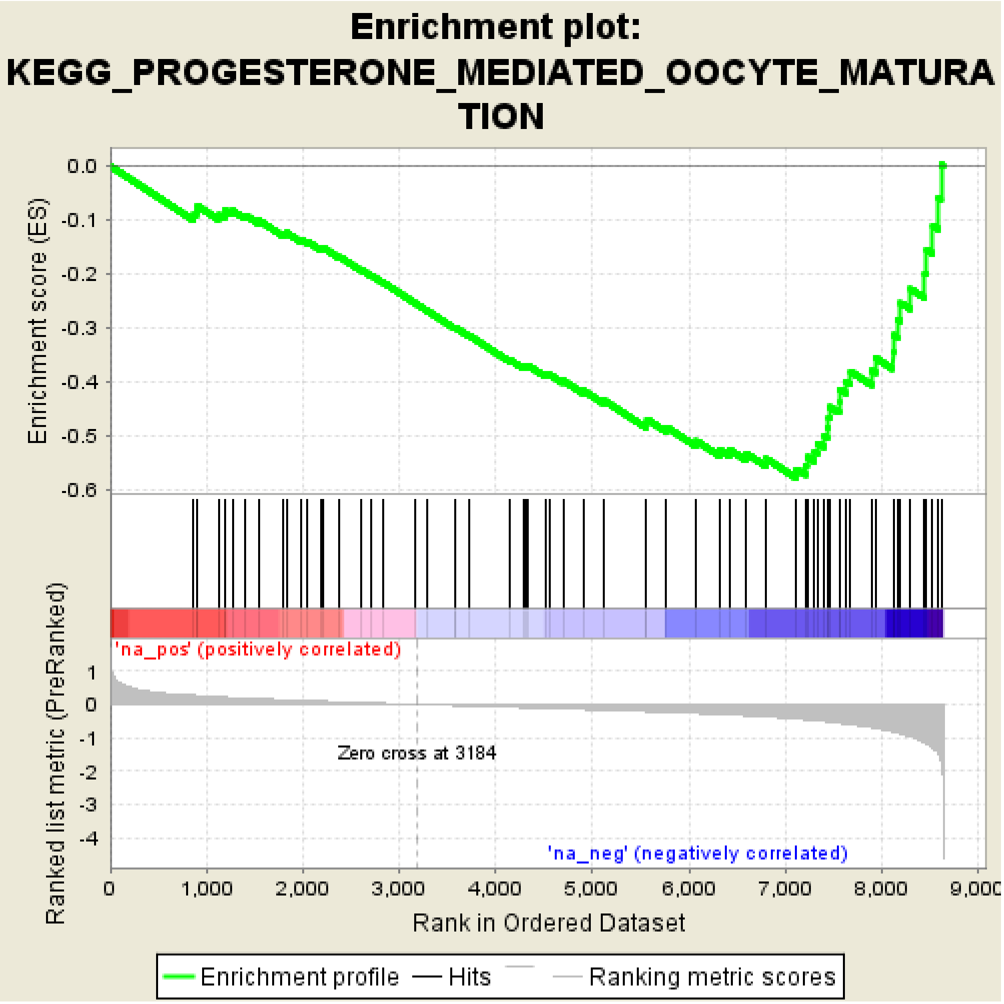

Fig 1: Enrichment plot: KEGG_PROGESTERONE_MEDIATED_OOCYTE_MATURATION

Profile of the Running ES Score & Positions of GeneSet Members on the Rank Ordered List

Table: GSEA details [plain text format]

	PROBE	GENE SYMBOL	GENE_TITLE	RANK IN GENE LIST	RANK METRIC SCORE	RUNNING ES	CORE ENRICHMENT
1	ADCY1	ADCY1 Entrez, Source	adenylate cyclase 1 (brain)	868	0.269	-0.0907	No
2	PRKACA	PRKACA Entrez, Source	protein kinase, cAMP-dependent, catalytic, alpha	893	0.264	-0.0831	No
3	MAPK8	MAPK8 Entrez, Source	mitogen-activated protein kinase 8	906	0.262	-0.0742	No
4	IGF1R	IGF1R Entrez, Source	insulin-like growth factor 1 receptor	1122	0.222	-0.0906	No
5	ADCY5	ADCY5 Entrez, Source	adenylate cyclase 5	1188	0.212	-0.0899	No
6	CCNA2	CCNA2 Entrez, Source	cyclin A2	1192	0.212	-0.0819	No
7	PIK3CD	PIK3CD Entrez, Source	phosphoinositide-3-kinase, catalytic, delta polypeptide	1265	0.200	-0.0825	No
8	PIK3R5	PIK3R5 Entrez, Source	phosphoinositide-3-kinase, regulatory subunit 5, p101	1401	0.182	-0.0911	No
9	RPS6KA1	RPS6KA1 Entrez, Source	ribosomal protein S6 kinase, 90kDa, polypeptide 1	1540	0.165	-0.1007	No
10	RAF1	RAF1 Entrez, Source	v-raf-1 murine leukemia viral oncogene homolog 1	1794	0.134	-0.1250	No
11	MAPK11	MAPK11 Entrez, Source	mitogen-activated protein kinase 11	1826	0.131	-0.1234	No
12	PIK3R2	PIK3R2 Entrez, Source	phosphoinositide-3-kinase, regulatory subunit 2 (p85 beta)	1989	0.114	-0.1379	No
13	ADCY6	ADCY6 Entrez, Source	adenylate cyclase 6	2045	0.108	-0.1400	No
14	MAPK12	MAPK12 Entrez, Source	mitogen-activated protein kinase 12	2182	0.095	-0.1522	No
15	PIK3CG	PIK3CG Entrez, Source	phosphoinositide-3-kinase, catalytic, gamma polypeptide	2211	0.092	-0.1519	No

16	ADCY7	ADCY7 Entrez, Source	adenylate cyclase 7	2380	0.075	-0.1685	No
17	ANAPC4	ANAPC4 Entrez, Source	anaphase promoting complex subunit 4	2608	0.052	-0.1929	No
18	ANAPC7	ANAPC7 Entrez, Source	anaphase promoting complex subunit 7	2717	0.041	-0.2039	No
19	PDE3B	PDE3B Entrez, Source	phosphodiesterase 3B, cGMP-inhibited	2834	0.030	-0.2163	No
20	RPS6KA2	RPS6KA2 Entrez, Source	ribosomal protein S6 kinase, 90kDa, polypeptide 2	3164	0.002	-0.2546	No
21	ANAPC2	ANAPC2 Entrez, Source	anaphase promoting complex subunit 2	3291	-0.010	-0.2689	No
22	CDC25B	CDC25B Entrez, Source	cell division cycle 25B	3573	-0.034	-0.3004	No
23	ADCY2	ADCY2 Entrez, Source	adenylate cyclase 2 (brain)	3580	-0.035	-0.2997	No
24	HSP90AA1	HSP90AA1 Entrez, Source	heat shock protein 90kDa alpha (cytosolic), class A member 1	3719	-0.047	-0.3140	No
25	CDC25A	CDC25A Entrez, Source	cell division cycle 25A	4147	-0.084	-0.3605	No
26	HSP90AB1	HSP90AB1 Entrez, Source	heat shock protein 90kDa alpha (cytosolic), class B member 1	4282	-0.094	-0.3724	No
27	SPDYA	SPDYA Entrez, Source	speedy homolog A (Drosophila)	4305	-0.096	-0.3712	No
28	CPEB1	CPEB1 Entrez, Source	cytoplasmic polyadenylation element binding protein 1	4332	-0.098	-0.3704	No
29	MAPK3	MAPK3 Entrez, Source	mitogen-activated protein kinase 3	4509	-0.114	-0.3865	No
30	CDC27	CDC27 Entrez, Source	cell division cycle 27	4553	-0.117	-0.3869	No
31	MAPK13	MAPK13 Entrez, Source	mitogen-activated protein kinase 13	4691	-0.129	-0.3978	No
32	CDC16	CDC16 Entrez, Source	CDC16 cell division cycle 16 homolog (S. cerevisiae)	4900	-0.149	-0.4162	No

33	MAD2L2	MAD2L2 Entrez, Source	MAD2 mitotic arrest deficient-like 2 (yeast)	5108	-0.167	-0.4338	No
34	MAP2K1	MAP2K1 Entrez, Source	mitogen-activated protein kinase kinase 1	5558	-0.211	-0.4779	No
35	CCNB2	CCNB2 Entrez, Source	cyclin B2	5560	-0.211	-0.4697	No
36	GNAI2	GNAI2 Entrez, Source	guanine nucleotide binding protein (G protein), alpha inhibiting activity polypeptide 2	5765	-0.232	-0.4844	No
37	PGR	PGR Entrez, Source	progesterone receptor	6075	-0.266	-0.5100	No
38	GNAI3	GNAI3 Entrez, Source	guanine nucleotide binding protein (G protein), alpha inhibiting activity polypeptide 3	6319	-0.295	-0.5268	No
39	MAPK10	MAPK10 Entrez, Source	mitogen-activated protein kinase 10	6420	-0.307	-0.5264	No
40	PIK3CB	PIK3CB Entrez, Source	phosphoinositide-3-kinase, catalytic, beta polypeptide	6592	-0.333	-0.5333	No
41	PIK3CA	PIK3CA Entrez, Source	phosphoinositide-3-kinase, catalytic, alpha polypeptide	6801	-0.370	-0.5430	No
42	CDC23	CDC23 Entrez, Source	CDC23 (cell division cycle 23, yeast, homolog)	7111	-0.429	-0.5622	Yes
43	PIK3R3	PIK3R3 Entrez, Source	phosphoinositide-3-kinase, regulatory subunit 3 (p55, gamma)	7209	-0.454	-0.5557	Yes
44	ADCY8	ADCY8 Entrez, Source	adenylate cyclase 8 (brain)	7224	-0.457	-0.5394	Yes
45	BUB1	BUB1 Entrez, Source	BUB1 budding uninhibited by benzimidazoles 1 homolog (yeast)	7300	-0.479	-0.5294	Yes
46	GNAI1	GNAI1 Entrez, Source	guanine nucleotide binding protein (G protein), alpha inhibiting activity polypeptide 1	7333	-0.488	-0.5140	Yes
47	ANAPC1	ANAPC1 Entrez, Source	anaphase promoting complex subunit 1	7403	-0.507	-0.5021	Yes
48	ANAPC5	ANAPC5 Entrez, Source	anaphase promoting complex subunit 5	7436	-0.519	-0.4855	Yes

49	MAPK1	MAPK1 Entrez, Source	mitogen-activated protein kinase 1	7446	-0.522	-0.4660	Yes
50	ADCY3	ADCY3 Entrez, Source	adenylate cyclase 3	7460	-0.528	-0.4468	Yes
51	RPS6KA3	RPS6KA3 Entrez, Source	ribosomal protein S6 kinase, 90kDa, polypeptide 3	7559	-0.558	-0.4363	Yes
52	ANAPC10	ANAPC10 Entrez, Source	anaphase promoting complex subunit 10	7564	-0.560	-0.4148	Yes
53	IGF1	IGF1 Entrez, Source	insulin-like growth factor 1 (somatomedin C)	7626	-0.580	-0.3992	Yes
54	RPS6KA6	RPS6KA6 Entrez, Source	ribosomal protein S6 kinase, 90kDa, polypeptide 6	7676	-0.599	-0.3814	Yes
55	FZR1	FZR1 Entrez, Source	fizzy/cell division cycle 20 related 1 (Drosophila)	7896	-0.694	-0.3797	Yes
56	PRKX	PRKX Entrez, Source	protein kinase, X-linked	7937	-0.713	-0.3563	Yes
57	AKT1	AKT1 Entrez, Source	v-akt murine thymoma viral oncogene homolog 1	8115	-0.817	-0.3449	Yes
58	AKT2	AKT2 Entrez, Source	v-akt murine thymoma viral oncogene homolog 2	8121	-0.821	-0.3133	Yes
59	ARAF	ARAF Entrez, Source	v-raf murine sarcoma 3611 viral oncogene homolog	8164	-0.845	-0.2850	Yes
60	MAPK14	MAPK14 Entrez, Source	mitogen-activated protein kinase 14	8194	-0.875	-0.2540	Yes
61	BRAF	BRAF Entrez, Source	v-raf murine sarcoma viral oncogene homolog B1	8297	-0.967	-0.2280	Yes
62	KRAS	KRAS Entrez, Source	v-Ki-ras2 Kirsten rat sarcoma viral oncogene homolog	8444	-1.153	-0.1997	Yes
63	PIK3R1	PIK3R1 Entrez, Source	phosphoinositide-3-kinase, regulatory subunit 1 (p85 alpha)	8463	-1.179	-0.1555	Yes
64	MAPK9	MAPK9 Entrez, Source	mitogen-activated protein kinase 9	8528	-1.320	-0.1112	Yes
65	AKT3	AKT3 Entrez, Source	v-akt murine thymoma viral oncogene homolog 3 (protein kinase B, gamma)	8586	-1.473	-0.0600	Yes

66	PRKACB	PRKACB Entrez, Source	protein kinase, cAMP-dependent, catalytic, beta	8614	-1.677	0.0027	Yes

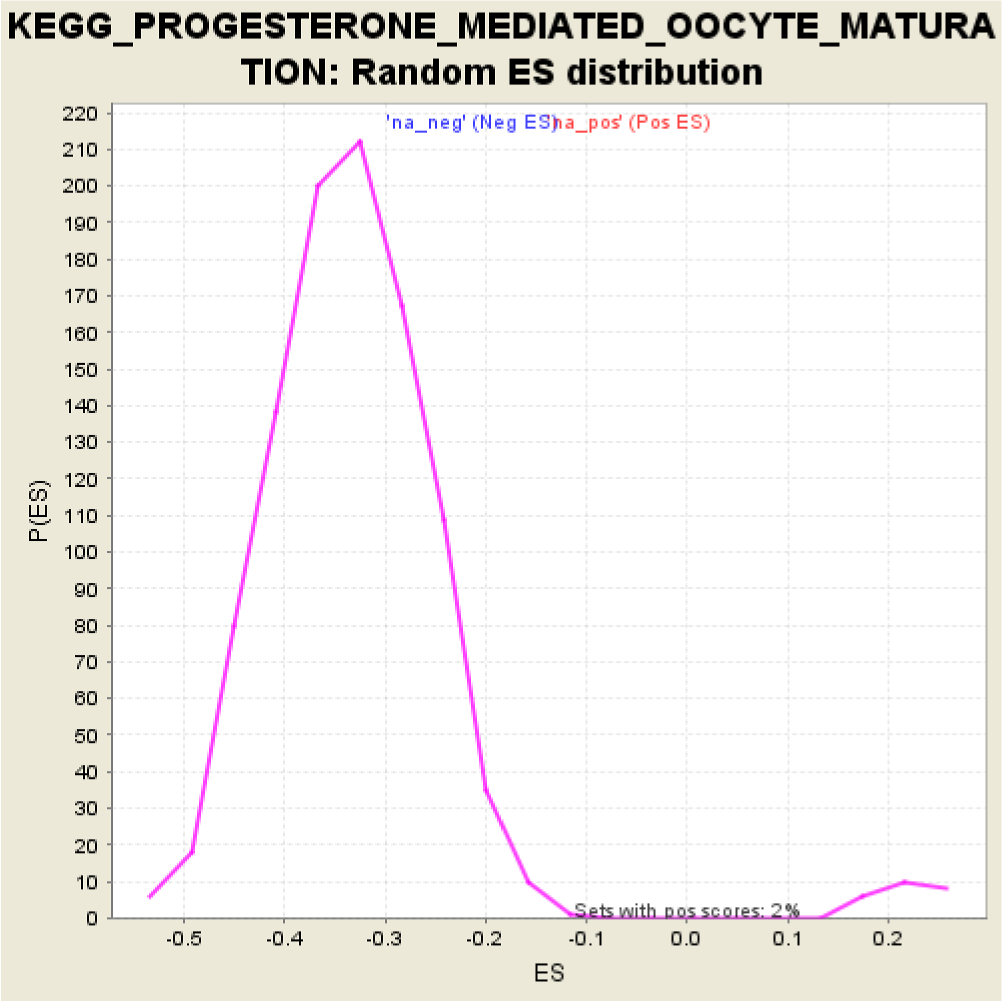

Fig 2: KEGG_PROGESTERONE_MEDIATED_OOCYTE_MATURATION: Random ES distribution
Gene set null distribution of ES for KEGG_PROGESTERONE_MEDIATED_OOCYTE_MATURATION

Table: GSEA Results Summary

Dataset	GSEA_18M_collapsed
Phenotype	NoPhenotypeAvailable
Upregulated in class	na_neg
GeneSet	KEGG_NOD_LIKE_RECEPTOR_SIGNALING_PATHWAY
Enrichment Score (ES)	-0.43181878
Normalized Enrichment Score (NES)	-1.1397108
Nominal p-value	0.29501083
FDR q-value	0.49746913
FWER p-Value	1.0

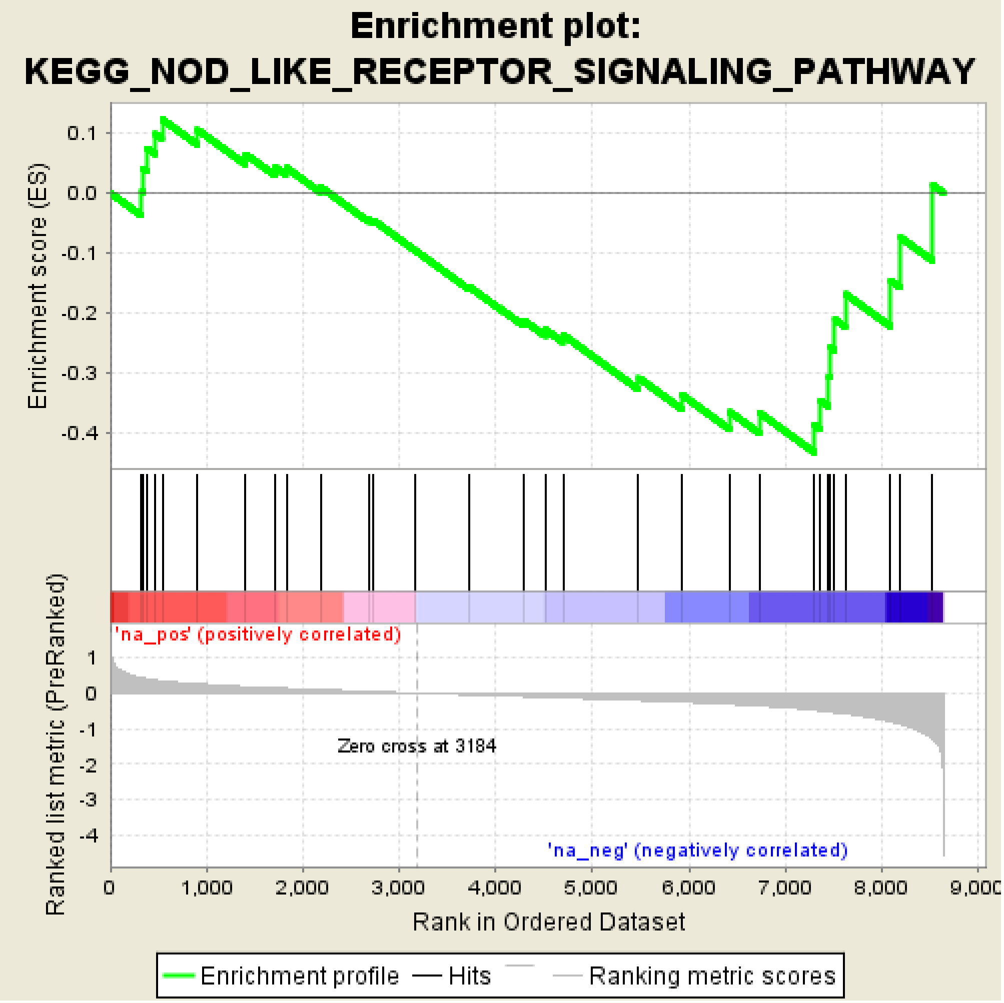

Fig 1: Enrichment plot: KEGG_NOD_LIKE_RECEPTOR_SIGNALING_PATHWAY Profile of the Running ES Score & Positions of GeneSet Members on the Rank Ordered List

Table: GSEA details [plain text format]

	PROBE	GENE	GENE_TITLE	RANK	RANK	RUNNING	CORE

		SYMBOL		IN GENE LIST	METRIC SCORE	ES	ENRICHMENT
1	NFKBIA	NFKBIA Entrez, Source	nuclear factor of kappa light polypeptide gene enhancer in B-cells inhibitor, alpha	330	0.423	0.0020	No
2	TRIP6	TRIP6 Entrez, Source	thyroid hormone receptor interactor 6	341	0.419	0.0408	No
3	PSTPIP1	PSTPIP1 Entrez, Source	proline-serine-threonine phosphatase interacting protein 1	382	0.399	0.0742	No
4	ERBB2IP	ERBB2IP Entrez, Source	erbb2 interacting protein	470	0.357	0.0981	No
5	CHUK	CHUK Entrez, Source	conserved helix-loop-helix ubiquitous kinase	539	0.337	0.1224	No
6	MAPK8	MAPK8 Entrez, Source	mitogen-activated protein kinase 8	906	0.262	0.1048	No
7	IKBKB	IKBKB Entrez, Source	inhibitor of kappa light polypeptide gene enhancer in B-cells, kinase beta	1403	0.181	0.0645	No
8	IL18	IL18 Entrez, Source	interleukin 18 (interferon-gamma-inducing factor)	1714	0.145	0.0423	No
9	MAPK11	MAPK11 Entrez, Source	mitogen-activated protein kinase 11	1826	0.131	0.0420	No
10	MAPK12	MAPK12 Entrez, Source	mitogen-activated protein kinase 12	2182	0.095	0.0098	No
11	RELA	RELA Entrez, Source	v-rel reticuloendotheliosis viral oncogene homolog A, nuclear factor of kappa light polypeptide gene enhancer in B-cells 3, p65 (avian)	2685	0.044	-0.0444	No
12	CCL8	CCL8 Entrez, Source	chemokine (C-C motif) ligand 8	2731	0.040	-0.0458	No
13	BIRC2	BIRC2 Entrez, Source	baculoviral IAP repeat-containing 2	3159	0.002	-0.0952	No
14	HSP90AA1	HSP90AA1 Entrez, Source	heat shock protein 90kDa alpha (cytosolic), class A member 1	3719	-0.047	-0.1556	No
15	HSP90AB1	HSP90AB1 Entrez, Source	heat shock protein 90kDa alpha (cytosolic), class B member 1	4282	-0.094	-0.2119	No

16	MAPK3	MAPK3 Entrez, Source	mitogen-activated protein kinase 3	4509	-0.114	-0.2273	No
17	MAPK13	MAPK13 Entrez, Source	mitogen-activated protein kinase 13	4691	-0.129	-0.2361	No
18	NFKB1	NFKB1 Entrez, Source	nuclear factor of kappa light polypeptide gene enhancer in B-cells 1 (p105)	5476	-0.203	-0.3078	No
19	CASP1	CASP1 Entrez, Source	caspase 1, apoptosis-related cysteine peptidase (interleukin 1, beta, convertase)	5928	-0.250	-0.3364	No
20	MAPK10	MAPK10 Entrez, Source	mitogen-activated protein kinase 10	6420	-0.307	-0.3641	No
21	TNFAIP3	TNFAIP3 Entrez, Source	tumor necrosis factor, alpha-induced protein 3	6731	-0.358	-0.3659	No
22	IKBKG	IKBKG Entrez, Source	inhibitor of kappa light polypeptide gene enhancer in B-cells, kinase gamma	7299	-0.479	-0.3861	Yes
23	SUGT1	SUGT1 Entrez, Source	SGT1, suppressor of G2 allele of SKP1 (S. cerevisiae)	7362	-0.498	-0.3458	Yes
24	MAPK1	MAPK1 Entrez, Source	mitogen-activated protein kinase 1	7446	-0.522	-0.3057	Yes
25	NFKBIB	NFKBIB Entrez, Source	nuclear factor of kappa light polypeptide gene enhancer in B-cells inhibitor, beta	7465	-0.529	-0.2573	Yes
26	TRAF6	TRAF6 Entrez, Source	TNF receptor-associated factor 6	7512	-0.544	-0.2108	Yes
27	HSP90B1	HSP90B1 Entrez, Source	heat shock protein 90kDa beta (Grp94), member 1	7620	-0.578	-0.1680	Yes
28	MAP3K7	MAP3K7 Entrez, Source	mitogen-activated protein kinase kinase kinase 7	8090	-0.803	-0.1460	Yes
29	MAPK14	MAPK14 Entrez, Source	mitogen-activated protein kinase 14	8194	-0.875	-0.0745	Yes
30	MAPK9	MAPK9 Entrez, Source	mitogen-activated protein kinase 9	8528	-1.320	0.0127	Yes

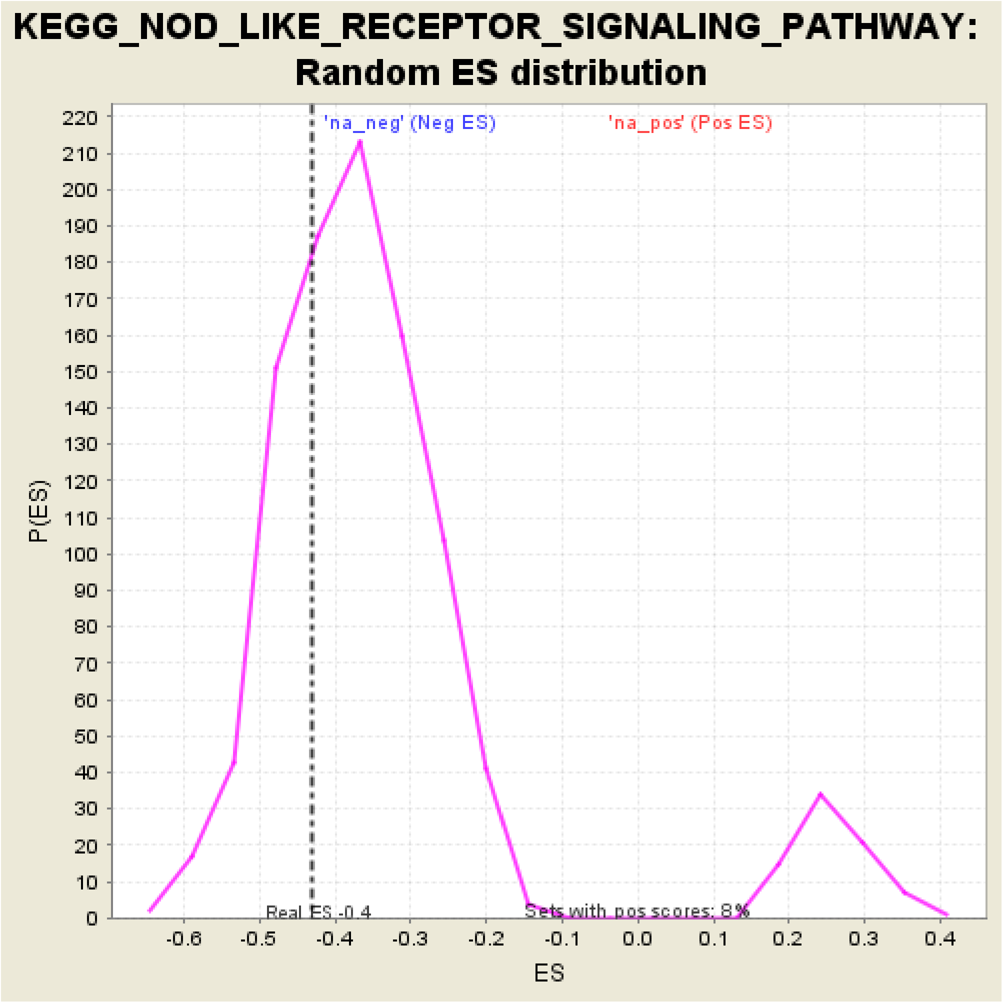

Fig 2: KEGG_NOD_LIKE_RECEPTOR_SIGNALING_PATHWAY: Random ES distribution

Gene set null distribution of ES for KEGG_NOD_LIKE_RECEPTOR_SIGNALING_PATHWAY

Table: GSEA Results Summary

Dataset	GSEA_18M_collapsed
Phenotype	NoPhenotypeAvailable
Upregulated in class	na_neg
GeneSet	KEGG_ENDOCYTOSIS
Enrichment Score (ES)	-0.54580843
Normalized Enrichment Score (NES)	-1.6477028
Nominal p-value	0.0
FDR q-value	0.056569893
FWER p-Value	0.256

Fig 1: Enrichment plot: KEGG_ENDOCYTOSIS
Profile of the Running ES Score & Positions of GeneSet Members on the Rank Ordered List

Table: GSEA details [plain text format]

	PROBE	GENE SYMBOL	GENE_TITLE	RANK IN GENE LIST	RANK METRIC SCORE	RUNNING ES	CORE ENRICHMENT
1	PLD2	PLD2 Entrez, Source	phospholipase D2	408	0.383	-0.0394	No
2	DAB2	DAB2 Entrez, Source	disabled homolog 2, mitogen-responsive phosphoprotein (Drosophila)	505	0.347	-0.0429	No
3	ADRB1	ADRB1 Entrez, Source	adrenergic, beta-1-, receptor	604	0.320	-0.0474	No
4	FLT1	FLT1 Entrez, Source	fms-related tyrosine kinase 1 (vascular endothelial growth factor/vascular permeability factor receptor)	800	0.281	-0.0640	No
5	IGF1R	IGF1R Entrez, Source	insulin-like growth factor 1 receptor	1122	0.222	-0.0967	No
6	CBLB	CBLB Entrez, Source	Cas-Br-M (murine) ecotropic retroviral transforming sequence b	1168	0.215	-0.0973	No
7	CLTC	CLTC Entrez,	clathrin, heavy chain (Hc)	1194	0.211	-0.0955	No

		Source					
8	PIP5K1B	PIP5K1B Entrez, Source	phosphatidylinositol-4-phosphate 5-kinase, type I, beta	1254	0.202	-0.0980	No
9	F2R	F2R Entrez, Source	coagulation factor II (thrombin) receptor	1279	0.197	-0.0964	No
10	PLD1	PLD1 Entrez, Source	phospholipase D1, phosphatidylcholine-specific	1289	0.196	-0.0931	No
11	IL2RB	IL2RB Entrez, Source	interleukin 2 receptor, beta	1344	0.188	-0.0953	No
12	ADRB3	ADRB3 Entrez, Source	adrenergic, beta-3-, receptor	1367	0.185	-0.0938	No
13	USP8	USP8 Entrez, Source	ubiquitin specific peptidase 8	1507	0.170	-0.1063	No
14	ADRBK2	ADRBK2 Entrez, Source	adrenergic, beta, receptor kinase 2	1691	0.148	-0.1245	No
15	NTRK1	NTRK1 Entrez, Source	neurotrophic tyrosine kinase, receptor, type 1	1695	0.147	-0.1216	No
16	RAB11B	RAB11B Entrez, Source	RAB11B, member RAS oncogene family	1726	0.144	-0.1219	No
17	PSD	PSD Entrez, Source	pleckstrin and Sec7 domain containing	1759	0.139	-0.1226	No
18	HSPA8	HSPA8 Entrez, Source	heat shock 70kDa protein 8	1905	0.122	-0.1369	No
19	AP2A1	AP2A1 Entrez, Source	adaptor-related protein complex 2, alpha 1 subunit	1919	0.121	-0.1357	No
20	SMURF1	SMURF1 Entrez, Source	SMAD specific E3 ubiquitin protein ligase 1	1954	0.117	-0.1371	No
21	CCR5	CCR5 Entrez, Source	chemokine (C-C motif) receptor 5	1973	0.115	-0.1367	No
22	CLTA	CLTA Entrez, Source	clathrin, light chain (Lca)	2024	0.110	-0.1401	No
23	VPS37C	VPS37C Entrez, Source	vacuolar protein sorting 37 homolog C (S. cerevisiae)	2264	0.086	-0.1662	No

24	CXCR4	CXCR4 Entrez, Source	chemokine (C-X-C motif) receptor 4	2355	0.078	-0.1751	No
25	IQSEC2	IQSEC2 Entrez, Source	IQ motif and Sec7 domain 2	2690	0.044	-0.2133	No
26	IQSEC1	IQSEC1 Entrez, Source	IQ motif and Sec7 domain 1	2777	0.035	-0.2226	No
27	VPS4B	VPS4B Entrez, Source	vacuolar protein sorting 4 homolog B (S. cerevisiae)	2781	0.035	-0.2222	No
28	TFRC	TFRC Entrez, Source	transferrin receptor (p90, CD71)	2950	0.020	-0.2414	No
29	SH3GL3	SH3GL3 Entrez, Source	SH3-domain GRB2-like 3	2987	0.016	-0.2453	No
30	ADRB2	ADRB2 Entrez, Source	adrenergic, beta-2-, receptor, surface	3187	-0.000	-0.2686	No
31	ITCH	ITCH Entrez, Source	itchy homolog E3 ubiquitin protein ligase (mouse)	3262	-0.007	-0.2772	No
32	EGFR	EGFR Entrez, Source	epidermal growth factor receptor (erythroblastic leukemia viral (v-erb-b) oncogene homolog, avian)	3352	-0.015	-0.2873	No
33	EPN1	EPN1 Entrez, Source	epsin 1	3398	-0.019	-0.2921	No
34	PRKCI	PRKCI Entrez, Source	protein kinase C, iota	3461	-0.023	-0.2989	No
35	LDLR	LDLR Entrez, Source	low density lipoprotein receptor (familial hypercholesterolemia)	3513	-0.027	-0.3043	No
36	PSD2	PSD2 Entrez, Source	pleckstrin and Sec7 domain containing 2	3626	-0.039	-0.3165	No
37	VPS37D	VPS37D Entrez, Source	vacuolar protein sorting 37 homolog D (S. cerevisiae)	3655	-0.042	-0.3189	No
38	GIT2	GIT2 Entrez, Source	G protein-coupled receptor kinase interactor 2	3866	-0.058	-0.3422	No
39	PARD6B	PARD6B Entrez, Source	par-6 partitioning defective 6 homolog beta (C. elegans)	4010	-0.072	-0.3574	No

40	EPN2	EPN2 Entrez, Source	epsin 2	4042	-0.074	-0.3594	No
41	AP2M1	AP2M1 Entrez, Source	adaptor-related protein complex 2, mu 1 subunit	4116	-0.081	-0.3662	No
42	HSPA1B	HSPA1B Entrez, Source	heat shock 70kDa protein 1B	4249	-0.092	-0.3796	No
43	ARRB2	ARRB2 Entrez, Source	arrestin, beta 2	4303	-0.096	-0.3837	No
44	SH3GL1	SH3GL1 Entrez, Source	SH3-domain GRB2-like 1	4373	-0.102	-0.3896	No
45	SNF8	SNF8 Entrez, Source	SNF8, ESCRT-II complex subunit, homolog (S. cerevisiae)	4395	-0.103	-0.3897	No
46	ERBB3	ERBB3 Entrez, Source	v-erb-b2 erythroblastic leukemia viral oncogene homolog 3 (avian)	4711	-0.131	-0.4238	No
47	GIT1	GIT1 Entrez, Source	G protein-coupled receptor kinase interactor 1	4714	-0.131	-0.4211	No
48	ARFGAP1	ARFGAP1 Entrez, Source	ADP-ribosylation factor GTPase activating protein 1	4800	-0.139	-0.4280	No
49	CSF1R	CSF1R Entrez, Source	colony stimulating factor 1 receptor, formerly McDonough feline sarcoma viral (v-fms) oncogene homolog	4946	-0.153	-0.4416	No
50	RAB5C	RAB5C Entrez, Source	RAB5C, member RAS oncogene family	5085	-0.165	-0.4542	No
51	EPN3	EPN3 Entrez, Source	epsin 3	5124	-0.169	-0.4549	No
52	IQSEC3	IQSEC3 Entrez, Source	IQ motif and Sec7 domain 3	5154	-0.171	-0.4545	No
53	ARFGAP3	ARFGAP3 Entrez, Source	ADP-ribosylation factor GTPase activating protein 3	5251	-0.180	-0.4618	No
54	HSPA2	HSPA2 Entrez, Source	heat shock 70kDa protein 2	5320	-0.186	-0.4656	No
55	EHD2	EHD2 Entrez, Source	EH-domain containing 2	5323	-0.186	-0.4617	No

56	SH3GLB1	SH3GLB1 Entrez, Source	SH3-domain GRB2-like endophilin B1	5409	-0.196	-0.4674	No
57	RAB11FIP5	RAB11FIP5 Entrez, Source	RAB11 family interacting protein 5 (class I)	5518	-0.207	-0.4754	No
58	STAM	STAM Entrez, Source	signal transducing adaptor molecule (SH3 domain and ITAM motif) 1	5624	-0.217	-0.4829	No
59	VPS28	VPS28 Entrez, Source	vacuolar protein sorting 28 homolog (S. cerevisiae)	5632	-0.217	-0.4789	No
60	HSPA1A	HSPA1A Entrez, Source	heat shock 70kDa protein 1A	5635	-0.218	-0.4744	No
61	EHD3	EHD3 Entrez, Source	EH-domain containing 3	5780	-0.233	-0.4861	No
62	PSD3	PSD3 Entrez, Source	pleckstrin and Sec7 domain containing 3	5918	-0.249	-0.4966	No
63	VPS37A	VPS37A Entrez, Source	vacuolar protein sorting 37 homolog A (S. cerevisiae)	5952	-0.252	-0.4949	No
64	VPS37B	VPS37B Entrez, Source	vacuolar protein sorting 37 homolog B (S. cerevisiae)	6101	-0.269	-0.5063	No
65	MDM2	MDM2 Entrez, Source	Mdm2, transformed 3T3 cell double minute 2, p53 binding protein (mouse)	6102	-0.269	-0.5003	No
66	EHD4	EHD4 Entrez, Source	EH-domain containing 4	6136	-0.272	-0.4982	No
67	CLTB	CLTB Entrez, Source	clathrin, light chain (Lcb)	6176	-0.276	-0.4966	No
68	RAB4A	RAB4A Entrez, Source	RAB4A, member RAS oncogene family	6227	-0.283	-0.4962	No
69	DNM2	DNM2 Entrez, Source	dynamin 2	6331	-0.296	-0.5017	No
70	PRKCZ	PRKCZ Entrez, Source	protein kinase C, zeta	6381	-0.303	-0.5008	No
71	STAM2	STAM2 Entrez, Source	signal transducing adaptor molecule (SH3 domain and ITAM motif) 2	6394	-0.305	-0.4954	No
72	EHD1	EHD1 Entrez, Source	EH-domain containing 1	6800	-0.370	-0.5347	No

73	ERBB4	ERBB4 Entrez, Source	v-erb-a erythroblastic leukemia viral oncogene homolog 4 (avian)	6803	-0.370	-0.5268	No
74	DNM3	DNM3 Entrez, Source	dynamin 3	6887	-0.386	-0.5279	No
75	LDLRAP1	LDLRAP1 Entrez, Source	low density lipoprotein receptor adaptor protein 1	7004	-0.407	-0.5325	No
76	PARD6A	PARD6A Entrez, Source	par-6 partitioning defective 6 homolog alpha (C.elegans)	7071	-0.422	-0.5309	No
77	ADRBK1	ADRBK1 Entrez, Source	adrenergic, beta, receptor kinase 1	7199	-0.452	-0.5358	Yes
78	PARD3	PARD3 Entrez, Source	par-3 partitioning defective 3 homolog (C. elegans)	7203	-0.453	-0.5261	Yes
79	RAB22A	RAB22A Entrez, Source	RAB22A, member RAS oncogene family	7244	-0.463	-0.5205	Yes
80	RABEP1	RABEP1 Entrez, Source	rabaptin, RAB GTPase binding effector protein 1	7253	-0.466	-0.5111	Yes
81	RAB11FIP2	RAB11FIP2 Entrez, Source	RAB11 family interacting protein 2 (class I)	7260	-0.468	-0.5015	Yes
82	VPS25	VPS25 Entrez, Source	vacuolar protein sorting 25 homolog (S. cerevisiae)	7287	-0.477	-0.4939	Yes
83	RAB11A	RAB11A Entrez, Source	RAB11A, member RAS oncogene family	7318	-0.484	-0.4867	Yes
84	SMURF2	SMURF2 Entrez, Source	SMAD specific E3 ubiquitin protein ligase 2	7359	-0.496	-0.4804	Yes
85	KIT	KIT Entrez, Source	v-kit Hardy-Zuckerman 4 feline sarcoma viral oncogene homolog	7372	-0.500	-0.4707	Yes
86	ARF6	ARF6 Entrez, Source	ADP-ribosylation factor 6	7420	-0.513	-0.4649	Yes
87	PDGFRA	PDGFRA Entrez, Source	platelet-derived growth factor receptor, alpha polypeptide	7458	-0.526	-0.4575	Yes
88	RAB11FIP4	RAB11FIP4 Entrez, Source	RAB11 family interacting protein 4 (class II)	7497	-0.540	-0.4500	Yes
89	TRAF6	TRAF6 Entrez, Source	TNF receptor-associated factor 6	7512	-0.544	-0.4396	Yes

90	NEDD4L	NEDD4L Entrez, Source	neural precursor cell expressed, developmentally down-regulated 4-like	7522	-0.548	-0.4285	Yes
91	RAB5B	RAB5B Entrez, Source	RAB5B, member RAS oncogene family	7631	-0.580	-0.4283	Yes
92	WWP1	WWP1 Entrez, Source	WW domain containing E3 ubiquitin protein ligase 1	7679	-0.601	-0.4205	Yes
93	SH3KBP1	SH3KBP1 Entrez, Source	SH3-domain kinase binding protein 1	7855	-0.678	-0.4260	Yes
94	RET	RET Entrez, Source	ret proto-oncogene (multiple endocrine neoplasia and medullary thyroid carcinoma 1, Hirschsprung disease)	7882	-0.688	-0.4138	Yes
95	PIP5K1A	PIP5K1A Entrez, Source	phosphatidylinositol-4-phosphate 5-kinase, type I, alpha	7914	-0.701	-0.4019	Yes
96	FGFR3	FGFR3 Entrez, Source	fibroblast growth factor receptor 3 (achondroplasia, thanatophoric dwarfism)	7961	-0.722	-0.3913	Yes
97	DNM1	DNM1 Entrez, Source	dynamin 1	7993	-0.744	-0.3784	Yes
98	PIP5K1C	PIP5K1C Entrez, Source	phosphatidylinositol-4-phosphate 5-kinase, type I, gamma	8086	-0.800	-0.3715	Yes
99	RNF41	RNF41 Entrez, Source	ring finger protein 41	8088	-0.801	-0.3538	Yes
100	PARD6G	PARD6G Entrez, Source	par-6 partitioning defective 6 homolog gamma (C. elegans)	8116	-0.817	-0.3389	Yes
101	AP2A2	AP2A2 Entrez, Source	adaptor-related protein complex 2, alpha 2 subunit	8259	-0.929	-0.3349	Yes
102	NEDD4	NEDD4 Entrez, Source	neural precursor cell expressed, developmentally down-regulated 4	8350	-1.020	-0.3229	Yes
103	SMAP1	SMAP1 Entrez, Source	stromal membrane-associated protein 1	8356	-1.023	-0.3008	Yes
104	CBL	CBL Entrez, Source	Cas-Br-M (murine) ecotropic retroviral transforming sequence	8393	-1.070	-0.2813	Yes
105	FGFR2	FGFR2 Entrez, Source	ffer syndrome, Jackson-Weiss syndrome)	8439	-1.146	-0.2611	Yes

106	STAMBP	STAMBP Entrez, Source	STAM binding protein	8452	-1.159	-0.2368	Yes
107	EPS15	EPS15 Entrez, Source	epidermal growth factor receptor pathway substrate 15	8469	-1.184	-0.2125	Yes
108	ZFYVE20	ZFYVE20 Entrez, Source	zinc finger, FYVE domain containing 20	8492	-1.223	-0.1879	Yes
109	PDCD6IP	PDCD6IP Entrez, Source	programmed cell death 6 interacting protein	8493	-1.224	-0.1608	Yes
110	SH3GL2	SH3GL2 Entrez, Source	SH3-domain GRB2-like 2	8533	-1.335	-0.1357	Yes
111	DNAJC6	DNAJC6 Entrez, Source	DnaJ (Hsp40) homolog, subfamily C, member 6	8536	-1.342	-0.1062	Yes
112	AP2B1	AP2B1 Entrez, Source	adaptor-related protein complex 2, beta 1 subunit	8540	-1.348	-0.0767	Yes
113	RAB31	RAB31 Entrez, Source	RAB31, member RAS oncogene family	8565	-1.397	-0.0485	Yes
114	DNM1L	DNM1L Entrez, Source	dynamin 1-like	8635	-2.564	0.0002	Yes

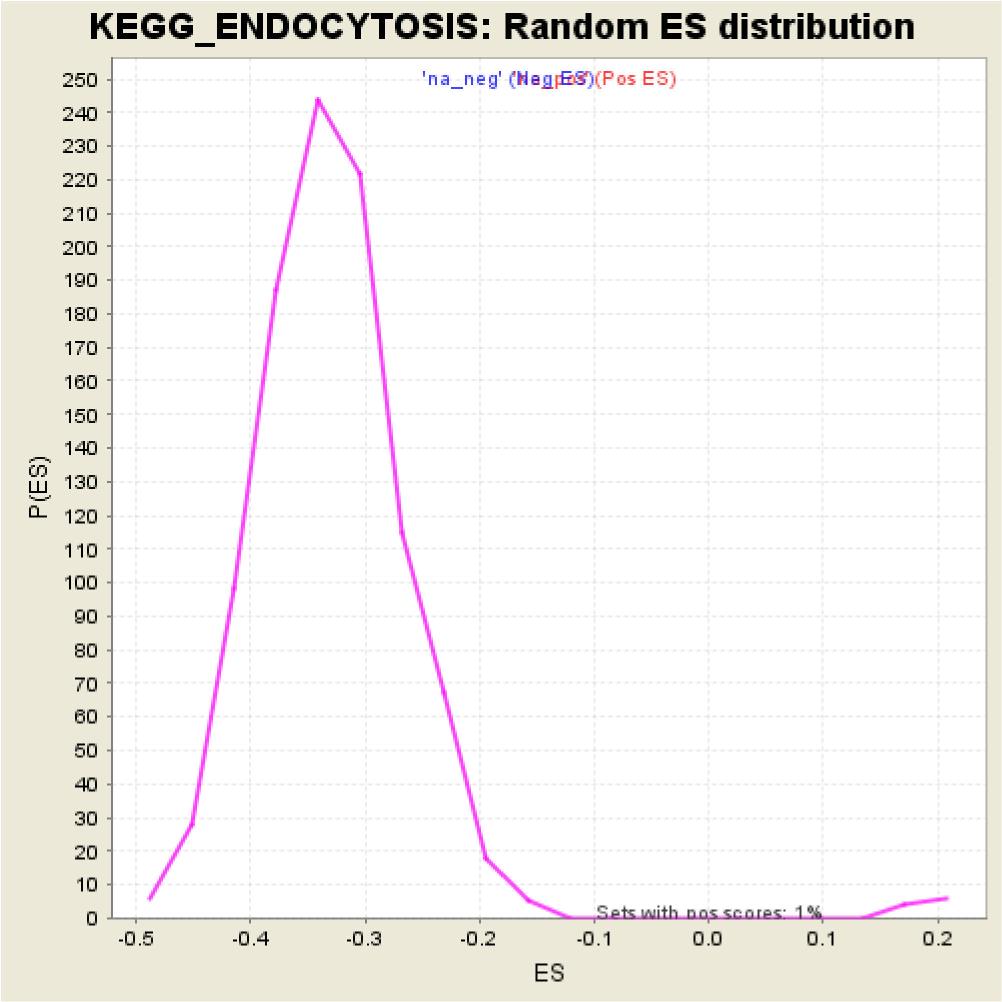

Fig 2: KEGG_ENDOCYTOSIS: Random ES distribution
Gene set null distribution of ES for KEGG_ENDOCYTOSIS

Table: GSEA Results Summary

Dataset	GSEA_18M_collapsed
Phenotype	NoPhenotypeAvailable
Upregulated in class	na_neg
GeneSet	KEGG_ALANINE_ASPARTATE_AND_GLUTAMATE_METABOLISM
Enrichment Score (ES)	-0.6235607
Normalized Enrichment Score (NES)	-1.5814384
Nominal p-value	0.010215664
FDR q-value	0.11791854
FWER p-Value	0.524

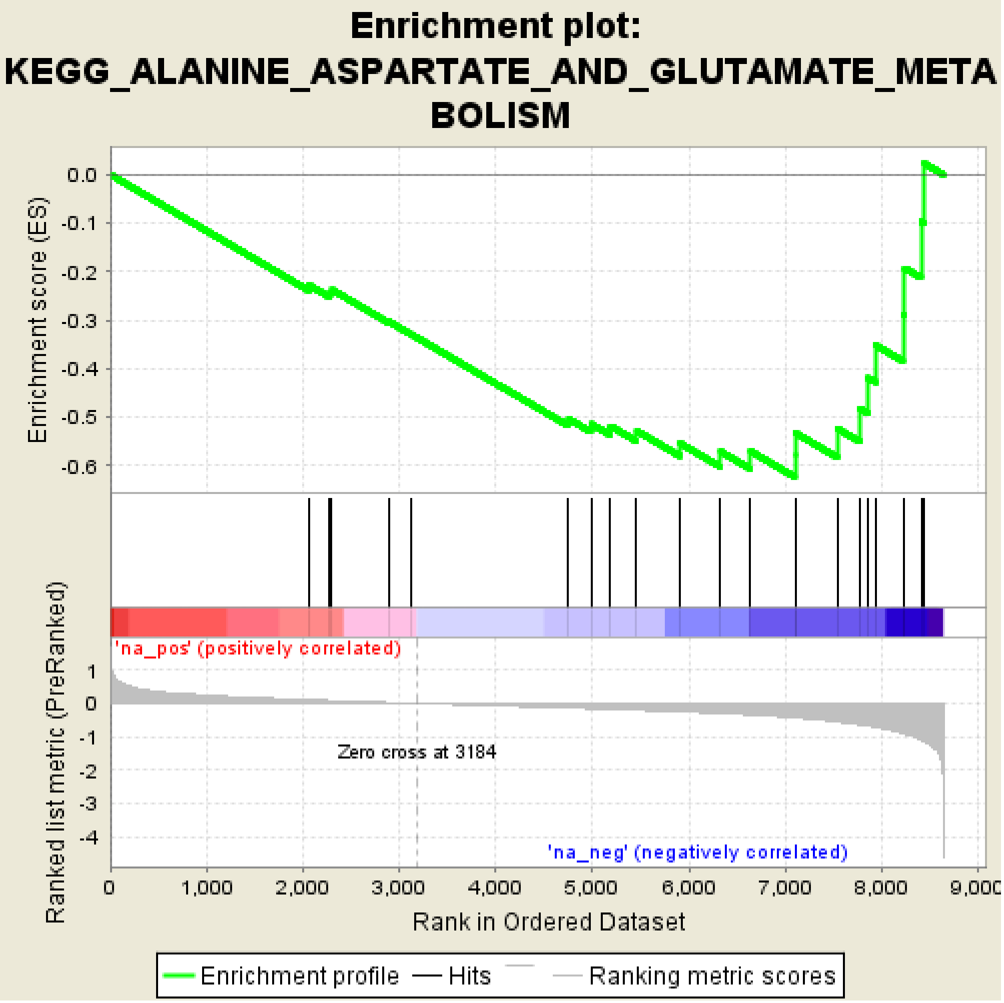

Fig 1: Enrichment plot: KEGG_ALANINE_ASPARTATE_AND_GLUTAMATE_METABOLISM
Profile of the Running ES Score & Positions of GeneSet Members on the Rank Ordered List

Table: GSEA details [plain text format]

	PROBE	GENE SYMBOL	GENE_TITLE	RANK IN GENE LIST	RANK METRIC SCORE	RUNNING ES	CORE ENRICHMENT
1	ASL	ASL Entrez, Source	argininosuccinate lyase	2059	0.107	-0.2275	No
2	GLS	GLS Entrez, Source	glutaminase	2277	0.085	-0.2436	No
3	GLS2	GLS2 Entrez, Source	glutaminase 2 (liver, mitochondrial)	2302	0.083	-0.2375	No
4	ACY3	ACY3 Entrez, Source	aspartoacylase (aminocyclase) 3	2888	0.026	-0.3027	No
5	CAD	CAD Entrez, Source	carbamoyl-phosphate synthetase 2, aspartate transcarbamylase, and dihydroorotase	3117	0.005	-0.3286	No
6	ALDH5A1	ALDH5A1 Entrez, Source	aldehyde dehydrogenase 5 family, member A1 (succinate-semialdehyde dehydrogenase)	4750	-0.134	-0.5036	No
7	PPAT	PPAT Entrez, Source	phosphoribosyl pyrophosphate amidotransferase	4983	-0.156	-0.5138	No
8	ADSS	ADSS Entrez, Source	adenylosuccinate synthase	5183	-0.174	-0.5182	No
9	ADSSL1	ADSSL1 Entrez, Source	adenylosuccinate synthase like 1	5457	-0.201	-0.5284	No
10	ABAT	ABAT Entrez, Source	4-aminobutyrate aminotransferase	5908	-0.247	-0.5541	No
11	GLUL	GLUL Entrez, Source	glutamate-ammonia ligase (glutamine synthetase)	6327	-0.296	-0.5710	No
12	ASPA	ASPA Entrez, Source	aspartoacylase (Canavan disease)	6638	-0.342	-0.5703	No
13	DDO	DDO Entrez, Source	D-aspartate oxidase	7098	-0.426	-0.5779	Yes
14	NIT2	NIT2 Entrez, Source	nitrilase family, member 2	7100	-0.426	-0.5323	Yes

15	GLUD1	GLUD1 Entrez, Source	glutamate dehydrogenase 1	7538	-0.554	-0.5237	Yes
16	ASS1	ASS1 Entrez, Source	argininosuccinate synthetase 1	7773	-0.640	-0.4822	Yes
17	GOT1	GOT1 Entrez, Source	glutamic-oxaloacetic transaminase 1, soluble (aspartate aminotransferase 1)	7856	-0.678	-0.4190	Yes
18	GFPT1	GFPT1 Entrez, Source	glutamine-fructose-6-phosphate transaminase 1	7935	-0.712	-0.3518	Yes
19	GAD2	GAD2 Entrez, Source	glutamate decarboxylase 2 (pancreatic islets and brain, 65kDa)	8220	-0.898	-0.2886	Yes
20	GOT2	GOT2 Entrez, Source	glutamic-oxaloacetic transaminase 2, mitochondrial (aspartate aminotransferase 2)	8235	-0.909	-0.1928	Yes
21	GPT2	GPT2 Entrez, Source	glutamic pyruvate transaminase (alanine aminotransferase) 2	8407	-1.082	-0.0967	Yes
22	GAD1	GAD1 Entrez, Source	glutamate decarboxylase 1 (brain, 67kDa)	8441	-1.150	0.0227	Yes

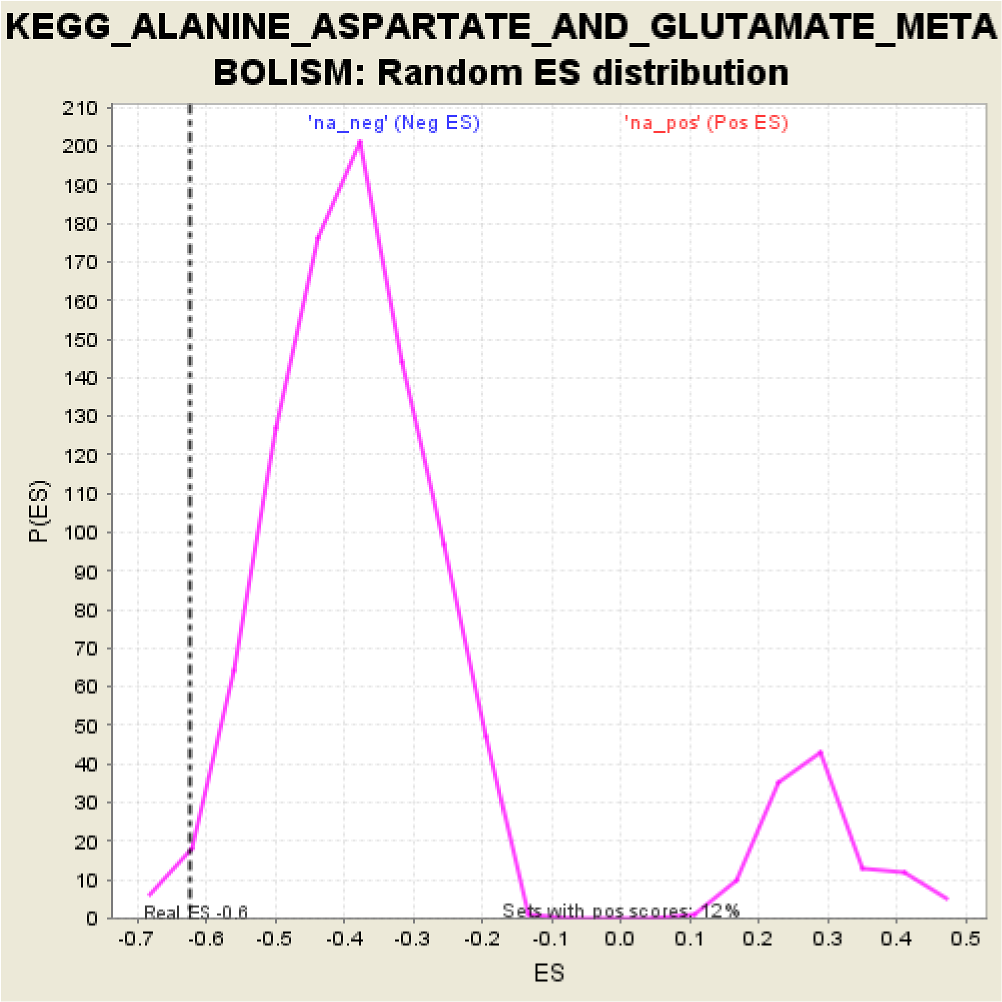

Fig 2: KEGG_ALANINE_ASPARTATE_AND_GLUTAMATE_METABOLISM: Random ES distribution
Gene set null distribution of ES for
KEGG_ALANINE_ASPARTATE_AND_GLUTAMATE_METABOLISM

Table: GSEA Results Summary

Dataset	GSEA_18M_collapsed
Phenotype	NoPhenotypeAvailable
Upregulated in class	na_neg
GeneSet	KEGG_ENDOMETRIAL_CANCER
Enrichment Score (ES)	-0.5580239
Normalized Enrichment Score (NES)	-1.5566992
Nominal p-value	0.005393743
FDR q-value	0.13755791
FWER p-Value	0.644

Fig 1: Enrichment plot: KEGG_ENDOMETRIAL_CANCER
Profile of the Running ES Score & Positions of GeneSet Members on the Rank Ordered List

Table: GSEA details [plain text format]

	PROBE	GENE SYMBOL	GENE_TITLE	RANK IN GENE LIST	RANK METRIC SCORE	RUNNING ES	CORE ENRICHMENT
1	SOS2	SOS2 Entrez, Source	son of sevenless homolog 2 (Drosophila)	811	0.278	-0.0776	No
2	PTEN	PTEN Entrez, Source	phosphatase and tensin homolog (mutated in multiple advanced cancers 1)	855	0.271	-0.0663	No
3	AXIN1	AXIN1 Entrez, Source	axin 1	1233	0.204	-0.0978	No
4	PIK3CD	PIK3CD Entrez, Source	phosphoinositide-3-kinase, catalytic, delta polypeptide	1265	0.200	-0.0894	No
5	PIK3R5	PIK3R5 Entrez, Source	phosphoinositide-3-kinase, regulatory subunit 5, p101	1401	0.182	-0.0942	No
6	RAF1	RAF1 Entrez, Source	v-raf-1 murine leukemia viral oncogene homolog 1	1794	0.134	-0.1317	No
7	PIK3R2	PIK3R2 Entrez, Source	phosphoinositide-3-kinase, regulatory subunit 2 (p85 beta)	1989	0.114	-0.1474	No

8	PIK3CG	PIK3CG Entrez, Source	phosphoinositide-3-kinase, catalytic, gamma polypeptide	2211	0.092	-0.1676	No
9	PDPK1	PDPK1 Entrez, Source	3-phosphoinositide dependent protein kinase-1	3347	-0.015	-0.2988	No
10	EGFR	EGFR Entrez, Source	epidermal growth factor receptor (erythroblastic leukemia viral (v-erb-b) oncogene homolog, avian)	3352	-0.015	-0.2983	No
11	CCND1	CCND1 Entrez, Source	cyclin D1	3616	-0.038	-0.3266	No
12	TCF7	TCF7 Entrez, Source	transcription factor 7 (T-cell specific, HMG-box)	3636	-0.040	-0.3264	No
13	GSK3B	GSK3B Entrez, Source	glycogen synthase kinase 3 beta	4405	-0.104	-0.4095	No
14	CDH1	CDH1 Entrez, Source	cadherin 1, type 1, E-cadherin (epithelial)	4474	-0.111	-0.4107	No
15	MAPK3	MAPK3 Entrez, Source	mitogen-activated protein kinase 3	4509	-0.114	-0.4078	No
16	CASP9	CASP9 Entrez, Source	caspase 9, apoptosis-related cysteine peptidase	4581	-0.119	-0.4088	No
17	GRB2	GRB2 Entrez, Source	growth factor receptor-bound protein 2	4744	-0.134	-0.4196	No
18	NRAS	NRAS Entrez, Source	neuroblastoma RAS viral (v-ras) oncogene homolog	4809	-0.140	-0.4187	No
19	LEF1	LEF1 Entrez, Source	lymphoid enhancer-binding factor 1	5170	-0.173	-0.4501	No
20	MAP2K1	MAP2K1 Entrez, Source	mitogen-activated protein kinase kinase 1	5558	-0.211	-0.4824	No
21	TCF7L2	TCF7L2 Entrez, Source	transcription factor 7-like 2 (T-cell specific, HMG-box)	5722	-0.227	-0.4877	No
22	APC2	APC2 Entrez, Source	adenomatosis polyposis coli 2	6107	-0.269	-0.5162	No
23	MYC	MYC Entrez, Source	v-myc myelocytomatosis viral oncogene homolog (avian)	6413	-0.306	-0.5332	No

24	PIK3CB	PIK3CB Entrez, Source	phosphoinositide-3-kinase, catalytic, beta polypeptide	6592	-0.333	-0.5338	No
25	PIK3CA	PIK3CA Entrez, Source	phosphoinositide-3-kinase, catalytic, alpha polypeptide	6801	-0.370	-0.5357	Yes
26	AXIN2	AXIN2 Entrez, Source	axin 2 (conductin, axil)	6948	-0.397	-0.5288	Yes
27	ELK1	ELK1 Entrez, Source	ELK1, member of ETS oncogene family	7187	-0.449	-0.5295	Yes
28	PIK3R3	PIK3R3 Entrez, Source	phosphoinositide-3-kinase, regulatory subunit 3 (p55, gamma)	7209	-0.454	-0.5046	Yes
29	MAPK1	MAPK1 Entrez, Source	mitogen-activated protein kinase 1	7446	-0.522	-0.5006	Yes
30	CTNNA2	CTNNA2 Entrez, Source	catenin (cadherin-associated protein), alpha 2	7492	-0.538	-0.4734	Yes
31	CTNNA3	CTNNA3 Entrez, Source	catenin (cadherin-associated protein), alpha 3	7546	-0.555	-0.4461	Yes
32	SOS1	SOS1 Entrez, Source	son of sevenless homolog 1 (Drosophila)	7606	-0.574	-0.4184	Yes
33	APC	APC Entrez, Source	adenomatosis polyposis coli	7732	-0.619	-0.3957	Yes
34	AKT1	AKT1 Entrez, Source	v-akt murine thymoma viral oncogene homolog 1	8115	-0.817	-0.3909	Yes
35	AKT2	AKT2 Entrez, Source	v-akt murine thymoma viral oncogene homolog 2	8121	-0.821	-0.3421	Yes
36	ARAF	ARAF Entrez, Source	v-raf murine sarcoma 3611 viral oncogene homolog	8164	-0.845	-0.2960	Yes
37	BRAF	BRAF Entrez, Source	v-raf murine sarcoma viral oncogene homolog B1	8297	-0.967	-0.2532	Yes
38	CTNNB1	CTNNB1 Entrez, Source	catenin (cadherin-associated protein), beta 1, 88kDa	8376	-1.047	-0.1992	Yes
39	KRAS	KRAS Entrez, Source	v-Ki-ras2 Kirsten rat sarcoma viral oncogene homolog	8444	-1.153	-0.1375	Yes
40	PIK3R1	PIK3R1 Entrez, Source	phosphoinositide-3-kinase, regulatory subunit 1 (p85 alpha)	8463	-1.179	-0.0686	Yes

41	AKT3	AKT3 Entrez, Source	v-akt murine thymoma viral oncogene homolog 3 (protein kinase B, gamma)	8586	-1.473	0.0059	Yes

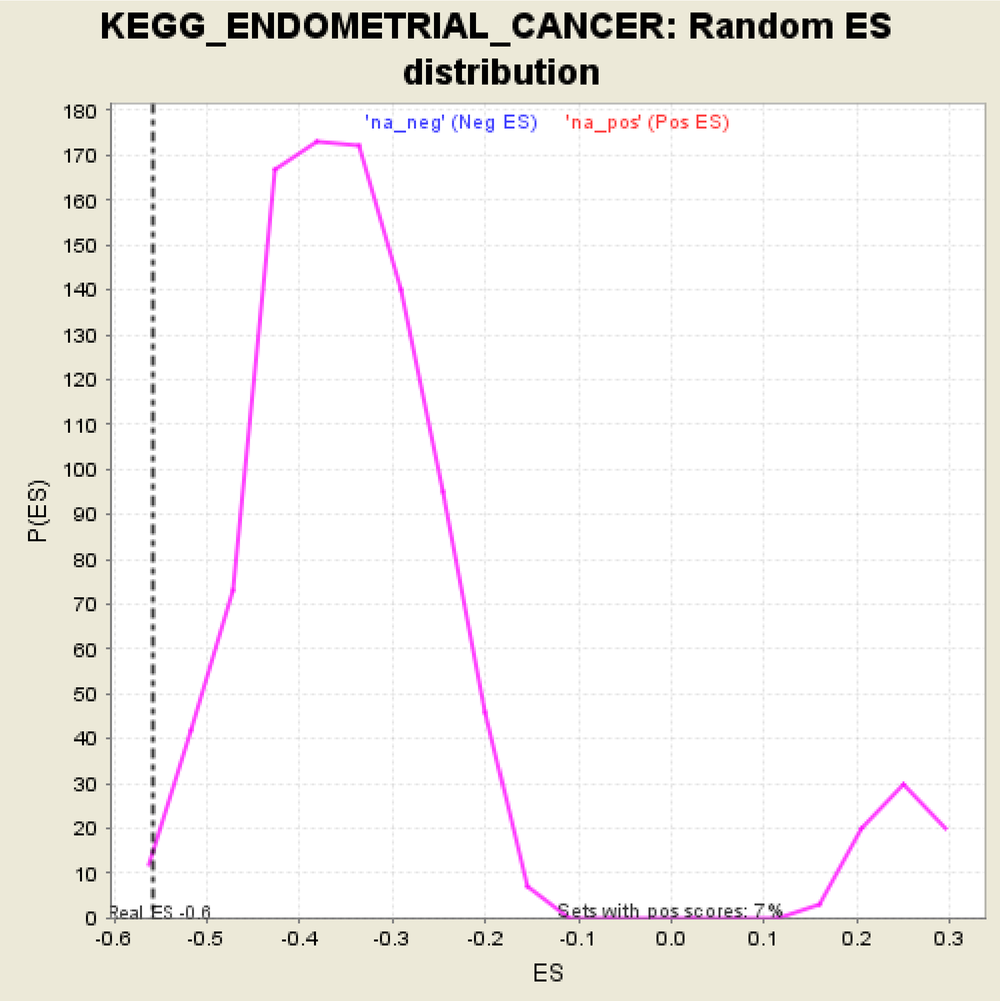

Fig 2: KEGG_ENDOMETRIAL_CANCER: Random ES distribution
Gene set null distribution of ES for KEGG_ENDOMETRIAL_CANCER

Table: GSEA Results Summary

Dataset	GSEA_18M_collapsed
Phenotype	NoPhenotypeAvailable
Upregulated in class	na_neg
GeneSet	KEGG_GAP_JUNCTION
Enrichment Score (ES)	-0.54057395
Normalized Enrichment Score (NES)	-1.5557499
Nominal p-value	0.004162331
FDR q-value	0.1217212
FWER p-Value	0.649

Fig 1: Enrichment plot: KEGG_GAP_JUNCTION
Profile of the Running ES Score & Positions of GeneSet Members on the Rank Ordered List

Table: GSEA details [plain text format]

	PROBE	GENE SYMBOL	GENE_TITLE	RANK IN GENE LIST	RANK METRIC SCORE	RUNNING ES	CORE ENRICHMENT
1	HTR2C	HTR2C Entrez, Source	5-hydroxytryptamine (serotonin) receptor 2C	17	0.987	0.0359	No
2	PDGFRB	PDGFRB Entrez, Source	platelet-derived growth factor receptor, beta polypeptide	324	0.427	0.0167	No
3	GNAS	GNAS Entrez, Source	GNAS complex locus	403	0.384	0.0224	No
4	ADRB1	ADRB1 Entrez, Source	adrenergic, beta-1-, receptor	604	0.320	0.0114	No
5	PDGFC	PDGFC Entrez, Source	platelet derived growth factor C	669	0.304	0.0156	No
6	PLCB3	PLCB3 Entrez, Source	phospholipase C, beta 3 (phosphatidylinositol-specific)	733	0.292	0.0195	No
7	SOS2	SOS2 Entrez, Source	son of sevenless homolog 2 (Drosophila)	811	0.278	0.0212	No

8	ADCY1	ADCY1 Entrez, Source	adenylate cyclase 1 (brain)	868	0.269	0.0250	No
9	PRKACA	PRKACA Entrez, Source	protein kinase, cAMP-dependent, catalytic, alpha	893	0.264	0.0324	No
10	TJP1	TJP1 Entrez, Source	tight junction protein 1 (zona occludens 1)	934	0.255	0.0375	No
11	ADCY5	ADCY5 Entrez, Source	adenylate cyclase 5	1188	0.212	0.0162	No
12	TUBB2A	TUBB2A Entrez, Source	tubulin, beta 2A	1497	0.171	-0.0131	No
13	ITPR2	ITPR2 Entrez, Source	inositol 1,4,5-triphosphate receptor, type 2	1615	0.156	-0.0208	No
14	RAF1	RAF1 Entrez, Source	v-raf-1 murine leukemia viral oncogene homolog 1	1794	0.134	-0.0363	No
15	PRKG1	PRKG1 Entrez, Source	protein kinase, cGMP-dependent, type I	1869	0.127	-0.0401	No
16	ADCY6	ADCY6 Entrez, Source	adenylate cyclase 6	2045	0.108	-0.0564	No
17	ADCY7	ADCY7 Entrez, Source	adenylate cyclase 7	2380	0.075	-0.0924	No
18	GRM1	GRM1 Entrez, Source	glutamate receptor, metabotropic 1	2606	0.052	-0.1166	No
19	MAPK7	MAPK7 Entrez, Source	mitogen-activated protein kinase 7	2852	0.029	-0.1441	No
20	PDGFB	PDGFB Entrez, Source	platelet-derived growth factor beta polypeptide (simian sarcoma viral (v-sis) oncogene homolog)	2886	0.026	-0.1469	No
21	EGFR	EGFR Entrez, Source	epidermal growth factor receptor (erythroblastic leukemia viral (v-erb-b) oncogene homolog, avian)	3352	-0.015	-0.2005	No
22	ADCY2	ADCY2 Entrez, Source	adenylate cyclase 2 (brain)	3580	-0.035	-0.2256	No
23	PLCB1	PLCB1 Entrez, Source	phospholipase C, beta 1 (phosphoinositide-specific)	3646	-0.041	-0.2316	No

24	DRD2	DRD2 Entrez, Source	dopamine receptor D2	4163	-0.085	-0.2885	No
25	ITPR3	ITPR3 Entrez, Source	inositol 1,4,5-triphosphate receptor, type 3	4184	-0.087	-0.2875	No
26	MAPK3	MAPK3 Entrez, Source	mitogen-activated protein kinase 3	4509	-0.114	-0.3209	No
27	GRB2	GRB2 Entrez, Source	growth factor receptor-bound protein 2	4744	-0.134	-0.3430	No
28	NRAS	NRAS Entrez, Source	neuroblastoma RAS viral (v-ras) oncogene homolog	4809	-0.140	-0.3451	No
29	PDGFA	PDGFA Entrez, Source	platelet-derived growth factor alpha polypeptide	5171	-0.173	-0.3805	No
30	MAP2K1	MAP2K1 Entrez, Source	mitogen-activated protein kinase kinase 1	5558	-0.211	-0.4174	No
31	GNAI2	GNAI2 Entrez, Source	guanine nucleotide binding protein (G protein), alpha inhibiting activity polypeptide 2	5765	-0.232	-0.4325	No
32	TUBB3	TUBB3 Entrez, Source	tubulin, beta 3	5999	-0.258	-0.4498	No
33	GNAI3	GNAI3 Entrez, Source	guanine nucleotide binding protein (G protein), alpha inhibiting activity polypeptide 3	6319	-0.295	-0.4756	No
34	MAP3K2	MAP3K2 Entrez, Source	mitogen-activated protein kinase kinase kinase 2	6405	-0.306	-0.4738	No
35	PRKCA	PRKCA Entrez, Source	protein kinase C, alpha	6631	-0.341	-0.4869	No
36	PRKG2	PRKG2 Entrez, Source	protein kinase, cGMP-dependent, type II	6933	-0.394	-0.5068	No
37	ADCY8	ADCY8 Entrez, Source	adenylate cyclase 8 (brain)	7224	-0.457	-0.5230	Yes
38	TUBA8	TUBA8 Entrez, Source	tubulin, alpha 8	7330	-0.487	-0.5165	Yes
39	GNAI1	GNAI1 Entrez, Source	guanine nucleotide binding protein (G protein), alpha inhibiting activity polypeptide 1	7333	-0.488	-0.4980	Yes
40	GRM5	GRM5 Entrez, Source	glutamate receptor, metabotropic 5	7423	-0.514	-0.4886	Yes

41	PDGFD	PDGFD Entrez, Source	platelet derived growth factor D	7429	-0.517	-0.4693	Yes
42	MAPK1	MAPK1 Entrez, Source	mitogen-activated protein kinase 1	7446	-0.522	-0.4511	Yes
43	PDGFRA	PDGFRA Entrez, Source	platelet-derived growth factor receptor, alpha polypeptide	7458	-0.526	-0.4322	Yes
44	ADCY3	ADCY3 Entrez, Source	adenylate cyclase 3	7460	-0.528	-0.4120	Yes
45	SOS1	SOS1 Entrez, Source	son of sevenless homolog 1 (Drosophila)	7606	-0.574	-0.4068	Yes
46	ITPR1	ITPR1 Entrez, Source	inositol 1,4,5-triphosphate receptor, type 1	7830	-0.669	-0.4071	Yes
47	PLCB4	PLCB4 Entrez, Source	phospholipase C, beta 4	7874	-0.685	-0.3858	Yes
48	PRKX	PRKX Entrez, Source	protein kinase, X-linked	7937	-0.713	-0.3656	Yes
49	GUCY1B3	GUCY1B3 Entrez, Source	guanylate cyclase 1, soluble, beta 3	8244	-0.915	-0.3661	Yes
50	MAP2K5	MAP2K5 Entrez, Source	mitogen-activated protein kinase kinase 5	8321	-0.987	-0.3370	Yes
51	KRAS	KRAS Entrez, Source	v-Ki-ras2 Kirsten rat sarcoma viral oncogene homolog	8444	-1.153	-0.3069	Yes
52	GUCY1A3	GUCY1A3 Entrez, Source	guanylate cyclase 1, soluble, alpha 3	8465	-1.181	-0.2638	Yes
53	CSNK1D	CSNK1D Entrez, Source	casein kinase 1, delta	8514	-1.258	-0.2210	Yes
54	GNAQ	GNAQ Entrez, Source	guanine nucleotide binding protein (G protein), q polypeptide	8542	-1.352	-0.1722	Yes
55	TUBB2B	TUBB2B Entrez, Source	tubulin, beta 2B	8577	-1.422	-0.1215	Yes
56	GJA1	GJA1 Entrez, Source	gap junction protein, alpha 1, 43kDa (connexin 43)	8612	-1.658	-0.0617	Yes
57	PRKACB	PRKACB Entrez, Source	protein kinase, cAMP-dependent, catalytic, beta	8614	-1.677	0.0027	Yes

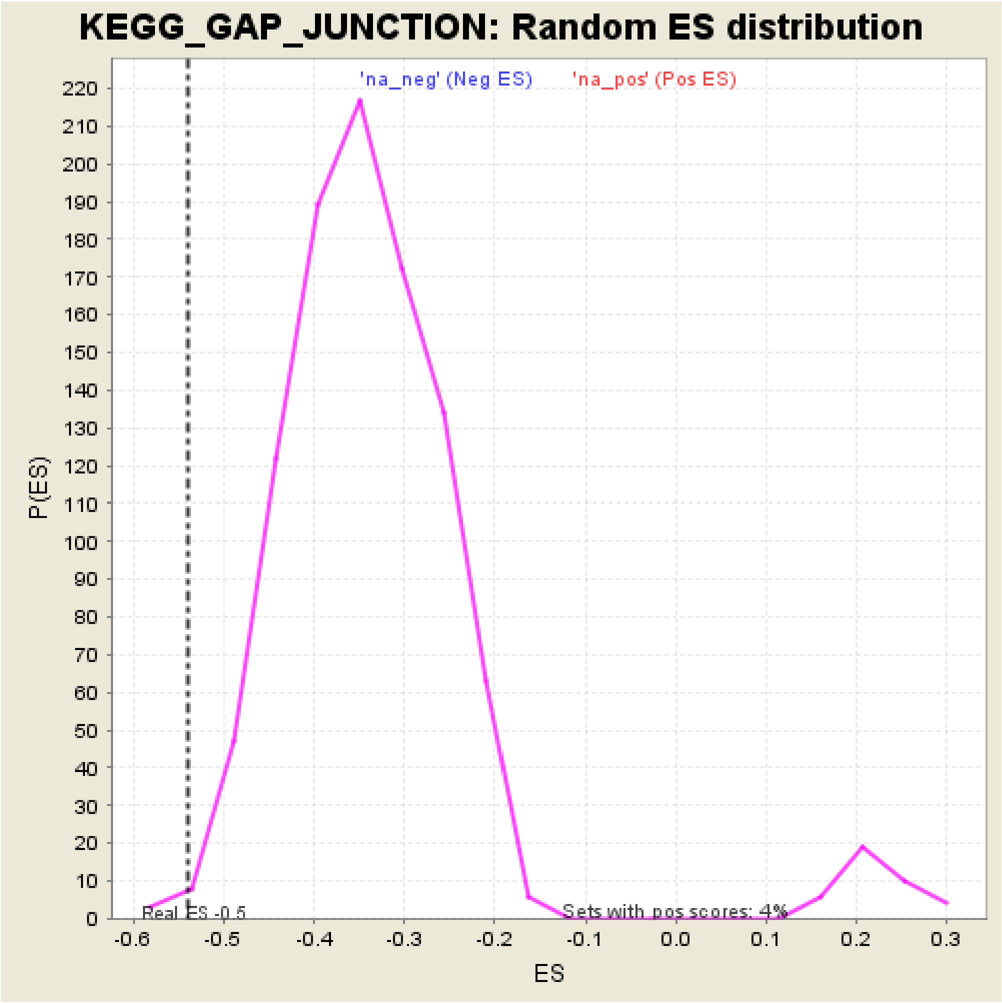

Fig 2: KEGG_GAP_JUNCTION: Random ES distribution
Gene set null distribution of ES for KEGG_GAP_JUNCTION

Table: GSEA Results Summary

Dataset	GSEA_18M_collapsed
Phenotype	NoPhenotypeAvailable
Upregulated in class	na_neg
GeneSet	KEGG_COLORECTAL_CANCER
Enrichment Score (ES)	-0.5416446
Normalized Enrichment Score (NES)	-1.5400101
Nominal p-value	0.0041710115
FDR q-value	0.13035494
FWER p-Value	0.723

Fig 1: Enrichment plot: KEGG_COLORECTAL_CANCER
Profile of the Running ES Score & Positions of GeneSet Members on the Rank Ordered List

Table: GSEA details [plain text format]

	PROBE	GENE SYMBOL	GENE_TITLE	RANK IN GENE LIST	RANK METRIC SCORE	RUNNING ES	CORE ENRICHMENT
1	BCL2	BCL2 Entrez, Source	B-cell CLL/lymphoma 2	787	0.283	-0.0784	No
2	MAPK8	MAPK8 Entrez, Source	mitogen-activated protein kinase 8	906	0.262	-0.0799	No
3	AXIN1	AXIN1 Entrez, Source	axin 1	1233	0.204	-0.1082	No
4	PIK3CD	PIK3CD Entrez, Source	phosphoinositide-3-kinase, catalytic, delta polypeptide	1265	0.200	-0.1025	No
5	PIK3R5	PIK3R5 Entrez, Source	phosphoinositide-3-kinase, regulatory subunit 5, p101	1401	0.182	-0.1097	No
6	FOS	FOS Entrez, Source	v-fos FBJ murine osteosarcoma viral oncogene homolog	1548	0.164	-0.1190	No
7	RAF1	RAF1 Entrez, Source	v-raf-1 murine leukemia viral oncogene homolog 1	1794	0.134	-0.1412	No

8	PIK3R2	PIK3R2 Entrez, Source	phosphoinositide-3-kinase, regulatory subunit 2 (p85 beta)	1989	0.114	-0.1585	No
9	PIK3CG	PIK3CG Entrez, Source	phosphoinositide-3-kinase, catalytic, gamma polypeptide	2211	0.092	-0.1799	No
10	CYCS	CYCS Entrez, Source	cytochrome c, somatic	2610	0.052	-0.2238	No
11	MSH3	MSH3 Entrez, Source	mutS homolog 3 (E. coli)	3020	0.013	-0.2709	No
12	TGFB2	TGFB2 Entrez, Source	transforming growth factor, beta 2	3370	-0.016	-0.3108	No
13	TGFBR2	TGFBR2 Entrez, Source	transforming growth factor, beta receptor II (70/80kDa)	3485	-0.025	-0.3229	No
14	CCND1	CCND1 Entrez, Source	cyclin D1	3616	-0.038	-0.3362	No
15	TCF7	TCF7 Entrez, Source	transcription factor 7 (T-cell specific, HMG-box)	3636	-0.040	-0.3366	No
16	GSK3B	GSK3B Entrez, Source	glycogen synthase kinase 3 beta	4405	-0.104	-0.4211	No
17	MAPK3	MAPK3 Entrez, Source	mitogen-activated protein kinase 3	4509	-0.114	-0.4278	No
18	CASP9	CASP9 Entrez, Source	caspase 9, apoptosis-related cysteine peptidase	4581	-0.119	-0.4304	No
19	SMAD4	SMAD4 Entrez, Source	SMAD, mothers against DPP homolog 4 (Drosophila)	4671	-0.127	-0.4348	No
20	TGFB3	TGFB3 Entrez, Source	transforming growth factor, beta 3	4880	-0.147	-0.4522	No
21	LEF1	LEF1 Entrez, Source	lymphoid enhancer-binding factor 1	5170	-0.173	-0.4777	No
22	MSH2	MSH2 Entrez, Source	mutS homolog 2, colon cancer, nonpolyposis type 1 (E. coli)	5307	-0.185	-0.4848	No
23	BIRC5	BIRC5 Entrez, Source	baculoviral IAP repeat-containing 5 (survivin)	5437	-0.199	-0.4905	No
24	MAP2K1	MAP2K1 Entrez, Source	mitogen-activated protein kinase kinase 1	5558	-0.211	-0.4946	No

25	TCF7L2	TCF7L2 Entrez, Source	transcription factor 7-like 2 (T-cell specific, HMG-box)	5722	-0.227	-0.5030	No
26	CASP3	CASP3 Entrez, Source	caspase 3, apoptosis-related cysteine peptidase	6055	-0.264	-0.5292	Yes
27	APC2	APC2 Entrez, Source	adenomatosis polyposis coli 2	6107	-0.269	-0.5225	Yes
28	JUN	JUN Entrez, Source	jun oncogene	6134	-0.272	-0.5128	Yes
29	RAC3	RAC3 Entrez, Source	ras-related C3 botulinum toxin substrate 3 (rho family, small GTP binding protein Rac3)	6207	-0.281	-0.5080	Yes
30	TGFB1	TGFB1 Entrez, Source	transforming growth factor, beta 1 (Camurati-Engelmann disease)	6283	-0.290	-0.5032	Yes
31	MYC	MYC Entrez, Source	v-myc myelocytomatosis viral oncogene homolog (avian)	6413	-0.306	-0.5038	Yes
32	MAPK10	MAPK10 Entrez, Source	mitogen-activated protein kinase 10	6420	-0.307	-0.4901	Yes
33	PIK3CB	PIK3CB Entrez, Source	phosphoinositide-3-kinase, catalytic, beta polypeptide	6592	-0.333	-0.4944	Yes
34	PIK3CA	PIK3CA Entrez, Source	phosphoinositide-3-kinase, catalytic, alpha polypeptide	6801	-0.370	-0.5012	Yes
35	TGFBR1	TGFBR1 Entrez, Source	transforming growth factor, beta receptor I (activin A receptor type II-like kinase, 53kDa)	6843	-0.376	-0.4883	Yes
36	DCC	DCC Entrez, Source	deleted in colorectal carcinoma	6868	-0.381	-0.4733	Yes
37	MSH6	MSH6 Entrez, Source	mutS homolog 6 (E. coli)	6890	-0.387	-0.4575	Yes
38	AXIN2	AXIN2 Entrez, Source	axin 2 (conductin, axil)	6948	-0.397	-0.4456	Yes
39	PIK3R3	PIK3R3 Entrez, Source	phosphoinositide-3-kinase, regulatory subunit 3 (p55, gamma)	7209	-0.454	-0.4546	Yes
40	SMAD2	SMAD2 Entrez, Source	SMAD, mothers against DPP homolog 2 (Drosophila)	7390	-0.505	-0.4518	Yes

41	MAPK1	MAPK1 Entrez, Source	mitogen-activated protein kinase 1	7446	-0.522	-0.4337	Yes
42	APC	APC Entrez, Source	adenomatosis polyposis coli	7732	-0.619	-0.4379	Yes
43	AKT1	AKT1 Entrez, Source	v-akt murine thymoma viral oncogene homolog 1	8115	-0.817	-0.4441	Yes
44	AKT2	AKT2 Entrez, Source	v-akt murine thymoma viral oncogene homolog 2	8121	-0.821	-0.4061	Yes
45	ARAF	ARAF Entrez, Source	v-raf murine sarcoma 3611 viral oncogene homolog	8164	-0.845	-0.3713	Yes
46	RAC1	RAC1 Entrez, Source	ras-related C3 botulinum toxin substrate 1 (rho family, small GTP binding protein Rac1)	8293	-0.964	-0.3410	Yes
47	SMAD3	SMAD3 Entrez, Source	SMAD, mothers against DPP homolog 3 (Drosophila)	8295	-0.964	-0.2959	Yes
48	BRAF	BRAF Entrez, Source	v-raf murine sarcoma viral oncogene homolog B1	8297	-0.967	-0.2507	Yes
49	CTNNB1	CTNNB1 Entrez, Source	catenin (cadherin-associated protein), beta 1, 88kDa	8376	-1.047	-0.2106	Yes
50	KRAS	KRAS Entrez, Source	v-Ki-ras2 Kirsten rat sarcoma viral oncogene homolog	8444	-1.153	-0.1643	Yes
51	PIK3R1	PIK3R1 Entrez, Source	phosphoinositide-3-kinase, regulatory subunit 1 (p85 alpha)	8463	-1.179	-0.1110	Yes
52	MAPK9	MAPK9 Entrez, Source	mitogen-activated protein kinase 9	8528	-1.320	-0.0565	Yes
53	AKT3	AKT3 Entrez, Source	v-akt murine thymoma viral oncogene homolog 3 (protein kinase B, gamma)	8586	-1.473	0.0059	Yes

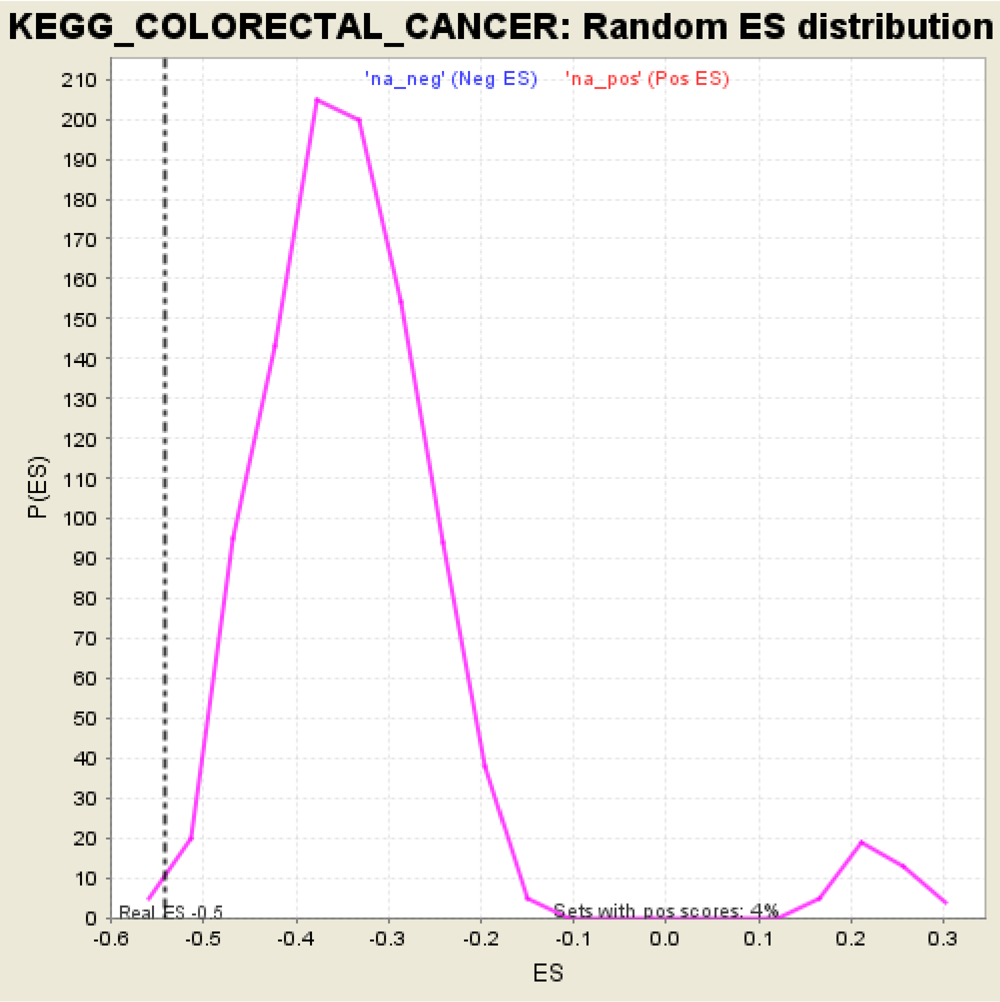

Fig 2: KEGG_COLORECTAL_CANCER: Random ES distribution
Gene set null distribution of ES for KEGG_COLORECTAL_CANCER

Table: GSEA Results Summary

Dataset	GSEA_18M_collapsed
Phenotype	NoPhenotypeAvailable
Upregulated in class	na_neg
GeneSet	KEGG_ERBB_SIGNALING_PATHWAY
Enrichment Score (ES)	-0.52239454
Normalized Enrichment Score (NES)	-1.5398098
Nominal p-value	0.0030737706
FDR q-value	0.11761037
FWER p-Value	0.723

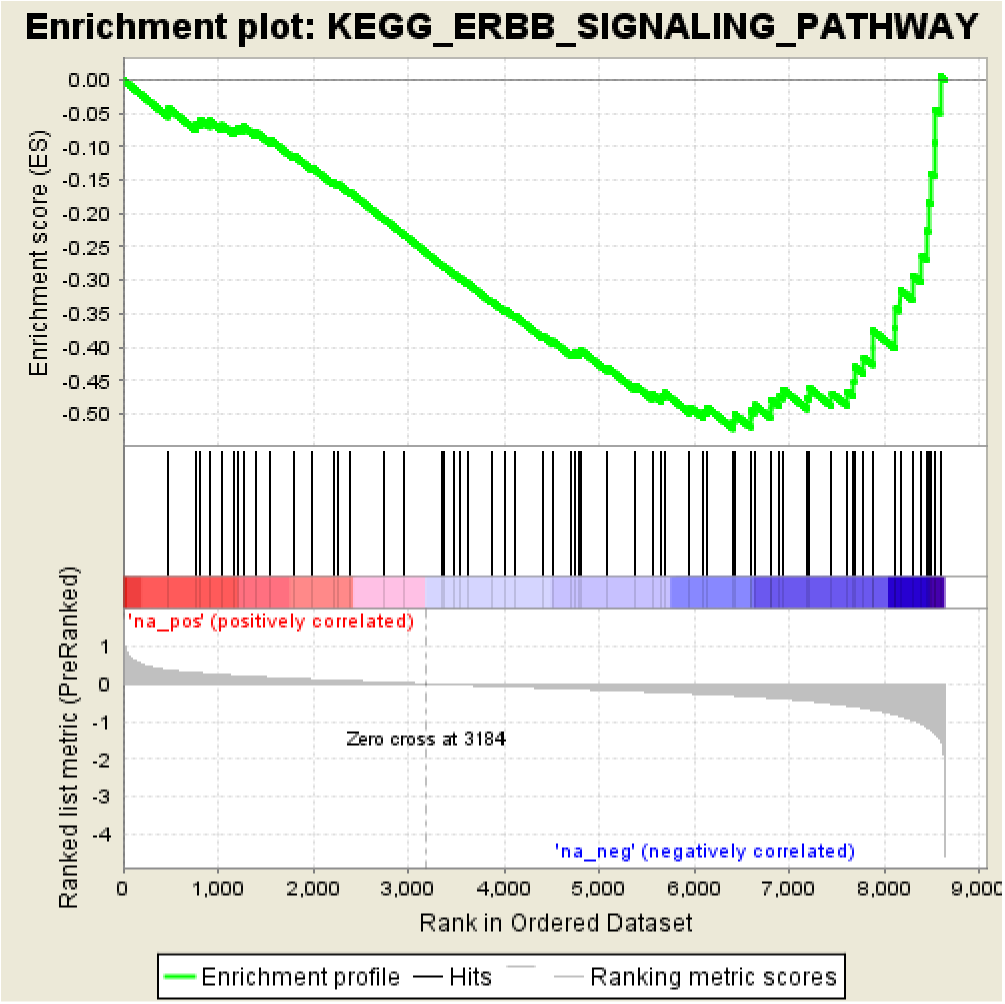

Fig 1: Enrichment plot: KEGG_ERBB_SIGNALING_PATHWAY
Profile of the Running ES Score & Positions of GeneSet Members on the Rank Ordered List

Table: GSEA details [plain text format]

	PROBE	GENE	GENE_TITLE	RANK	RANK	RUNNING	CORE

		SYMBOL		IN GENE LIST	METRIC SCORE	ES	ENRICHMENT
1	NCK2	NCK2 Entrez, Source	NCK adaptor protein 2	477	0.356	-0.0422	No
2	TGFA	TGFA Entrez, Source	transforming growth factor, alpha	769	0.287	-0.0653	No
3	SOS2	SOS2 Entrez, Source	son of sevenless homolog 2 (Drosophila)	811	0.278	-0.0595	No
4	MAPK8	MAPK8 Entrez, Source	mitogen-activated protein kinase 8	906	0.262	-0.0606	No
5	PAK6	PAK6 Entrez, Source	p21(CDKN1A)-activated kinase 6	1041	0.236	-0.0673	No
6	CBLB	CBLB Entrez, Source	Cas-Br-M (murine) ecotropic retroviral transforming sequence b	1168	0.215	-0.0739	No
7	NRG3	NRG3 Entrez, Source	neuregulin 3	1208	0.208	-0.0705	No
8	PIK3CD	PIK3CD Entrez, Source	phosphoinositide-3-kinase, catalytic, delta polypeptide	1265	0.200	-0.0695	No
9	PIK3R5	PIK3R5 Entrez, Source	phosphoinositide-3-kinase, regulatory subunit 5, p101	1401	0.182	-0.0784	No
10	RPS6KB2	RPS6KB2 Entrez, Source	ribosomal protein S6 kinase, 70kDa, polypeptide 2	1550	0.164	-0.0894	No
11	RAF1	RAF1 Entrez, Source	v-raf-1 murine leukemia viral oncogene homolog 1	1794	0.134	-0.1127	No
12	PIK3R2	PIK3R2 Entrez, Source	phosphoinositide-3-kinase, regulatory subunit 2 (p85 beta)	1989	0.114	-0.1311	No
13	PIK3CG	PIK3CG Entrez, Source	phosphoinositide-3-kinase, catalytic, gamma polypeptide	2211	0.092	-0.1534	No
14	ABL1	ABL1 Entrez, Source	v-abl Abelson murine leukemia viral oncogene homolog 1	2251	0.088	-0.1546	No
15	PAK3	PAK3 Entrez, Source	p21 (CDKN1A)-activated kinase 3	2388	0.075	-0.1676	No
16	PLCG2	PLCG2 Entrez, Source	phospholipase C, gamma 2 (phosphatidylinositol-specific)	2753	0.038	-0.2087	No

17	CDKN1A	CDKN1A Entrez, Source	cyclin-dependent kinase inhibitor 1A (p21, Cip1)	2964	0.018	-0.2325	No
18	EGFR	EGFR Entrez, Source	epidermal growth factor receptor (erythroblastic leukemia viral (v-erb-b) oncogene homolog, avian)	3352	-0.015	-0.2771	No
19	CAMK2G	CAMK2G Entrez, Source	calcium/calmodulin-dependent protein kinase (CaM kinase) II gamma	3386	-0.017	-0.2803	No
20	CDKN1B	CDKN1B Entrez, Source	cyclin-dependent kinase inhibitor 1B (p27, Kip1)	3490	-0.026	-0.2913	No
21	BTC	BTC Entrez, Source	betacellulin	3554	-0.032	-0.2974	No
22	CAMK2D	CAMK2D Entrez, Source	calcium/calmodulin-dependent protein kinase (CaM kinase) II delta	3620	-0.039	-0.3036	No
23	PAK7	PAK7 Entrez, Source	p21(CDKN1A)-activated kinase 7	3875	-0.059	-0.3310	No
24	STAT5B	STAT5B Entrez, Source	signal transducer and activator of transcription 5B	4017	-0.072	-0.3447	No
25	NCK1	NCK1 Entrez, Source	NCK adaptor protein 1	4109	-0.080	-0.3523	No
26	GSK3B	GSK3B Entrez, Source	glycogen synthase kinase 3 beta	4405	-0.104	-0.3828	No
27	MAPK3	MAPK3 Entrez, Source	mitogen-activated protein kinase 3	4509	-0.114	-0.3905	No
28	ERBB3	ERBB3 Entrez, Source	v-erb-b2 erythroblastic leukemia viral oncogene homolog 3 (avian)	4711	-0.131	-0.4090	No
29	GRB2	GRB2 Entrez, Source	growth factor receptor-bound protein 2	4744	-0.134	-0.4077	No
30	RPS6KB1	RPS6KB1 Entrez, Source	ribosomal protein S6 kinase, 70kDa, polypeptide 1	4796	-0.138	-0.4084	No
31	NRAS	NRAS Entrez, Source	neuroblastoma RAS viral (v-ras) oncogene homolog	4809	-0.140	-0.4045	No
32	SHC1	SHC1 Entrez, Source	SHC (Src homology 2 domain containing) transforming protein 1	5080	-0.164	-0.4298	No

33	GAB1	GAB1 Entrez, Source	GRB2-associated binding protein 1	5377	-0.192	-0.4570	No
34	MAP2K1	MAP2K1 Entrez, Source	mitogen-activated protein kinase kinase 1	5558	-0.211	-0.4701	No
35	PTK2	PTK2 Entrez, Source	PTK2 protein tyrosine kinase 2	5657	-0.220	-0.4732	No
36	CAMK2B	CAMK2B Entrez, Source	calcium/calmodulin-dependent protein kinase (CaM kinase) II beta	5683	-0.222	-0.4677	No
37	SHC4	SHC4 Entrez, Source	SHC (Src homology 2 domain containing) family, member 4	5953	-0.252	-0.4895	No
38	HBEGF	HBEGF Entrez, Source	heparin-binding EGF-like growth factor	6096	-0.268	-0.4959	No
39	JUN	JUN Entrez, Source	jun oncogene	6134	-0.272	-0.4900	No
40	MYC	MYC Entrez, Source	v-myc myelocytomatosis viral oncogene homolog (avian)	6413	-0.306	-0.5108	Yes
41	MAPK10	MAPK10 Entrez, Source	mitogen-activated protein kinase 10	6420	-0.307	-0.4999	Yes
42	PIK3CB	PIK3CB Entrez, Source	phosphoinositide-3-kinase, catalytic, beta polypeptide	6592	-0.333	-0.5072	Yes
43	SHC3	SHC3 Entrez, Source	SHC (Src homology 2 domain containing) transforming protein 3	6594	-0.333	-0.4947	Yes
44	PRKCA	PRKCA Entrez, Source	protein kinase C, alpha	6631	-0.341	-0.4860	Yes
45	PIK3CA	PIK3CA Entrez, Source	phosphoinositide-3-kinase, catalytic, alpha polypeptide	6801	-0.370	-0.4917	Yes
46	ERBB4	ERBB4 Entrez, Source	v-erb-a erythroblastic leukemia viral oncogene homolog 4 (avian)	6803	-0.370	-0.4778	Yes
47	NRG1	NRG1 Entrez, Source	neuregulin 1	6895	-0.388	-0.4737	Yes
48	PAK2	PAK2 Entrez, Source	p21 (CDKN1A)-activated kinase 2	6929	-0.394	-0.4627	Yes
49	ELK1	ELK1 Entrez, Source	ELK1, member of ETS oncogene family	7187	-0.449	-0.4757	Yes

50	PIK3R3	PIK3R3 Entrez, Source	phosphoinositide-3-kinase, regulatory subunit 3 (p55, gamma)	7209	-0.454	-0.4609	Yes
51	MAPK1	MAPK1 Entrez, Source	mitogen-activated protein kinase 1	7446	-0.522	-0.4687	Yes
52	SOS1	SOS1 Entrez, Source	son of sevenless homolog 1 (Drosophila)	7606	-0.574	-0.4655	Yes
53	MAP2K7	MAP2K7 Entrez, Source	mitogen-activated protein kinase kinase 7	7677	-0.600	-0.4510	Yes
54	MAP2K4	MAP2K4 Entrez, Source	mitogen-activated protein kinase kinase 4	7683	-0.601	-0.4288	Yes
55	SHC2	SHC2 Entrez, Source	SHC (Src homology 2 domain containing) transforming protein 2	7781	-0.643	-0.4158	Yes
56	CAMK2A	CAMK2A Entrez, Source	calcium/calmodulin-dependent protein kinase (CaM kinase) II alpha	7876	-0.685	-0.4008	Yes
57	PAK1	PAK1 Entrez, Source	p21/Cdc42/Rac1-activated kinase 1 (STE20 homolog, yeast)	7877	-0.686	-0.3748	Yes
58	AKT1	AKT1 Entrez, Source	v-akt murine thymoma viral oncogene homolog 1	8115	-0.817	-0.3715	Yes
59	AKT2	AKT2 Entrez, Source	v-akt murine thymoma viral oncogene homolog 2	8121	-0.821	-0.3410	Yes
60	ARAF	ARAF Entrez, Source	v-raf murine sarcoma 3611 viral oncogene homolog	8164	-0.845	-0.3139	Yes
61	BRAF	BRAF Entrez, Source	v-raf murine sarcoma viral oncogene homolog B1	8297	-0.967	-0.2927	Yes
62	CBL	CBL Entrez, Source	Cas-Br-M (murine) ecotropic retroviral transforming sequence	8393	-1.070	-0.2633	Yes
63	KRAS	KRAS Entrez, Source	v-Ki-ras2 Kirsten rat sarcoma viral oncogene homolog	8444	-1.153	-0.2254	Yes
64	PIK3R1	PIK3R1 Entrez, Source	phosphoinositide-3-kinase, regulatory subunit 1 (p85 alpha)	8463	-1.179	-0.1829	Yes
65	CRK	CRK Entrez, Source	v-crk sarcoma virus CT10 oncogene homolog (avian)	8490	-1.223	-0.1396	Yes
66	MAPK9	MAPK9 Entrez, Source	mitogen-activated protein kinase 9	8528	-1.320	-0.0940	Yes

67	CRKL	CRKL Entrez, Source	v-crk sarcoma virus CT10 oncogene homolog (avian)-like	8535	-1.339	-0.0440	Yes
68	AKT3	AKT3 Entrez, Source	v-akt murine thymoma viral oncogene homolog 3 (protein kinase B, gamma)	8586	-1.473	0.0060	Yes

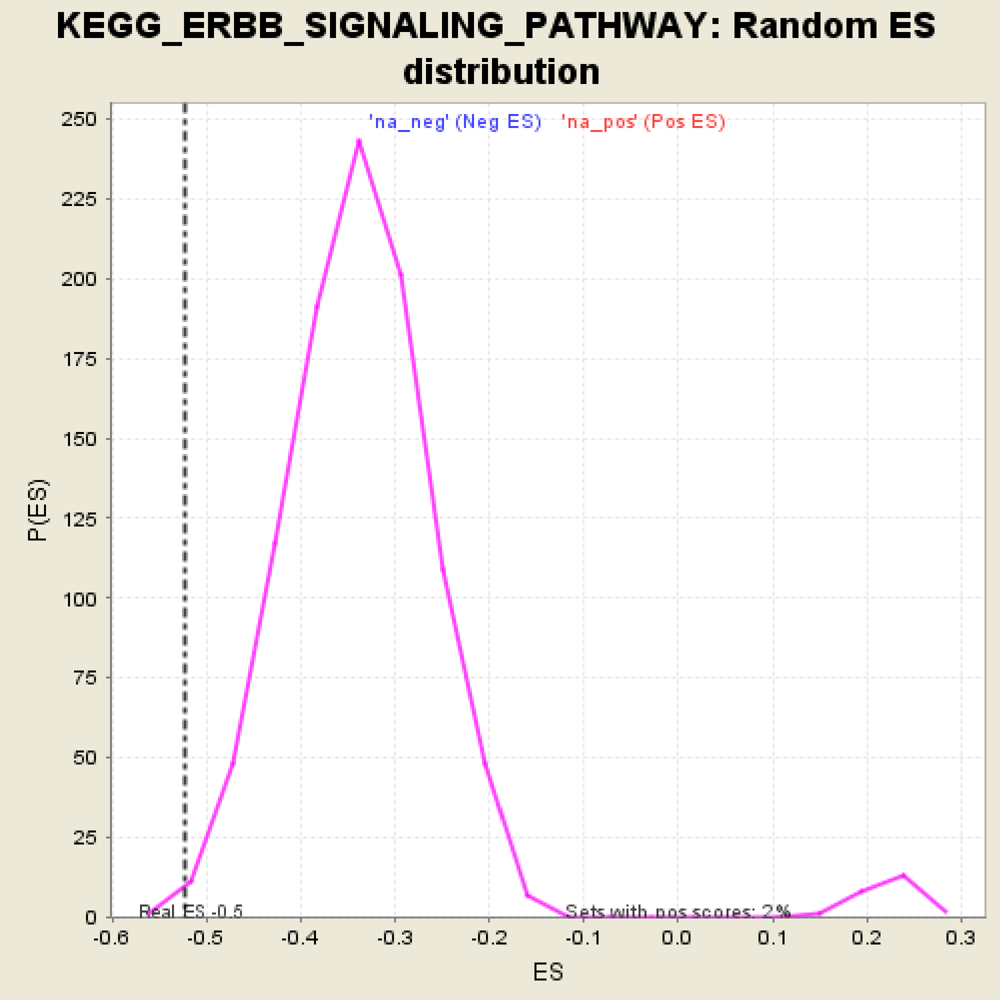

Fig 2: KEGG_ERBB_SIGNALING_PATHWAY: Random ES distribution
Gene set null distribution of ES for KEGG_ERBB_SIGNALING_PATHWAY

Table: GSEA Results Summary

Dataset	GSEA_18M_collapsed
Phenotype	NoPhenotypeAvailable
Upregulated in class	na_neg
GeneSet	KEGG_SPHINGOLIPID_METABOLISM
Enrichment Score (ES)	-0.59689146
Normalized Enrichment Score (NES)	-1.5293154
Nominal p-value	0.022727273
FDR q-value	0.12168655
FWER p-Value	0.766

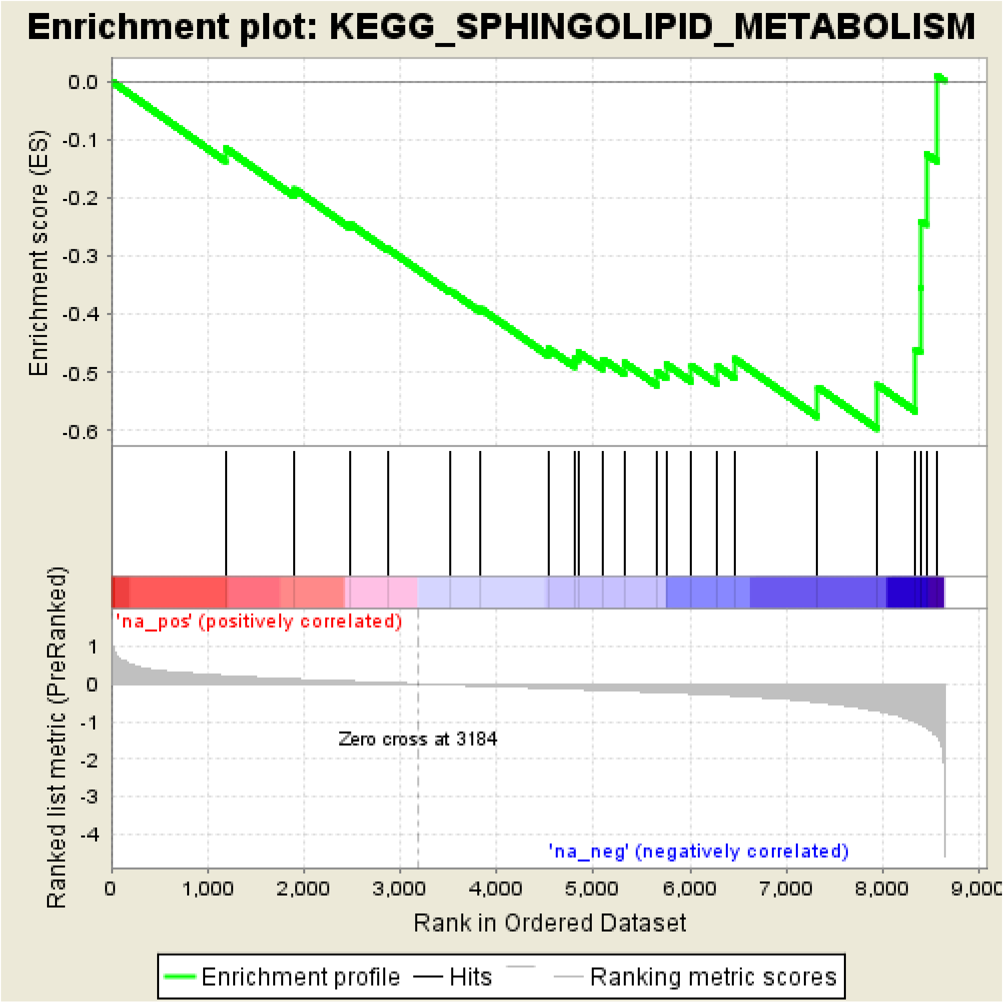

Fig 1: Enrichment plot: KEGG_SPHINGOLIPID_METABOLISM
Profile of the Running ES Score & Positions of GeneSet Members on the Rank Ordered List

Table: GSEA details [plain text format]

	PROBE	GENE	GENE_TITLE	RANK	RANK	RUNNING	CORE

		SYMBOL		IN GENE LIST	METRIC SCORE	ES	ENRICHMENT
1	GBA	GBA Entrez, Source	glucosidase, beta; acid (includes glucosylceramidase)	1185	0.212	-0.1151	No
2	PPAP2B	PPAP2B Entrez, Source	phosphatidic acid phosphatase type 2B	1901	0.123	-0.1852	No
3	ARSA	ARSA Entrez, Source	arylsulfatase A	2482	0.065	-0.2456	No
4	SPTLC2	SPTLC2 Entrez, Source	serine palmitoyltransferase, long chain base subunit 2	2868	0.027	-0.2874	No
5	NEU2	NEU2 Entrez, Source	sialidase 2 (cytosolic sialidase)	3521	-0.028	-0.3601	No
6	GAL3ST1	GAL3ST1 Entrez, Source	galactose-3-O-sulfotransferase 1	3837	-0.056	-0.3908	No
7	SMPD2	SMPD2 Entrez, Source	sphingomyelin phosphodiesterase 2, neutral membrane (neutral sphingomyelinase)	4529	-0.115	-0.4588	No
8	NEU1	NEU1 Entrez, Source	sialidase 1 (lysosomal sialidase)	4805	-0.139	-0.4760	No
9	SMPD3	SMPD3 Entrez, Source	sphingomyelin phosphodiesterase 3, neutral membrane (neutral sphingomyelinase II)	4843	-0.144	-0.4651	No
10	GLB1	GLB1 Entrez, Source	galactosidase, beta 1	5096	-0.165	-0.4769	No
11	ASAH2	ASAH2 Entrez, Source	N-acylsphingosine amidohydrolase (non-lysosomal ceramidase) 2	5318	-0.186	-0.4829	No
12	PPAP2C	PPAP2C Entrez, Source	phosphatidic acid phosphatase type 2C	5658	-0.220	-0.4990	No
13	PPAP2A	PPAP2A Entrez, Source	phosphatidic acid phosphatase type 2A	5754	-0.230	-0.4858	No
14	SGPP2	SGPP2 Entrez, Source	sphingosine-1-phosphate phosphotase 2	6014	-0.259	-0.4885	No
15	SPHK1	SPHK1 Entrez, Source	sphingosine kinase 1	6284	-0.290	-0.4891	No

16	CERK	CERK Entrez, Source	ceramide kinase	6462	-0.315	-0.4764	No
17	SMPD4	SMPD4 Entrez, Source	sphingomyelin phosphodiesterase 4, neutral membrane (neutral sphingomyelinase-3)	7325	-0.487	-0.5250	Yes
18	GLA	GLA Entrez, Source	galactosidase, alpha	7945	-0.717	-0.5212	Yes
19	SGPL1	SGPL1 Entrez, Source	sphingosine-1-phosphate lyase 1	8339	-1.009	-0.4604	Yes
20	GALC	GALC Entrez, Source	galactosylceramidase	8389	-1.067	-0.3534	Yes
21	B4GALT6	B4GALT6 Entrez, Source	UDP-Gal:betaGlcNAc beta 1,4-galactosyltransferase, polypeptide 6	8397	-1.072	-0.2410	Yes
22	UGCG	UGCG Entrez, Source	UDP-glucose ceramide glucosyltransferase	8449	-1.156	-0.1250	Yes
23	SGPP1	SGPP1 Entrez, Source	sphingosine-1-phosphate phosphatase 1	8562	-1.389	0.0087	Yes

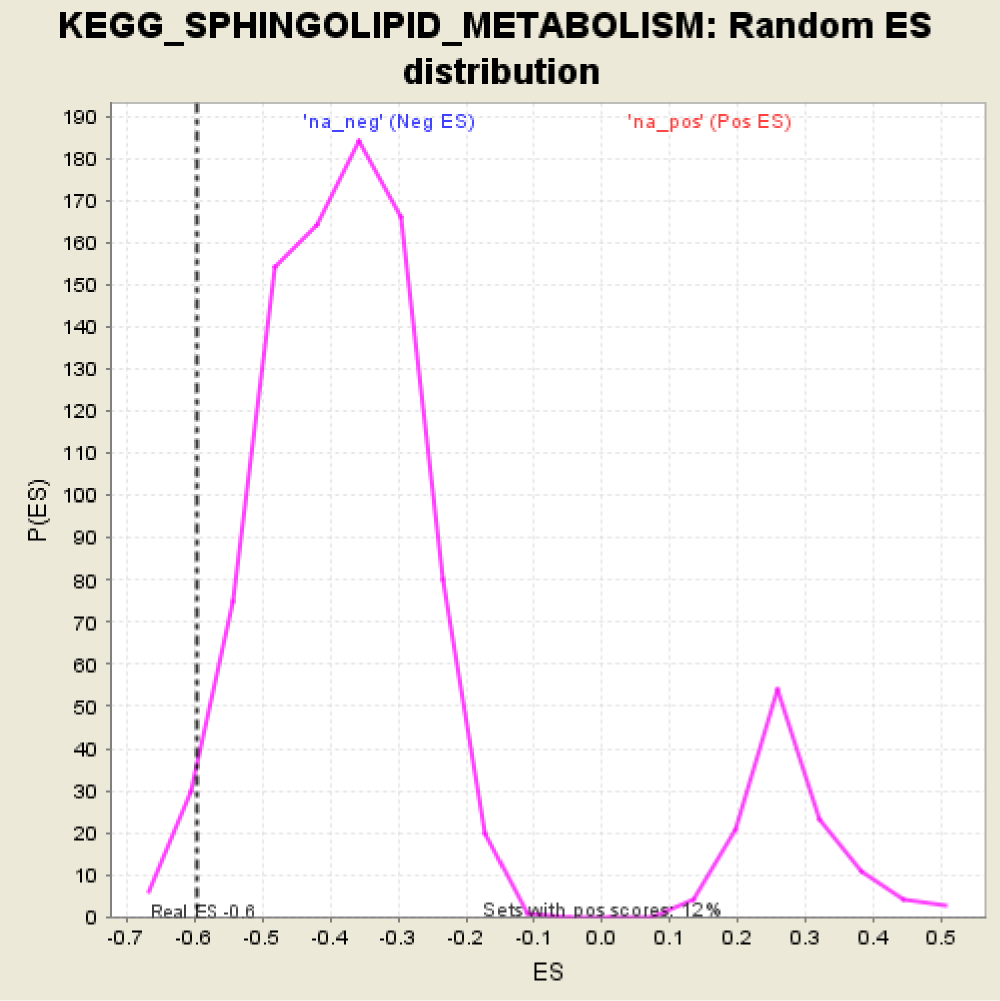

Fig 2: KEGG_SPHINGOLIPID_METABOLISM: Random ES distribution
Gene set null distribution of ES for KEGG_SPHINGOLIPID_METABOLISM

Table: GSEA Results Summary

Dataset	GSEA_18M_collapsed
Phenotype	NoPhenotypeAvailable
Upregulated in class	na_neg
GeneSet	KEGG_LONG_TERM_DEPRESSION
Enrichment Score (ES)	-0.53285664
Normalized Enrichment Score (NES)	-1.5241755
Nominal p-value	0.008368201
FDR q-value	0.11846876
FWER p-Value	0.785

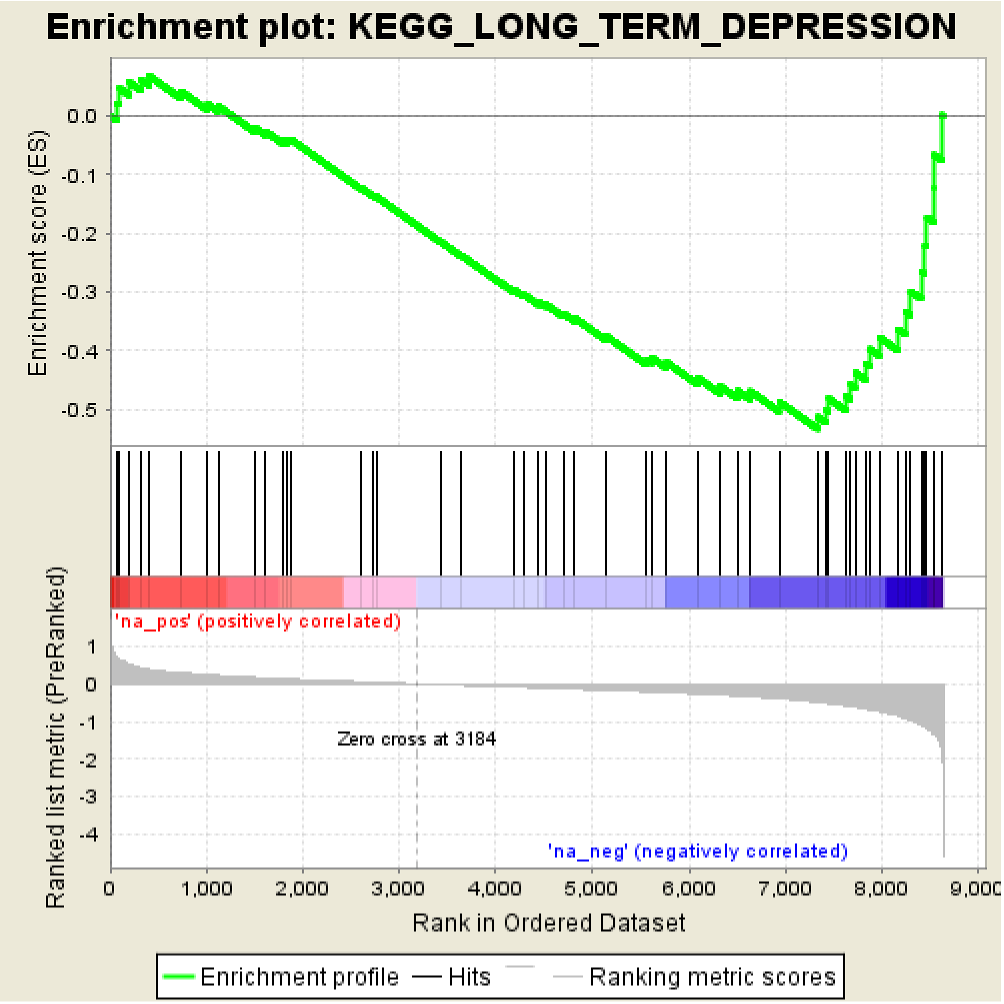

Fig 1: Enrichment plot: KEGG_LONG_TERM_DEPRESSION
Profile of the Running ES Score & Positions of GeneSet Members on the Rank Ordered List

Table: GSEA details [plain text format]

	PROBE	GENE	GENE_TITLE	RANK	RANK	RUNNING	CORE

		SYMBOL		IN GENE LIST	METRIC SCORE	ES	ENRICHMENT
1	NOS1	NOS1 Entrez, Source	nitric oxide synthase 1 (neuronal)	73	0.704	0.0214	No
2	PLA2G4B	PLA2G4B Entrez, Source	phospholipase A2, group IVB (cytosolic)	97	0.651	0.0464	No
3	RYR1	RYR1 Entrez, Source	ryanodine receptor 1 (skeletal)	198	0.523	0.0569	No
4	PLA2G2F	PLA2G2F Entrez, Source	phospholipase A2, group IIF	322	0.428	0.0608	No
5	GNAS	GNAS Entrez, Source	GNAS complex locus	403	0.384	0.0678	No
6	PLCB3	PLCB3 Entrez, Source	phospholipase C, beta 3 (phosphatidylinositol-specific)	733	0.292	0.0419	No
7	PLA2G4A	PLA2G4A Entrez, Source	phospholipase A2, group IVA (cytosolic, calcium-dependent)	1013	0.240	0.0196	No
8	IGF1R	IGF1R Entrez, Source	insulin-like growth factor 1 receptor	1122	0.222	0.0164	No
9	PLA2G4E	PLA2G4E Entrez, Source	phospholipase A2, group IVE	1495	0.171	-0.0196	No
10	ITPR2	ITPR2 Entrez, Source	inositol 1,4,5-triphosphate receptor, type 2	1615	0.156	-0.0269	No
11	RAF1	RAF1 Entrez, Source	v-raf-1 murine leukemia viral oncogene homolog 1	1794	0.134	-0.0419	No
12	PPP2R1B	PPP2R1B Entrez, Source	protein phosphatase 2 (formerly 2A), regulatory subunit A (PR 65), beta isoform	1840	0.130	-0.0416	No
13	PRKG1	PRKG1 Entrez, Source	protein kinase, cGMP-dependent, type I	1869	0.127	-0.0395	No
14	GRM1	GRM1 Entrez, Source	glutamate receptor, metabotropic 1	2606	0.052	-0.1230	No
15	GRIA2	GRIA2 Entrez, Source	glutamate receptor, ionotropic, AMPA 2	2733	0.040	-0.1360	No

16	CACNA1A	CACNA1A Entrez, Source	calcium channel, voltage-dependent, P/Q type, alpha 1A subunit	2760	0.037	-0.1374	No
17	GRIA3	GRIA3 Entrez, Source	glutamate receptor, ionotrophic, AMPA 3	3429	-0.021	-0.2143	No
18	PLCB1	PLCB1 Entrez, Source	phospholipase C, beta 1 (phosphoinositide-specific)	3646	-0.041	-0.2377	No
19	ITPR3	ITPR3 Entrez, Source	inositol 1,4,5-triphosphate receptor, type 3	4184	-0.087	-0.2966	No
20	GNAO1	GNAO1 Entrez, Source	guanine nucleotide binding protein (G protein), alpha activating activity polypeptide O	4285	-0.095	-0.3042	No
21	LYN	LYN Entrez, Source	v-yes-1 Yamaguchi sarcoma viral related oncogene homolog	4439	-0.108	-0.3175	No
22	MAPK3	MAPK3 Entrez, Source	mitogen-activated protein kinase 3	4509	-0.114	-0.3207	No
23	CRH	CRH Entrez, Source	corticotropin releasing hormone	4694	-0.129	-0.3366	No
24	NRAS	NRAS Entrez, Source	neuroblastoma RAS viral (v-ras) oncogene homolog	4809	-0.140	-0.3439	No
25	PPP2CB	PPP2CB Entrez, Source	protein phosphatase 2 (formerly 2A), catalytic subunit, beta isoform	5137	-0.170	-0.3748	No
26	MAP2K1	MAP2K1 Entrez, Source	mitogen-activated protein kinase kinase 1	5558	-0.211	-0.4148	No
27	GRIA1	GRIA1 Entrez, Source	glutamate receptor, ionotropic, AMPA 1	5612	-0.215	-0.4118	No
28	GNAI2	GNAI2 Entrez, Source	guanine nucleotide binding protein (G protein), alpha inhibiting activity polypeptide 2	5765	-0.232	-0.4197	No
29	GNAZ	GNAZ Entrez, Source	guanine nucleotide binding protein (G protein), alpha z polypeptide	6093	-0.268	-0.4464	No
30	GNAI3	GNAI3 Entrez, Source	guanine nucleotide binding protein (G protein), alpha inhibiting activity polypeptide 3	6319	-0.295	-0.4600	No
31	PLA2G12A	PLA2G12A Entrez, Source	phospholipase A2, group XIIA	6502	-0.320	-0.4677	No

32	PRKCA	PRKCA Entrez, Source	protein kinase C, alpha	6631	-0.341	-0.4681	No
33	PRKG2	PRKG2 Entrez, Source	protein kinase, cGMP-dependent, type II	6933	-0.394	-0.4864	No
34	GNAI1	GNAI1 Entrez, Source	guanine nucleotide binding protein (G protein), alpha inhibiting activity polypeptide 1	7333	-0.488	-0.5121	Yes
35	GRM5	GRM5 Entrez, Source	glutamate receptor, metabotropic 5	7423	-0.514	-0.5007	Yes
36	MAPK1	MAPK1 Entrez, Source	mitogen-activated protein kinase 1	7446	-0.522	-0.4811	Yes
37	IGF1	IGF1 Entrez, Source	insulin-like growth factor 1 (somatomedin C)	7626	-0.580	-0.4773	Yes
38	PLA2G3	PLA2G3 Entrez, Source	phospholipase A2, group III	7674	-0.598	-0.4573	Yes
39	PLA2G6	PLA2G6 Entrez, Source	phospholipase A2, group VI (cytosolic, calcium-independent)	7724	-0.616	-0.4369	Yes
40	ITPR1	ITPR1 Entrez, Source	inositol 1,4,5-triphosphate receptor, type 1	7830	-0.669	-0.4207	Yes
41	PLCB4	PLCB4 Entrez, Source	phospholipase C, beta 4	7874	-0.685	-0.3966	Yes
42	CRHR1	CRHR1 Entrez, Source	corticotropin releasing hormone receptor 1	7974	-0.729	-0.3772	Yes
43	ARAF	ARAF Entrez, Source	v-raf murine sarcoma 3611 viral oncogene homolog	8164	-0.845	-0.3633	Yes
44	GUCY1B3	GUCY1B3 Entrez, Source	guanylate cyclase 1, soluble, beta 3	8244	-0.915	-0.3336	Yes
45	BRAF	BRAF Entrez, Source	v-raf murine sarcoma viral oncogene homolog B1	8297	-0.967	-0.2986	Yes
46	GNA12	GNA12 Entrez, Source	guanine nucleotide binding protein (G protein) alpha 12	8409	-1.090	-0.2652	Yes
47	KRAS	KRAS Entrez, Source	v-Ki-ras2 Kirsten rat sarcoma viral oncogene homolog	8444	-1.153	-0.2202	Yes

48	GUCY1A3	GUCY1A3 Entrez, Source	guanylate cyclase 1, soluble, alpha 3	8465	-1.181	-0.1724	Yes
49	GNA13	GNA13 Entrez, Source	guanine nucleotide binding protein (G protein), alpha 13	8538	-1.347	-0.1235	Yes
50	GNAQ	GNAQ Entrez, Source	guanine nucleotide binding protein (G protein), q polypeptide	8542	-1.352	-0.0665	Yes
51	GRID2	GRID2 Entrez, Source	glutamate receptor, ionotropic, delta 2	8624	-1.822	0.0015	Yes

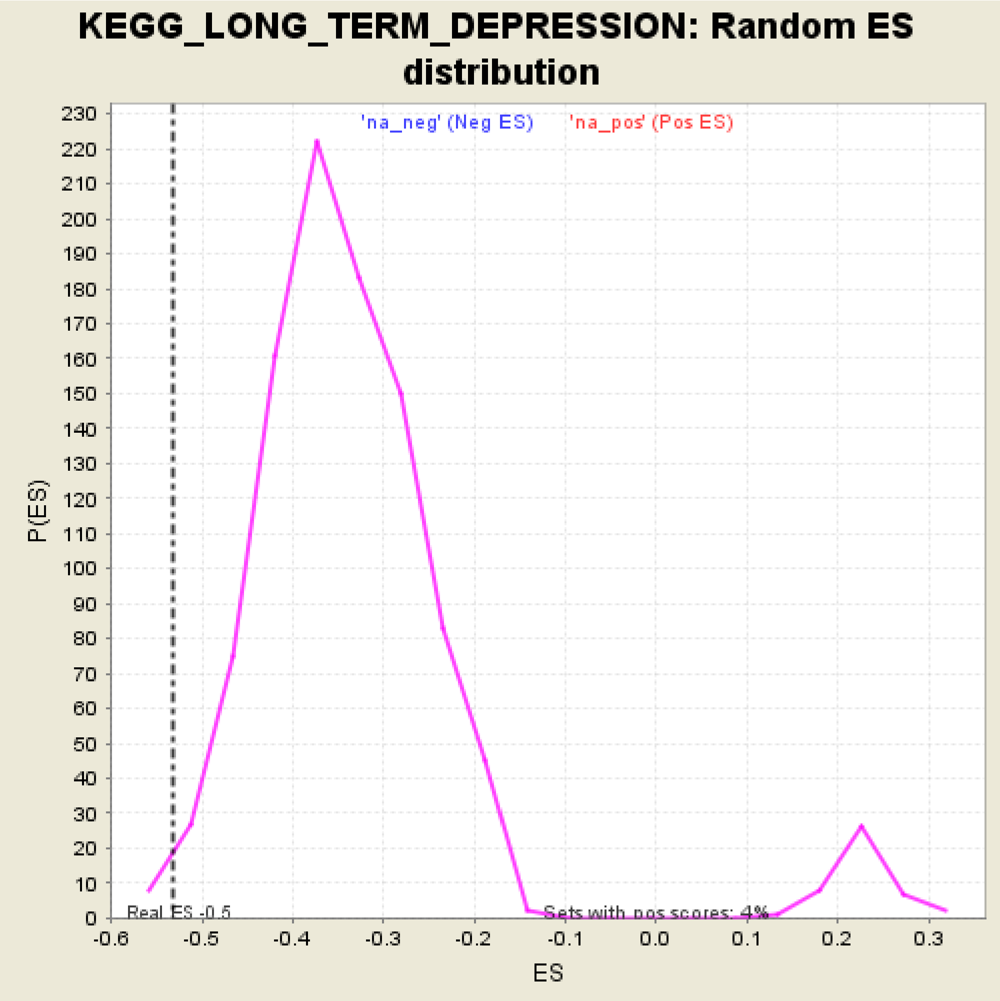

Fig 2: KEGG_LONG_TERM_DEPRESSION: Random ES distribution
Gene set null distribution of ES for KEGG_LONG_TERM_DEPRESSION

Table: GSEA Results Summary

Dataset	GSEA_18M_collapsed
Phenotype	NoPhenotypeAvailable
Upregulated in class	na_neg
GeneSet	REACTOME_RECYCLING_PATHWAY_OF_L1
Enrichment Score (ES)	-0.7328634
Normalized Enrichment Score (NES)	-1.8283837
Nominal p-value	0.0011235955
FDR q-value	0.030726112
FWER p-Value	0.029

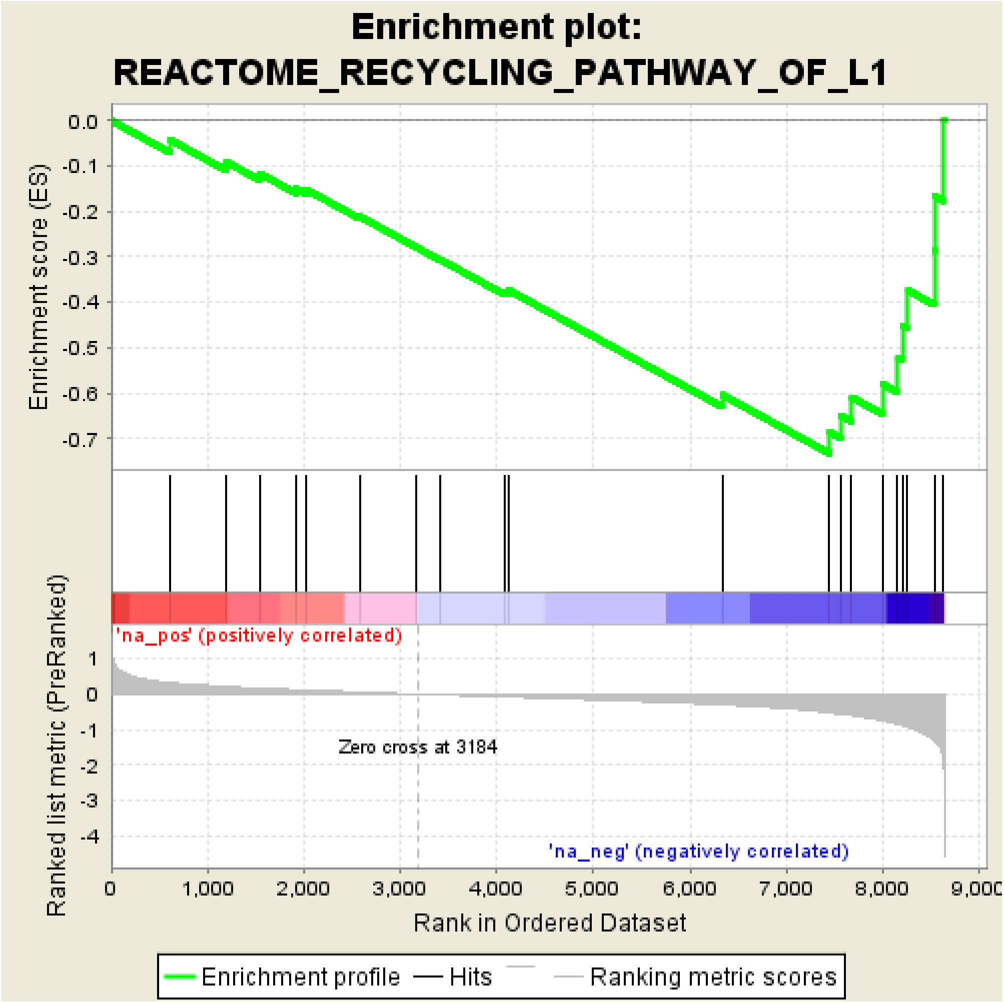

Fig 1: Enrichment plot: REACTOME_RECYCLING_PATHWAY_OF_L1
Profile of the Running ES Score & Positions of GeneSet Members on the Rank Ordered List

Table: GSEA details [plain text format]

	PROBE	GENE	GENE_TITLE	RANK	RANK	RUNNING	CORE

		SYMBOL		IN GENE LIST	METRIC SCORE	ES	ENRICHMENT
1	DPYSL2	DPYSL2 Entrez, Source	dihydropyrimidinase-like 2	610	0.319	-0.0423	No
2	CLTC	CLTC Entrez, Source	clathrin, heavy chain (Hc)	1194	0.211	-0.0911	No
3	RPS6KA1	RPS6KA1 Entrez, Source	ribosomal protein S6 kinase, 90kDa, polypeptide 1	1540	0.165	-0.1164	No
4	AP2A1	AP2A1 Entrez, Source	adaptor-related protein complex 2, alpha 1 subunit	1919	0.121	-0.1495	No
5	CLTA	CLTA Entrez, Source	clathrin, light chain (Lca)	2024	0.110	-0.1518	No
6	RPS6KA4	RPS6KA4 Entrez, Source	ribosomal protein S6 kinase, 90kDa, polypeptide 4	2576	0.055	-0.2108	No
7	RPS6KA2	RPS6KA2 Entrez, Source	ribosomal protein S6 kinase, 90kDa, polypeptide 2	3164	0.002	-0.2788	No
8	NUMB	NUMB Entrez, Source	numb homolog (Drosophila)	3403	-0.019	-0.3047	No
9	RDX	RDX Entrez, Source	radixin	4080	-0.077	-0.3762	No
10	AP2M1	AP2M1 Entrez, Source	adaptor-related protein complex 2, mu 1 subunit	4116	-0.081	-0.3731	No
11	DNM2	DNM2 Entrez, Source	dynamin 2	6331	-0.296	-0.6036	No
12	MAPK1	MAPK1 Entrez, Source	mitogen-activated protein kinase 1	7446	-0.522	-0.6863	Yes
13	RPS6KA3	RPS6KA3 Entrez, Source	ribosomal protein S6 kinase, 90kDa, polypeptide 3	7559	-0.558	-0.6495	Yes
14	RPS6KA6	RPS6KA6 Entrez, Source	ribosomal protein S6 kinase, 90kDa, polypeptide 6	7676	-0.599	-0.6094	Yes
15	DNM1	DNM1 Entrez, Source	dynamin 1	7993	-0.744	-0.5797	Yes
16	MSN	MSN Entrez, Source	moesin	8155	-0.837	-0.5237	Yes

17	L1CAM	L1CAM Entrez, Source	L1 cell adhesion molecule	8199	-0.881	-0.4501	Yes
18	AP2A2	AP2A2 Entrez, Source	adaptor-related protein complex 2, alpha 2 subunit	8259	-0.929	-0.3740	Yes
19	SH3GL2	SH3GL2 Entrez, Source	SH3-domain GRB2-like 2	8533	-1.335	-0.2865	Yes
20	AP2B1	AP2B1 Entrez, Source	adaptor-related protein complex 2, beta 1 subunit	8540	-1.348	-0.1670	Yes
21	RPS6KA5	RPS6KA5 Entrez, Source	ribosomal protein S6 kinase, 90kDa, polypeptide 5	8631	-1.996	0.0007	Yes

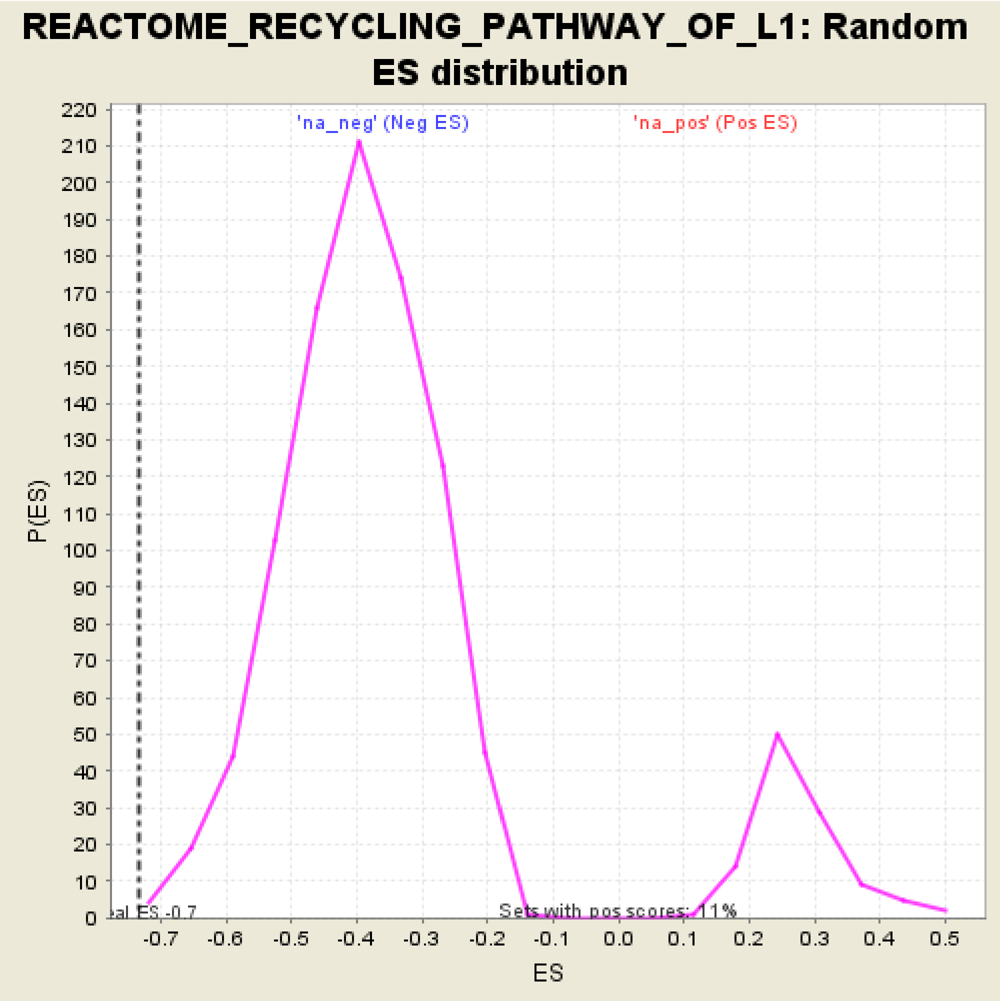

Fig 2: REACTOME_RECYCLING_PATHWAY_OF_L1: Random ES distribution
Gene set null distribution of ES for REACTOME_RECYCLING_PATHWAY_OF_L1

Table: GSEA Results Summary

Dataset	GSEA_18M_collapsed
Phenotype	NoPhenotypeAvailable
Upregulated in class	na_neg
GeneSet	REACTOME_SIGNAL_TRANSDUCTION_BY_L1
Enrichment Score (ES)	-0.6515924
Normalized Enrichment Score (NES)	-1.7733538
Nominal p-value	0.0
FDR q-value	0.049035944
FWER p-Value	0.092

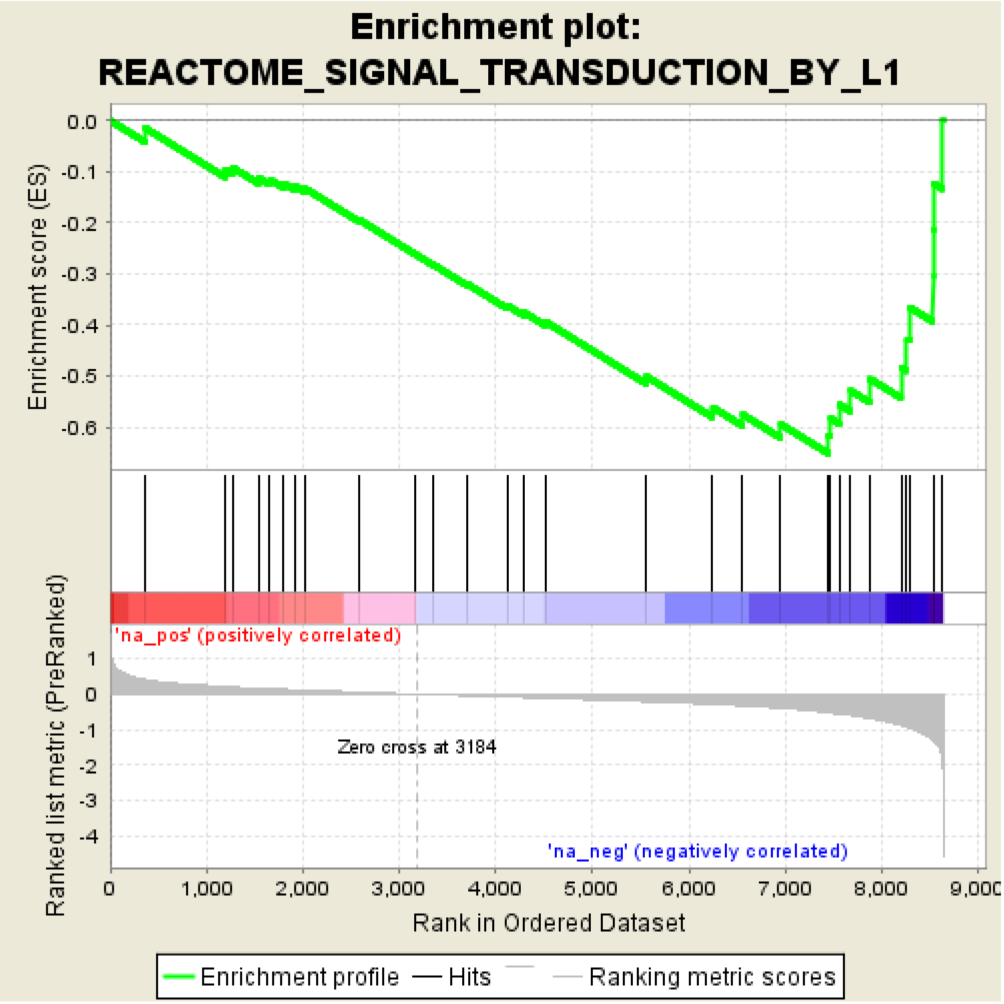

Fig 1: Enrichment plot: REACTOME_SIGNAL_TRANSDUCTION_BY_L1 Profile of the Running ES Score & Positions of GeneSet Members on the Rank Ordered List

Table: GSEA details [plain text format]

	PROBE	GENE	GENE_TITLE	RANK	RANK	RUNNING	CORE

		SYMBOL		IN GENE LIST	METRIC SCORE	ES	ENRICHMENT
1	CSNK2A1	CSNK2A1 Entrez, Source	casein kinase 2, alpha 1 polypeptide	364	0.408	-0.0147	No
2	CLTC	CLTC Entrez, Source	clathrin, heavy chain (Hc)	1194	0.211	-0.0968	No
3	NRP1	NRP1 Entrez, Source	neuropilin 1	1269	0.199	-0.0919	No
4	RPS6KA1	RPS6KA1 Entrez, Source	ribosomal protein S6 kinase, 90kDa, polypeptide 1	1540	0.165	-0.1121	No
5	ITGA5	ITGA5 Entrez, Source	integrin, alpha 5 (fibronectin receptor, alpha polypeptide)	1658	0.151	-0.1155	No
6	ITGA2B	ITGA2B Entrez, Source	integrin, alpha 2b (platelet glycoprotein IIb of IIb/IIIa complex, antigen CD41)	1795	0.134	-0.1222	No
7	AP2A1	AP2A1 Entrez, Source	adaptor-related protein complex 2, alpha 1 subunit	1919	0.121	-0.1283	No
8	CLTA	CLTA Entrez, Source	clathrin, light chain (Lca)	2024	0.110	-0.1330	No
9	RPS6KA4	RPS6KA4 Entrez, Source	ribosomal protein S6 kinase, 90kDa, polypeptide 4	2576	0.055	-0.1933	No
10	RPS6KA2	RPS6KA2 Entrez, Source	ribosomal protein S6 kinase, 90kDa, polypeptide 2	3164	0.002	-0.2614	No
11	EGFR	EGFR Entrez, Source	epidermal growth factor receptor (erythroblastic leukemia viral (v-erb-b) oncogene homolog, avian)	3352	-0.015	-0.2821	No
12	ITGA9	ITGA9 Entrez, Source	integrin, alpha 9	3707	-0.046	-0.3201	No
13	AP2M1	AP2M1 Entrez, Source	adaptor-related protein complex 2, mu 1 subunit	4116	-0.081	-0.3621	No
14	NCAM1	NCAM1 Entrez, Source	neural cell adhesion molecule 1	4284	-0.095	-0.3751	No
15	MAPK3	MAPK3 Entrez, Source	mitogen-activated protein kinase 3	4509	-0.114	-0.3934	No

16	MAP2K1	MAP2K1 Entrez, Source	mitogen-activated protein kinase kinase 1	5558	-0.211	-0.5010	No
17	FGFR1	FGFR1 Entrez, Source	fibroblast growth factor receptor 1 (fms-related tyrosine kinase 2, Pfeiffer syndrome)	6245	-0.286	-0.5614	No
18	CSNK2B	CSNK2B Entrez, Source	casein kinase 2, beta polypeptide	6556	-0.328	-0.5752	No
19	ITGB1	ITGB1 Entrez, Source	integrin, beta 1 (fibronectin receptor, beta polypeptide, antigen CD29 includes MDF2, MSK12)	6950	-0.397	-0.5941	No
20	MAPK1	MAPK1 Entrez, Source	mitogen-activated protein kinase 1	7446	-0.522	-0.6163	Yes
21	CSNK2A2	CSNK2A2 Entrez, Source	casein kinase 2, alpha prime polypeptide	7468	-0.530	-0.5829	Yes
22	RPS6KA3	RPS6KA3 Entrez, Source	ribosomal protein S6 kinase, 90kDa, polypeptide 3	7559	-0.558	-0.5557	Yes
23	RPS6KA6	RPS6KA6 Entrez, Source	ribosomal protein S6 kinase, 90kDa, polypeptide 6	7676	-0.599	-0.5287	Yes
24	PAK1	PAK1 Entrez, Source	p21/Cdc42/Rac1-activated kinase 1 (STE20 homolog, yeast)	7877	-0.686	-0.5055	Yes
25	L1CAM	L1CAM Entrez, Source	L1 cell adhesion molecule	8199	-0.881	-0.4833	Yes
26	AP2A2	AP2A2 Entrez, Source	adaptor-related protein complex 2, alpha 2 subunit	8259	-0.929	-0.4274	Yes
27	RAC1	RAC1 Entrez, Source	ras-related C3 botulinum toxin substrate 1 (rho family, small GTP binding protein Rac1)	8293	-0.964	-0.3661	Yes
28	ITGAV	ITGAV Entrez, Source	integrin, alpha V (vitronectin receptor, alpha polypeptide, antigen CD51)	8530	-1.325	-0.3040	Yes
29	SH3GL2	SH3GL2 Entrez, Source	SH3-domain GRB2-like 2	8533	-1.335	-0.2140	Yes
30	AP2B1	AP2B1 Entrez, Source	adaptor-related protein complex 2, beta 1 subunit	8540	-1.348	-0.1237	Yes
31	RPS6KA5	RPS6KA5 Entrez, Source	ribosomal protein S6 kinase, 90kDa, polypeptide 5	8631	-1.996	0.0007	Yes

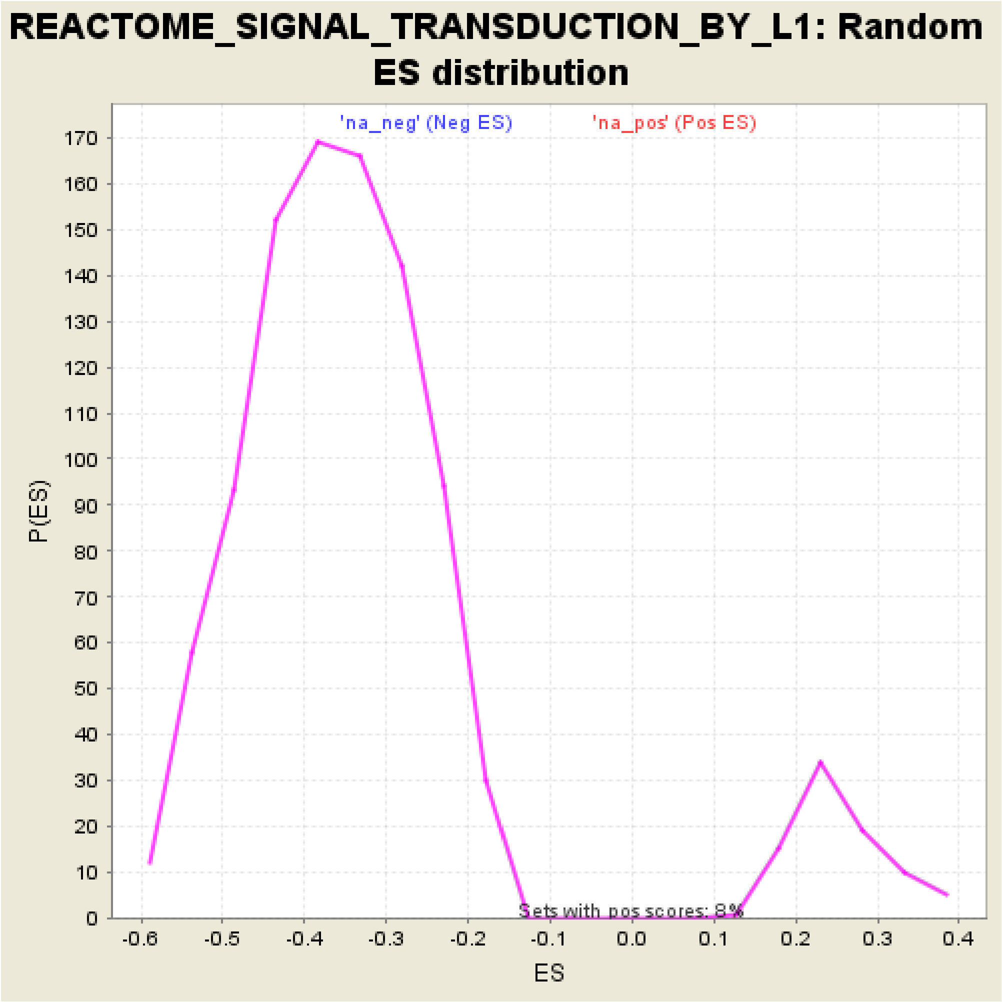

Fig 2: REACTOME_SIGNAL_TRANSDUCTION_BY_L1: Random ES distribution
Gene set null distribution of ES for REACTOME_SIGNAL_TRANSDUCTION_BY_L1

Table: GSEA Results Summary

Dataset	GSEA_18M_collapsed
Phenotype	NoPhenotypeAvailable
Upregulated in class	na_neg
GeneSet	REACTOME_ANTIVIRAL_MECHANISM_BY_IFN_STIMULATED_GENES
Enrichment Score (ES)	-0.6171727
Normalized Enrichment Score (NES)	-1.7333592
Nominal p-value	0.0010695187
FDR q-value	0.071224384
FWER p-Value	0.187

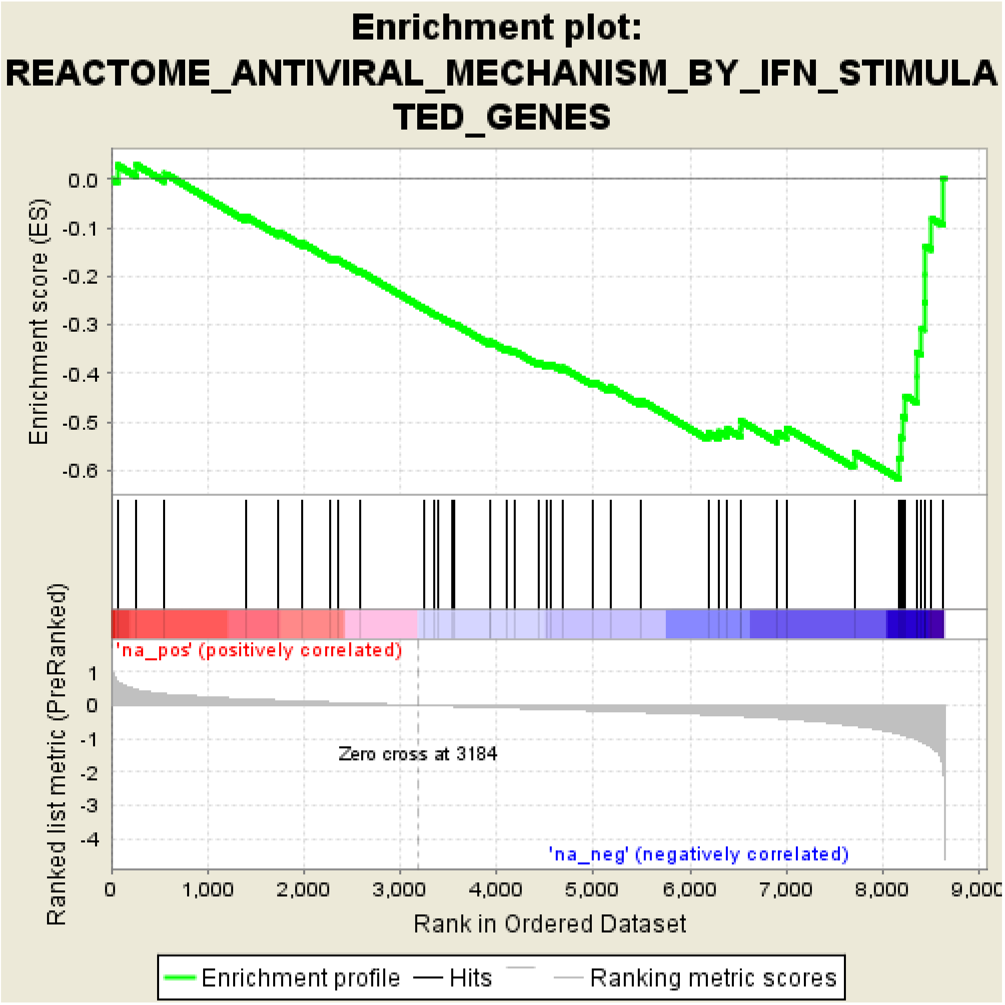

Fig 1: Enrichment plot: REACTOME_ANTIVIRAL_MECHANISM_BY_IFN_STIMULATED_GENES Profile of the Running ES Score & Positions of GeneSet Members on the Rank Ordered List

Table: GSEA details [plain text format]

	PROBE	GENE SYMBOL	GENE_TITLE	RANK IN GENE LIST	RANK METRIC SCORE	RUNNING ES	CORE ENRICHMENT
1	NUP85	NUP85 Entrez, Source	nucleoporin 85kDa	65	0.719	0.0290	No
2	FLNB	FLNB Entrez, Source	filamin B, beta (actin binding protein 278)	263	0.460	0.0294	No
3	IRF3	IRF3 Entrez, Source	interferon regulatory factor 3	558	0.331	0.0120	No
4	NUP210	NUP210 Entrez, Source	nucleoporin 210kDa	1396	0.182	-0.0761	No
5	EIF2AK2	EIF2AK2 Entrez, Source	eukaryotic translation initiation factor 2-alpha kinase 2	1740	0.141	-0.1089	No
6	EIF4E3	EIF4E3 Entrez, Source	eukaryotic translation initiation factor 4E member 3	1983	0.114	-0.1312	No
7	NUP188	NUP188 Entrez, Source	nucleoporin 188kDa	2280	0.085	-0.1614	No
8	NUP43	NUP43 Entrez, Source	nucleoporin 43kDa	2347	0.079	-0.1651	No
9	RAE1	RAE1 Entrez, Source	RAE1 RNA export 1 homolog (S. pombe)	2583	0.055	-0.1896	No
10	IFIT1	IFIT1 Entrez, Source	interferon-induced protein with tetratricopeptide repeats 1	3247	-0.006	-0.2665	No
11	TPR	TPR Entrez, Source	translocated promoter region (to activated MET oncogene)	3356	-0.015	-0.2783	No
12	EIF4G3	EIF4G3 Entrez, Source	eukaryotic translation initiation factor 4 gamma, 3	3402	-0.019	-0.2826	No
13	AAAS	AAAS Entrez, Source	achalasia, adrenocortical insufficiency, alacrimia (Allgrove, triple-A)	3532	-0.029	-0.2961	No
14	RANBP2	RANBP2 Entrez, Source	RAN binding protein 2	3558	-0.032	-0.2974	No
15	EIF4G2	EIF4G2 Entrez, Source	eukaryotic translation initiation factor 4 gamma, 2	3929	-0.064	-0.3372	No

16	NUP153	NUP153 Entrez, Source	nucleoporin 153kDa	3931	-0.064	-0.3341	No
17	USP18	USP18 Entrez, Source	ubiquitin specific peptidase 18	4105	-0.080	-0.3501	No
18	PIN1	PIN1 Entrez, Source	protein (peptidylprolyl cis/trans isomerase) NIMA-interacting 1	4183	-0.087	-0.3547	No
19	NUP62	NUP62 Entrez, Source	nucleoporin 62kDa	4427	-0.107	-0.3775	No
20	MAPK3	MAPK3 Entrez, Source	mitogen-activated protein kinase 3	4509	-0.114	-0.3812	No
21	TRIM25	TRIM25 Entrez, Source	tripartite motif-containing 25	4556	-0.117	-0.3806	No
22	HERC5	HERC5 Entrez, Source	hect domain and RLD 5	4674	-0.128	-0.3877	No
23	UBE2N	UBE2N Entrez, Source	ubiquitin-conjugating enzyme E2N (UBC13 homolog, yeast)	4997	-0.157	-0.4172	No
24	NUP205	NUP205 Entrez, Source	nucleoporin 205kDa	5172	-0.173	-0.4286	No
25	NUP214	NUP214 Entrez, Source	nucleoporin 214kDa	5488	-0.204	-0.4549	No
26	NUP93	NUP93 Entrez, Source	nucleoporin 93kDa	6187	-0.278	-0.5220	No
27	EIF4E2	EIF4E2 Entrez, Source	eukaryotic translation initiation factor 4E member 2	6298	-0.292	-0.5200	No
28	NUP54	NUP54 Entrez, Source	nucleoporin 54kDa	6387	-0.303	-0.5148	No
29	NUP50	NUP50 Entrez, Source	nucleoporin 50kDa	6523	-0.323	-0.5141	No
30	KPNA2	KPNA2 Entrez, Source	karyopherin alpha 2 (RAG cohort 1, importin alpha 1)	6526	-0.323	-0.4979	No
31	EIF4E	EIF4E Entrez, Source	eukaryotic translation initiation factor 4E	6901	-0.388	-0.5217	No
32	STAT1	STAT1 Entrez, Source	signal transducer and activator of transcription 1, 91kDa	6998	-0.406	-0.5122	No

33	NUP133	NUP133 Entrez, Source	nucleoporin 133kDa	7701	-0.608	-0.5629	No
34	NUPL1	NUPL1 Entrez, Source	nucleoporin like 1	8168	-0.851	-0.5739	Yes
35	EIF4G1	EIF4G1 Entrez, Source	eukaryotic translation initiation factor 4 gamma, 1	8184	-0.864	-0.5318	Yes
36	SEH1L	SEH1L Entrez, Source	SEH1-like (S. cerevisiae)	8202	-0.884	-0.4888	Yes
37	JAK1	JAK1 Entrez, Source	Janus kinase 1 (a protein tyrosine kinase)	8234	-0.908	-0.4463	Yes
38	NEDD4	NEDD4 Entrez, Source	neural precursor cell expressed, developmentally down-regulated 4	8350	-1.020	-0.4078	Yes
39	KPNA1	KPNA1 Entrez, Source	karyopherin alpha 1 (importin alpha 5)	8352	-1.020	-0.3561	Yes
40	EIF4A1	EIF4A1 Entrez, Source	eukaryotic translation initiation factor 4A, isoform 1	8402	-1.078	-0.3070	Yes
41	PPM1B	PPM1B Entrez, Source	protein phosphatase 1B (formerly 2C), magnesium-dependent, beta isoform	8427	-1.116	-0.2530	Yes
42	ARIH1	ARIH1 Entrez, Source	ariadne homolog, ubiquitin-conjugating enzyme E2 binding protein, 1 (Drosophila)	8440	-1.150	-0.1960	Yes
43	KPNA3	KPNA3 Entrez, Source	karyopherin alpha 3 (importin alpha 4)	8442	-1.152	-0.1375	Yes
44	KPNA4	KPNA4 Entrez, Source	karyopherin alpha 4 (importin alpha 3)	8508	-1.248	-0.0816	Yes
45	KPNB1	KPNB1 Entrez, Source	karyopherin (importin) beta 1	8626	-1.899	0.0013	Yes

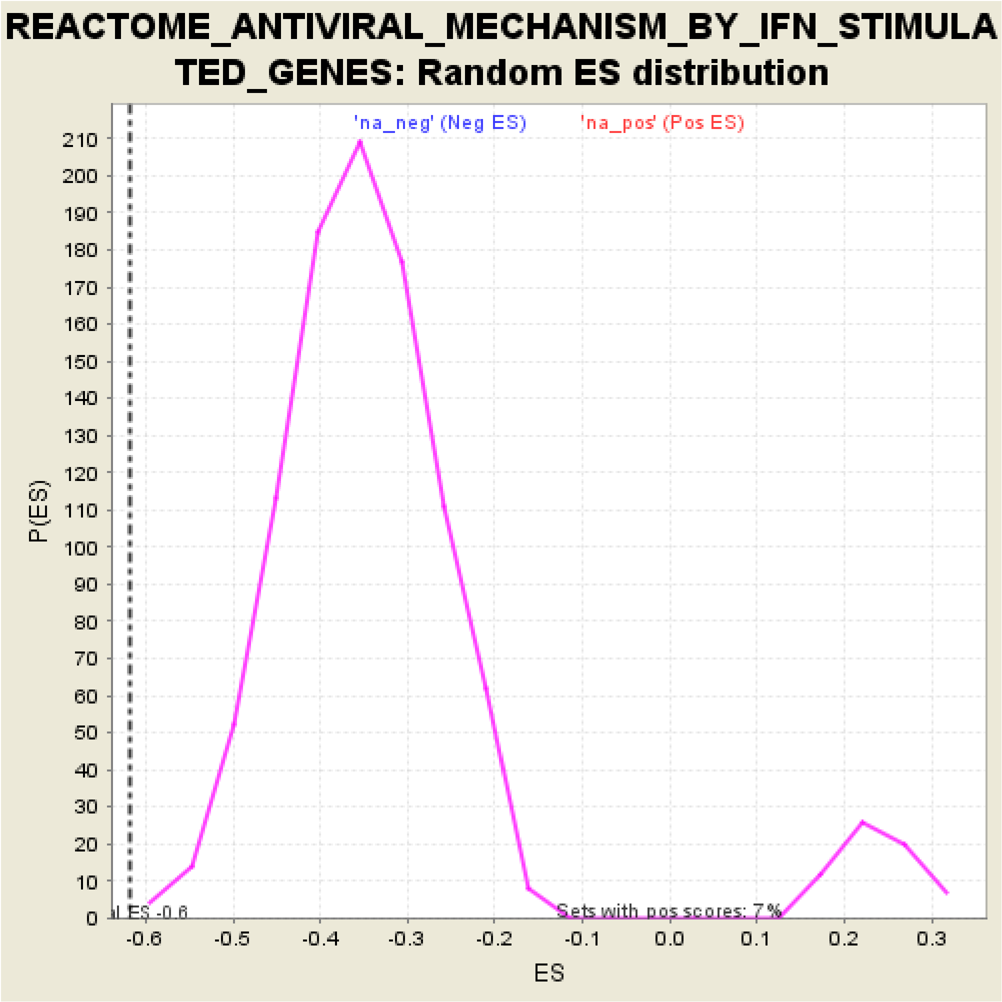

Fig 2: REACTOME_ANTIVIRAL_MECHANISM_BY_IFN_STIMULATED_GENES: Random ES distribution
Gene set null distribution of ES for REACTOME_ANTIVIRAL_MECHANISM_BY_IFN_STIMULATED_GENES

Table: GSEA Results Summary

Dataset	GSEA_18M_collapsed
Phenotype	NoPhenotypeAvailable
Upregulated in class	na_neg
GeneSet	REACTOME_EGFR_DOWNREGULATION
Enrichment Score (ES)	-0.71340793
Normalized Enrichment Score (NES)	-1.724851
Nominal p-value	0.0023364485
FDR q-value	0.06289053
FWER p-Value	0.217

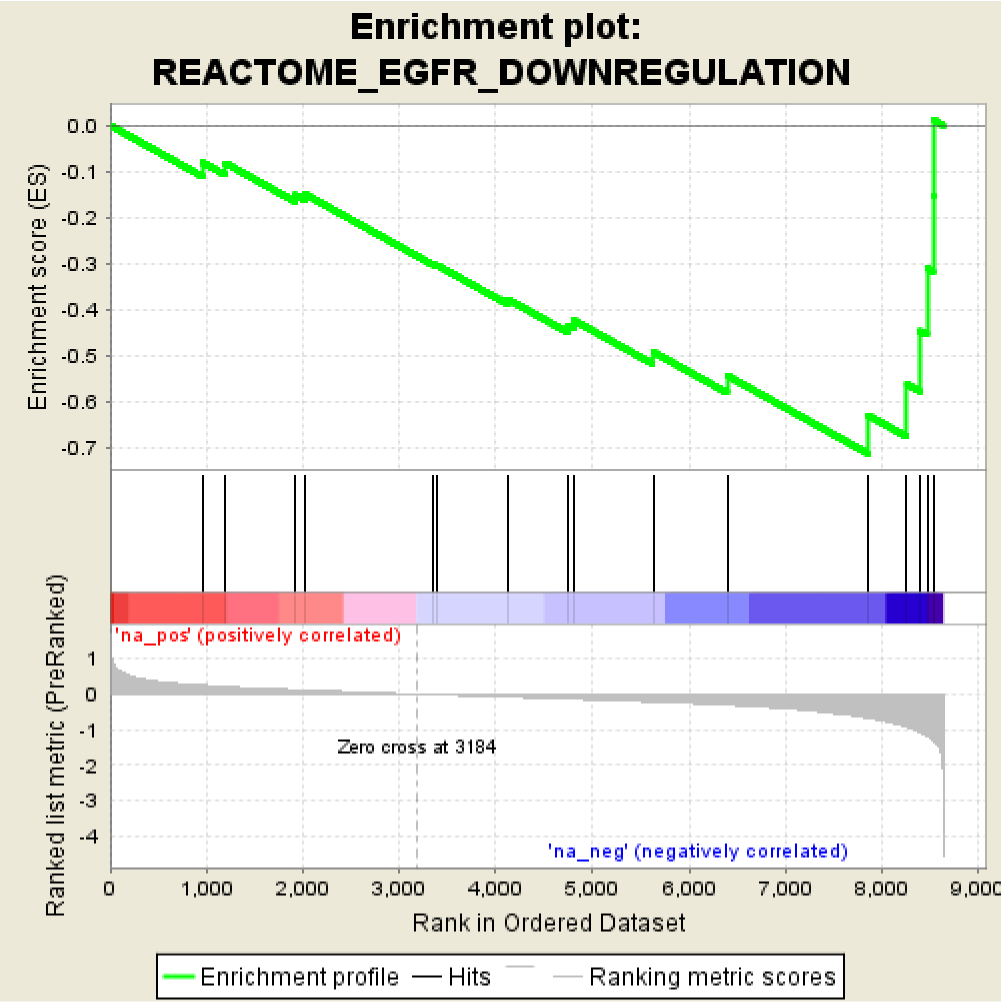

Fig 1: Enrichment plot: REACTOME_EGFR_DOWNREGULATION
Profile of the Running ES Score & Positions of GeneSet Members on the Rank Ordered List

Table: GSEA details [plain text format]

	PROBE	GENE	GENE_TITLE	RANK	RANK	RUNNING	CORE

		SYMBOL		IN GENE LIST	METRIC SCORE	ES	ENRICHMENT
1	EPS15L1	EPS15L1 Entrez, Source	epidermal growth factor receptor pathway substrate 15-like 1	960	0.250	-0.0807	No
2	CLTC	CLTC Entrez, Source	clathrin, heavy chain (Hc)	1194	0.211	-0.0818	No
3	AP2A1	AP2A1 Entrez, Source	adaptor-related protein complex 2, alpha 1 subunit	1919	0.121	-0.1509	No
4	CLTA	CLTA Entrez, Source	clathrin, light chain (Lca)	2024	0.110	-0.1495	No
5	EGFR	EGFR Entrez, Source	epidermal growth factor receptor (erythroblastic leukemia viral (v-erb-b) oncogene homolog, avian)	3352	-0.015	-0.3016	No
6	EPN1	EPN1 Entrez, Source	epsin 1	3398	-0.019	-0.3045	No
7	AP2M1	AP2M1 Entrez, Source	adaptor-related protein complex 2, mu 1 subunit	4116	-0.081	-0.3778	No
8	GRB2	GRB2 Entrez, Source	growth factor receptor-bound protein 2	4744	-0.134	-0.4341	No
9	SPRY2	SPRY2 Entrez, Source	sprouty homolog 2 (Drosophila)	4802	-0.139	-0.4236	No
10	STAM	STAM Entrez, Source	signal transducing adaptor molecule (SH3 domain and ITAM motif) 1	5624	-0.217	-0.4923	No
11	STAM2	STAM2 Entrez, Source	signal transducing adaptor molecule (SH3 domain and ITAM motif) 2	6394	-0.305	-0.5441	No
12	SH3KBP1	SH3KBP1 Entrez, Source	SH3-domain kinase binding protein 1	7855	-0.678	-0.6301	Yes
13	AP2A2	AP2A2 Entrez, Source	adaptor-related protein complex 2, alpha 2 subunit	8259	-0.929	-0.5628	Yes
14	CBL	CBL Entrez, Source	Cas-Br-M (murine) ecotropic retroviral transforming sequence	8393	-1.070	-0.4468	Yes
15	EPS15	EPS15 Entrez, Source	epidermal growth factor receptor pathway substrate 15	8469	-1.184	-0.3101	Yes

16	SH3GL2	SH3GL2 Entrez, Source	SH3-domain GRB2-like 2	8533	-1.335	-0.1535	Yes
17	AP2B1	AP2B1 Entrez, Source	adaptor-related protein complex 2, beta 1 subunit	8540	-1.348	0.0113	Yes

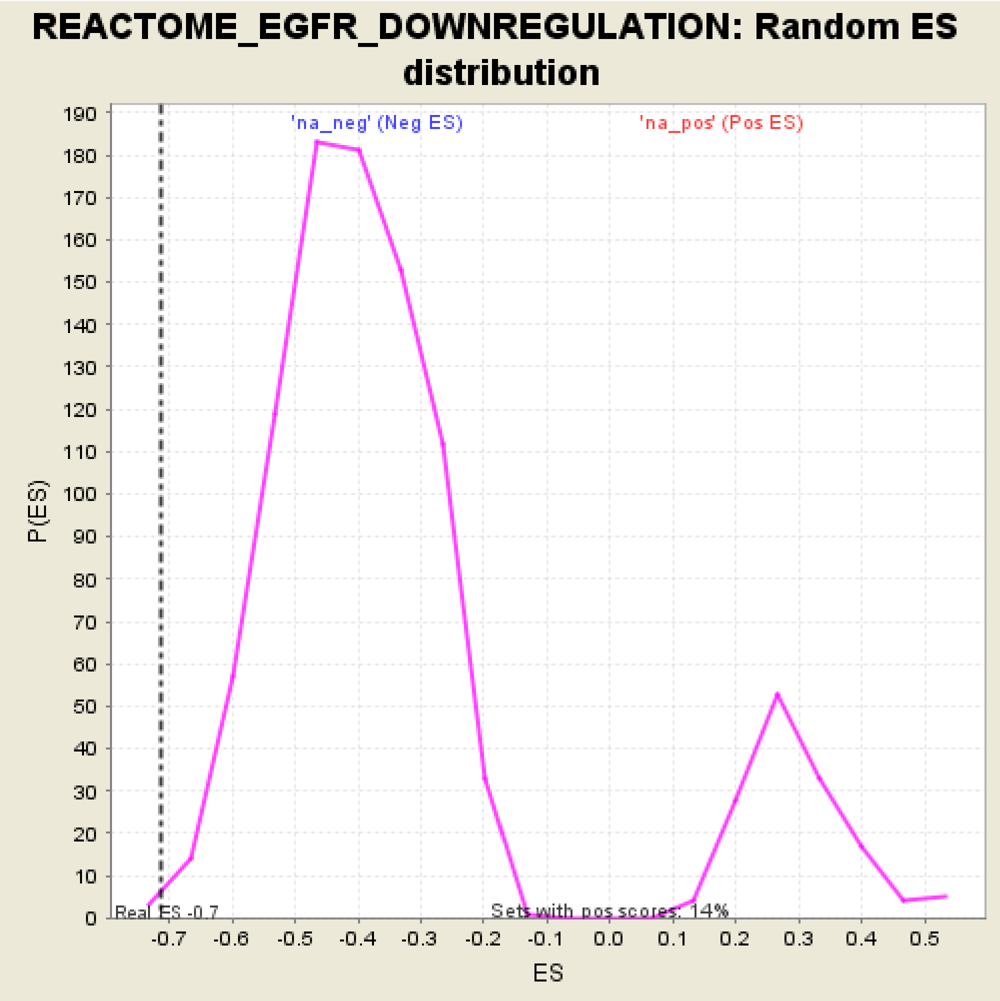

Fig 2: REACTOME_EGFR_DOWNREGULATION: Random ES distribution
Gene set null distribution of ES for REACTOME_EGFR_DOWNREGULATION

Table: GSEA Results Summary

Dataset	GSEA_18M_collapsed
Phenotype	NoPhenotypeAvailable
Upregulated in class	na_neg
GeneSet	REACTOME_GABA_SYNTHESIS_RELEASE_REUPTAKE_AND_DEGRADATION
Enrichment Score (ES)	-0.7022085
Normalized Enrichment Score (NES)	-1.6867505
Nominal p-value	0.0023923444
FDR q-value	0.09922064
FWER p-Value	0.384

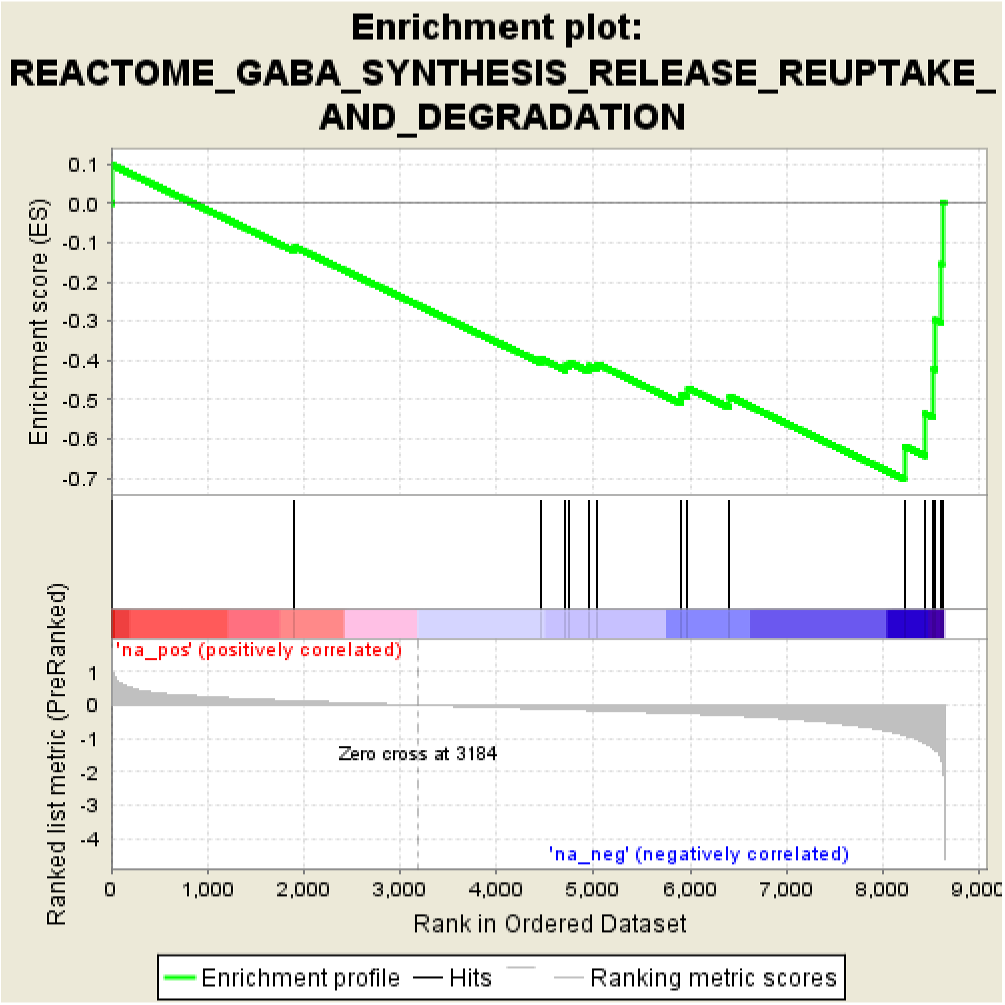

Fig 1: Enrichment plot: REACTOME_GABA_SYNTHESIS_RELEASE_REUPTAKE_AND_DEGRADATION Profile of the Running ES Score & Positions of GeneSet Members on the Rank Ordered List

Table: GSEA details [plain text format]

	PROBE	GENE SYMBOL	GENE_TITLE	RANK IN GENE LIST	RANK METRIC SCORE	RUNNING ES	CORE ENRICHMENT
1	SLC6A1	SLC6A1 Entrez, Source	solute carrier family 6 (neurotransmitter transporter, GABA), member 1	13	1.049	0.0968	No
2	HSPA8	HSPA8 Entrez, Source	heat shock 70kDa protein 8	1905	0.122	-0.1110	No
3	STX1A	STX1A Entrez, Source	syntaxin 1A (brain)	4451	-0.109	-0.3960	No
4	SLC6A11	SLC6A11 Entrez, Source	solute carrier family 6 (neurotransmitter transporter, GABA), member 11	4708	-0.130	-0.4135	No
5	ALDH5A1	ALDH5A1 Entrez, Source	aldehyde dehydrogenase 5 family, member A1 (succinate-semialdehyde dehydrogenase)	4750	-0.134	-0.4056	No
6	RIMS1	RIMS1 Entrez, Source	regulating synaptic membrane exocytosis 1	4945	-0.153	-0.4138	No
7	SLC32A1	SLC32A1 Entrez, Source	solute carrier family 32 (GABA vesicular transporter), member 1	5034	-0.160	-0.4090	No
8	ABAT	ABAT Entrez, Source	4-aminobutyrate aminotransferase	5908	-0.247	-0.4871	No
9	SLC6A12	SLC6A12 Entrez, Source	solute carrier family 6 (neurotransmitter transporter, betaine/GABA), member 12	5976	-0.256	-0.4709	No
10	SNAP25	SNAP25 Entrez, Source	synaptosomal-associated protein, 25kDa	6408	-0.306	-0.4922	No
11	GAD2	GAD2 Entrez, Source	glutamate decarboxylase 2 (pancreatic islets and brain, 65kDa)	8220	-0.898	-0.6181	Yes
12	GAD1	GAD1 Entrez, Source	glutamate decarboxylase 1 (brain, 67kDa)	8441	-1.150	-0.5358	Yes
13	VAMP2	VAMP2 Entrez, Source	vesicle-associated membrane protein 2 (synaptobrevin 2)	8526	-1.314	-0.4223	Yes
14	DNAJC5	DNAJC5 Entrez, Source	DnaJ (Hsp40) homolog, subfamily C, member 5	8545	-1.363	-0.2966	Yes
15	STXBP1	STXBP1 Entrez, Source	syntaxin binding protein 1	8609	-1.599	-0.1541	Yes

16	SYT1	SYT1 Entrez, Source	synaptotagmin I	8615	-1.677	0.0026	Yes

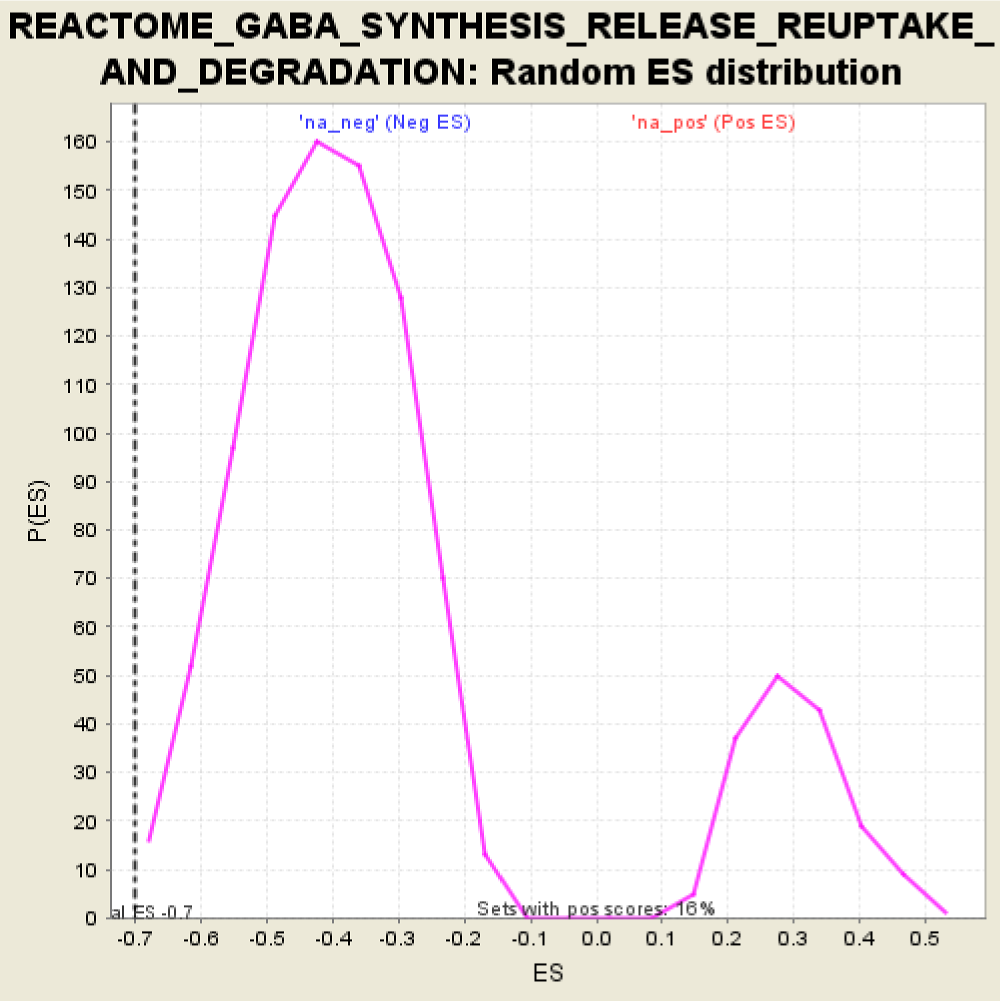

Fig 2: REACTOME_GABA_SYNTHESIS_RELEASE_REUPTAKE_AND_DEGRADATION: Random ES distribution
Gene set null distribution of ES for REACTOME_GABA_SYNTHESIS_RELEASE_REUPTAKE_AND_DEGRADATION

Table: GSEA Results Summary

Dataset	GSEA_18M_collapsed
Phenotype	NoPhenotypeAvailable
Upregulated in class	na_neg
GeneSet	REACTOME_NEUROTRANSMITTER_RELEASE_CYCLE
Enrichment Score (ES)	-0.6173815
Normalized Enrichment Score (NES)	-1.6624899
Nominal p-value	0.0044247787
FDR q-value	0.120829426
FWER p-Value	0.513

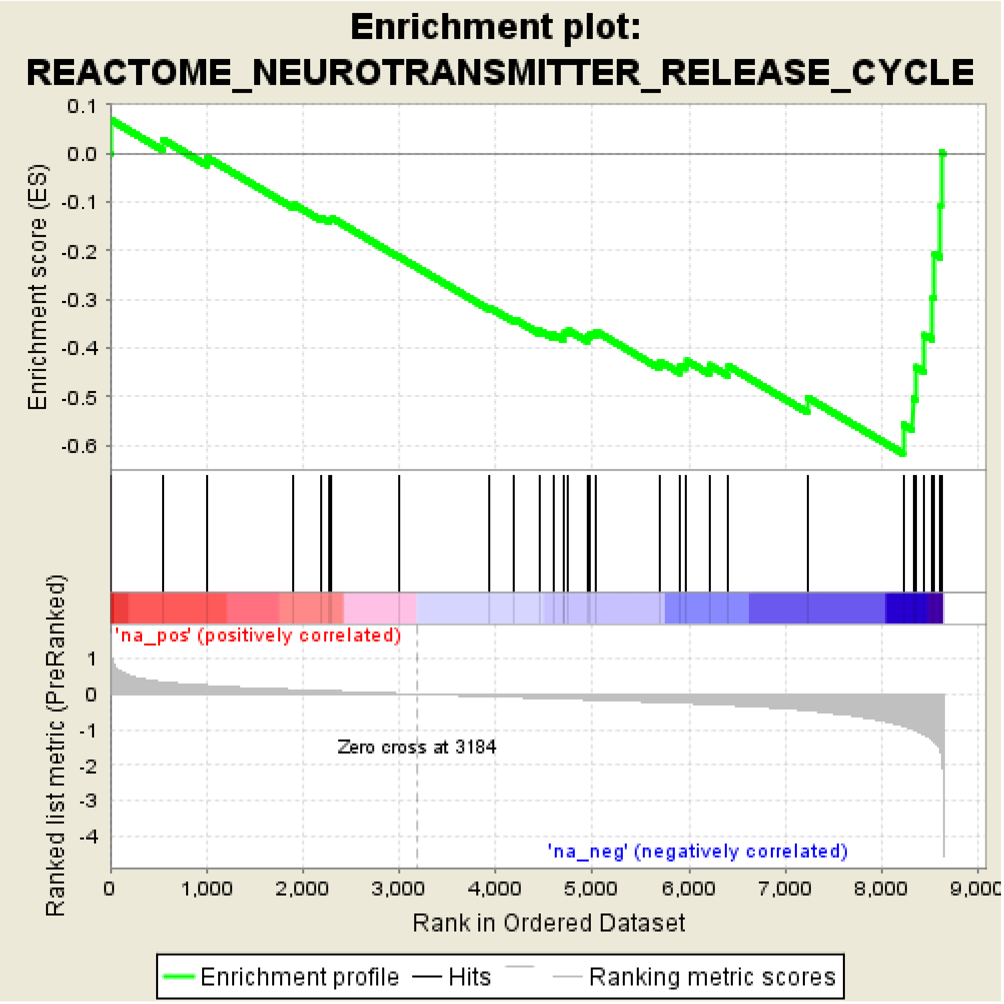

Fig 1: Enrichment plot: REACTOME_NEUROTRANSMITTER_RELEASE_CYCLE Profile of the Running ES Score & Positions of GeneSet Members on the Rank Ordered List

Table: GSEA details [plain text format]

	PROBE	GENE	GENE_TITLE	RANK	RANK	RUNNING	CORE

		SYMBOL		IN GENE LIST	METRIC SCORE	ES	ENRICHMENT
1	SLC6A1	SLC6A1 Entrez, Source	solute carrier family 6 (neurotransmitter transporter, GABA), member 1	13	1.049	0.0682	No
2	UNC13B	UNC13B Entrez, Source	unc-13 homolog B (C. elegans)	548	0.335	0.0284	No
3	CHAT	CHAT Entrez, Source	choline acetyltransferase	1005	0.242	-0.0085	No
4	HSPA8	HSPA8 Entrez, Source	heat shock 70kDa protein 8	1905	0.122	-0.1049	No
5	SLC22A2	SLC22A2 Entrez, Source	solute carrier family 22 (organic cation transporter), member 2	2195	0.094	-0.1322	No
6	GLS	GLS Entrez, Source	glutaminase	2277	0.085	-0.1360	No
7	GLS2	GLS2 Entrez, Source	glutaminase 2 (liver, mitochondrial)	2302	0.083	-0.1333	No
8	SYN3	SYN3 Entrez, Source	synapsin III	2990	0.016	-0.2120	No
9	SLC5A7	SLC5A7 Entrez, Source	solute carrier family 5 (choline transporter), member 7	3940	-0.064	-0.3180	No
10	SLC18A3	SLC18A3 Entrez, Source	solute carrier family 18 (vesicular acetylcholine), member 3	4190	-0.087	-0.3412	No
11	STX1A	STX1A Entrez, Source	syntaxin 1A (brain)	4451	-0.109	-0.3641	No
12	SYN2	SYN2 Entrez, Source	synapsin II	4593	-0.120	-0.3726	No
13	SLC38A2	SLC38A2 Entrez, Source	solute carrier family 38, member 2	4698	-0.129	-0.3760	No
14	SLC6A11	SLC6A11 Entrez, Source	solute carrier family 6 (neurotransmitter transporter, GABA), member 11	4708	-0.130	-0.3684	No
15	ALDH5A1	ALDH5A1 Entrez, Source	aldehyde dehydrogenase 5 family, member A1 (succinate-semialdehyde dehydrogenase)	4750	-0.134	-0.3643	No
16	RIMS1	RIMS1 Entrez, Source	regulating synaptic membrane exocytosis 1	4945	-0.153	-0.3766	No

17	SLC17A7	SLC17A7 Entrez, Source	solute carrier family 17 (sodium-dependent inorganic phosphate cotransporter), member 7	4973	-0.156	-0.3694	No
18	SLC32A1	SLC32A1 Entrez, Source	solute carrier family 32 (GABA vesicular transporter), member 1	5034	-0.160	-0.3658	No
19	SLC1A6	SLC1A6 Entrez, Source	solute carrier family 1 (high affinity aspartate/glutamate transporter), member 6	5697	-0.224	-0.4278	No
20	ABAT	ABAT Entrez, Source	4-aminobutyrate aminotransferase	5908	-0.247	-0.4358	No
21	SLC6A12	SLC6A12 Entrez, Source	solute carrier family 6 (neurotransmitter transporter, betaine/GABA), member 12	5976	-0.256	-0.4265	No
22	SYN1	SYN1 Entrez, Source	synapsin I	6211	-0.281	-0.4350	No
23	SNAP25	SNAP25 Entrez, Source	synaptosomal-associated protein, 25kDa	6408	-0.306	-0.4375	No
24	MAOA	MAOA Entrez, Source	monoamine oxidase A	7228	-0.458	-0.5022	No
25	GAD2	GAD2 Entrez, Source	glutamate decarboxylase 2 (pancreatic islets and brain, 65kDa)	8220	-0.898	-0.5577	Yes
26	SLC18A2	SLC18A2 Entrez, Source	solute carrier family 18 (vesicular monoamine), member 2	8322	-0.988	-0.5038	Yes
27	SLC1A1	SLC1A1 Entrez, Source	solute carrier family 1 (neuronal/epithelial high affinity glutamate transporter, system Xag), member 1	8349	-1.019	-0.4391	Yes
28	GAD1	GAD1 Entrez, Source	glutamate decarboxylase 1 (brain, 67kDa)	8441	-1.150	-0.3733	Yes
29	VAMP2	VAMP2 Entrez, Source	vesicle-associated membrane protein 2 (synaptobrevin 2)	8526	-1.314	-0.2957	Yes
30	DNAJC5	DNAJC5 Entrez, Source	DnaJ (Hsp40) homolog, subfamily C, member 5	8545	-1.363	-0.2072	Yes
31	STXBP1	STXBP1 Entrez, Source	syntaxin binding protein 1	8609	-1.599	-0.1083	Yes
32	SYT1	SYT1 Entrez, Source	synaptotagmin I	8615	-1.677	0.0026	Yes

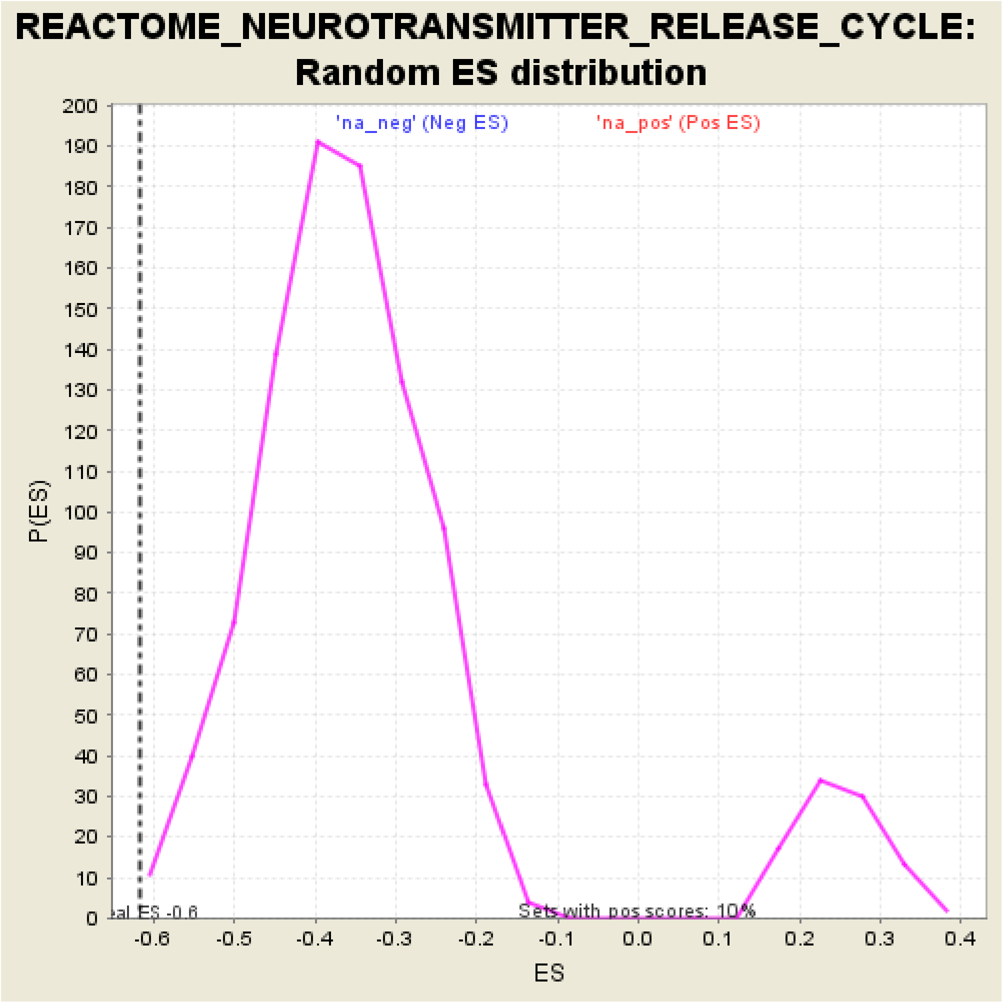

Fig 2: REACTOME_NEUROTRANSMITTER_RELEASE_CYCLE: Random ES distribution
Gene set null distribution of ES for REACTOME_NEUROTRANSMITTER_RELEASE_CYCLE

Table: GSEA Results Summary

Dataset	GSEA_18M_collapsed
Phenotype	NoPhenotypeAvailable
Upregulated in class	na_neg
GeneSet	REACTOME_LIGAND_GATED_ION_CHANNEL_TRANSPORT
Enrichment Score (ES)	-0.6861634
Normalized Enrichment Score (NES)	-1.6194682
Nominal p-value	0.008293839
FDR q-value	0.19161622
FWER p-Value	0.731

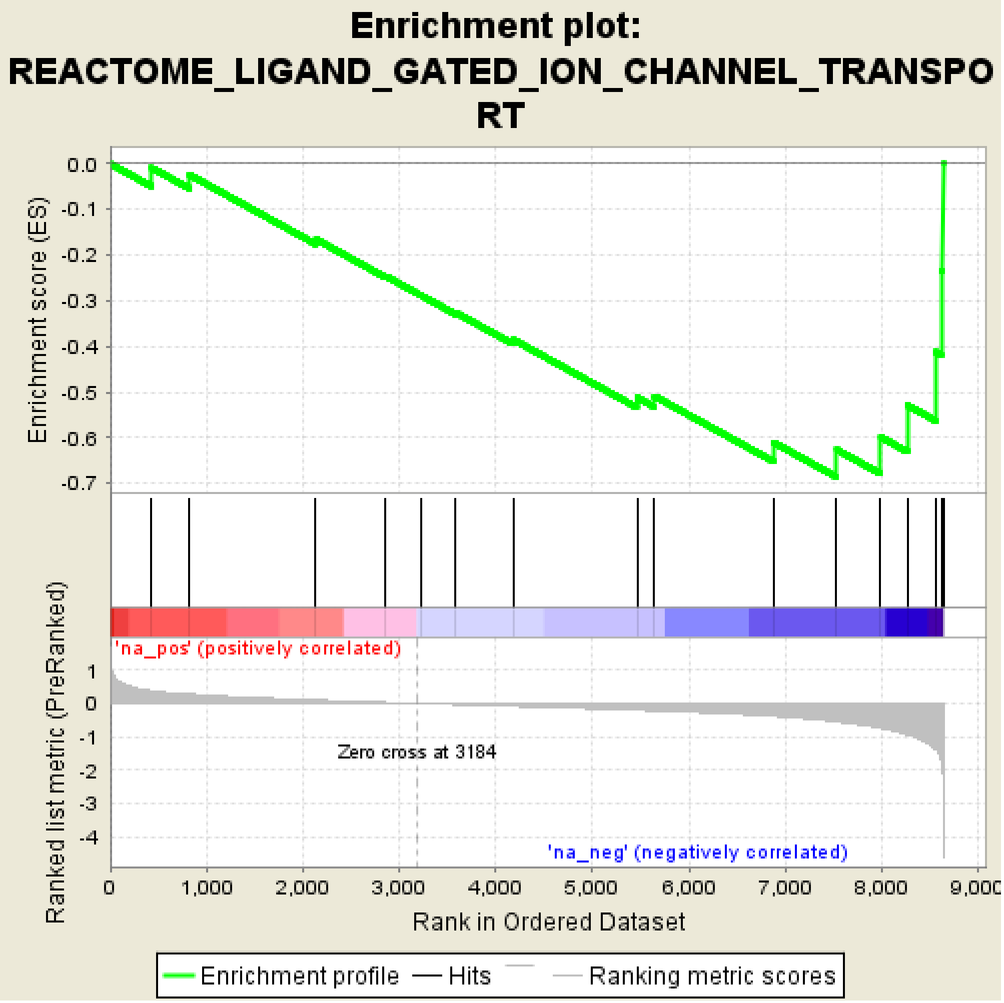

Fig 1: Enrichment plot: REACTOME_LIGAND_GATED_ION_CHANNEL_TRANSPORT Profile of the Running ES Score & Positions of GeneSet Members on the Rank Ordered List

Table: GSEA details [plain text format]

	PROBE	GENE	GENE_TITLE	RANK	RANK	RUNNING	CORE

		SYMBOL		IN GENE LIST	METRIC SCORE	ES	ENRICHMENT
1	GABRG3	GABRG3 Entrez, Source	gamma-aminobutyric acid (GABA) A receptor, gamma 3	429	0.375	-0.0089	No
2	GLRA2	GLRA2 Entrez, Source	glycine receptor, alpha 2	822	0.277	-0.0242	No
3	GABRB1	GABRB1 Entrez, Source	gamma-aminobutyric acid (GABA) A receptor, beta 1	2136	0.100	-0.1656	No
4	GLRA3	GLRA3 Entrez, Source	glycine receptor, alpha 3	2859	0.028	-0.2463	No
5	GABRA3	GABRA3 Entrez, Source	gamma-aminobutyric acid (GABA) A receptor, alpha 3	3222	-0.003	-0.2879	No
6	GLRA1	GLRA1 Entrez, Source	glycine receptor, alpha 1 (startle disease/hyperekplexia, stiff man syndrome)	3587	-0.036	-0.3263	No
7	GABRR2	GABRR2 Entrez, Source	gamma-aminobutyric acid (GABA) receptor, rho 2	4177	-0.086	-0.3852	No
8	GABRA5	GABRA5 Entrez, Source	gamma-aminobutyric acid (GABA) A receptor, alpha 5	5460	-0.201	-0.5120	No
9	GABRA4	GABRA4 Entrez, Source	gamma-aminobutyric acid (GABA) A receptor, alpha 4	5639	-0.218	-0.5088	No
10	ARHGEF9	ARHGEF9 Entrez, Source	Cdc42 guanine nucleotide exchange factor (GEF) 9	6877	-0.384	-0.6104	No
11	GABRA2	GABRA2 Entrez, Source	gamma-aminobutyric acid (GABA) A receptor, alpha 2	7531	-0.550	-0.6262	Yes
12	GABRA6	GABRA6 Entrez, Source	gamma-aminobutyric acid (GABA) A receptor, alpha 6	7988	-0.737	-0.5987	Yes
13	GABRG2	GABRG2 Entrez, Source	gamma-aminobutyric acid (GABA) A receptor, gamma 2	8267	-0.934	-0.5291	Yes
14	GABRA1	GABRA1 Entrez, Source	gamma-aminobutyric acid (GABA) A receptor, alpha 1	8559	-1.387	-0.4117	Yes
15	GABRB3	GABRB3 Entrez, Source	gamma-aminobutyric acid (GABA) A receptor, beta 3	8619	-1.698	-0.2335	Yes

16	GABRB2	GABRB2 Entrez, Source	gamma-aminobutyric acid (GABA) A receptor, beta 2	8633	-2.160	0.0005	Yes

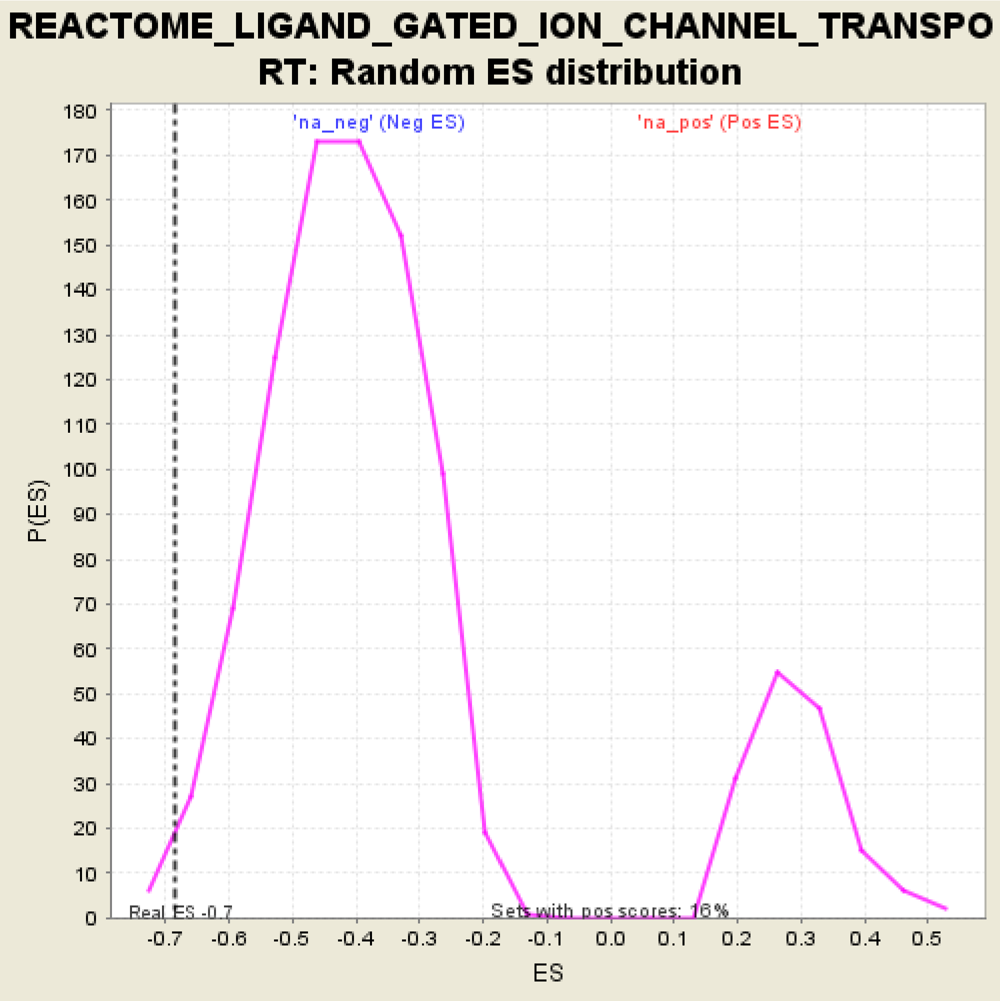

Fig 2: REACTOME_LIGAND_GATED_ION_CHANNEL_TRANSPORT: Random ES distribution
Gene set null distribution of ES for REACTOME_LIGAND_GATED_ION_CHANNEL_TRANSPORT

Table: GSEA Results Summary

Dataset	GSEA_18M_collapsed
Phenotype	NoPhenotypeAvailable
Upregulated in class	na_neg
GeneSet	REACTOME_LYSOSOME_VESICLE_BIOGENESIS
Enrichment Score (ES)	-0.6641736
Normalized Enrichment Score (NES)	-1.6016495
Nominal p-value	0.010575793
FDR q-value	0.20676766
FWER p-Value	0.802

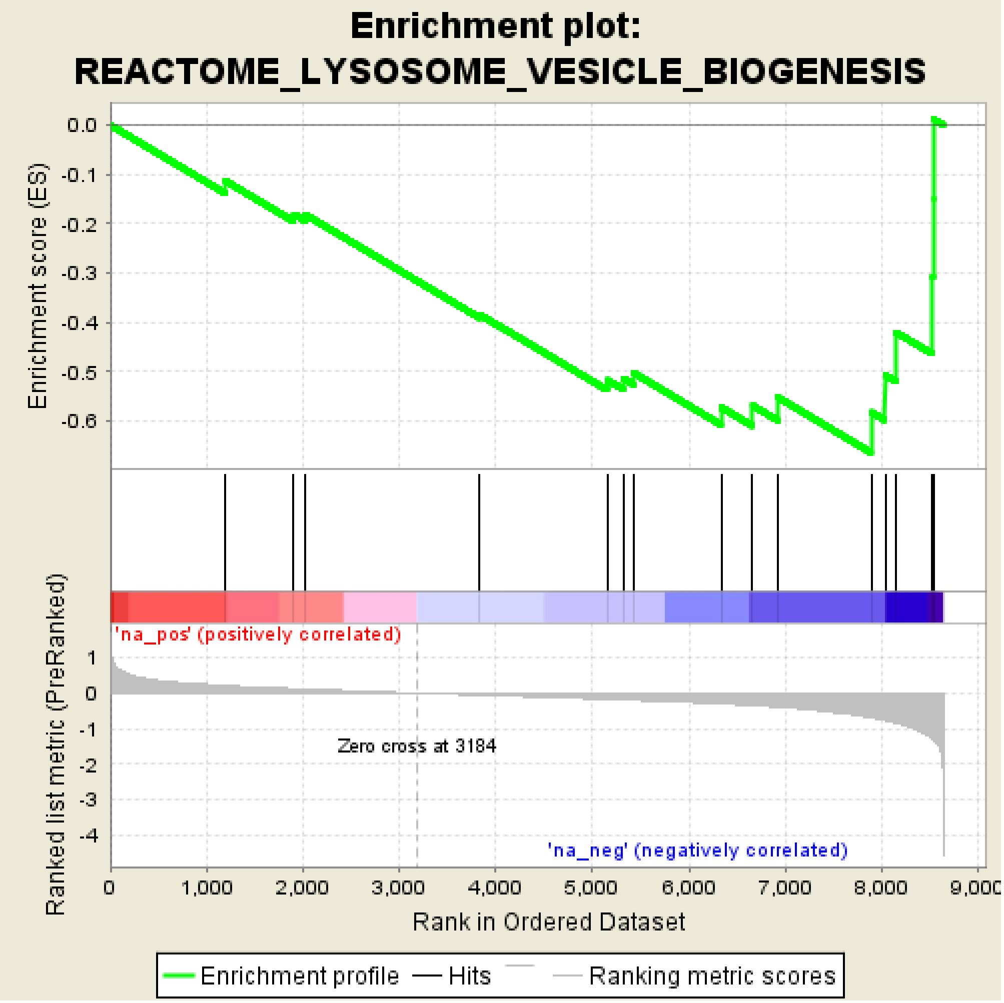

Fig 1: Enrichment plot: REACTOME_LYSOSOME_VESICLE_BIOGENESIS Profile of the Running ES Score & Positions of GeneSet Members on the Rank Ordered List

Table: GSEA details [plain text format]

	PROBE	GENE	GENE_TITLE	RANK IN	RANK	RUNNING	CORE

		SYMBOL		GENE LIST	METRIC SCORE	ES	ENRICHMENT
1	CLTC	CLTC Entrez, Source	clathrin, heavy chain (Hc)	1194	0.211	-0.1133	No
2	HSPA8	HSPA8 Entrez, Source	heat shock 70kDa protein 8	1905	0.122	-0.1810	No
3	CLTA	CLTA Entrez, Source	clathrin, light chain (Lca)	2024	0.110	-0.1816	No
4	AP1S2	AP1S2 Entrez, Source	adaptor-related protein complex 1, sigma 2 subunit	3836	-0.056	-0.3850	No
5	AP1S1	AP1S1 Entrez, Source	adaptor-related protein complex 1, sigma 1 subunit	5150	-0.171	-0.5168	No
6	BLOC1S1	BLOC1S1 Entrez, Source	biogenesis of lysosome-related organelles complex-1, subunit 1	5317	-0.186	-0.5139	No
7	CTSZ	CTSZ Entrez, Source	cathepsin Z	5423	-0.197	-0.5025	No
8	DNM2	DNM2 Entrez, Source	dynamin 2	6331	-0.296	-0.5723	No
9	VAMP8	VAMP8 Entrez, Source	vesicle-associated membrane protein 8 (endobrevin)	6661	-0.346	-0.5691	No
10	AP1G1	AP1G1 Entrez, Source	adaptor-related protein complex 1, gamma 1 subunit	6920	-0.393	-0.5521	No
11	ARF1	ARF1 Entrez, Source	ADP-ribosylation factor 1	7887	-0.689	-0.5819	Yes
12	M6PR	M6PR Entrez, Source	mannose-6-phosphate receptor (cation dependent)	8031	-0.768	-0.5067	Yes
13	TXNDC5	TXNDC5 Entrez, Source	thioredoxin domain containing 5	8147	-0.834	-0.4204	Yes
14	VAMP2	VAMP2 Entrez, Source	vesicle-associated membrane protein 2 (synaptobrevin 2)	8526	-1.314	-0.3072	Yes
15	SH3GL2	SH3GL2 Entrez, Source	SH3-domain GRB2-like 2	8533	-1.335	-0.1484	Yes
16	DNAJC6	DNAJC6 Entrez, Source	DnaJ (Hsp40) homolog, subfamily C, member 6	8536	-1.342	0.0117	Yes

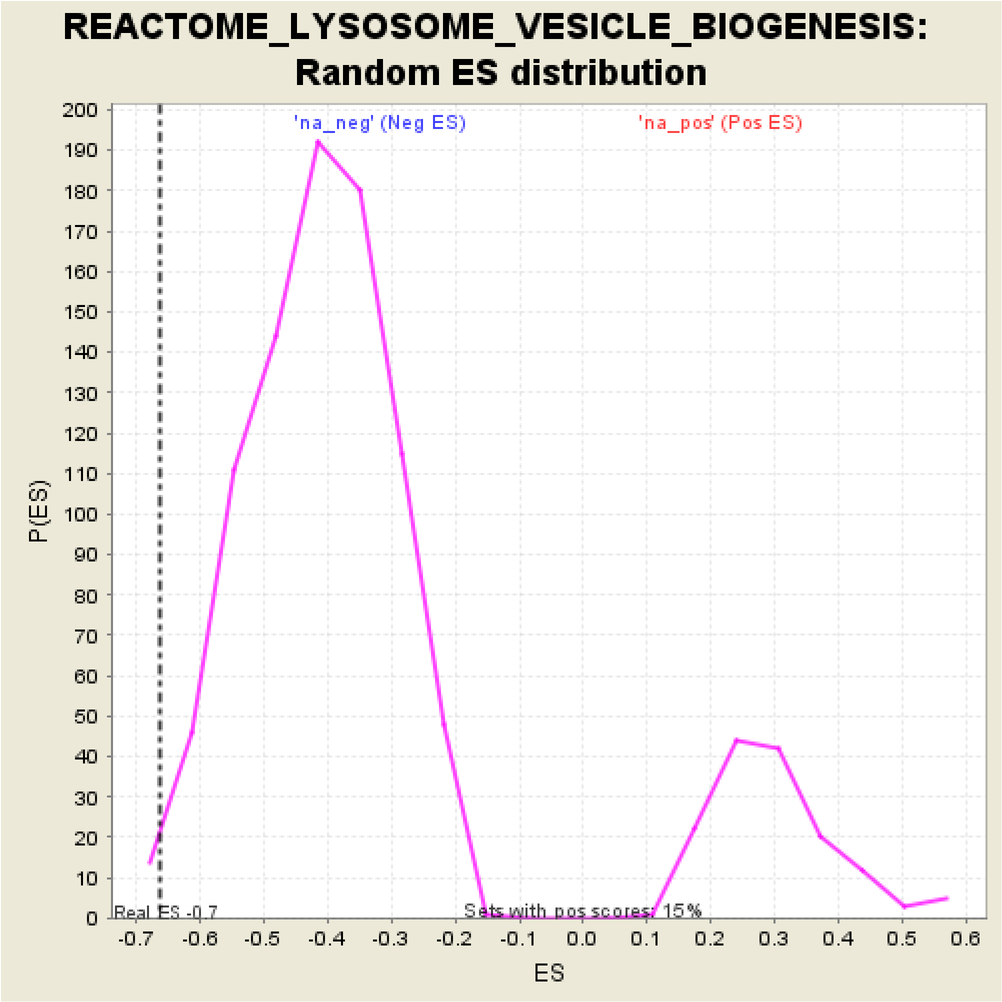

Fig 2: REACTOME_LYSOSOME_VESICLE_BIOGENESIS: Random ES distribution
Gene set null distribution of ES for REACTOME_LYSOSOME_VESICLE_BIOGENESIS

Table: GSEA Results Summary

Dataset	GSEA_18M_collapsed
Phenotype	NoPhenotypeAvailable
Upregulated in class	na_neg
GeneSet	REACTOME_SIGNALING_BY_ILS
Enrichment Score (ES)	-0.53783953
Normalized Enrichment Score (NES)	-1.583865
Nominal p-value	0.0010235414
FDR q-value	0.23137298
FWER p-Value	0.865

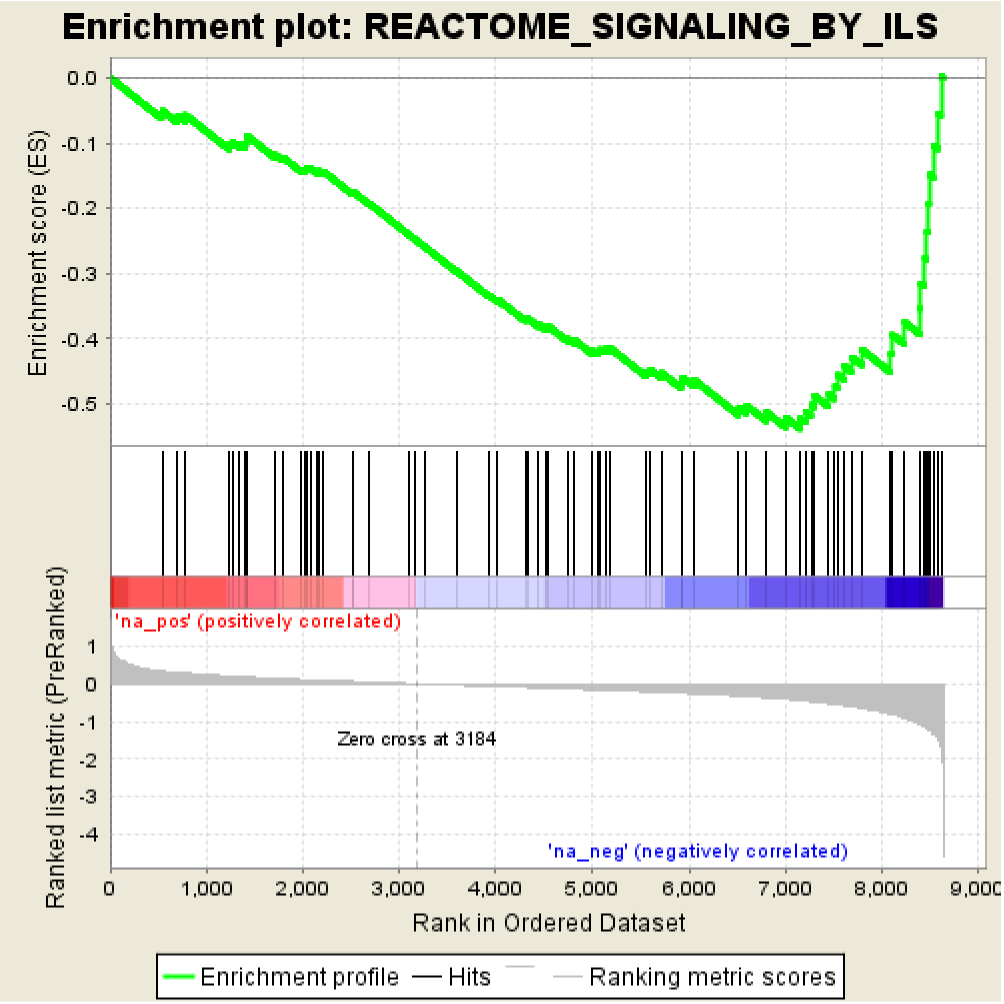

Fig 1: Enrichment plot: REACTOME_SIGNALING_BY_ILS
Profile of the Running ES Score & Positions of GeneSet Members on the Rank Ordered List

Table: GSEA details [plain text format]

	PROBE	GENE	GENE_TITLE	RANK	RANK	RUNNING	CORE

		SYMBOL		IN GENE LIST	METRIC SCORE	ES	ENRICHMENT
1	CHUK	CHUK Entrez, Source	conserved helix-loop-helix ubiquitous kinase	539	0.337	-0.0504	No
2	GAB2	GAB2 Entrez, Source	GRB2-associated binding protein 2	702	0.297	-0.0584	No
3	MAP3K8	MAP3K8 Entrez, Source	mitogen-activated protein kinase kinase kinase 8	781	0.285	-0.0570	No
4	YWHAZ	YWHAZ Entrez, Source	tyrosine 3-monooxygenase/tryptophan 5-monooxygenase activation protein, zeta polypeptide	1235	0.204	-0.1023	No
5	PIK3CD	PIK3CD Entrez, Source	phosphoinositide-3-kinase, catalytic, delta polypeptide	1265	0.200	-0.0983	No
6	IL2RB	IL2RB Entrez, Source	interleukin 2 receptor, beta	1344	0.188	-0.1005	No
7	IKBKB	IKBKB Entrez, Source	inhibitor of kappa light polypeptide gene enhancer in B-cells, kinase beta	1403	0.181	-0.1006	No
8	PTPN6	PTPN6 Entrez, Source	protein tyrosine phosphatase, non-receptor type 6	1412	0.180	-0.0948	No
9	NFKB2	NFKB2 Entrez, Source	nuclear factor of kappa light polypeptide gene enhancer in B-cells 2 (p49/p100)	1416	0.180	-0.0885	No
10	IL18	IL18 Entrez, Source	interleukin 18 (interferon-gamma-inducing factor)	1714	0.145	-0.1178	No
11	RAF1	RAF1 Entrez, Source	v-raf-1 murine leukemia viral oncogene homolog 1	1794	0.134	-0.1221	No
12	PIK3R2	PIK3R2 Entrez, Source	phosphoinositide-3-kinase, regulatory subunit 2 (p85 beta)	1989	0.114	-0.1405	No
13	IRAK3	IRAK3 Entrez, Source	interleukin-1 receptor-associated kinase 3	2027	0.109	-0.1408	No
14	RBX1	RBX1 Entrez, Source	ring-box 1	2043	0.108	-0.1386	No
15	IRAK1	IRAK1 Entrez, Source	interleukin-1 receptor-associated kinase 1	2078	0.104	-0.1387	No

16	STAT3	STAT3 Entrez, Source	signal transducer and activator of transcription 3 (acute-phase response factor)	2150	0.098	-0.1433	No
17	HGF	HGF Entrez, Source	hepatocyte growth factor (hepapoietin A; scatter factor)	2173	0.095	-0.1424	No
18	IL1R1	IL1R1 Entrez, Source	interleukin 1 receptor, type I	2214	0.092	-0.1437	No
19	CUL1	CUL1 Entrez, Source	cullin 1	2512	0.062	-0.1760	No
20	TYK2	TYK2 Entrez, Source	tyrosine kinase 2	2519	0.062	-0.1745	No
21	RELA	RELA Entrez, Source	v-rel reticuloendotheliosis viral oncogene homolog A, nuclear factor of kappa light polypeptide gene enhancer in B-cells 3, p65 (avian)	2685	0.044	-0.1921	No
22	PTK2B	PTK2B Entrez, Source	PTK2B protein tyrosine kinase 2 beta	3106	0.006	-0.2409	No
23	CSF2RA	CSF2RA Entrez, Source	colony stimulating factor 2 receptor, alpha, low-affinity (granulocyte-macrophage)	3166	0.001	-0.2477	No
24	INPPL1	INPPL1 Entrez, Source	inositol polyphosphate phosphatase-like 1	3265	-0.008	-0.2589	No
25	YES1	YES1 Entrez, Source	v-yes-1 Yamaguchi sarcoma viral oncogene homolog 1	3591	-0.036	-0.2955	No
26	MAP3K3	MAP3K3 Entrez, Source	mitogen-activated protein kinase kinase kinase 3	3936	-0.064	-0.3333	No
27	STAT5B	STAT5B Entrez, Source	signal transducer and activator of transcription 5B	4017	-0.072	-0.3400	No
28	IL7R	IL7R Entrez, Source	interleukin 7 receptor	4298	-0.096	-0.3691	No
29	FYN	FYN Entrez, Source	FYN oncogene related to SRC, FGR, YES	4317	-0.097	-0.3676	No
30	LYN	LYN Entrez, Source	v-yes-1 Yamaguchi sarcoma viral related oncogene homolog	4439	-0.108	-0.3778	No
31	MAPK3	MAPK3 Entrez, Source	mitogen-activated protein kinase 3	4509	-0.114	-0.3816	No

32	SOCS3	SOCS3 Entrez, Source	suppressor of cytokine signaling 3	4541	-0.117	-0.3809	No
33	GRB2	GRB2 Entrez, Source	growth factor receptor-bound protein 2	4744	-0.134	-0.3996	No
34	NRAS	NRAS Entrez, Source	neuroblastoma RAS viral (v-ras) oncogene homolog	4809	-0.140	-0.4019	No
35	UBE2N	UBE2N Entrez, Source	ubiquitin-conjugating enzyme E2N (UBC13 homolog, yeast)	4997	-0.157	-0.4179	No
36	BLNK	BLNK Entrez, Source	B-cell linker	5054	-0.162	-0.4184	No
37	SHC1	SHC1 Entrez, Source	SHC (Src homology 2 domain containing) transforming protein 1	5080	-0.164	-0.4153	No
38	JAK3	JAK3 Entrez, Source	Janus kinase 3 (a protein tyrosine kinase, leukocyte)	5127	-0.169	-0.4144	No
39	IL1RAP	IL1RAP Entrez, Source	interleukin 1 receptor accessory protein	5173	-0.173	-0.4133	No
40	MAP2K1	MAP2K1 Entrez, Source	mitogen-activated protein kinase kinase 1	5558	-0.211	-0.4503	No
41	MAP2K6	MAP2K6 Entrez, Source	mitogen-activated protein kinase kinase 6	5589	-0.213	-0.4459	No
42	SQSTM1	SQSTM1 Entrez, Source	sequestosome 1	5710	-0.225	-0.4516	No
43	LCK	LCK Entrez, Source	lymphocyte-specific protein tyrosine kinase	5917	-0.249	-0.4665	No
44	CASP1	CASP1 Entrez, Source	caspase 1, apoptosis-related cysteine peptidase (interleukin 1, beta, convertase)	5928	-0.250	-0.4584	No
45	TNIP2	TNIP2 Entrez, Source	TNFAIP3 interacting protein 2	6051	-0.264	-0.4629	No
46	RAPGEF1	RAPGEF1 Entrez, Source	Rap guanine nucleotide exchange factor (GEF) 1	6514	-0.322	-0.5049	No
47	PIK3CB	PIK3CB Entrez, Source	phosphoinositide-3-kinase, catalytic, beta polypeptide	6592	-0.333	-0.5016	No
48	PIK3CA	PIK3CA Entrez, Source	phosphoinositide-3-kinase, catalytic, alpha polypeptide	6801	-0.370	-0.5122	No

49	STAT1	STAT1 Entrez, Source	signal transducer and activator of transcription 1, 91kDa	6998	-0.406	-0.5201	No
50	HCK	HCK Entrez, Source	hemopoietic cell kinase	7151	-0.437	-0.5217	Yes
51	PIK3R3	PIK3R3 Entrez, Source	phosphoinositide-3-kinase, regulatory subunit 3 (p55, gamma)	7209	-0.454	-0.5116	Yes
52	PELI2	PELI2 Entrez, Source	pellino homolog 2 (Drosophila)	7265	-0.469	-0.5007	Yes
53	IKBKG	IKBKG Entrez, Source	inhibitor of kappa light polypeptide gene enhancer in B-cells, kinase gamma	7299	-0.479	-0.4868	Yes
54	MAPK1	MAPK1 Entrez, Source	mitogen-activated protein kinase 1	7446	-0.522	-0.4846	Yes
55	TRAF6	TRAF6 Entrez, Source	TNF receptor-associated factor 6	7512	-0.544	-0.4721	Yes
56	JAK2	JAK2 Entrez, Source	Janus kinase 2 (a protein tyrosine kinase)	7541	-0.554	-0.4548	Yes
57	SOS1	SOS1 Entrez, Source	son of sevenless homolog 1 (Drosophila)	7606	-0.574	-0.4411	Yes
58	MAP2K4	MAP2K4 Entrez, Source	mitogen-activated protein kinase kinase 4	7683	-0.601	-0.4278	Yes
59	BTRC	BTRC Entrez, Source	beta-transducin repeat containing	7791	-0.647	-0.4163	Yes
60	MAP3K7	MAP3K7 Entrez, Source	mitogen-activated protein kinase kinase kinase 7	8090	-0.803	-0.4215	Yes
61	IL6ST	IL6ST Entrez, Source	interleukin 6 signal transducer (gp130, oncostatin M receptor)	8110	-0.814	-0.3936	Yes
62	JAK1	JAK1 Entrez, Source	Janus kinase 1 (a protein tyrosine kinase)	8234	-0.908	-0.3744	Yes
63	CBL	CBL Entrez, Source	Cas-Br-M (murine) ecotropic retroviral transforming sequence	8393	-1.070	-0.3533	Yes
64	YWHAB	YWHAB Entrez, Source	tyrosine 3-monooxygenase/tryptophan 5-monooxygenase activation protein, beta polypeptide	8401	-1.077	-0.3143	Yes

65	KRAS	KRAS Entrez, Source	v-Ki-ras2 Kirsten rat sarcoma viral oncogene homolog	8444	-1.153	-0.2766	Yes
66	PIK3R1	PIK3R1 Entrez, Source	phosphoinositide-3-kinase, regulatory subunit 1 (p85 alpha)	8463	-1.179	-0.2351	Yes
67	PELI1	PELI1 Entrez, Source	pellino homolog 1 (Drosophila)	8482	-1.209	-0.1925	Yes
68	CRK	CRK Entrez, Source	v-crk sarcoma virus CT10 oncogene homolog (avian)	8490	-1.223	-0.1482	Yes
69	CRKL	CRKL Entrez, Source	v-crk sarcoma virus CT10 oncogene homolog (avian)-like	8535	-1.339	-0.1038	Yes
70	TOLLIP	TOLLIP Entrez, Source	toll interacting protein	8584	-1.449	-0.0559	Yes
71	PRKACB	PRKACB Entrez, Source	protein kinase, cAMP-dependent, catalytic, beta	8614	-1.677	0.0027	Yes

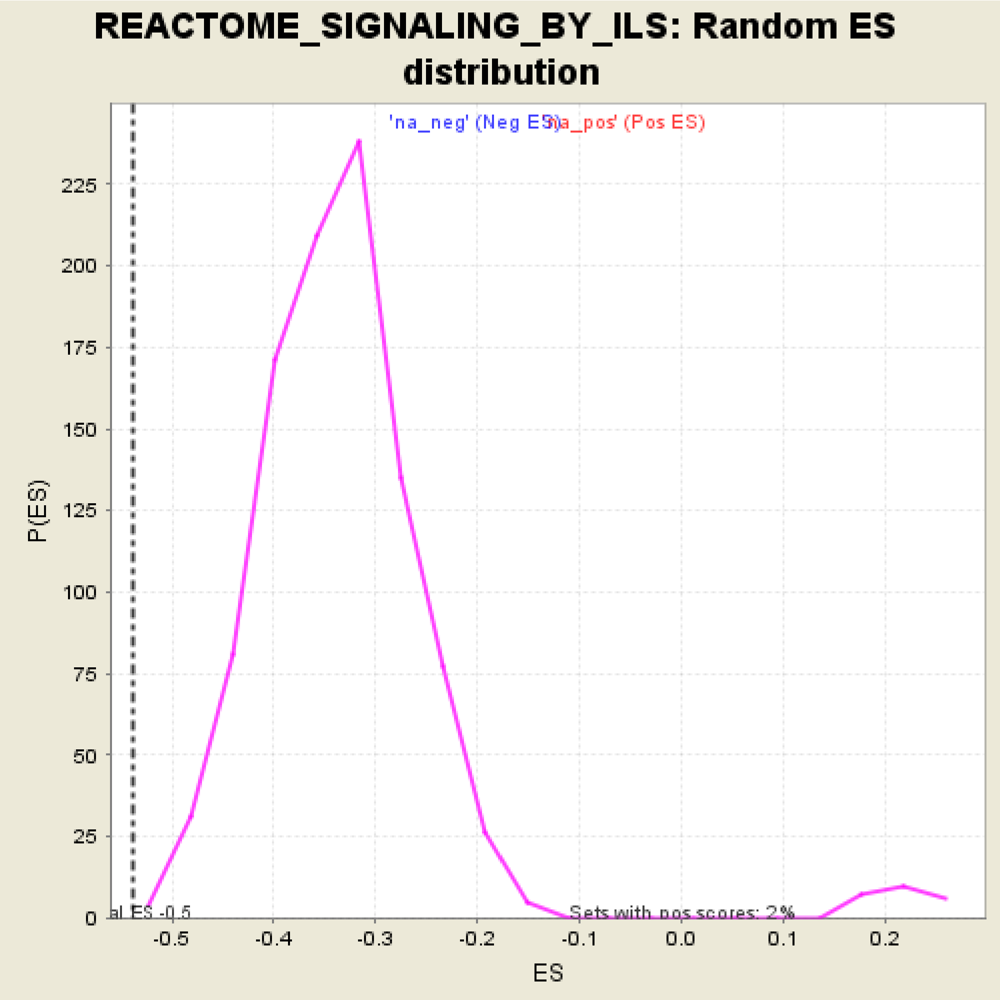

Fig 2: REACTOME_SIGNALING_BY_ILS: Random ES distribution
Gene set null distribution of ES for REACTOME_SIGNALING_BY_ILS

Table: GSEA Results Summary

Dataset	GSEA_18M_collapsed
Phenotype	NoPhenotypeAvailable
Upregulated in class	na_neg
GeneSet	REACTOME_MAP_KINASE_ACTIVATION_IN_TLR_CASCADE
Enrichment Score (ES)	-0.57339615
Normalized Enrichment Score (NES)	-1.5781901
Nominal p-value	0.005376344
FDR q-value	0.22318275
FWER p-Value	0.878

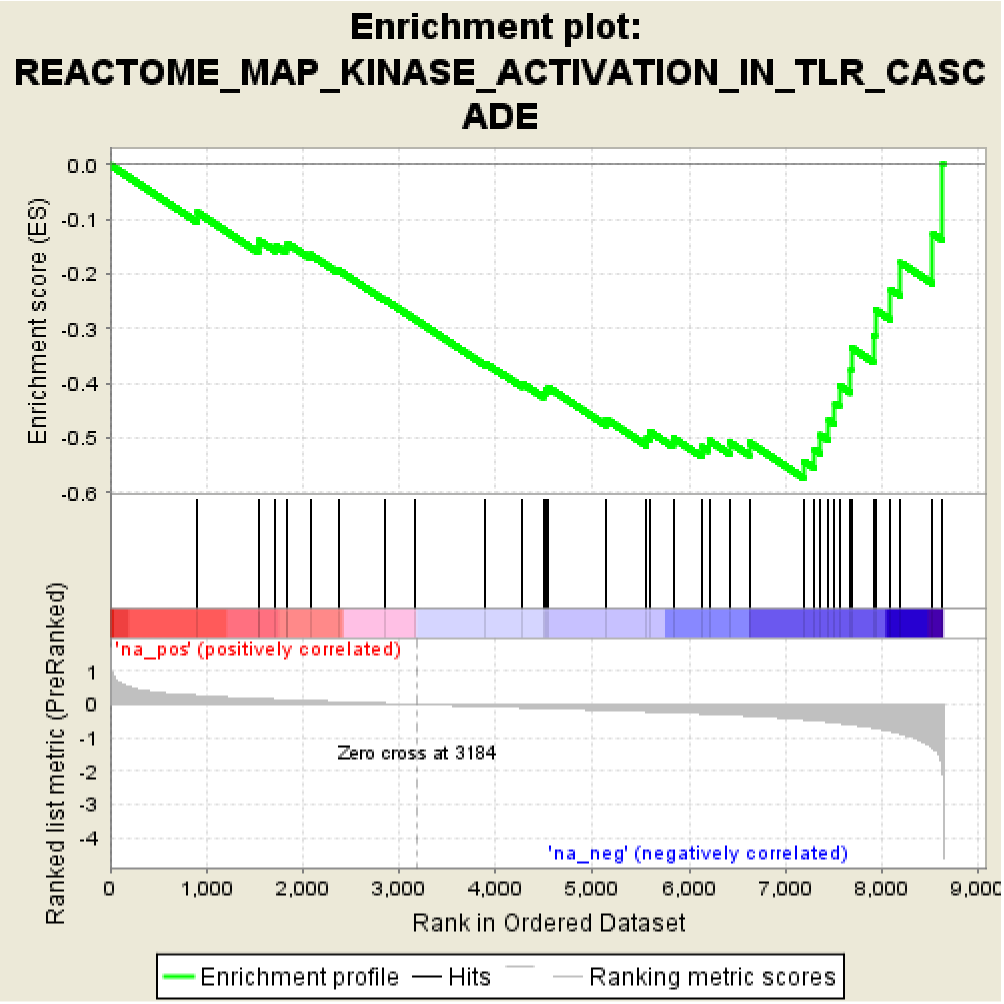

Fig 1: Enrichment plot: REACTOME_MAP_KINASE_ACTIVATION_IN_TLR_CASCADE Profile of the Running ES Score & Positions of GeneSet Members on the Rank Ordered List

Table: GSEA details [plain text format]

	PROBE	GENE	GENE_TITLE	RANK	RANK	RUNNING	CORE

		SYMBOL		IN GENE LIST	METRIC SCORE	ES	ENRICHMENT
1	MAPK8	MAPK8 Entrez, Source	mitogen-activated protein kinase 8	906	0.262	-0.0871	No
2	RPS6KA1	RPS6KA1 Entrez, Source	ribosomal protein S6 kinase, 90kDa, polypeptide 1	1540	0.165	-0.1493	No
3	FOS	FOS Entrez, Source	v-fos FBJ murine osteosarcoma viral oncogene homolog	1548	0.164	-0.1386	No
4	MEF2A	MEF2A Entrez, Source	MADS box transcription enhancer factor 2, polypeptide A (myocyte enhancer factor 2A)	1719	0.145	-0.1483	No
5	MAPK11	MAPK11 Entrez, Source	mitogen-activated protein kinase 11	1826	0.131	-0.1515	No
6	PPP2R1B	PPP2R1B Entrez, Source	protein phosphatase 2 (formerly 2A), regulatory subunit A (PR 65), beta isoform	1840	0.130	-0.1440	No
7	IRAK1	IRAK1 Entrez, Source	interleukin-1 receptor-associated kinase 1	2078	0.104	-0.1643	No
8	MAP2K3	MAP2K3 Entrez, Source	mitogen-activated protein kinase kinase 3	2365	0.077	-0.1922	No
9	MAPK7	MAPK7 Entrez, Source	mitogen-activated protein kinase 7	2852	0.029	-0.2467	No
10	RPS6KA2	RPS6KA2 Entrez, Source	ribosomal protein S6 kinase, 90kDa, polypeptide 2	3164	0.002	-0.2828	No
11	MAPKAPK3	MAPKAPK3 Entrez, Source	mitogen-activated protein kinase-activated protein kinase 3	3892	-0.060	-0.3631	No
12	DUSP3	DUSP3 Entrez, Source	dual specificity phosphatase 3 (vaccinia virus phosphatase VH1-related)	4269	-0.093	-0.4004	No
13	MAPKAPK2	MAPKAPK2 Entrez, Source	mitogen-activated protein kinase-activated protein kinase 2	4486	-0.112	-0.4177	No
14	MAPK3	MAPK3 Entrez, Source	mitogen-activated protein kinase 3	4509	-0.114	-0.4124	No
15	ATF1	ATF1 Entrez, Source	activating transcription factor 1	4537	-0.116	-0.4074	No

16	PPP2CB	PPP2CB Entrez, Source	protein phosphatase 2 (formerly 2A), catalytic subunit, beta isoform	5137	-0.170	-0.4653	No
17	MAP2K1	MAP2K1 Entrez, Source	mitogen-activated protein kinase kinase 1	5558	-0.211	-0.4995	No
18	MAP2K6	MAP2K6 Entrez, Source	mitogen-activated protein kinase kinase 6	5589	-0.213	-0.4882	No
19	CREB1	CREB1 Entrez, Source	cAMP responsive element binding protein 1	5833	-0.238	-0.4999	No
20	JUN	JUN Entrez, Source	jun oncogene	6134	-0.272	-0.5159	No
21	DUSP7	DUSP7 Entrez, Source	dual specificity phosphatase 7	6205	-0.281	-0.5045	No
22	MAPK10	MAPK10 Entrez, Source	mitogen-activated protein kinase 10	6420	-0.307	-0.5080	No
23	MEF2C	MEF2C Entrez, Source	MADS box transcription enhancer factor 2, polypeptide C (myocyte enhancer factor 2C)	6625	-0.339	-0.5082	No
24	ELK1	ELK1 Entrez, Source	ELK1, member of ETS oncogene family	7187	-0.449	-0.5422	Yes
25	IKBKG	IKBKG Entrez, Source	inhibitor of kappa light polypeptide gene enhancer in B-cells, kinase gamma	7299	-0.479	-0.5218	Yes
26	DUSP6	DUSP6 Entrez, Source	dual specificity phosphatase 6	7361	-0.498	-0.4943	Yes
27	MAPK1	MAPK1 Entrez, Source	mitogen-activated protein kinase 1	7446	-0.522	-0.4678	Yes
28	TRAF6	TRAF6 Entrez, Source	TNF receptor-associated factor 6	7512	-0.544	-0.4375	Yes
29	RPS6KA3	RPS6KA3 Entrez, Source	ribosomal protein S6 kinase, 90kDa, polypeptide 3	7559	-0.558	-0.4041	Yes
30	MAP2K7	MAP2K7 Entrez, Source	mitogen-activated protein kinase kinase 7	7677	-0.600	-0.3760	Yes
31	MAP2K4	MAP2K4 Entrez, Source	mitogen-activated protein kinase kinase 4	7683	-0.601	-0.3348	Yes

32	DUSP4	DUSP4 Entrez, Source	dual specificity phosphatase 4	7913	-0.701	-0.3127	Yes
33	ATF2	ATF2 Entrez, Source	activating transcription factor 2	7931	-0.710	-0.2654	Yes
34	MAP3K7	MAP3K7 Entrez, Source	mitogen-activated protein kinase kinase kinase 7	8090	-0.803	-0.2280	Yes
35	MAPK14	MAPK14 Entrez, Source	mitogen-activated protein kinase 14	8194	-0.875	-0.1791	Yes
36	MAPK9	MAPK9 Entrez, Source	mitogen-activated protein kinase 9	8528	-1.320	-0.1261	Yes
37	RPS6KA5	RPS6KA5 Entrez, Source	ribosomal protein S6 kinase, 90kDa, polypeptide 5	8631	-1.996	0.0007	Yes

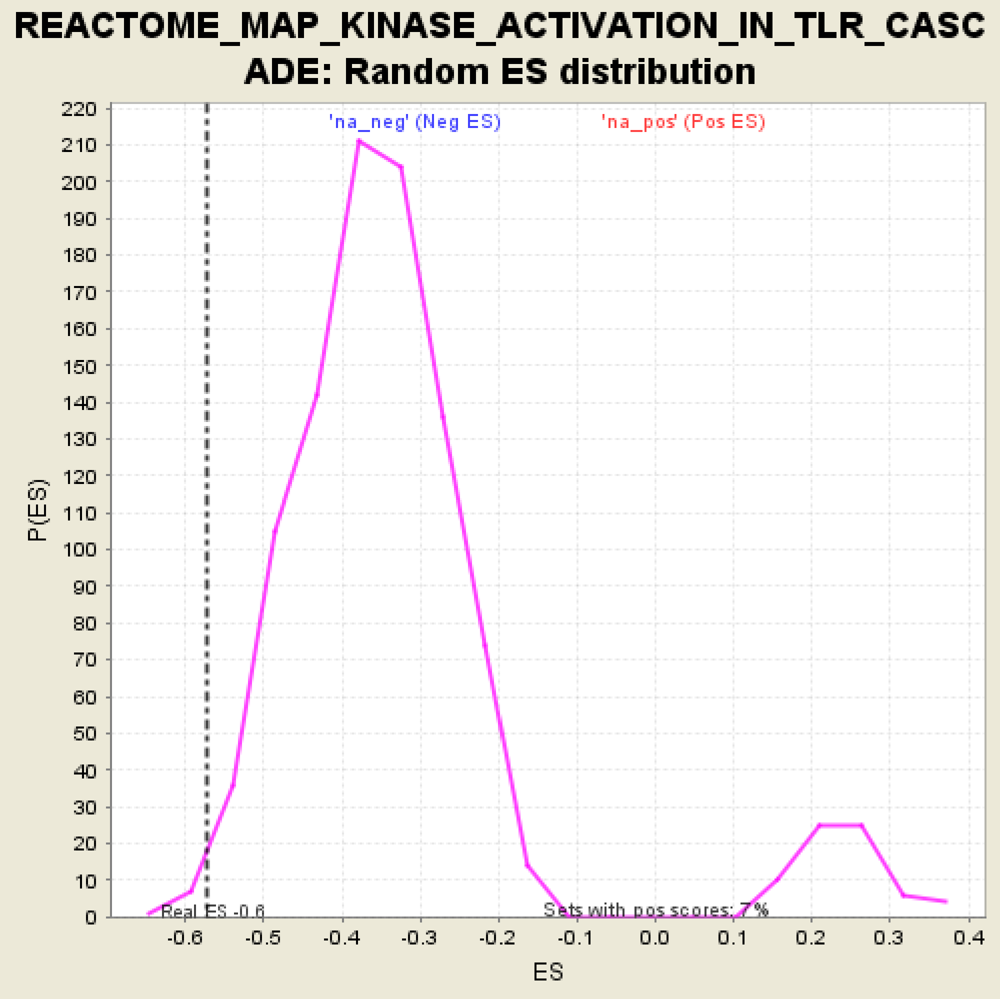

Fig 2: REACTOME_MAP_KINASE_ACTIVATION_IN_TLR_CASCADE: Random ES distribution
Gene set null distribution of ES for REACTOME_MAP_KINASE_ACTIVATION_IN_TLR_CASCADE

Table: GSEA Results Summary

Dataset	GSEA_18M_collapsed
Phenotype	NoPhenotypeAvailable
Upregulated in class	na_neg
GeneSet	REACTOME_ION_CHANNEL_TRANSPORT
Enrichment Score (ES)	-0.57235
Normalized Enrichment Score (NES)	-1.5682837
Nominal p-value	0.0075593954
FDR q-value	0.23073831
FWER p-Value	0.907

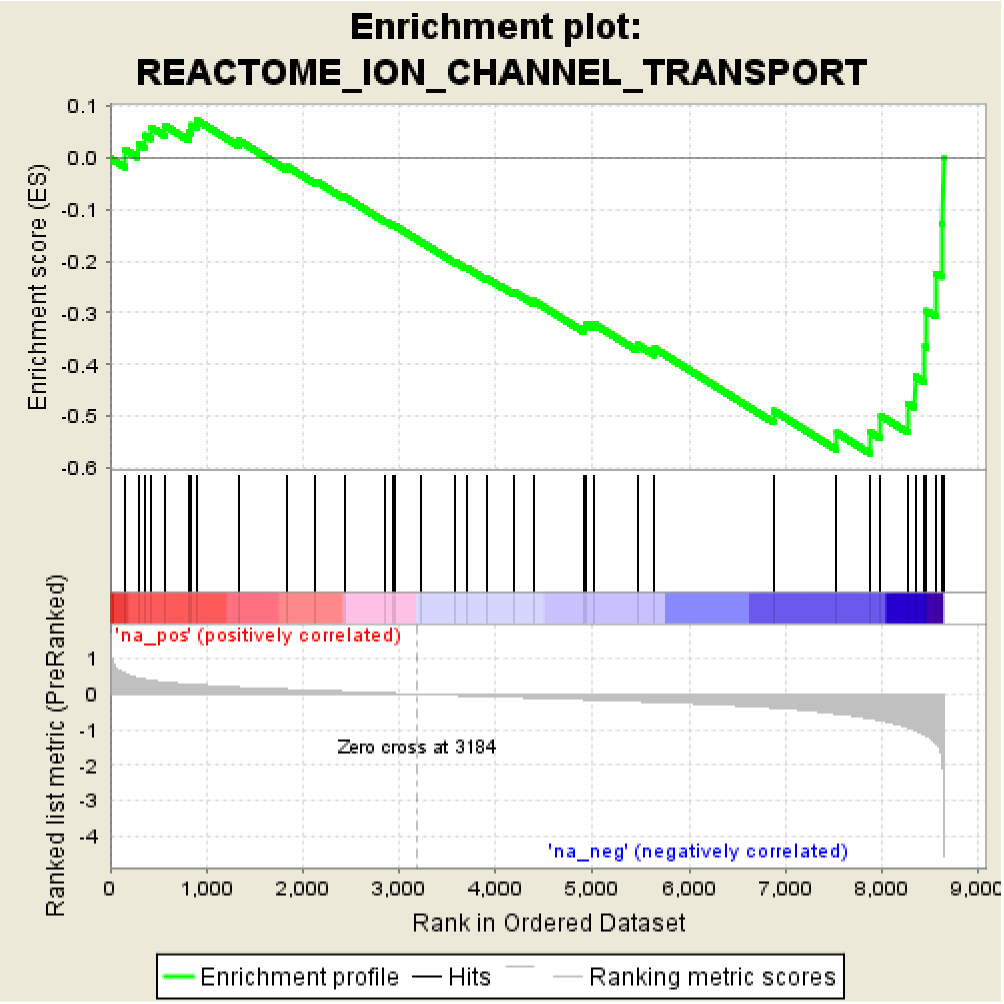

Fig 1: Enrichment plot: REACTOME_ION_CHANNEL_TRANSPORT
Profile of the Running ES Score & Positions of GeneSet Members on the Rank Ordered List

Table: GSEA details [plain text format]

	PROBE	GENE	GENE_TITLE	RANK	RANK	RUNNING	CORE

		SYMBOL		IN GENE LIST	METRIC SCORE	ES	ENRICHMENT
1	ATP1A1	ATP1A1 Entrez, Source	ATPase, Na+/K+ transporting, alpha 1 polypeptide	157	0.571	0.0162	No
2	ATP2A1	ATP2A1 Entrez, Source	ATPase, Ca++ transporting, cardiac muscle, fast twitch 1	289	0.446	0.0278	No
3	FXYD2	FXYD2 Entrez, Source	FXYD domain containing ion transport regulator 2	358	0.410	0.0446	No
4	GABRG3	GABRG3 Entrez, Source	gamma-aminobutyric acid (GABA) A receptor, gamma 3	429	0.375	0.0591	No
5	ATP7B	ATP7B Entrez, Source	ATPase, Cu++ transporting, beta polypeptide	570	0.328	0.0626	No
6	GLRA2	GLRA2 Entrez, Source	glycine receptor, alpha 2	822	0.277	0.0501	No
7	ATP10A	ATP10A Entrez, Source	ATPase, Class V, type 10A	845	0.273	0.0640	No
8	ATP8A2	ATP8A2 Entrez, Source	ATPase, aminophospholipid transporter-like, Class I, type 8A, member 2	903	0.262	0.0731	No
9	ATP8B1	ATP8B1 Entrez, Source	ATPase, Class I, type 8B, member 1	1339	0.189	0.0339	No
10	ATP11B	ATP11B Entrez, Source	ATPase, Class VI, type 11B	1843	0.130	-0.0168	No
11	GABRB1	GABRB1 Entrez, Source	gamma-aminobutyric acid (GABA) A receptor, beta 1	2136	0.100	-0.0447	No
12	ATP7A	ATP7A Entrez, Source	ATPase, Cu++ transporting, alpha polypeptide (Menkes syndrome)	2432	0.070	-0.0748	No
13	GLRA3	GLRA3 Entrez, Source	glycine receptor, alpha 3	2859	0.028	-0.1226	No
14	ATP9B	ATP9B Entrez, Source	ATPase, Class II, type 9B	2927	0.022	-0.1291	No
15	ATP1A2	ATP1A2 Entrez, Source	ATPase, Na+/K+ transporting, alpha 2 (+) polypeptide	2948	0.020	-0.1302	No
16	GABRA3	GABRA3 Entrez, Source	gamma-aminobutyric acid (GABA) A receptor, alpha 3	3222	-0.003	-0.1617	No

17	GLRA1	GLRA1 Entrez, Source	glycine receptor, alpha 1 (startle disease/hyperekplexia, stiff man syndrome)	3587	-0.036	-0.2019	No
18	ATP2A2	ATP2A2 Entrez, Source	ATPase, Ca++ transporting, cardiac muscle, slow twitch 2	3695	-0.045	-0.2116	No
19	ATP2B3	ATP2B3 Entrez, Source	ATPase, Ca++ transporting, plasma membrane 3	3908	-0.061	-0.2326	No
20	GABRR2	GABRR2 Entrez, Source	gamma-aminobutyric acid (GABA) receptor, rho 2	4177	-0.086	-0.2586	No
21	ATP2B2	ATP2B2 Entrez, Source	ATPase, Ca++ transporting, plasma membrane 2	4383	-0.103	-0.2762	No
22	ATP10B	ATP10B Entrez, Source	ATPase, Class V, type 10B	4905	-0.150	-0.3278	No
23	ATP1B3	ATP1B3 Entrez, Source	ATPase, Na+/K+ transporting, beta 3 polypeptide	4925	-0.151	-0.3209	No
24	ATP8A1	ATP8A1 Entrez, Source	ATPase, aminophospholipid transporter (APLT), Class I, type 8A, member 1	5014	-0.159	-0.3215	No
25	GABRA5	GABRA5 Entrez, Source	gamma-aminobutyric acid (GABA) A receptor, alpha 5	5460	-0.201	-0.3612	No
26	GABRA4	GABRA4 Entrez, Source	gamma-aminobutyric acid (GABA) A receptor, alpha 4	5639	-0.218	-0.3687	No
27	ARHGEF9	ARHGEF9 Entrez, Source	Cdc42 guanine nucleotide exchange factor (GEF) 9	6877	-0.384	-0.4894	No
28	GABRA2	GABRA2 Entrez, Source	gamma-aminobutyric acid (GABA) A receptor, alpha 2	7531	-0.550	-0.5321	Yes
29	ATP2A3	ATP2A3 Entrez, Source	ATPase, Ca++ transporting, ubiquitous	7878	-0.687	-0.5310	Yes
30	GABRA6	GABRA6 Entrez, Source	gamma-aminobutyric acid (GABA) A receptor, alpha 6	7988	-0.737	-0.4992	Yes
31	GABRG2	GABRG2 Entrez, Source	gamma-aminobutyric acid (GABA) A receptor, gamma 2	8267	-0.934	-0.4753	Yes
32	ATP11C	ATP11C Entrez, Source	ATPase, Class VI, type 11C	8343	-1.013	-0.4230	Yes

33	ATP2C1	ATP2C1 Entrez, Source	ATPase, Ca++ transporting, type 2C, member 1	8445	-1.154	-0.3652	Yes
34	ATP1B2	ATP1B2 Entrez, Source	ATPase, Na+/K+ transporting, beta 2 polypeptide	8462	-1.179	-0.2960	Yes
35	GABRA1	GABRA1 Entrez, Source	gamma-aminobutyric acid (GABA) A receptor, alpha 1	8559	-1.387	-0.2236	Yes
36	GABRB3	GABRB3 Entrez, Source	gamma-aminobutyric acid (GABA) A receptor, beta 3	8619	-1.698	-0.1282	Yes
37	GABRB2	GABRB2 Entrez, Source	gamma-aminobutyric acid (GABA) A receptor, beta 2	8633	-2.160	0.0005	Yes

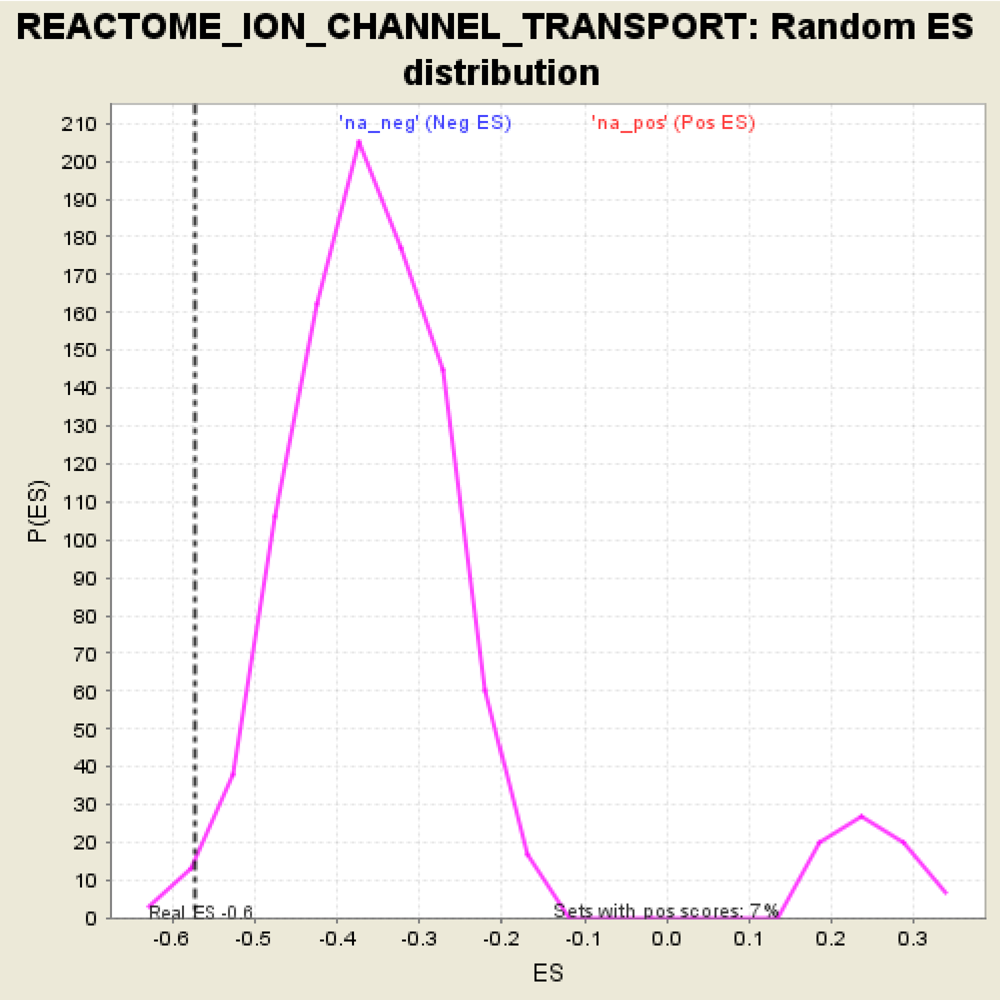

Fig 2: REACTOME_ION_CHANNEL_TRANSPORT: Random ES distribution
Gene set null distribution of ES for REACTOME_ION_CHANNEL_TRANSPORT

Table: GSEA Results Summary

Dataset	GSEA_18M_collapsed
Phenotype	NoPhenotypeAvailable
Upregulated in class	na_neg
GeneSet	REACTOME_SIGNALING_BY_EGFR_IN_CANCER
Enrichment Score (ES)	-0.5272721
Normalized Enrichment Score (NES)	-1.5673778
Nominal p-value	0.0
FDR q-value	0.21364374
FWER p-Value	0.91

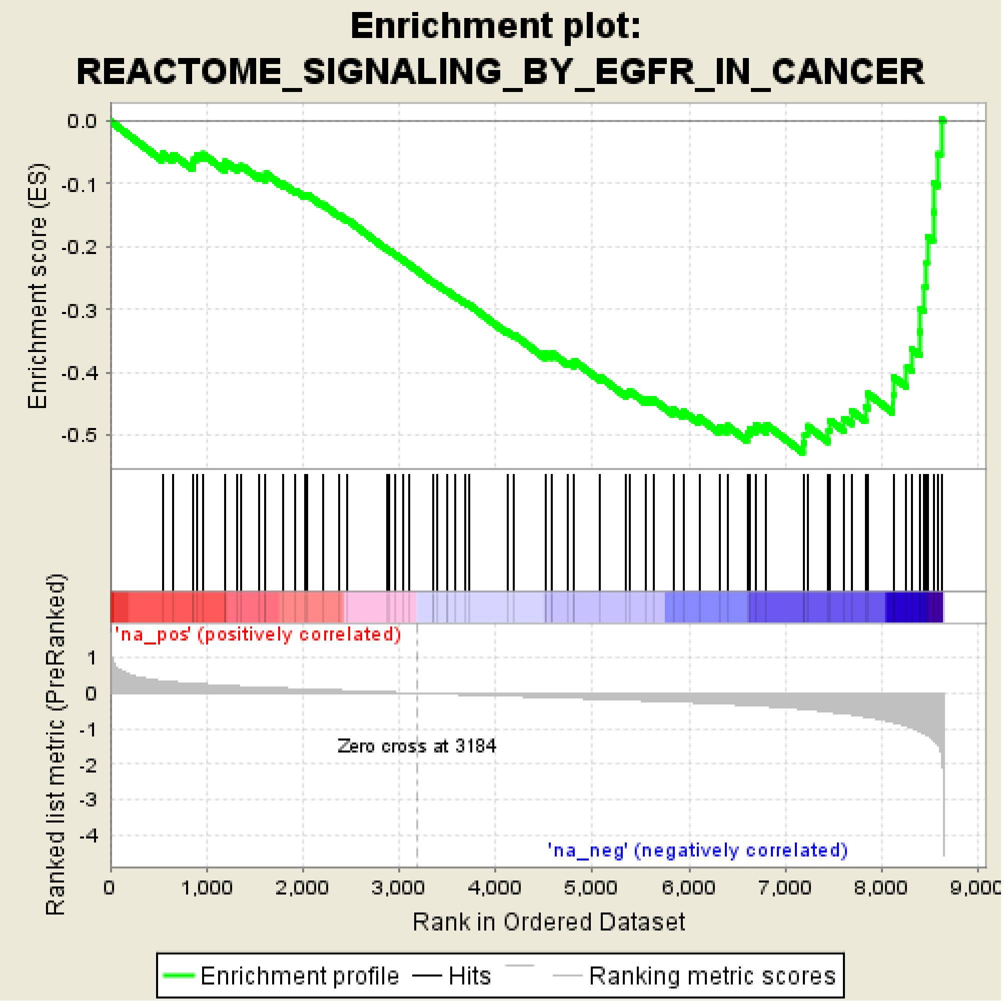

Fig 1: Enrichment plot: REACTOME_SIGNALING_BY_EGFR_IN_CANCER Profile of the Running ES Score & Positions of GeneSet Members on the Rank Ordered List

Table: GSEA details [plain text format]

	PROBE	GENE	GENE_TITLE	RANK	RANK	RUNNING	CORE

		SYMBOL		IN GENE LIST	METRIC SCORE	ES	ENRICHMENT
1	CHUK	CHUK Entrez, Source	conserved helix-loop-helix ubiquitous kinase	539	0.337	-0.0512	No
2	NR4A1	NR4A1 Entrez, Source	nuclear receptor subfamily 4, group A, member 1	647	0.310	-0.0529	No
3	PTEN	PTEN Entrez, Source	phosphatase and tensin homolog (mutated in multiple advanced cancers 1)	855	0.271	-0.0677	No
4	ADCY1	ADCY1 Entrez, Source	adenylate cyclase 1 (brain)	868	0.269	-0.0597	No
5	PRKACA	PRKACA Entrez, Source	protein kinase, cAMP-dependent, catalytic, alpha	893	0.264	-0.0533	No
6	EPS15L1	EPS15L1 Entrez, Source	epidermal growth factor receptor pathway substrate 15-like 1	960	0.250	-0.0524	No
7	ADCY5	ADCY5 Entrez, Source	adenylate cyclase 5	1188	0.212	-0.0715	No
8	CLTC	CLTC Entrez, Source	clathrin, heavy chain (Hc)	1194	0.211	-0.0648	No
9	ADAM12	ADAM12 Entrez, Source	ADAM metallopeptidase domain 12 (meltrin alpha)	1315	0.192	-0.0721	No
10	TRIB3	TRIB3 Entrez, Source	tribbles homolog 3 (Drosophila)	1365	0.185	-0.0713	No
11	RPS6KB2	RPS6KB2 Entrez, Source	ribosomal protein S6 kinase, 70kDa, polypeptide 2	1550	0.164	-0.0871	No
12	ITPR2	ITPR2 Entrez, Source	inositol 1,4,5-triphosphate receptor, type 2	1615	0.156	-0.0892	No
13	ADAM10	ADAM10 Entrez, Source	ADAM metallopeptidase domain 10	1617	0.156	-0.0839	No
14	RAF1	RAF1 Entrez, Source	v-raf-1 murine leukemia viral oncogene homolog 1	1794	0.134	-0.0998	No
15	AP2A1	AP2A1 Entrez, Source	adaptor-related protein complex 2, alpha 1 subunit	1919	0.121	-0.1100	No
16	CLTA	CLTA Entrez, Source	clathrin, light chain (Lca)	2024	0.110	-0.1184	No

17	ADCY6	ADCY6 Entrez, Source	adenylate cyclase 6	2045	0.108	-0.1170	No
18	PRKCD	PRKCD Entrez, Source	protein kinase C, delta	2203	0.093	-0.1321	No
19	ADCY7	ADCY7 Entrez, Source	adenylate cyclase 7	2380	0.075	-0.1500	No
20	PDE1B	PDE1B Entrez, Source	phosphodiesterase 1B, calmodulin-dependent	2464	0.067	-0.1574	No
21	GSK3A	GSK3A Entrez, Source	glycogen synthase kinase 3 alpha	2867	0.027	-0.2034	No
22	CSK	CSK Entrez, Source	c-src tyrosine kinase	2899	0.025	-0.2061	No
23	CDKN1A	CDKN1A Entrez, Source	cyclin-dependent kinase inhibitor 1A (p21, Cip1)	2964	0.018	-0.2130	No
24	PAG1	PAG1 Entrez, Source	phosphoprotein associated with glycosphingolipid microdomains 1	3035	0.012	-0.2207	No
25	PDE1A	PDE1A Entrez, Source	phosphodiesterase 1A, calmodulin-dependent	3101	0.006	-0.2281	No
26	PDPK1	PDPK1 Entrez, Source	3-phosphoinositide dependent protein kinase-1	3347	-0.015	-0.2562	No
27	EGFR	EGFR Entrez, Source	epidermal growth factor receptor (erythroblastic leukemia viral (v-erb-b) oncogene homolog, avian)	3352	-0.015	-0.2562	No
28	EPN1	EPN1 Entrez, Source	epsin 1	3398	-0.019	-0.2608	No
29	CDKN1B	CDKN1B Entrez, Source	cyclin-dependent kinase inhibitor 1B (p27, Kip1)	3490	-0.026	-0.2705	No
30	LRIG1	LRIG1 Entrez, Source	leucine-rich repeats and immunoglobulin-like domains 1	3496	-0.026	-0.2702	No
31	ADCY2	ADCY2 Entrez, Source	adenylate cyclase 2 (brain)	3580	-0.035	-0.2787	No
32	PRKAR2A	PRKAR2A Entrez, Source	protein kinase, cAMP-dependent, regulatory, type II, alpha	3673	-0.043	-0.2879	No

33	HSP90AA1	HSP90AA1 Entrez, Source	heat shock protein 90kDa alpha (cytosolic), class A member 1	3719	-0.047	-0.2916	No
34	AP2M1	AP2M1 Entrez, Source	adaptor-related protein complex 2, mu 1 subunit	4116	-0.081	-0.3350	No
35	ITPR3	ITPR3 Entrez, Source	inositol 1,4,5-triphosphate receptor, type 3	4184	-0.087	-0.3398	No
36	MAPKAP1	MAPKAP1 Entrez, Source	mitogen-activated protein kinase associated protein 1	4506	-0.114	-0.3734	No
37	MAPK3	MAPK3 Entrez, Source	mitogen-activated protein kinase 3	4509	-0.114	-0.3697	No
38	CAMK4	CAMK4 Entrez, Source	calcium/calmodulin-dependent protein kinase IV	4574	-0.119	-0.3730	No
39	CASP9	CASP9 Entrez, Source	caspase 9, apoptosis-related cysteine peptidase	4581	-0.119	-0.3696	No
40	GRB2	GRB2 Entrez, Source	growth factor receptor-bound protein 2	4744	-0.134	-0.3838	No
41	SPRY2	SPRY2 Entrez, Source	sprouty homolog 2 (Drosophila)	4802	-0.139	-0.3856	No
42	NRAS	NRAS Entrez, Source	neuroblastoma RAS viral (v-ras) oncogene homolog	4809	-0.140	-0.3815	No
43	SHC1	SHC1 Entrez, Source	SHC (Src homology 2 domain containing) transforming protein 1	5080	-0.164	-0.4073	No
44	CALM1	CALM1 Entrez, Source	calmodulin 1 (phosphorylase kinase, delta)	5346	-0.188	-0.4317	No
45	GAB1	GAB1 Entrez, Source	GRB2-associated binding protein 1	5377	-0.192	-0.4286	No
46	MAP2K1	MAP2K1 Entrez, Source	mitogen-activated protein kinase kinase 1	5558	-0.211	-0.4423	No
47	STAM	STAM Entrez, Source	signal transducing adaptor molecule (SH3 domain and ITAM motif) 1	5624	-0.217	-0.4423	No
48	CREB1	CREB1 Entrez, Source	cAMP responsive element binding protein 1	5833	-0.238	-0.4583	No
49	PRKCE	PRKCE Entrez, Source	protein kinase C, epsilon	5938	-0.251	-0.4618	No

50	MDM2	MDM2 Entrez, Source	Mdm2, transformed 3T3 cell double minute 2, p53 binding protein (mouse)	6102	-0.269	-0.4714	No
51	AKT1S1	AKT1S1 Entrez, Source	AKT1 substrate 1 (proline-rich)	6312	-0.294	-0.4856	No
52	STAM2	STAM2 Entrez, Source	signal transducing adaptor molecule (SH3 domain and ITAM motif) 2	6394	-0.305	-0.4845	No
53	PRKAR1B	PRKAR1B Entrez, Source	protein kinase, cAMP-dependent, regulatory, type I, beta	6602	-0.334	-0.4970	No
54	PRKCA	PRKCA Entrez, Source	protein kinase C, alpha	6631	-0.341	-0.4884	No
55	CALM3	CALM3 Entrez, Source	calmodulin 3 (phosphorylase kinase, delta)	6689	-0.350	-0.4829	No
56	PIK3CA	PIK3CA Entrez, Source	phosphoinositide-3-kinase, catalytic, alpha polypeptide	6801	-0.370	-0.4830	No
57	ADAM17	ADAM17 Entrez, Source	ADAM metallopeptidase domain 17 (tumor necrosis factor, alpha, converting enzyme)	7181	-0.447	-0.5117	Yes
58	ADRBK1	ADRBK1 Entrez, Source	adrenergic, beta, receptor kinase 1	7199	-0.452	-0.4980	Yes
59	ADCY8	ADCY8 Entrez, Source	adenylate cyclase 8 (brain)	7224	-0.457	-0.4849	Yes
60	MAPK1	MAPK1 Entrez, Source	mitogen-activated protein kinase 1	7446	-0.522	-0.4926	Yes
61	ADCY3	ADCY3 Entrez, Source	adenylate cyclase 3	7460	-0.528	-0.4757	Yes
62	SOS1	SOS1 Entrez, Source	son of sevenless homolog 1 (Drosophila)	7606	-0.574	-0.4727	Yes
63	CALM2	CALM2 Entrez, Source	calmodulin 2 (phosphorylase kinase, delta)	7688	-0.604	-0.4611	Yes
64	PRKAR2B	PRKAR2B Entrez, Source	protein kinase, cAMP-dependent, regulatory, type II, beta	7828	-0.668	-0.4541	Yes
65	SH3KBP1	SH3KBP1 Entrez, Source	SH3-domain kinase binding protein 1	7855	-0.678	-0.4336	Yes
66	AKT1	AKT1 Entrez, Source	v-akt murine thymoma viral oncogene homolog 1	8115	-0.817	-0.4354	Yes

67	AKT2	AKT2 Entrez, Source	v-akt murine thymoma viral oncogene homolog 2	8121	-0.821	-0.4074	Yes
68	AP2A2	AP2A2 Entrez, Source	adaptor-related protein complex 2, alpha 2 subunit	8259	-0.929	-0.3911	Yes
69	PRKAR1A	PRKAR1A Entrez, Source	protein kinase, cAMP-dependent, regulatory, type I, alpha (tissue specific extinguisher 1)	8313	-0.980	-0.3632	Yes
70	CBL	CBL Entrez, Source	Cas-Br-M (murine) ecotropic retroviral transforming sequence	8393	-1.070	-0.3352	Yes
71	YWHAB	YWHAB Entrez, Source	tyrosine 3-monooxygenase/tryptophan 5-monooxygenase activation protein, beta polypeptide	8401	-1.077	-0.2985	Yes
72	KRAS	KRAS Entrez, Source	v-Ki-ras2 Kirsten rat sarcoma viral oncogene homolog	8444	-1.153	-0.2633	Yes
73	PIK3R1	PIK3R1 Entrez, Source	phosphoinositide-3-kinase, regulatory subunit 1 (p85 alpha)	8463	-1.179	-0.2244	Yes
74	EPS15	EPS15 Entrez, Source	epidermal growth factor receptor pathway substrate 15	8469	-1.184	-0.1838	Yes
75	SH3GL2	SH3GL2 Entrez, Source	SH3-domain GRB2-like 2	8533	-1.335	-0.1447	Yes
76	AP2B1	AP2B1 Entrez, Source	adaptor-related protein complex 2, beta 1 subunit	8540	-1.348	-0.0985	Yes
77	AKT3	AKT3 Entrez, Source	v-akt murine thymoma viral oncogene homolog 3 (protein kinase B, gamma)	8586	-1.473	-0.0525	Yes
78	PRKACB	PRKACB Entrez, Source	protein kinase, cAMP-dependent, catalytic, beta	8614	-1.677	0.0027	Yes

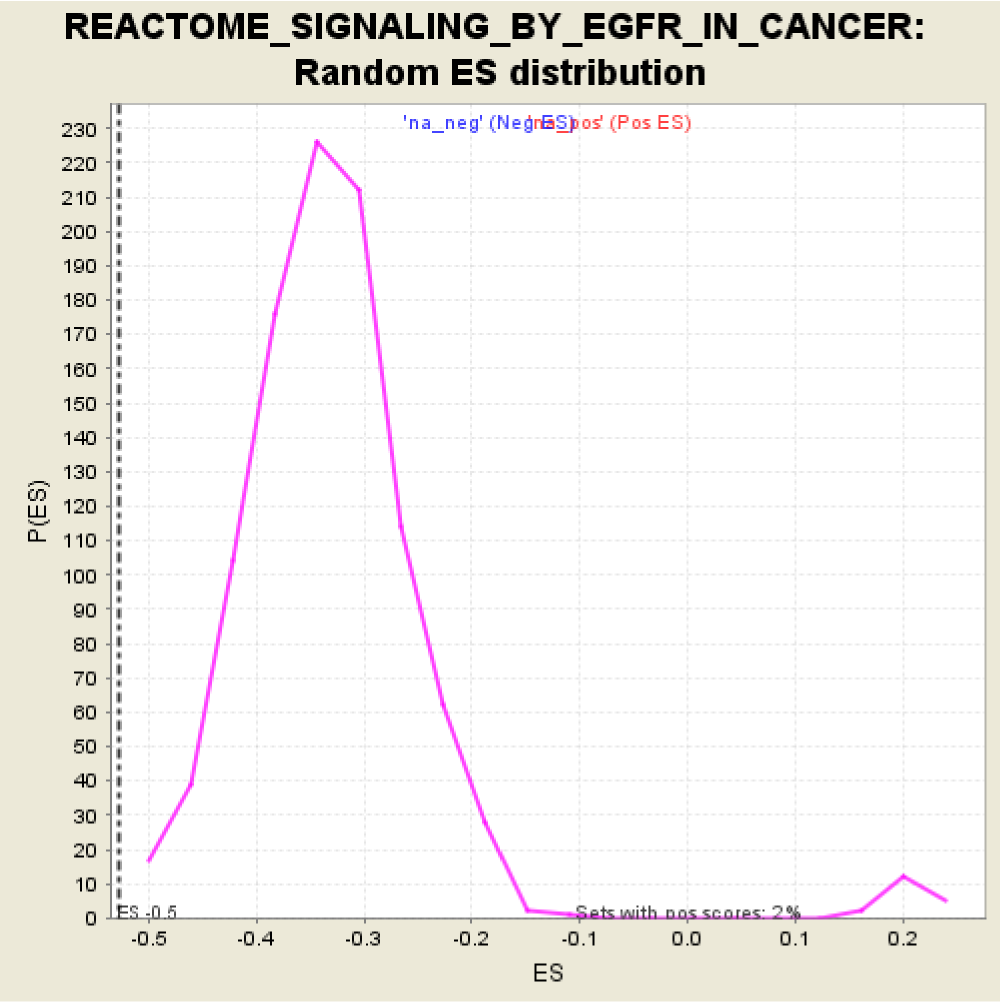

Fig 2: REACTOME_SIGNALING_BY_EGFR_IN_CANCER: Random ES distribution
Gene set null distribution of ES for REACTOME_SIGNALING_BY_EGFR_IN_CANCER
