# Supplementary material for: Progression of pathology in PINK1-deficient mouse brain from splicing via ubiquitination, ER stress, and mitophagy changes to neuroinflammation
Source: J Neuroinflammation. 2017 Aug 2;14:154. doi: 10.1186/s12974-017-0928-0 (PMC5541666; doi:10.1186/s12974-017-0928-0)

(A) Reactome pathway
“Negative regulators of
RIG I and MDA5 signaling”

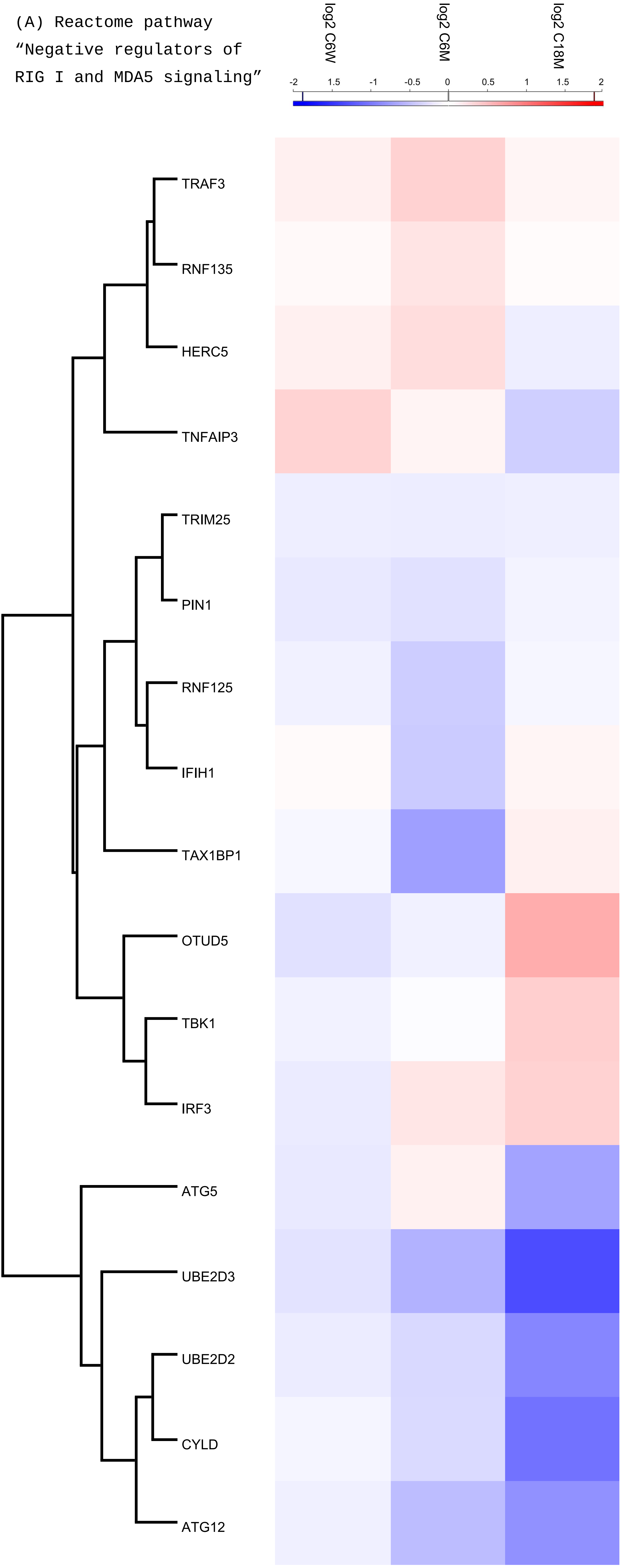

(B) Reactome pathway “Antiviral mechanism by IFN stimulated genes”

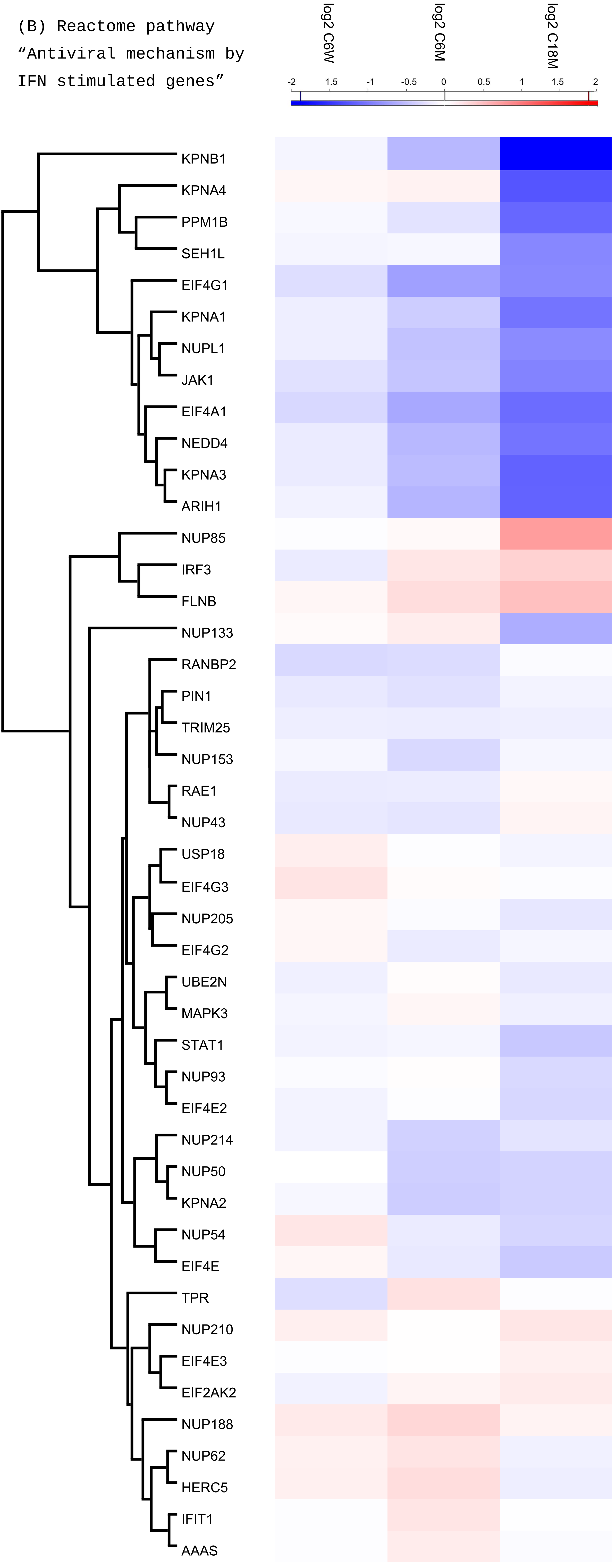

(C)Reactome pathway “Antigen processing ubiquitination proteasome degradation”

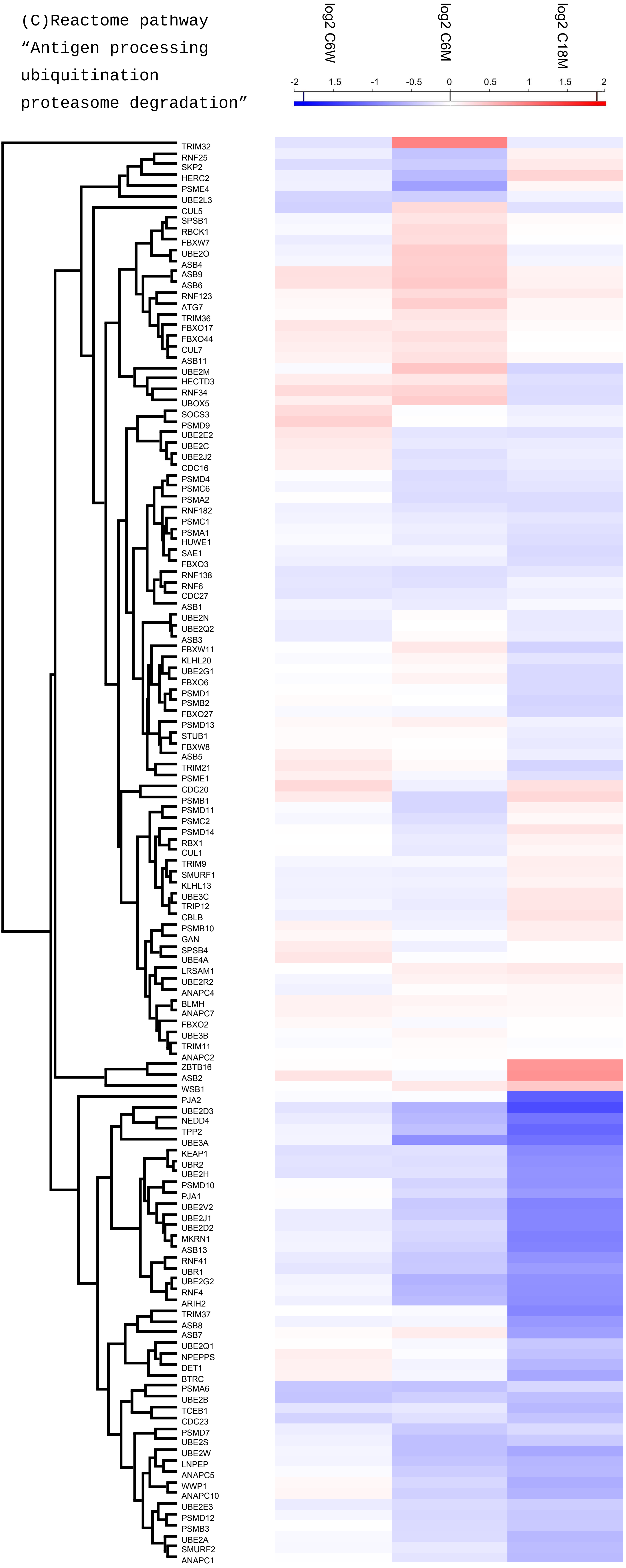

(D) Reactome pathway
“Activation of chaperone genes
by Xbp1s”

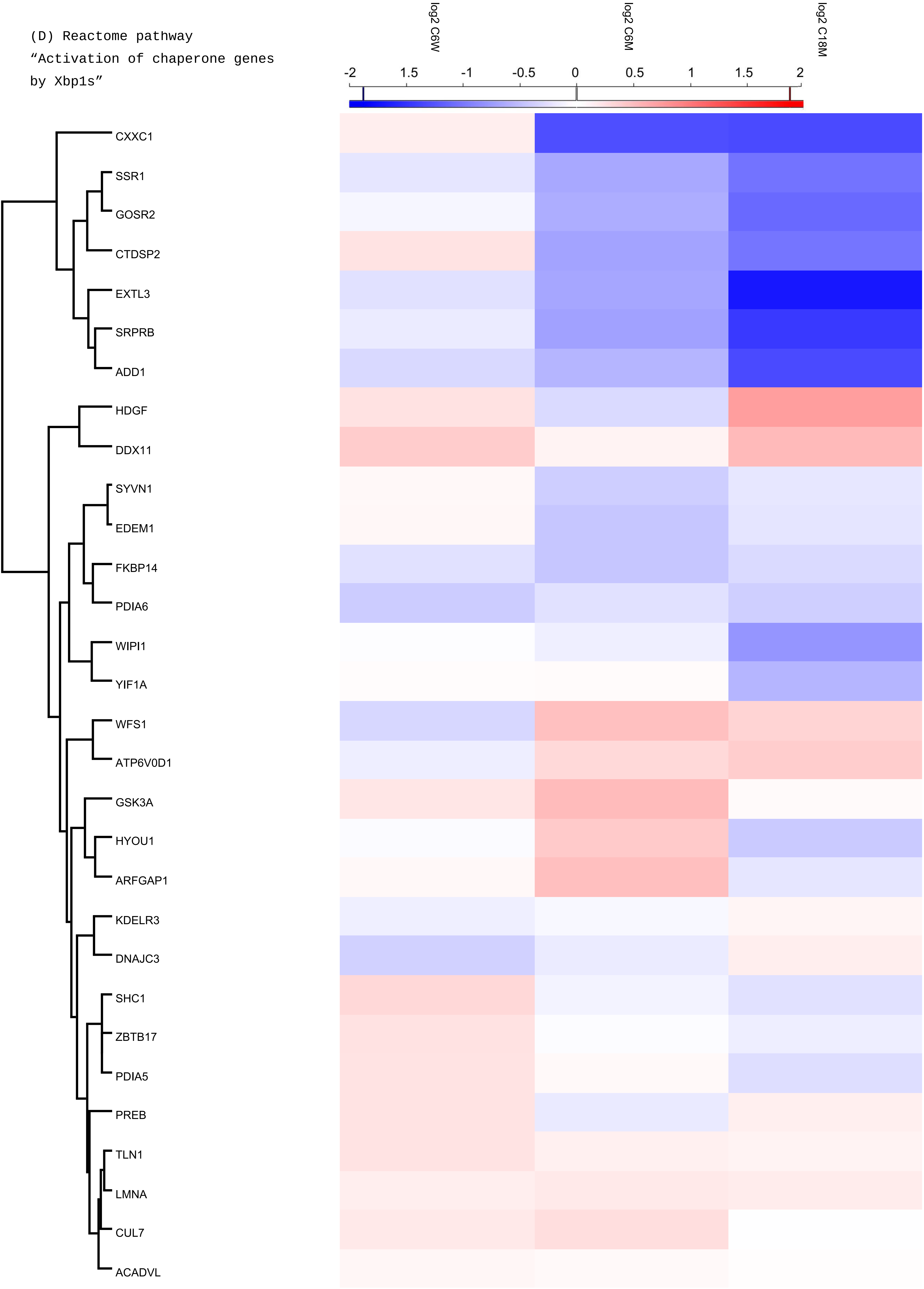

Supplement: Supplementary file 7 — GSEA bioinformatics assessment—heat maps. Heat maps to illustrate the progression of expression dysregulation for all individual components of selected pathways of relevance, namely (A) the Reactome pathway “Negative regulators of RIG I and MDA5 signaling”, (B) the Reactome pathway “Antiviral mechanism by IFN stimulated genes”, (C) the Reactome pathway “Antigen processing ubiquitination proteasome degradation”, and (D) the Reactome pathway “Activation of chaperone genes by Xbp1s”. (PDF 135 kb) [file 12974_2017_928_MOESM7_ESM.pdf]
