# Supplementary material for: Progression of pathology in PINK1-deficient mouse brain from splicing via ubiquitination, ER stress, and mitophagy changes to neuroinflammation
Source: J Neuroinflammation. 2017 Aug 2;14:154. doi: 10.1186/s12974-017-0928-0 (PMC5541666; doi:10.1186/s12974-017-0928-0)
Supplement: Supplementary file 9 — PINK1-deficient transcriptome profile of primary neuron cultures. Ranked list of significant expression changes in global transcriptome profile of primary neuron cultures at 12 days after dissection of brains at postnatal stage, taken from 3 Pink1 −/− and 3 age- and sex- matched WT controls from the same ancestors. FC = fold change. (PDF 60 kb) [file 12974_2017_928_MOESM9_ESM.pdf]

Probe Set ID	Gene Symbol	Chromo-some Number	pvalue ([wt] Vs [ko])	FCAbsolute ([wt] Vs [ko])	Authors' Comments
1451148_at	Pink1	chr4	6,97E-05	48,452423	KO
1441937_s_at	Pink1	chr4	9,74E-05	45,783978	KO
1424524_at	Dram1 - 1200002N14Rik	chr10	1,37E-04	1,7332038	Dram1 DNA-damage regulated autophagy modulator 1; implicated in PARK2 variants of Parkinson's disease (Tatura-R 2016 Parkinsonism Relat Disord)
1452730_at	1110033J19Rik	chr6	2,23E-04	2,0384903	Rps4l ribosomal protein S4-like
1422616_s_at	Wdr54	chr6	5,54E-04	1,7951214	
1429118_a_at	Gin1 - 4930429M06Rik	chr1	6,74E-04	1,78669	Gin1 gypsy retrotransposon integrase 1
1436359_at	Ret	chr6	9,93E-04	1,7081106	PINK1/Parkin interactions with RET: Klein-P 2014 EMBO J for PINK1, Meka-DP 2015 J Clin Invest for Parkin
1436462_at			0,001027774	1,9132199	
1428991_at	Hrasls	chr16	0,001528755	2,8665628	
1448239_at	Hmox1	chr8	0,001866731	1,7795864	cleaves the heme ring and liberates iron; innate immunity factor; crucial for mitochondrial quality control; implicated in Parkinson's disease (Schipper-HM 2015 Int J Mol Sci; Wegliel-B 2015 Free Radic Biol Med; Hull-TD 2016)
1454714_x_at	Phgdh - LOC546010	chr7	0,002202771	124,833885	
1427730_a_at	Zfp148	chr16	0,003365849	1,630802	
1424257_at	Cdk7	chr13	0,004854731	2,1732583	
1432443_at	1700021P22Rik	chr7	0,004899022	1,543802	Golgi-associated Rab2B interactor-like 5
1418172_at	Hebp1	chr6	0,004931319	1,8345174	heme binding protein 1, includes a natural chemoattractant peptide of 21 amino acids at the N-terminus, which is a natural ligand for formyl peptide receptor-like receptor 3 (FPRL3) and promotes calcium mobilization and chemotaxis (Devosse-T 2011)
1456490_at	Cdk2ap2	chr19	0,00539204	1,8029057	
1456565_s_at	Map3k12	chr15	0,005866624	1,8616774	
1451680_at	Srxn1	chr2	0,006438374	1,5373628	oxidative stress resistence
1426766_at	6330403K07Rik	chr11	0,00683536	1,8228908	UGS148, involved in intracellular sorting, trafficking, exocytosis and membrane insertion
1417602_at	Per2	chr1	0,006929158	1,5086643	
1434735_at	Hlf	chr11	0,008153346	1,5317298	upstream of HIF1alpha
1429241_at	Npal3	chr4	0,008624177	2,5109963	
1424938_at	Steap1	chr5	0,009155522	1,9902649	Metalloreductase that has the ability to reduce both $Fe(3+)$ to $Fe(2+)$ and $Cu(2+)$ to $Cu(1+)$. Uses NAD(+) as acceptor
1432372_a_at	Spr	chr6	0,009455642	1,6994692	Dopa-responsive dystonia and Parkinsonism
1432217_a_at	1700019F09Rik	chr11	0,009735948	2,368865	WDRPUH, with WD40 repeat, cilia- and flagella-associated protein 52
1431708_a_at	Tia1	chr6	0,010277364	4,357368	starvation regulator at stress granules
1430837_a_at	Mbd1	chr18	0,010326727	3,3089137	Regulator Of Fibroblast Growth Factor 2 (FGF-2) Transcription
1437753_at			0,010944241	1,5809302	
1436045_at	Tsga10	chr1	0,010945085	1,9227682	prevents nuclear localization of HIF1alpha
1456256_at	Eif5	chr12	0,011162037	1,6740198	
1456041_at	Snx16	chr3	0,013978108	1,5146377	promotes degradation of EGFR after stimulation, intracellular transport of vesicular stomatitis virus nucleocapsids (Le Blanc-I 2005 Nat Cell Biol)
1421009_at	Rsad2	chr12	0,014488435	1,7024405	Interferon-inducible iron-sulfur (4FE-4S) cluster-binding antiviral protein (Chen-WQ 2015 Dev Comp Immunol)
1442165_at	Armc9	chr1	0,015487114	1,840675	
1449862_a_at	Pi4k2b	chr5	0,016058756	1,8411335	
1422256_at	Sstr2	chr11	0,017115435	1,7433645	mediates negative regulation of insulin receptor signaling
1452992_at	Cdc26	chr4	0,017132841	1,52979	Component of APC/C complex, a cell cycle-regulated E3 ubiquitin ligase
1455279_at	Gm1060	chr5	0,018250158	2,1300688	
1432189_a_at	Sox5	chr6	0,01899819	1,6616669	
1460227_at	Timp1	chrX	0,019330187	1,5041677	inducible by cytokines and toll-like receptors (Li-L 2014 Mol Cell Biochem; Swanberg-M 2006 Neurobiol Dis)
1450432_s_at	Mus81	chr19	0,019486142	1,5867147	Up-regulated in cells treated with agents that damage DNA or block replication
1434894_at	3110050K21Rik	chr14	0,019493764	1,6834528	Zc3h13 zinc finger CCCH type containing 13
1436519_a_at	1110057K04Rik	chr12	0,019745862	1,5311022	Ldah lipid droplet associated hydrolase
1423667_at	Mat2a	chr6	0,020037618	1,6003249	hypoxia induces DNA demethylation through Mat2a upregulation
1456471_x_at	LOC224870 - Phgdh	chr3	0,021097671	22,651728	
1427524_a_at	4930548G07Rik	chr14	0,021225194	1,5558258	Mphosph8 M-phase phosphoprotein 8
1451621_at	5830417C01Rik	chr1	0,021344382	1,6901493	Desi2 desumoylating isopeptidase 2
1423500_a_at	Sox5	chr6	0,022289125	1,5124487	
1455064_at	Rab36	chr10	0,02343256	1,7044375	
1457264_at	Phf20l1	chr15	0,023548324	1,7924849	influences DNA cytosine methylation via DNMT1 (Estève-PO 2014 J Biol Chem)
1421752_a_at	Serpinb5	chr1	0,023890194	1,5496114	tumor suppressor, interactor of interferon regulatory factor 6
1422725_at	Mak	chr13	0,024152678	2,2695055	

1424253_at	1810073G14Rik	chr11	0,024282003	1,6238674	
1421441_at	Angpt1	chr15	0,024920227	1,5130731	
1435688_at			0,02493906	2,226304	
1460006_at	Atbf1	chr8	0,02682056	1,7013267	
1417988_at	Resp18	chr1	0,0270382	2,6684775	RESP18 is involved in the cytotoxicity of dopaminergic neurotoxins in MN9D cells, overexpression aggravated cell death induced by MPP+ and 6OH-DA
1435036_at	A530050D06Rik	chr12	0,027661307	1,7793468	Aspg asparaginase homolog
1425191_at	9430098E02Rik	chr8	0,029728629	1,5816218	Ocel1 occludin/ELL domain containing 1, NRF1 target like PINK1
1419749_at	Dnmt2	chr2	0,029773517	1,5979004	cytosine methylation of RNA and DNA; role in stress granules represents a defense mechanism against viral infection (Schaefer-M 2010 Genes Dev)
1427133_s_at	LOC545422	chr2	0,030742085	1,5075078	
1434510_at	Papss2	chr19	0,030916411	1,6075696	sulfation activation pathway; expression regulated by lipopolysaccharide treatment; neighbor gene of PTEN (Kim-MS 2004 Am J Physiol Endocrinol Metab; Marbiah-MM 2014 EMBO J; Liu-Z 2016 Free Radic Biol Med)
1436058_at	Rsad2	chr12	0,03116718	1,7421006	Interferon-inducible iron-sulfur (4FE-4S) cluster-binding antiviral protein (Seo-JY 2011 Cell Host Microbe; Chen-WQ 2015 Dev Comp Immunol)
1421141_a_at	Foxp1	chr6	0,031862367	1,7421789	modulated by alpha-synuclein, responsible for DAergic midbrain neurons
1431630_a_at	Klf3	chr5	0,032135975	1,8721375	Binds to the CACCC box of erythroid cell-expressed genes, regulated by Interleukin-17, regulates inflammatory Galectin-3 (Ahmed-M 2013 Cytokine; Knights-AJ 2016 J Biol Chem)
1455260_at	A830039H10Rik	chr5	0,032324962	1,5346051	
1429380_at	Rgs12	chr5	0,03331049	1,7056541	Ras/Raf/Mek-scaffold in nerve-growth-factor-mediated differentiation
1434929_at	BC035044	chr6	0,033569746	1,6217262	
1453470_a_at	Gna13	chr11	0,033591755	1,5725456	interacts with RhoGEF
1426631_at	C330017I15Rik	chr5	0,03389128	1,5001372	Pus7 pseudouridylate synthase 7 homolog
1441306_at	6820408C15Rik	chr2	0,03397604	2,9458206	
1429588_at	2810474O19Rik	chr6	0,034188952	1,9378095	
1452595_at	Adamts4	chr1	0,03418908	1,8432097	induced by interleukin-1, repressed by hyaluronan (Bondeson-J 2007 J Pheumatol; Yaikasli-KO 2009 Mol Cell Biochem; Yatabe-T 2009 Ann Rheum Dis)
1435642_at	9630019K15Rik	chr17	0,034402315	2,4065404	Prr18, proline rich 18
1449390_at	Gpatc4	chr3	0,035351932	1,5143058	
1423997_at	Csde1	chr3	0,03547374	1,7396368	synonymous to UNR; internal initiation of translation of virus RNA; regulation of c-Fos RNA (Grosset-C 2000 Cell; Boussadia-O 2003 J Virol)
1426114_at	Hnrpab	chr11	0,03590401	1,5990748	binds single-stranded RNA
1439582_at	Macf1	chr4	0,03621851	1,9254653	Wnt signaling pathway of wound healing
1434315_at	Npal3	chr4	0,036787618	1,8032653	
1452139_at	Slc35c1	chr2	0,037324883	1,6265639	GDP-fucose import from the cytoplasm into the Golgi lumen
1422919_at	Hrasls	chr16	0,03812369	1,9519243	
1435529_at	Ifit3	chr19	0,038313616	1,5106372	enhances MAVS-mediated host antiviral responses, as adaptor leading to activation of TNFR-associated factor family member-associated NF-κB activator-binding kinase 1 (TBK1) (Liu-XY 2011 J Immunol)
1419069_at	Rabgef1	chr5	0,03857006	1,606571	
1427942_at	Pamci	chr10	0,03936968	1,8311158	endosomal RASSF9
1422253_at	Col10a1	chr10	0,039610066	2,9920065	
1421321_a_at	Net1	chr13	0,040916238	1,6481261	Rho-GEF Proto-Oncogene P65, induced by TGFB1, in JNK pathway
1434868_at	4933431E20Rik	chr3	0,041321814	1,808017	
1429566_a_at	Hipk2	chr6	0,041372046	1,5986654	response to hypoxia by acting as a transcriptional co-suppressor of HIF1A, involved in p53/TP53-mediated cellular apoptosis
1448566_at	Slc40a1	chr1	0,041440252	1,9830165	iron-regulated transporter; regulated by toll-like receptors and lipopolysaccharides; dysregulated in Parkinson's disease (Layoun-A 2012 Am J Pathol; Rhodes-SL 2014 Neurobiol Dis)
1417481_at	Ramp1	chr1	0,0420383	1,9016539	
1425349_a_at	Myef2	chr2	0,042601384	2,0290363	
1428751_at	Pacrg	chr17	0,044120647	1,5269926	
1424966_at	Tmem40	chr6	0,044930708	1,5544113	
1448417_at	Ninj1	chr13	0,045032132	1,5059755	upregulated after nerve injury
1419420_at	St6galnac5	chr3	0,0450764	1,626536	
1422033_a_at	Cntf	chr19	0,045293726	1,5433439	Liu-J 2010 Proteomics, von Bohlen und Halbach-O 2009 Adv Exp Med Biol
1429217_at	Zfp655	chr5	0,045893386	1,7568653	
1428599_at	2410012C07Rik	chr7	0,04663539	1,507578	
1435069_at	BC064078	chr6	0,046687637	1,6245518	
1455147_at			0,04702163	1,697666	
1424098_at	Elovl7	chr13	0,04778445	1,5016968	Elongation Of Very Long Chain Fatty Acids

1417618_at	Itih2	chr2	0,048334748	2,5506465	
1445404_at	Kif27	chr13	0,04843511	1,5450956	
1440147_at	Lgi2	chr5	0,0489945	2,0282414	phenotype epilepsy
1429059_s_at	1110004B13Rik	chr11	0,049165368	1,7148007	Tmem107 transmembrane protein 107
1453734_at	Atrx	chrX	0,049633674	1,5523869	PML nuclear body component involved in antiviral defense (Scherer-M 2016 J Virol)
1427944_at	C1qdc1	chr6	0,049639553	1,6935508	

Color coding:
Pink1-KO
trophic factors
already implicated in neurodegenerative processes
DNA/RNA quality control
hypoxia/antioxidant factors
autophago-lysosomal modulator
vesicle trafficking
anti-microbial state factors
